# Dataset on the effect of the reaction temperature during spray pyrolysis for the synthesis of the hierarchical yolk-shell CNT-(NiCo)O/C microspheres

**DOI:** 10.1016/j.dib.2019.104302

**Published:** 2019-07-24

**Authors:** Se Hwan Oh, Jung Sang Cho

**Affiliations:** Department of Engineering Chemistry, Chungbuk National University, Chungbuk, 361-763, Republic of Korea

**Keywords:** Reaction temperature, Spray pyrolysis, CNT, Anodes, Lithium ion batteries

## Abstract

The data presented in this article are related to the research article entitled “Hierarchical yolk-shell CNT-(NiCo)O_C microspheres prepared by one-pot spray pyrolysis as anodes in lithium-ion batteries” (Oh et al., 2019). The data presented in this manuscript showed the effect of the reaction temperature during spray pyrolysis on the obtained microspheres morphology. Each morphology and phase of the microspheres obtained after spray pyrolysis were investigated.

**Table:** Specifications table

Subject area	*Chemistry*
More specific subject area	*Inorganic chemistry*
Type of data	*Figures*
How data was acquired	*FE-SEM (JEOL, JSM-6060), XRD(X'Pert PRO MPD, PANalytical)*
Data format	*Raw, analyzed data*
Experimental factors	*Reaction temperature during spray pyrolysis*
Experimental features	*Microspheres with various morphologies and phases obtained after spray pyrolysis*
Data source location	*Cheongju, Republic of Korea*
Data accessibility	*Data included in this article*
Related research article	*S. H. Oh, M. S. Jo, S. M. Jeong, Y. C. Kang, J. S. Cho, Hierarchical yolk-shell CNT-(NiCo)O/C microspheres prepared by one-pot spray pyrolysis as anodes in lithium-ion batteries, Chemical Engineering Journal,**https://doi.org/10.1016/j.cej.2019.02.144*

**Table:** 

**Value of the data** • These data provide the appropriate reaction temperature to obtain the hierarchical yolk–shell structured microspheres by using spray pyrolysis process. • To understand the effect of the reaction temperature on the obtained powder morphology and phase. • These data could be applied to prepare other metal compounds with hierarchical yolk–shell structure.

## 1 Data

**Fig. 1:**
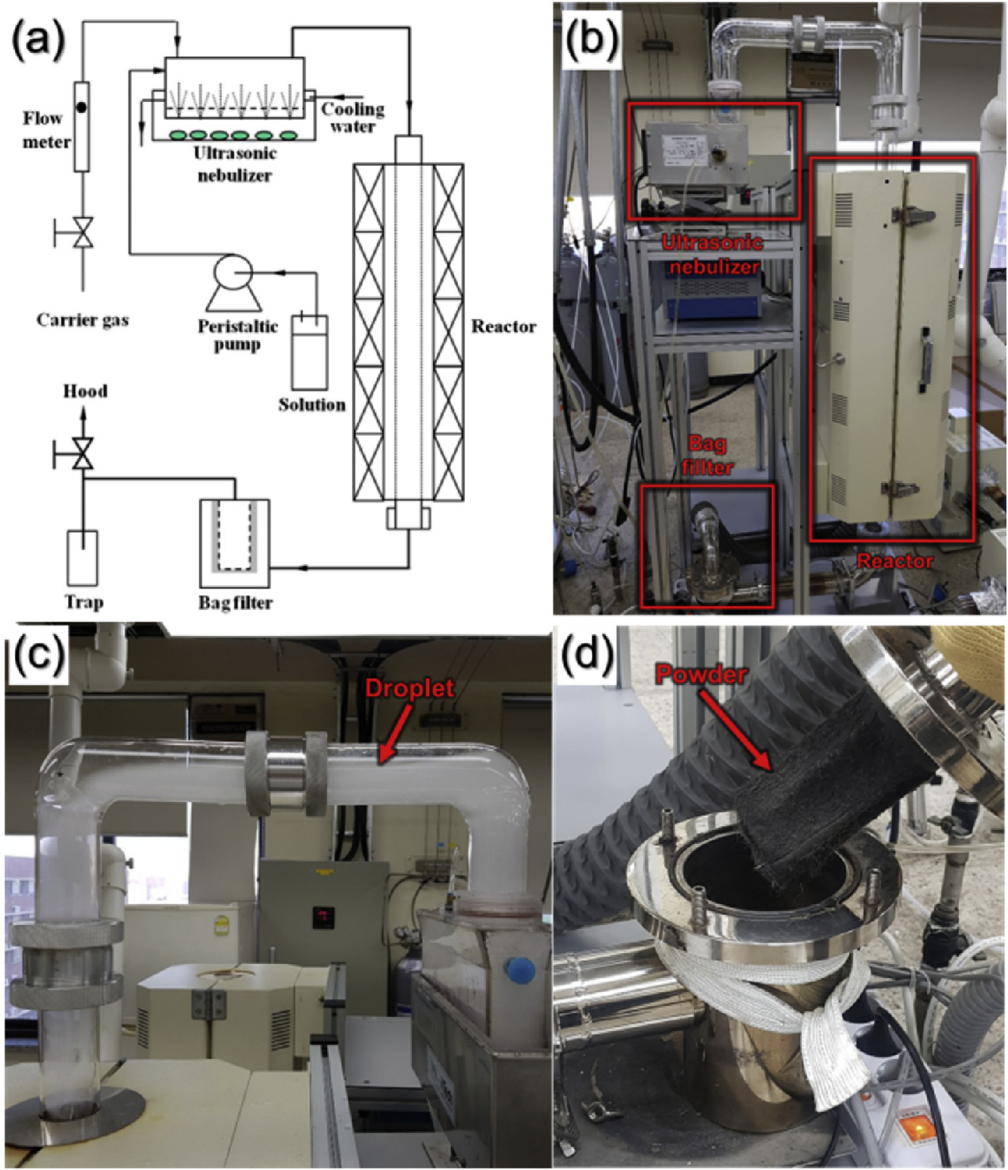
(a) Schematic diagram, (b) digital photo of spray pyrolysis process applied in the preparation of the microspheres, and the detailed photos of (c) generated droplet and (d) collected powders on the bag filter.

**Fig. 2:**
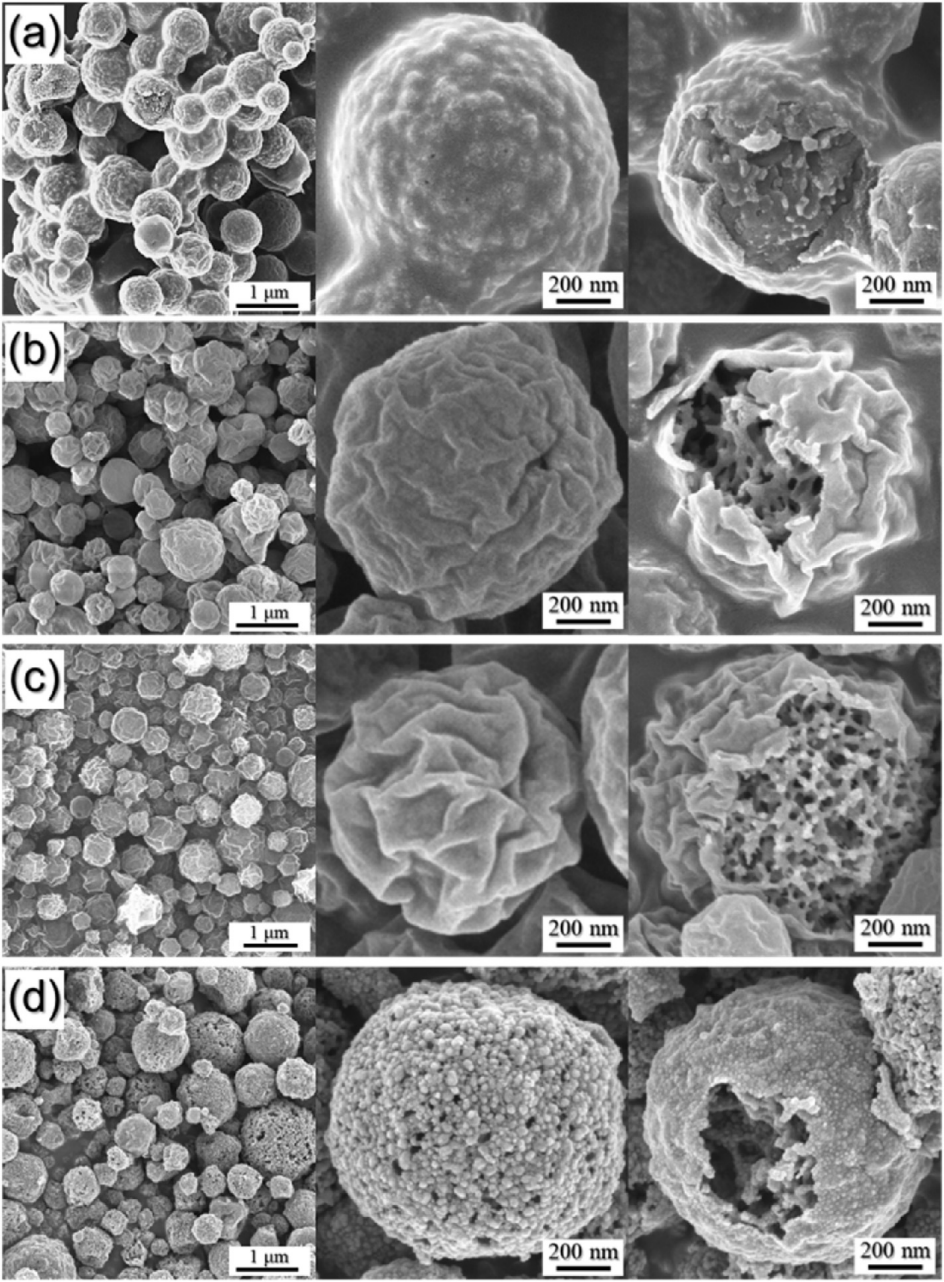
FE-SEM images of the microspheres obtained at various reaction temperatures: (a) 400 °C, (b) 550 °C, (c) 700 °C, and (d) 850 °C.

**Fig. 3:**
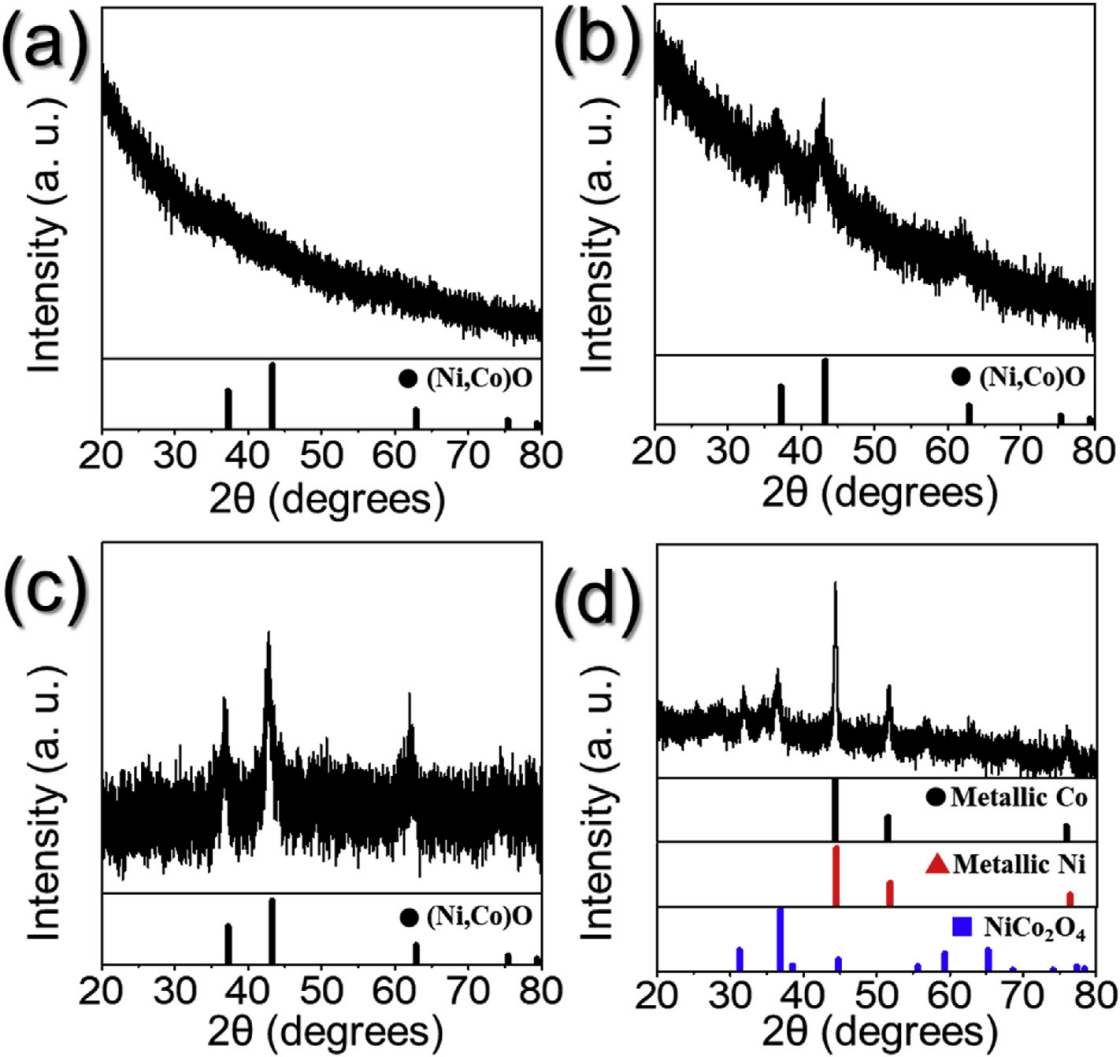
XRD patterns of the microspheres obtained at various reaction temperatures during spray pyrolysis: (a) 400 °C, (b) 550 °C, (c) 700 °C, and (d) 850 °C.

The data presented in this manuscript were generated from a study that showed the effect of reaction temperature during spray pyrolysis on the morphology and phase of the resulting microspheres. Fig. 1 shows the schematic diagram and digital photo of spray pyrolysis process applied in the preparation of the microspheres. Fig. 2 shows the FE-SEM images of the microspheres obtained at various reaction temperatures. Fig. 3 shows the XRD patterns of the microspheres obtained at various reaction temperatures during spray pyrolysis.

## 2 Experimental design, materials, and methods

Spray pyrolysis process was applied to prepare the hierarchical yolk–shell structured microspheres [1]. A schematic diagram and digital photo of spray pyrolysis process applied in the preparation of the microspheres were shown in Fig. 1a and b. The spray pyrolysis was composed of ultrasonic nebulizer for generating droplets, reactor for synthesizing particles, and bag filter for collecting particles. Piezoelectric vibration exerted on the liquid generates droplets. The aerosol stream is moved to the reactor by N_2_ carrier gas. In the reactor, the solvent evaporates and precipitation starts to occur from the outer shell. The precipitated precursors are decomposed. Nucleation and growth occur to form a particle subsequently. Finally, particles moved to bag filter and are collected [2], [3].

To understand the relationship between the reaction temperature during spray pyrolysis and the obtained microspheres, the microspheres were synthesized at different reaction temperatures (400–850 °C) using the solution containing optimum amount of Ni–Co salt, PVP, CNTs, and PS nanobeads, used in the work [1]. On increasing the reaction temperature during the spray pyrolysis, the decomposition of the Ni–Co salt, PVP, and PS nanobeads occurred sequentially. The microspheres obtained at 400 °C had rough powder surface due to containing PS nanobeads in the composite (Fig. 2a). The PS nanobeads were then decomposed and formed numerous mesopores inside the composite when the reaction temperature increased to 550 °C (Fig. 2b). The wavy powder surfaces were also shown in the microspheres obtained at 550 °C and 700 °C (Fig. 2b and c). The microspheres obtained at 850 °C showed large grains on the powder surface due to the grain growth at high reaction temperature of 850 °C.

The phases of the microspheres obtained at various reaction temperatures during spray pyrolysis were shown in Fig. 3. The microspheres obtained at 400 °C showed amorphous-like broad peak owing to the low reaction temperature. On increasing the reaction temperature, the microspheres obtained at 550 and 700 °C had a pure phase of (Ni, Co)O solid-solution. The microspheres obtained at 850 °C had mixed phases of NiCo_2_O_4_, metallic Ni, and Co, in which the metallic Ni and Co was formed due to carbothermal reduction at a high temperature.
