# Supplementary material for: Dataset on the effect of the reaction temperature during spray pyrolysis for the synthesis of the hierarchical yolk-shell CNT-(NiCo)O/C microspheres
Source: Data Brief. 2019 Jul 24;25:104302. doi: 10.1016/j.dib.2019.104302 (PMC6685701; doi:10.1016/j.dib.2019.104302)
Supplement: Supplementary file 1 [file mmc1.docx]

**Dataset on the effect of the reaction temperature during spray pyrolysis for the synthesis of the hierarchical yolk-shell CNT-(NiCo)O/C microspheres**

Se Hwan Oh, Jung Sang Cho*

Department of Engineering Chemistry, Chungbuk National University, Gaesin-Dong, Seowon-Gu, Cheongju-Si, Chungchengbuk-Do 361-763 Republic of Korea

*Corresponding authors.

E-mail: jscho@cbnu.ac.kr Tel.: +82-43-261-2489. Fax: +82-43-262-2380. (Jung Sang Cho)

**Raw data 1**. XRD raw data of microsphere obtained spray pyrolysis at 400 ℃.

; (content of file C:\DATA\CBNU_IN\ENG-Chemical\JoJungSang\Oh Sehwan\190104\Ni-Co_400C.raw)

_FILEVERSION = 2

_SAMPLE =

_+SAMPLE =

_SITE = Korea

_USER = "Chungbuk UNI."

_GONIOMETER_CODE = 21

; Goniometer : D8 theta/theta, stage : Unknown

_SAMPLE_CHANGER_CODE = 0

_ATTACHMENTS_CODE = 0

_GONIOMETER_RADIUS = 250

_FIXED_DIVSLIT = 0.6

_FIXED_SAMPLESLIT = 0

_FIXED_DETSLIT = 12.21

_MONOCHROMATOR = 0

; Incident beam monochromator : None

_THIN_FILM = N

_BETA_FILTER = N

_FIXED_ANTISLIT = 8.46

_ANALYZER_CODE = 4

; Received beam analyzer : Gobel mirror

_DATEMEASURED = "04-Jan-2019 14:31:29"

_WL_UNIT = A

_WL1 = 1.5406

_WL2 = 1.54439

_WL3 = 1.39222

_WLRATIO = 0.5

_ANODE = Cu

; Data for range 1

_DRIVE = COUPLED

_STEPTIME = 35.8

_STEPSIZE = 0.00908485

_STEPMODE = C

_START = 10

_THETA = 5

_2THETA = 10

_KHI = 0

_PHI = 0

_X = 0

_Y = 0

_Z = 0

_DETECTOR = 5

; Detector type : Unknown

_DETECTORSLIT = out

_AUX1 = 0

_AUX2 = 0

_AUX3 = 0

_TIMESTARTED = 14

_TEMP_RATE = -1

_TEMP_DELAY = -1

_KV = 40

_MA = 40

_RANGE_WL = 1.5406

_3DPLANE = 0

_V4_COUNTERS_MASK = 4096

_V4_DRIVES_MASK = 0

_V4_ENCODERS_MASK = 0

_2THETACOUNTS = 1

; 2THETA PSD

10.0000 1037

10.0091 1046

10.0182 1026

10.0273 1018

10.0363 1006

10.0454 1046

10.0545 1055

10.0636 1011

10.0727 1076

10.0818 1029

10.0908 1039

10.0999 1003

10.1090 1066

10.1181 1056

10.1272 1079

10.1363 1048

10.1454 1025

10.1544 1064

10.1635 1034

10.1726 1033

10.1817 1061

10.1908 1052

10.1999 1040

10.2090 1046

10.2180 1068

10.2271 1077

10.2362 1024

10.2453 979

10.2544 1060

10.2635 1041

10.2725 1032

10.2816 1045

10.2907 1046

10.2998 1083

10.3089 1019

10.3180 1010

10.3271 1034

10.3361 1087

10.3452 1055

10.3543 1111

10.3634 977

10.3725 1037

10.3816 1096

10.3906 1067

10.3997 1040

10.4088 1037

10.4179 1082

10.4270 1033

10.4361 986

10.4452 1069

10.4542 983

10.4633 1069

10.4724 1002

10.4815 1049

10.4906 1016

10.4997 1039

10.5088 1020

10.5178 1025

10.5269 1031

10.5360 1039

10.5451 1008

10.5542 999

10.5633 1064

10.5723 1062

10.5814 984

10.5905 1063

10.5996 981

10.6087 987

10.6178 1101

10.6269 994

10.6359 1020

10.6450 1002

10.6541 1011

10.6632 1046

10.6723 985

10.6814 1018

10.6904 1018

10.6995 988

10.7086 1052

10.7177 1011

10.7268 1067

10.7359 1022

10.7450 970

10.7540 1029

10.7631 997

10.7722 1005

10.7813 981

10.7904 1055

10.7995 1006

10.8086 1031

10.8176 1011

10.8267 982

10.8358 1049

10.8449 995

10.8540 1014

10.8631 984

10.8721 1042

10.8812 995

10.8903 986

10.8994 961

10.9085 1008

10.9176 1017

10.9267 1019

10.9357 937

10.9448 980

10.9539 1076

10.9630 1026

10.9721 1056

10.9812 1011

10.9902 1017

10.9993 1010

11.0084 971

11.0175 1036

11.0266 1021

11.0357 1041

11.0448 1061

11.0538 1024

11.0629 967

11.0720 1003

11.0811 1031

11.0902 1001

11.0993 1035

11.1084 992

11.1174 966

11.1265 989

11.1356 1038

11.1447 1034

11.1538 967

11.1629 997

11.1719 1026

11.1810 971

11.1901 989

11.1992 971

11.2083 1056

11.2174 1015

11.2265 997

11.2355 1008

11.2446 963

11.2537 961

11.2628 978

11.2719 955

11.2810 1059

11.2900 966

11.2991 1004

11.3082 988

11.3173 1016

11.3264 996

11.3355 1007

11.3446 980

11.3536 1012

11.3627 1026

11.3718 984

11.3809 952

11.3900 953

11.3991 962

11.4082 992

11.4172 1028

11.4263 968

11.4354 998

11.4445 1030

11.4536 969

11.4627 980

11.4717 1015

11.4808 1009

11.4899 1009

11.4990 1002

11.5081 1006

11.5172 1035

11.5263 1000

11.5353 928

11.5444 996

11.5535 995

11.5626 1012

11.5717 995

11.5808 1028

11.5898 958

11.5989 973

11.6080 1020

11.6171 1008

11.6262 977

11.6353 950

11.6444 998

11.6534 984

11.6625 965

11.6716 942

11.6807 990

11.6898 954

11.6989 996

11.7080 978

11.7170 956

11.7261 973

11.7352 967

11.7443 991

11.7534 1003

11.7625 1026

11.7715 1015

11.7806 927

11.7897 990

11.7988 1002

11.8079 1015

11.8170 1014

11.8261 1021

11.8351 1012

11.8442 1028

11.8533 989

11.8624 944

11.8715 1032

11.8806 944

11.8896 916

11.8987 944

11.9078 989

11.9169 974

11.9260 975

11.9351 964

11.9442 989

11.9532 942

11.9623 987

11.9714 936

11.9805 1030

11.9896 971

11.9987 964

12.0078 975

12.0168 992

12.0259 973

12.0350 947

12.0441 1008

12.0532 910

12.0623 1005

12.0713 960

12.0804 957

12.0895 983

12.0986 981

12.1077 956

12.1168 981

12.1259 925

12.1349 1026

12.1440 993

12.1531 969

12.1622 1034

12.1713 955

12.1804 1019

12.1894 945

12.1985 999

12.2076 1010

12.2167 965

12.2258 978

12.2349 947

12.2440 964

12.2530 928

12.2621 918

12.2712 956

12.2803 958

12.2894 905

12.2985 979

12.3076 973

12.3166 919

12.3257 975

12.3348 945

12.3439 937

12.3530 935

12.3621 955

12.3711 930

12.3802 958

12.3893 949

12.3984 929

12.4075 943

12.4166 981

12.4257 922

12.4347 1004

12.4438 929

12.4529 989

12.4620 959

12.4711 975

12.4802 1007

12.4892 989

12.4983 969

12.5074 953

12.5165 926

12.5256 945

12.5347 976

12.5438 970

12.5528 929

12.5619 926

12.5710 935

12.5801 943

12.5892 927

12.5983 982

12.6074 971

12.6164 983

12.6255 911

12.6346 950

12.6437 891

12.6528 931

12.6619 932

12.6709 961

12.6800 974

12.6891 967

12.6982 914

12.7073 954

12.7164 936

12.7255 946

12.7345 965

12.7436 903

12.7527 899

12.7618 903

12.7709 910

12.7800 935

12.7890 933

12.7981 998

12.8072 992

12.8163 993

12.8254 896

12.8345 985

12.8436 912

12.8526 893

12.8617 933

12.8708 912

12.8799 975

12.8890 900

12.8981 944

12.9072 928

12.9162 910

12.9253 951

12.9344 915

12.9435 956

12.9526 926

12.9617 921

12.9707 922

12.9798 941

12.9889 918

12.9980 881

13.0071 989

13.0162 900

13.0253 936

13.0343 917

13.0434 935

13.0525 857

13.0616 914

13.0707 901

13.0798 865

13.0888 924

13.0979 961

13.1070 924

13.1161 937

13.1252 893

13.1343 937

13.1434 916

13.1524 951

13.1615 980

13.1706 951

13.1797 868

13.1888 906

13.1979 957

13.2070 919

13.2160 932

13.2251 956

13.2342 818

13.2433 928

13.2524 887

13.2615 933

13.2705 928

13.2796 878

13.2887 900

13.2978 873

13.3069 994

13.3160 898

13.3251 913

13.3341 873

13.3432 960

13.3523 842

13.3614 909

13.3705 874

13.3796 938

13.3886 881

13.3977 973

13.4068 924

13.4159 893

13.4250 946

13.4341 917

13.4432 918

13.4522 931

13.4613 903

13.4704 860

13.4795 947

13.4886 948

13.4977 940

13.5068 908

13.5158 926

13.5249 887

13.5340 859

13.5431 900

13.5522 939

13.5613 904

13.5703 908

13.5794 924

13.5885 967

13.5976 900

13.6067 904

13.6158 893

13.6249 870

13.6339 884

13.6430 937

13.6521 897

13.6612 938

13.6703 907

13.6794 878

13.6884 903

13.6975 897

13.7066 933

13.7157 906

13.7248 867

13.7339 920

13.7430 914

13.7520 912

13.7611 924

13.7702 922

13.7793 888

13.7884 873

13.7975 871

13.8066 847

13.8156 926

13.8247 927

13.8338 897

13.8429 874

13.8520 937

13.8611 876

13.8701 871

13.8792 886

13.8883 874

13.8974 900

13.9065 885

13.9156 896

13.9247 905

13.9337 933

13.9428 878

13.9519 929

13.9610 928

13.9701 912

13.9792 891

13.9882 953

13.9973 896

14.0064 892

14.0155 878

14.0246 919

14.0337 937

14.0428 876

14.0518 834

14.0609 980

14.0700 919

14.0791 933

14.0882 879

14.0973 911

14.1064 819

14.1154 924

14.1245 916

14.1336 880

14.1427 944

14.1518 889

14.1609 903

14.1699 909

14.1790 890

14.1881 861

14.1972 848

14.2063 862

14.2154 881

14.2245 878

14.2335 853

14.2426 875

14.2517 873

14.2608 932

14.2699 930

14.2790 891

14.2880 958

14.2971 908

14.3062 859

14.3153 909

14.3244 910

14.3335 850

14.3426 880

14.3516 938

14.3607 905

14.3698 912

14.3789 814

14.3880 881

14.3971 902

14.4062 882

14.4152 917

14.4243 905

14.4334 910

14.4425 938

14.4516 907

14.4607 931

14.4697 900

14.4788 914

14.4879 905

14.4970 868

14.5061 881

14.5152 843

14.5243 870

14.5333 906

14.5424 885

14.5515 882

14.5606 858

14.5697 897

14.5788 850

14.5878 863

14.5969 885

14.6060 910

14.6151 885

14.6242 927

14.6333 843

14.6424 843

14.6514 849

14.6605 908

14.6696 888

14.6787 859

14.6878 906

14.6969 853

14.7060 902

14.7150 884

14.7241 880

14.7332 874

14.7423 920

14.7514 843

14.7605 857

14.7695 885

14.7786 872

14.7877 881

14.7968 861

14.8059 885

14.8150 865

14.8241 878

14.8331 913

14.8422 843

14.8513 871

14.8604 868

14.8695 865

14.8786 880

14.8876 887

14.8967 887

14.9058 871

14.9149 893

14.9240 840

14.9331 844

14.9422 859

14.9512 855

14.9603 883

14.9694 847

14.9785 906

14.9876 885

14.9967 904

15.0058 919

15.0148 850

15.0239 894

15.0330 843

15.0421 868

15.0512 893

15.0603 888

15.0693 881

15.0784 871

15.0875 882

15.0966 865

15.1057 891

15.1148 857

15.1239 891

15.1329 925

15.1420 882

15.1511 874

15.1602 949

15.1693 840

15.1784 886

15.1874 866

15.1965 860

15.2056 863

15.2147 857

15.2238 814

15.2329 913

15.2420 845

15.2510 898

15.2601 915

15.2692 811

15.2783 874

15.2874 860

15.2965 915

15.3056 913

15.3146 880

15.3237 828

15.3328 845

15.3419 884

15.3510 920

15.3601 887

15.3691 826

15.3782 836

15.3873 889

15.3964 896

15.4055 855

15.4146 862

15.4237 901

15.4327 875

15.4418 859

15.4509 900

15.4600 883

15.4691 830

15.4782 875

15.4872 853

15.4963 874

15.5054 848

15.5145 834

15.5236 935

15.5327 881

15.5418 845

15.5508 829

15.5599 910

15.5690 896

15.5781 824

15.5872 885

15.5963 876

15.6054 808

15.6144 890

15.6235 778

15.6326 853

15.6417 874

15.6508 897

15.6599 859

15.6689 825

15.6780 823

15.6871 902

15.6962 877

15.7053 943

15.7144 861

15.7235 843

15.7325 859

15.7416 910

15.7507 894

15.7598 851

15.7689 889

15.7780 863

15.7870 880

15.7961 876

15.8052 863

15.8143 892

15.8234 855

15.8325 861

15.8416 854

15.8506 918

15.8597 865

15.8688 851

15.8779 855

15.8870 848

15.8961 905

15.9052 878

15.9142 860

15.9233 850

15.9324 920

15.9415 845

15.9506 820

15.9597 804

15.9687 869

15.9778 892

15.9869 835

15.9960 892

16.0051 903

16.0142 846

16.0233 900

16.0323 880

16.0414 889

16.0505 888

16.0596 828

16.0687 817

16.0778 865

16.0868 919

16.0959 894

16.1050 902

16.1141 846

16.1232 813

16.1323 842

16.1414 884

16.1504 859

16.1595 822

16.1686 907

16.1777 850

16.1868 859

16.1959 854

16.2050 873

16.2140 861

16.2231 890

16.2322 897

16.2413 868

16.2504 823

16.2595 858

16.2685 862

16.2776 865

16.2867 799

16.2958 913

16.3049 904

16.3140 830

16.3231 763

16.3321 849

16.3412 876

16.3503 837

16.3594 883

16.3685 840

16.3776 881

16.3866 819

16.3957 853

16.4048 915

16.4139 867

16.4230 830

16.4321 804

16.4412 837

16.4502 864

16.4593 882

16.4684 867

16.4775 914

16.4866 818

16.4957 830

16.5048 868

16.5138 877

16.5229 862

16.5320 871

16.5411 868

16.5502 874

16.5593 793

16.5683 884

16.5774 838

16.5865 866

16.5956 867

16.6047 877

16.6138 812

16.6229 865

16.6319 895

16.6410 810

16.6501 862

16.6592 798

16.6683 883

16.6774 830

16.6864 903

16.6955 820

16.7046 821

16.7137 852

16.7228 852

16.7319 860

16.7410 849

16.7500 880

16.7591 826

16.7682 816

16.7773 878

16.7864 825

16.7955 873

16.8046 881

16.8136 797

16.8227 911

16.8318 852

16.8409 810

16.8500 895

16.8591 867

16.8681 817

16.8772 815

16.8863 839

16.8954 837

16.9045 881

16.9136 902

16.9227 831

16.9317 811

16.9408 828

16.9499 871

16.9590 880

16.9681 836

16.9772 881

16.9862 867

16.9953 820

17.0044 854

17.0135 892

17.0226 794

17.0317 847

17.0408 867

17.0498 879

17.0589 843

17.0680 860

17.0771 890

17.0862 852

17.0953 929

17.1044 845

17.1134 912

17.1225 818

17.1316 871

17.1407 923

17.1498 911

17.1589 863

17.1679 862

17.1770 869

17.1861 822

17.1952 857

17.2043 823

17.2134 864

17.2225 867

17.2315 861

17.2406 902

17.2497 880

17.2588 879

17.2679 832

17.2770 812

17.2860 824

17.2951 812

17.3042 844

17.3133 894

17.3224 836

17.3315 892

17.3406 888

17.3496 854

17.3587 816

17.3678 870

17.3769 783

17.3860 834

17.3951 912

17.4042 872

17.4132 892

17.4223 820

17.4314 874

17.4405 838

17.4496 834

17.4587 838

17.4677 854

17.4768 811

17.4859 859

17.4950 865

17.5041 864

17.5132 862

17.5223 839

17.5313 891

17.5404 844

17.5495 816

17.5586 881

17.5677 811

17.5768 874

17.5858 856

17.5949 875

17.6040 861

17.6131 847

17.6222 849

17.6313 866

17.6404 839

17.6494 814

17.6585 882

17.6676 848

17.6767 888

17.6858 878

17.6949 866

17.7040 850

17.7130 841

17.7221 846

17.7312 839

17.7403 827

17.7494 840

17.7585 883

17.7675 859

17.7766 876

17.7857 893

17.7948 849

17.8039 854

17.8130 847

17.8221 835

17.8311 825

17.8402 813

17.8493 850

17.8584 807

17.8675 871

17.8766 844

17.8856 880

17.8947 808

17.9038 860

17.9129 832

17.9220 869

17.9311 841

17.9402 871

17.9492 825

17.9583 848

17.9674 833

17.9765 877

17.9856 834

17.9947 859

18.0038 858

18.0128 825

18.0219 860

18.0310 870

18.0401 840

18.0492 797

18.0583 850

18.0673 829

18.0764 867

18.0855 843

18.0946 859

18.1037 843

18.1128 840

18.1219 847

18.1309 841

18.1400 832

18.1491 887

18.1582 854

18.1673 829

18.1764 839

18.1854 806

18.1945 856

18.2036 893

18.2127 810

18.2218 859

18.2309 827

18.2400 870

18.2490 804

18.2581 824

18.2672 841

18.2763 802

18.2854 852

18.2945 876

18.3036 817

18.3126 851

18.3217 862

18.3308 866

18.3399 832

18.3490 859

18.3581 857

18.3671 803

18.3762 872

18.3853 869

18.3944 909

18.4035 852

18.4126 840

18.4217 805

18.4307 838

18.4398 819

18.4489 847

18.4580 803

18.4671 827

18.4762 787

18.4852 882

18.4943 872

18.5034 893

18.5125 819

18.5216 855

18.5307 810

18.5398 866

18.5488 836

18.5579 854

18.5670 855

18.5761 828

18.5852 853

18.5943 776

18.6034 787

18.6124 890

18.6215 890

18.6306 794

18.6397 840

18.6488 839

18.6579 835

18.6669 799

18.6760 789

18.6851 832

18.6942 863

18.7033 845

18.7124 858

18.7215 877

18.7305 831

18.7396 854

18.7487 865

18.7578 851

18.7669 840

18.7760 803

18.7850 854

18.7941 875

18.8032 869

18.8123 858

18.8214 834

18.8305 819

18.8396 839

18.8486 794

18.8577 856

18.8668 844

18.8759 845

18.8850 866

18.8941 806

18.9032 821

18.9122 797

18.9213 783

18.9304 852

18.9395 844

18.9486 784

18.9577 842

18.9667 827

18.9758 835

18.9849 798

18.9940 849

19.0031 844

19.0122 821

19.0213 797

19.0303 849

19.0394 838

19.0485 848

19.0576 814

19.0667 815

19.0758 874

19.0848 821

19.0939 835

19.1030 848

19.1121 850

19.1212 845

19.1303 817

19.1394 843

19.1484 833

19.1575 879

19.1666 805

19.1757 824

19.1848 786

19.1939 801

19.2030 809

19.2120 799

19.2211 807

19.2302 817

19.2393 861

19.2484 846

19.2575 854

19.2665 822

19.2756 790

19.2847 922

19.2938 841

19.3029 850

19.3120 835

19.3211 810

19.3301 803

19.3392 841

19.3483 847

19.3574 831

19.3665 893

19.3756 820

19.3847 808

19.3937 778

19.4028 832

19.4119 780

19.4210 863

19.4301 848

19.4392 860

19.4482 754

19.4573 832

19.4664 822

19.4755 817

19.4846 844

19.4937 771

19.5028 861

19.5118 795

19.5209 854

19.5300 844

19.5391 811

19.5482 825

19.5573 853

19.5663 816

19.5754 817

19.5845 757

19.5936 823

19.6027 837

19.6118 871

19.6209 799

19.6299 795

19.6390 838

19.6481 792

19.6572 826

19.6663 795

19.6754 845

19.6845 856

19.6935 790

19.7026 823

19.7117 859

19.7208 812

19.7299 820

19.7390 842

19.7480 826

19.7571 853

19.7662 868

19.7753 852

19.7844 853

19.7935 851

19.8026 822

19.8116 814

19.8207 821

19.8298 806

19.8389 860

19.8480 834

19.8571 841

19.8661 841

19.8752 810

19.8843 852

19.8934 813

19.9025 825

19.9116 737

19.9207 814

19.9297 785

19.9388 808

19.9479 811

19.9570 813

19.9661 787

19.9752 765

19.9843 842

19.9933 785

20.0024 850

20.0115 855

20.0206 846

20.0297 797

20.0388 769

20.0478 816

20.0569 842

20.0660 782

20.0751 755

20.0842 872

20.0933 822

20.1024 755

20.1114 767

20.1205 791

20.1296 784

20.1387 828

20.1478 832

20.1569 820

20.1659 818

20.1750 806

20.1841 787

20.1932 803

20.2023 778

20.2114 818

20.2205 799

20.2295 824

20.2386 816

20.2477 824

20.2568 787

20.2659 792

20.2750 839

20.2841 820

20.2931 810

20.3022 786

20.3113 790

20.3204 811

20.3295 787

20.3386 831

20.3476 847

20.3567 783

20.3658 805

20.3749 783

20.3840 823

20.3931 826

20.4022 773

20.4112 822

20.4203 822

20.4294 772

20.4385 803

20.4476 808

20.4567 848

20.4657 843

20.4748 782

20.4839 753

20.4930 801

20.5021 796

20.5112 786

20.5203 808

20.5293 796

20.5384 784

20.5475 810

20.5566 786

20.5657 795

20.5748 825

20.5839 782

20.5929 776

20.6020 789

20.6111 830

20.6202 783

20.6293 777

20.6384 773

20.6474 813

20.6565 822

20.6656 799

20.6747 783

20.6838 783

20.6929 820

20.7020 855

20.7110 756

20.7201 759

20.7292 817

20.7383 797

20.7474 802

20.7565 791

20.7655 803

20.7746 811

20.7837 761

20.7928 833

20.8019 807

20.8110 747

20.8201 799

20.8291 803

20.8382 796

20.8473 783

20.8564 783

20.8655 777

20.8746 794

20.8837 732

20.8927 776

20.9018 793

20.9109 757

20.9200 725

20.9291 821

20.9382 755

20.9472 747

20.9563 770

20.9654 783

20.9745 765

20.9836 823

20.9927 770

21.0018 765

21.0108 812

21.0199 809

21.0290 816

21.0381 817

21.0472 799

21.0563 773

21.0653 769

21.0744 797

21.0835 794

21.0926 777

21.1017 825

21.1108 776

21.1199 778

21.1289 742

21.1380 766

21.1471 740

21.1562 733

21.1653 824

21.1744 813

21.1835 774

21.1925 783

21.2016 779

21.2107 824

21.2198 787

21.2289 749

21.2380 793

21.2470 786

21.2561 730

21.2652 746

21.2743 813

21.2834 801

21.2925 755

21.3016 738

21.3106 777

21.3197 747

21.3288 771

21.3379 768

21.3470 747

21.3561 737

21.3651 778

21.3742 786

21.3833 770

21.3924 805

21.4015 785

21.4106 771

21.4197 708

21.4287 769

21.4378 771

21.4469 760

21.4560 792

21.4651 715

21.4742 739

21.4833 727

21.4923 740

21.5014 736

21.5105 783

21.5196 778

21.5287 744

21.5378 820

21.5468 731

21.5559 773

21.5650 757

21.5741 760

21.5832 790

21.5923 771

21.6014 720

21.6104 743

21.6195 786

21.6286 827

21.6377 755

21.6468 744

21.6559 797

21.6649 774

21.6740 788

21.6831 815

21.6922 748

21.7013 780

21.7104 782

21.7195 758

21.7285 741

21.7376 756

21.7467 776

21.7558 793

21.7649 765

21.7740 778

21.7831 800

21.7921 727

21.8012 794

21.8103 786

21.8194 740

21.8285 750

21.8376 767

21.8466 768

21.8557 785

21.8648 758

21.8739 768

21.8830 776

21.8921 751

21.9012 708

21.9102 741

21.9193 717

21.9284 727

21.9375 717

21.9466 738

21.9557 713

21.9647 789

21.9738 760

21.9829 751

21.9920 761

22.0011 754

22.0102 754

22.0193 723

22.0283 723

22.0374 764

22.0465 731

22.0556 774

22.0647 778

22.0738 743

22.0829 734

22.0919 771

22.1010 730

22.1101 813

22.1192 788

22.1283 720

22.1374 754

22.1464 758

22.1555 778

22.1646 746

22.1737 749

22.1828 735

22.1919 775

22.2010 800

22.2100 754

22.2191 709

22.2282 699

22.2373 739

22.2464 744

22.2555 717

22.2645 746

22.2736 746

22.2827 721

22.2918 728

22.3009 751

22.3100 717

22.3191 713

22.3281 788

22.3372 740

22.3463 695

22.3554 735

22.3645 730

22.3736 720

22.3827 774

22.3917 736

22.4008 747

22.4099 756

22.4190 718

22.4281 748

22.4372 713

22.4462 703

22.4553 693

22.4644 691

22.4735 677

22.4826 730

22.4917 729

22.5008 740

22.5098 722

22.5189 773

22.5280 734

22.5371 777

22.5462 738

22.5553 761

22.5643 781

22.5734 731

22.5825 747

22.5916 716

22.6007 750

22.6098 712

22.6189 734

22.6279 734

22.6370 716

22.6461 759

22.6552 687

22.6643 745

22.6734 706

22.6825 718

22.6915 732

22.7006 700

22.7097 749

22.7188 740

22.7279 679

22.7370 701

22.7460 695

22.7551 733

22.7642 714

22.7733 699

22.7824 714

22.7915 678

22.8006 737

22.8096 713

22.8187 691

22.8278 743

22.8369 724

22.8460 730

22.8551 715

22.8641 677

22.8732 718

22.8823 741

22.8914 749

22.9005 686

22.9096 733

22.9187 698

22.9277 719

22.9368 710

22.9459 758

22.9550 697

22.9641 689

22.9732 668

22.9823 734

22.9913 697

23.0004 772

23.0095 744

23.0186 713

23.0277 692

23.0368 687

23.0458 762

23.0549 709

23.0640 706

23.0731 722

23.0822 686

23.0913 707

23.1004 690

23.1094 680

23.1185 690

23.1276 677

23.1367 691

23.1458 710

23.1549 682

23.1639 746

23.1730 751

23.1821 674

23.1912 716

23.2003 717

23.2094 716

23.2185 715

23.2275 710

23.2366 719

23.2457 712

23.2548 677

23.2639 728

23.2730 766

23.2821 713

23.2911 699

23.3002 690

23.3093 771

23.3184 706

23.3275 675

23.3366 757

23.3456 697

23.3547 700

23.3638 738

23.3729 704

23.3820 718

23.3911 703

23.4002 690

23.4092 695

23.4183 741

23.4274 702

23.4365 729

23.4456 759

23.4547 705

23.4637 688

23.4728 715

23.4819 694

23.4910 644

23.5001 695

23.5092 676

23.5183 678

23.5273 691

23.5364 668

23.5455 709

23.5546 672

23.5637 646

23.5728 692

23.5819 694

23.5909 712

23.6000 677

23.6091 708

23.6182 655

23.6273 697

23.6364 727

23.6454 741

23.6545 691

23.6636 697

23.6727 748

23.6818 666

23.6909 742

23.7000 682

23.7090 723

23.7181 723

23.7272 716

23.7363 676

23.7454 689

23.7545 680

23.7635 732

23.7726 747

23.7817 750

23.7908 669

23.7999 663

23.8090 683

23.8181 714

23.8271 704

23.8362 707

23.8453 707

23.8544 708

23.8635 667

23.8726 726

23.8817 679

23.8907 702

23.8998 694

23.9089 685

23.9180 718

23.9271 669

23.9362 677

23.9452 665

23.9543 720

23.9634 723

23.9725 721

23.9816 696

23.9907 729

23.9998 712

24.0088 707

24.0179 671

24.0270 706

24.0361 681

24.0452 649

24.0543 698

24.0633 677

24.0724 673

24.0815 667

24.0906 718

24.0997 683

24.1088 711

24.1179 699

24.1269 686

24.1360 682

24.1451 673

24.1542 679

24.1633 678

24.1724 711

24.1815 650

24.1905 651

24.1996 662

24.2087 709

24.2178 688

24.2269 701

24.2360 693

24.2450 693

24.2541 641

24.2632 702

24.2723 621

24.2814 656

24.2905 702

24.2996 706

24.3086 659

24.3177 696

24.3268 677

24.3359 684

24.3450 669

24.3541 725

24.3631 710

24.3722 667

24.3813 680

24.3904 697

24.3995 694

24.4086 656

24.4177 697

24.4267 648

24.4358 674

24.4449 686

24.4540 651

24.4631 690

24.4722 607

24.4813 694

24.4903 650

24.4994 674

24.5085 650

24.5176 692

24.5267 682

24.5358 703

24.5448 679

24.5539 669

24.5630 731

24.5721 668

24.5812 721

24.5903 691

24.5994 709

24.6084 705

24.6175 660

24.6266 662

24.6357 652

24.6448 697

24.6539 680

24.6629 704

24.6720 669

24.6811 649

24.6902 680

24.6993 687

24.7084 659

24.7175 651

24.7265 704

24.7356 658

24.7447 646

24.7538 689

24.7629 702

24.7720 676

24.7811 650

24.7901 643

24.7992 686

24.8083 671

24.8174 659

24.8265 634

24.8356 631

24.8446 650

24.8537 678

24.8628 647

24.8719 632

24.8810 693

24.8901 672

24.8992 615

24.9082 682

24.9173 722

24.9264 653

24.9355 681

24.9446 679

24.9537 694

24.9627 650

24.9718 740

24.9809 647

24.9900 710

24.9991 631

25.0082 704

25.0173 651

25.0263 684

25.0354 690

25.0445 676

25.0536 651

25.0627 663

25.0718 648

25.0809 684

25.0899 647

25.0990 641

25.1081 685

25.1172 704

25.1263 629

25.1354 630

25.1444 692

25.1535 684

25.1626 632

25.1717 648

25.1808 689

25.1899 668

25.1990 632

25.2080 680

25.2171 676

25.2262 679

25.2353 675

25.2444 680

25.2535 651

25.2625 667

25.2716 675

25.2807 664

25.2898 674

25.2989 672

25.3080 673

25.3171 691

25.3261 710

25.3352 707

25.3443 685

25.3534 662

25.3625 659

25.3716 664

25.3807 700

25.3897 661

25.3988 613

25.4079 677

25.4170 670

25.4261 653

25.4352 683

25.4442 678

25.4533 678

25.4624 660

25.4715 697

25.4806 665

25.4897 676

25.4988 640

25.5078 636

25.5169 637

25.5260 658

25.5351 685

25.5442 723

25.5533 702

25.5623 687

25.5714 646

25.5805 629

25.5896 667

25.5987 646

25.6078 653

25.6169 632

25.6259 588

25.6350 649

25.6441 644

25.6532 683

25.6623 673

25.6714 634

25.6805 671

25.6895 676

25.6986 645

25.7077 649

25.7168 648

25.7259 640

25.7350 666

25.7440 670

25.7531 629

25.7622 633

25.7713 679

25.7804 650

25.7895 639

25.7986 645

25.8076 620

25.8167 676

25.8258 666

25.8349 655

25.8440 665

25.8531 702

25.8621 647

25.8712 662

25.8803 608

25.8894 641

25.8985 682

25.9076 658

25.9167 610

25.9257 636

25.9348 649

25.9439 645

25.9530 635

25.9621 628

25.9712 664

25.9803 660

25.9893 628

25.9984 665

26.0075 693

26.0166 660

26.0257 662

26.0348 637

26.0438 576

26.0529 644

26.0620 689

26.0711 673

26.0802 625

26.0893 674

26.0984 660

26.1074 681

26.1165 645

26.1256 641

26.1347 621

26.1438 656

26.1529 619

26.1619 672

26.1710 649

26.1801 612

26.1892 632

26.1983 606

26.2074 647

26.2165 637

26.2255 624

26.2346 686

26.2437 640

26.2528 608

26.2619 660

26.2710 658

26.2801 653

26.2891 624

26.2982 620

26.3073 671

26.3164 671

26.3255 661

26.3346 654

26.3436 626

26.3527 586

26.3618 615

26.3709 624

26.3800 611

26.3891 653

26.3982 655

26.4072 688

26.4163 645

26.4254 595

26.4345 622

26.4436 610

26.4527 642

26.4617 715

26.4708 656

26.4799 644

26.4890 631

26.4981 628

26.5072 676

26.5163 632

26.5253 597

26.5344 624

26.5435 642

26.5526 628

26.5617 651

26.5708 605

26.5799 661

26.5889 600

26.5980 633

26.6071 684

26.6162 648

26.6253 668

26.6344 620

26.6434 619

26.6525 603

26.6616 664

26.6707 630

26.6798 667

26.6889 613

26.6980 581

26.7070 632

26.7161 651

26.7252 649

26.7343 631

26.7434 646

26.7525 644

26.7615 593

26.7706 605

26.7797 610

26.7888 622

26.7979 638

26.8070 596

26.8161 642

26.8251 576

26.8342 614

26.8433 599

26.8524 609

26.8615 584

26.8706 621

26.8797 600

26.8887 592

26.8978 604

26.9069 600

26.9160 592

26.9251 602

26.9342 646

26.9432 595

26.9523 650

26.9614 586

26.9705 622

26.9796 613

26.9887 614

26.9978 601

27.0068 630

27.0159 595

27.0250 600

27.0341 620

27.0432 610

27.0523 624

27.0613 616

27.0704 650

27.0795 617

27.0886 609

27.0977 607

27.1068 660

27.1159 638

27.1249 625

27.1340 615

27.1431 585

27.1522 613

27.1613 601

27.1704 643

27.1795 640

27.1885 620

27.1976 624

27.2067 633

27.2158 605

27.2249 650

27.2340 602

27.2430 637

27.2521 614

27.2612 666

27.2703 626

27.2794 585

27.2885 609

27.2976 632

27.3066 617

27.3157 635

27.3248 582

27.3339 626

27.3430 624

27.3521 664

27.3611 626

27.3702 608

27.3793 616

27.3884 639

27.3975 595

27.4066 618

27.4157 615

27.4247 627

27.4338 613

27.4429 687

27.4520 606

27.4611 650

27.4702 569

27.4793 582

27.4883 652

27.4974 642

27.5065 657

27.5156 660

27.5247 585

27.5338 639

27.5428 619

27.5519 619

27.5610 620

27.5701 640

27.5792 573

27.5883 683

27.5974 614

27.6064 617

27.6155 616

27.6246 605

27.6337 647

27.6428 599

27.6519 616

27.6609 649

27.6700 639

27.6791 649

27.6882 642

27.6973 629

27.7064 565

27.7155 574

27.7245 580

27.7336 609

27.7427 567

27.7518 626

27.7609 640

27.7700 613

27.7791 594

27.7881 614

27.7972 638

27.8063 660

27.8154 605

27.8245 631

27.8336 560

27.8426 600

27.8517 539

27.8608 545

27.8699 622

27.8790 589

27.8881 628

27.8972 613

27.9062 617

27.9153 641

27.9244 613

27.9335 610

27.9426 589

27.9517 563

27.9607 596

27.9698 584

27.9789 617

27.9880 565

27.9971 601

28.0062 581

28.0153 612

28.0243 612

28.0334 608

28.0425 616

28.0516 618

28.0607 601

28.0698 605

28.0789 617

28.0879 561

28.0970 607

28.1061 602

28.1152 557

28.1243 596

28.1334 583

28.1424 649

28.1515 584

28.1606 683

28.1697 569

28.1788 570

28.1879 588

28.1970 561

28.2060 667

28.2151 562

28.2242 602

28.2333 607

28.2424 607

28.2515 605

28.2605 557

28.2696 609

28.2787 610

28.2878 626

28.2969 607

28.3060 598

28.3151 602

28.3241 564

28.3332 616

28.3423 631

28.3514 601

28.3605 590

28.3696 549

28.3787 552

28.3877 582

28.3968 589

28.4059 651

28.4150 592

28.4241 590

28.4332 586

28.4422 623

28.4513 572

28.4604 620

28.4695 584

28.4786 592

28.4877 610

28.4968 588

28.5058 554

28.5149 618

28.5240 597

28.5331 582

28.5422 564

28.5513 600

28.5603 606

28.5694 572

28.5785 546

28.5876 567

28.5967 601

28.6058 560

28.6149 606

28.6239 566

28.6330 630

28.6421 623

28.6512 574

28.6603 584

28.6694 610

28.6785 579

28.6875 616

28.6966 600

28.7057 618

28.7148 591

28.7239 634

28.7330 514

28.7420 600

28.7511 607

28.7602 564

28.7693 562

28.7784 615

28.7875 569

28.7966 545

28.8056 599

28.8147 610

28.8238 590

28.8329 587

28.8420 623

28.8511 535

28.8601 571

28.8692 563

28.8783 576

28.8874 584

28.8965 604

28.9056 597

28.9147 608

28.9237 578

28.9328 570

28.9419 600

28.9510 655

28.9601 577

28.9692 610

28.9783 587

28.9873 592

28.9964 587

29.0055 563

29.0146 588

29.0237 605

29.0328 606

29.0418 578

29.0509 571

29.0600 595

29.0691 586

29.0782 616

29.0873 582

29.0964 573

29.1054 580

29.1145 563

29.1236 576

29.1327 620

29.1418 595

29.1509 598

29.1599 605

29.1690 572

29.1781 589

29.1872 574

29.1963 604

29.2054 589

29.2145 558

29.2235 577

29.2326 635

29.2417 567

29.2508 555

29.2599 583

29.2690 572

29.2781 562

29.2871 541

29.2962 594

29.3053 545

29.3144 533

29.3235 635

29.3326 557

29.3416 607

29.3507 597

29.3598 565

29.3689 576

29.3780 615

29.3871 555

29.3962 596

29.4052 569

29.4143 568

29.4234 536

29.4325 558

29.4416 550

29.4507 625

29.4597 618

29.4688 508

29.4779 484

29.4870 541

29.4961 605

29.5052 606

29.5143 613

29.5233 562

29.5324 544

29.5415 631

29.5506 624

29.5597 560

29.5688 552

29.5779 559

29.5869 568

29.5960 526

29.6051 600

29.6142 568

29.6233 586

29.6324 590

29.6414 566

29.6505 587

29.6596 575

29.6687 536

29.6778 586

29.6869 552

29.6960 641

29.7050 566

29.7141 554

29.7232 607

29.7323 574

29.7414 576

29.7505 577

29.7595 577

29.7686 571

29.7777 578

29.7868 577

29.7959 592

29.8050 601

29.8141 554

29.8231 549

29.8322 572

29.8413 572

29.8504 617

29.8595 564

29.8686 558

29.8777 574

29.8867 528

29.8958 547

29.9049 560

29.9140 568

29.9231 568

29.9322 554

29.9412 564

29.9503 583

29.9594 589

29.9685 566

29.9776 563

29.9867 574

29.9958 574

30.0048 560

30.0139 577

30.0230 555

30.0321 568

30.0412 586

30.0503 545

30.0593 556

30.0684 573

30.0775 608

30.0866 579

30.0957 550

30.1048 560

30.1139 569

30.1229 580

30.1320 539

30.1411 564

30.1502 521

30.1593 629

30.1684 593

30.1775 543

30.1865 556

30.1956 550

30.2047 574

30.2138 575

30.2229 593

30.2320 535

30.2410 521

30.2501 547

30.2592 572

30.2683 545

30.2774 589

30.2865 570

30.2956 582

30.3046 587

30.3137 550

30.3228 558

30.3319 561

30.3410 567

30.3501 550

30.3591 578

30.3682 540

30.3773 553

30.3864 540

30.3955 556

30.4046 542

30.4137 586

30.4227 552

30.4318 569

30.4409 555

30.4500 611

30.4591 539

30.4682 566

30.4773 559

30.4863 562

30.4954 549

30.5045 582

30.5136 544

30.5227 551

30.5318 569

30.5408 559

30.5499 553

30.5590 574

30.5681 532

30.5772 582

30.5863 549

30.5954 545

30.6044 512

30.6135 561

30.6226 599

30.6317 567

30.6408 567

30.6499 575

30.6589 589

30.6680 547

30.6771 562

30.6862 571

30.6953 527

30.7044 594

30.7135 576

30.7225 528

30.7316 539

30.7407 532

30.7498 551

30.7589 563

30.7680 564

30.7771 526

30.7861 598

30.7952 546

30.8043 544

30.8134 512

30.8225 573

30.8316 588

30.8406 555

30.8497 560

30.8588 534

30.8679 552

30.8770 542

30.8861 508

30.8952 582

30.9042 549

30.9133 543

30.9224 552

30.9315 571

30.9406 524

30.9497 567

30.9587 532

30.9678 544

30.9769 594

30.9860 538

30.9951 585

31.0042 543

31.0133 553

31.0223 567

31.0314 567

31.0405 548

31.0496 541

31.0587 576

31.0678 574

31.0769 539

31.0859 622

31.0950 555

31.1041 586

31.1132 553

31.1223 539

31.1314 564

31.1404 556

31.1495 566

31.1586 583

31.1677 578

31.1768 598

31.1859 498

31.1950 503

31.2040 569

31.2131 540

31.2222 588

31.2313 562

31.2404 558

31.2495 576

31.2585 617

31.2676 526

31.2767 526

31.2858 541

31.2949 535

31.3040 554

31.3131 526

31.3221 579

31.3312 595

31.3403 574

31.3494 551

31.3585 530

31.3676 546

31.3767 557

31.3857 527

31.3948 543

31.4039 534

31.4130 523

31.4221 490

31.4312 525

31.4402 532

31.4493 578

31.4584 577

31.4675 535

31.4766 558

31.4857 565

31.4948 554

31.5038 570

31.5129 568

31.5220 562

31.5311 582

31.5402 557

31.5493 573

31.5583 508

31.5674 554

31.5765 517

31.5856 507

31.5947 581

31.6038 527

31.6129 555

31.6219 555

31.6310 524

31.6401 559

31.6492 539

31.6583 572

31.6674 532

31.6765 553

31.6855 553

31.6946 592

31.7037 528

31.7128 556

31.7219 571

31.7310 543

31.7400 555

31.7491 540

31.7582 522

31.7673 500

31.7764 550

31.7855 532

31.7946 508

31.8036 578

31.8127 573

31.8218 553

31.8309 546

31.8400 547

31.8491 513

31.8581 546

31.8672 524

31.8763 542

31.8854 563

31.8945 521

31.9036 557

31.9127 575

31.9217 567

31.9308 568

31.9399 564

31.9490 574

31.9581 551

31.9672 519

31.9763 539

31.9853 557

31.9944 507

32.0035 539

32.0126 539

32.0217 551

32.0308 532

32.0398 557

32.0489 505

32.0580 568

32.0671 552

32.0762 543

32.0853 537

32.0944 576

32.1034 552

32.1125 555

32.1216 507

32.1307 548

32.1398 560

32.1489 532

32.1579 513

32.1670 519

32.1761 484

32.1852 537

32.1943 497

32.2034 541

32.2125 566

32.2215 539

32.2306 511

32.2397 578

32.2488 562

32.2579 512

32.2670 539

32.2761 528

32.2851 552

32.2942 513

32.3033 537

32.3124 552

32.3215 527

32.3306 527

32.3396 576

32.3487 544

32.3578 567

32.3669 540

32.3760 534

32.3851 506

32.3942 542

32.4032 534

32.4123 528

32.4214 559

32.4305 533

32.4396 541

32.4487 546

32.4577 518

32.4668 544

32.4759 518

32.4850 518

32.4941 538

32.5032 560

32.5123 528

32.5213 545

32.5304 527

32.5395 542

32.5486 519

32.5577 528

32.5668 533

32.5759 566

32.5849 538

32.5940 560

32.6031 544

32.6122 563

32.6213 519

32.6304 536

32.6394 501

32.6485 539

32.6576 559

32.6667 534

32.6758 529

32.6849 539

32.6940 489

32.7030 511

32.7121 529

32.7212 557

32.7303 540

32.7394 541

32.7485 559

32.7575 533

32.7666 500

32.7757 552

32.7848 564

32.7939 534

32.8030 534

32.8121 574

32.8211 530

32.8302 530

32.8393 534

32.8484 523

32.8575 531

32.8666 598

32.8757 559

32.8847 520

32.8938 499

32.9029 563

32.9120 564

32.9211 538

32.9302 518

32.9392 585

32.9483 545

32.9574 553

32.9665 545

32.9756 544

32.9847 527

32.9938 485

33.0028 541

33.0119 608

33.0210 548

33.0301 557

33.0392 562

33.0483 508

33.0573 510

33.0664 564

33.0755 595

33.0846 546

33.0937 517

33.1028 509

33.1119 515

33.1209 520

33.1300 531

33.1391 574

33.1482 546

33.1573 559

33.1664 557

33.1755 525

33.1845 537

33.1936 525

33.2027 517

33.2118 502

33.2209 524

33.2300 537

33.2390 557

33.2481 515

33.2572 565

33.2663 536

33.2754 548

33.2845 564

33.2936 582

33.3026 518

33.3117 541

33.3208 567

33.3299 511

33.3390 572

33.3481 531

33.3571 526

33.3662 541

33.3753 569

33.3844 533

33.3935 581

33.4026 538

33.4117 509

33.4207 566

33.4298 570

33.4389 522

33.4480 549

33.4571 503

33.4662 518

33.4753 522

33.4843 572

33.4934 517

33.5025 559

33.5116 551

33.5207 523

33.5298 521

33.5388 495

33.5479 524

33.5570 564

33.5661 533

33.5752 590

33.5843 529

33.5934 529

33.6024 513

33.6115 566

33.6206 530

33.6297 510

33.6388 561

33.6479 503

33.6569 538

33.6660 504

33.6751 562

33.6842 542

33.6933 536

33.7024 541

33.7115 543

33.7205 548

33.7296 560

33.7387 535

33.7478 514

33.7569 554

33.7660 523

33.7751 541

33.7841 556

33.7932 517

33.8023 571

33.8114 490

33.8205 512

33.8296 560

33.8386 543

33.8477 551

33.8568 560

33.8659 521

33.8750 509

33.8841 553

33.8932 539

33.9022 570

33.9113 512

33.9204 529

33.9295 562

33.9386 548

33.9477 539

33.9567 550

33.9658 521

33.9749 509

33.9840 568

33.9931 508

34.0022 522

34.0113 511

34.0203 568

34.0294 544

34.0385 549

34.0476 563

34.0567 520

34.0658 486

34.0749 537

34.0839 517

34.0930 529

34.1021 507

34.1112 505

34.1203 515

34.1294 511

34.1384 555

34.1475 562

34.1566 530

34.1657 556

34.1748 550

34.1839 531

34.1930 523

34.2020 549

34.2111 508

34.2202 521

34.2293 553

34.2384 524

34.2475 512

34.2565 538

34.2656 538

34.2747 492

34.2838 531

34.2929 542

34.3020 533

34.3111 524

34.3201 494

34.3292 544

34.3383 544

34.3474 582

34.3565 527

34.3656 502

34.3747 548

34.3837 480

34.3928 500

34.4019 537

34.4110 541

34.4201 513

34.4292 518

34.4382 503

34.4473 530

34.4564 535

34.4655 536

34.4746 498

34.4837 532

34.4928 529

34.5018 516

34.5109 529

34.5200 528

34.5291 519

34.5382 560

34.5473 562

34.5563 539

34.5654 509

34.5745 519

34.5836 508

34.5927 487

34.6018 582

34.6109 567

34.6199 536

34.6290 517

34.6381 482

34.6472 519

34.6563 483

34.6654 508

34.6745 539

34.6835 528

34.6926 524

34.7017 560

34.7108 547

34.7199 548

34.7290 558

34.7380 537

34.7471 527

34.7562 485

34.7653 537

34.7744 520

34.7835 565

34.7926 548

34.8016 555

34.8107 523

34.8198 516

34.8289 541

34.8380 510

34.8471 509

34.8561 516

34.8652 513

34.8743 547

34.8834 554

34.8925 508

34.9016 508

34.9107 519

34.9197 500

34.9288 500

34.9379 502

34.9470 502

34.9561 508

34.9652 550

34.9743 512

34.9833 477

34.9924 511

35.0015 537

35.0106 542

35.0197 540

35.0288 538

35.0378 473

35.0469 515

35.0560 498

35.0651 531

35.0742 543

35.0833 526

35.0924 506

35.1014 504

35.1105 531

35.1196 526

35.1287 514

35.1378 558

35.1469 536

35.1559 494

35.1650 510

35.1741 503

35.1832 513

35.1923 540

35.2014 504

35.2105 557

35.2195 532

35.2286 535

35.2377 523

35.2468 457

35.2559 548

35.2650 539

35.2741 542

35.2831 558

35.2922 482

35.3013 504

35.3104 488

35.3195 504

35.3286 539

35.3376 506

35.3467 529

35.3558 472

35.3649 514

35.3740 489

35.3831 508

35.3922 528

35.4012 467

35.4103 488

35.4194 544

35.4285 470

35.4376 548

35.4467 519

35.4557 531

35.4648 534

35.4739 542

35.4830 489

35.4921 531

35.5012 512

35.5103 499

35.5193 545

35.5284 482

35.5375 540

35.5466 522

35.5557 554

35.5648 516

35.5739 520

35.5829 489

35.5920 515

35.6011 549

35.6102 500

35.6193 530

35.6284 495

35.6374 499

35.6465 527

35.6556 539

35.6647 513

35.6738 505

35.6829 539

35.6920 488

35.7010 542

35.7101 538

35.7192 516

35.7283 483

35.7374 514

35.7465 507

35.7555 490

35.7646 509

35.7737 547

35.7828 514

35.7919 510

35.8010 492

35.8101 519

35.8191 546

35.8282 495

35.8373 492

35.8464 561

35.8555 528

35.8646 517

35.8737 533

35.8827 508

35.8918 529

35.9009 533

35.9100 499

35.9191 489

35.9282 527

35.9372 511

35.9463 522

35.9554 544

35.9645 488

35.9736 543

35.9827 533

35.9918 481

36.0008 558

36.0099 540

36.0190 510

36.0281 502

36.0372 493

36.0463 555

36.0553 557

36.0644 521

36.0735 528

36.0826 561

36.0917 551

36.1008 475

36.1099 513

36.1189 536

36.1280 546

36.1371 488

36.1462 527

36.1553 489

36.1644 514

36.1735 528

36.1825 475

36.1916 484

36.2007 506

36.2098 500

36.2189 521

36.2280 528

36.2370 525

36.2461 479

36.2552 538

36.2643 478

36.2734 538

36.2825 534

36.2916 504

36.3006 528

36.3097 498

36.3188 507

36.3279 521

36.3370 501

36.3461 503

36.3551 520

36.3642 509

36.3733 497

36.3824 510

36.3915 517

36.4006 519

36.4097 543

36.4187 510

36.4278 476

36.4369 502

36.4460 497

36.4551 526

36.4642 501

36.4733 509

36.4823 536

36.4914 511

36.5005 522

36.5096 517

36.5187 484

36.5278 574

36.5368 540

36.5459 563

36.5550 537

36.5641 512

36.5732 492

36.5823 510

36.5914 516

36.6004 503

36.6095 503

36.6186 570

36.6277 513

36.6368 520

36.6459 492

36.6549 509

36.6640 533

36.6731 538

36.6822 514

36.6913 554

36.7004 522

36.7095 540

36.7185 508

36.7276 523

36.7367 488

36.7458 534

36.7549 529

36.7640 532

36.7731 502

36.7821 522

36.7912 482

36.8003 527

36.8094 512

36.8185 492

36.8276 549

36.8366 469

36.8457 498

36.8548 477

36.8639 520

36.8730 536

36.8821 535

36.8912 476

36.9002 505

36.9093 513

36.9184 496

36.9275 525

36.9366 522

36.9457 502

36.9547 557

36.9638 519

36.9729 508

36.9820 510

36.9911 511

37.0002 514

37.0093 493

37.0183 535

37.0274 526

37.0365 529

37.0456 458

37.0547 525

37.0638 495

37.0729 493

37.0819 490

37.0910 524

37.1001 517

37.1092 478

37.1183 517

37.1274 493

37.1364 483

37.1455 484

37.1546 518

37.1637 519

37.1728 516

37.1819 479

37.1910 558

37.2000 482

37.2091 550

37.2182 518

37.2273 514

37.2364 520

37.2455 503

37.2546 503

37.2636 521

37.2727 519

37.2818 508

37.2909 555

37.3000 499

37.3091 491

37.3181 486

37.3272 506

37.3363 497

37.3454 533

37.3545 507

37.3636 510

37.3727 504

37.3817 494

37.3908 459

37.3999 530

37.4090 518

37.4181 471

37.4272 485

37.4362 482

37.4453 542

37.4544 498

37.4635 527

37.4726 501

37.4817 489

37.4908 497

37.4998 461

37.5089 530

37.5180 517

37.5271 500

37.5362 547

37.5453 503

37.5544 483

37.5634 508

37.5725 504

37.5816 526

37.5907 536

37.5998 530

37.6089 531

37.6179 505

37.6270 503

37.6361 536

37.6452 498

37.6543 507

37.6634 474

37.6725 459

37.6815 479

37.6906 468

37.6997 482

37.7088 480

37.7179 474

37.7270 502

37.7360 476

37.7451 524

37.7542 467

37.7633 503

37.7724 512

37.7815 517

37.7906 515

37.7996 512

37.8087 533

37.8178 537

37.8269 515

37.8360 510

37.8451 515

37.8542 477

37.8632 473

37.8723 545

37.8814 479

37.8905 513

37.8996 489

37.9087 545

37.9177 470

37.9268 534

37.9359 467

37.9450 482

37.9541 450

37.9632 520

37.9723 503

37.9813 509

37.9904 503

37.9995 492

38.0086 554

38.0177 506

38.0268 499

38.0358 500

38.0449 537

38.0540 517

38.0631 484

38.0722 453

38.0813 504

38.0904 469

38.0994 502

38.1085 510

38.1176 480

38.1267 471

38.1358 513

38.1449 512

38.1540 483

38.1630 494

38.1721 478

38.1812 503

38.1903 455

38.1994 487

38.2085 474

38.2175 522

38.2266 471

38.2357 477

38.2448 533

38.2539 522

38.2630 445

38.2721 446

38.2811 510

38.2902 499

38.2993 508

38.3084 488

38.3175 485

38.3266 510

38.3356 496

38.3447 523

38.3538 478

38.3629 470

38.3720 487

38.3811 508

38.3902 475

38.3992 476

38.4083 478

38.4174 509

38.4265 502

38.4356 431

38.4447 485

38.4538 499

38.4628 485

38.4719 524

38.4810 508

38.4901 493

38.4992 486

38.5083 508

38.5173 505

38.5264 502

38.5355 443

38.5446 517

38.5537 489

38.5628 527

38.5719 475

38.5809 468

38.5900 509

38.5991 527

38.6082 461

38.6173 513

38.6264 482

38.6354 494

38.6445 471

38.6536 486

38.6627 480

38.6718 503

38.6809 519

38.6900 447

38.6990 476

38.7081 475

38.7172 448

38.7263 418

38.7354 431

38.7445 468

38.7536 429

38.7626 504

38.7717 469

38.7808 490

38.7899 473

38.7990 530

38.8081 457

38.8171 504

38.8262 502

38.8353 449

38.8444 503

38.8535 458

38.8626 467

38.8717 487

38.8807 481

38.8898 496

38.8989 529

38.9080 486

38.9171 500

38.9262 482

38.9352 443

38.9443 530

38.9534 494

38.9625 489

38.9716 513

38.9807 478

38.9898 462

38.9988 439

39.0079 481

39.0170 463

39.0261 452

39.0352 508

39.0443 476

39.0534 494

39.0624 483

39.0715 505

39.0806 500

39.0897 461

39.0988 499

39.1079 455

39.1169 442

39.1260 473

39.1351 472

39.1442 466

39.1533 486

39.1624 442

39.1715 493

39.1805 471

39.1896 490

39.1987 481

39.2078 442

39.2169 458

39.2260 505

39.2350 466

39.2441 469

39.2532 500

39.2623 516

39.2714 462

39.2805 517

39.2896 518

39.2986 475

39.3077 505

39.3168 431

39.3259 494

39.3350 486

39.3441 479

39.3532 469

39.3622 463

39.3713 436

39.3804 460

39.3895 513

39.3986 489

39.4077 475

39.4167 461

39.4258 512

39.4349 470

39.4440 485

39.4531 491

39.4622 505

39.4713 505

39.4803 524

39.4894 488

39.4985 500

39.5076 461

39.5167 457

39.5258 479

39.5348 476

39.5439 478

39.5530 487

39.5621 517

39.5712 453

39.5803 489

39.5894 444

39.5984 479

39.6075 464

39.6166 480

39.6257 477

39.6348 442

39.6439 488

39.6530 476

39.6620 481

39.6711 453

39.6802 453

39.6893 475

39.6984 512

39.7075 512

39.7165 479

39.7256 419

39.7347 458

39.7438 466

39.7529 496

39.7620 454

39.7711 525

39.7801 479

39.7892 483

39.7983 474

39.8074 446

39.8165 496

39.8256 452

39.8346 465

39.8437 486

39.8528 436

39.8619 435

39.8710 449

39.8801 483

39.8892 471

39.8982 467

39.9073 473

39.9164 471

39.9255 487

39.9346 452

39.9437 480

39.9528 437

39.9618 474

39.9709 484

39.9800 473

39.9891 439

39.9982 489

40.0073 481

40.0163 482

40.0254 470

40.0345 451

40.0436 489

40.0527 465

40.0618 496

40.0709 461

40.0799 432

40.0890 443

40.0981 449

40.1072 462

40.1163 469

40.1254 453

40.1344 466

40.1435 529

40.1526 478

40.1617 425

40.1708 493

40.1799 466

40.1890 493

40.1980 497

40.2071 452

40.2162 501

40.2253 468

40.2344 515

40.2435 452

40.2526 496

40.2616 465

40.2707 493

40.2798 524

40.2889 473

40.2980 484

40.3071 479

40.3161 447

40.3252 481

40.3343 472

40.3434 449

40.3525 457

40.3616 499

40.3707 435

40.3797 495

40.3888 470

40.3979 517

40.4070 479

40.4161 449

40.4252 466

40.4342 452

40.4433 425

40.4524 465

40.4615 497

40.4706 456

40.4797 442

40.4888 487

40.4978 485

40.5069 474

40.5160 508

40.5251 481

40.5342 474

40.5433 457

40.5524 460

40.5614 473

40.5705 462

40.5796 449

40.5887 490

40.5978 495

40.6069 484

40.6159 502

40.6250 473

40.6341 487

40.6432 423

40.6523 504

40.6614 505

40.6705 480

40.6795 445

40.6886 453

40.6977 443

40.7068 454

40.7159 466

40.7250 454

40.7340 446

40.7431 447

40.7522 486

40.7613 450

40.7704 467

40.7795 479

40.7886 449

40.7976 470

40.8067 473

40.8158 462

40.8249 475

40.8340 432

40.8431 461

40.8522 440

40.8612 446

40.8703 505

40.8794 462

40.8885 468

40.8976 463

40.9067 479

40.9157 416

40.9248 457

40.9339 460

40.9430 461

40.9521 445

40.9612 455

40.9703 468

40.9793 482

40.9884 469

40.9975 469

41.0066 516

41.0157 479

41.0248 481

41.0338 484

41.0429 456

41.0520 459

41.0611 435

41.0702 472

41.0793 444

41.0884 448

41.0974 464

41.1065 473

41.1156 498

41.1247 446

41.1338 429

41.1429 464

41.1520 469

41.1610 444

41.1701 485

41.1792 446

41.1883 490

41.1974 510

41.2065 480

41.2155 442

41.2246 485

41.2337 441

41.2428 450

41.2519 410

41.2610 468

41.2701 461

41.2791 445

41.2882 493

41.2973 484

41.3064 446

41.3155 485

41.3246 432

41.3336 460

41.3427 461

41.3518 464

41.3609 446

41.3700 473

41.3791 476

41.3882 478

41.3972 451

41.4063 477

41.4154 457

41.4245 457

41.4336 459

41.4427 464

41.4518 481

41.4608 475

41.4699 447

41.4790 460

41.4881 469

41.4972 454

41.5063 432

41.5153 444

41.5244 465

41.5335 449

41.5426 466

41.5517 464

41.5608 481

41.5699 454

41.5789 455

41.5880 453

41.5971 469

41.6062 452

41.6153 448

41.6244 479

41.6334 434

41.6425 457

41.6516 444

41.6607 492

41.6698 439

41.6789 435

41.6880 510

41.6970 407

41.7061 467

41.7152 456

41.7243 446

41.7334 444

41.7425 413

41.7516 402

41.7606 434

41.7697 425

41.7788 465

41.7879 453

41.7970 414

41.8061 447

41.8151 507

41.8242 464

41.8333 452

41.8424 462

41.8515 478

41.8606 490

41.8697 478

41.8787 456

41.8878 418

41.8969 459

41.9060 437

41.9151 465

41.9242 442

41.9332 481

41.9423 439

41.9514 461

41.9605 439

41.9696 474

41.9787 480

41.9878 416

41.9968 432

42.0059 458

42.0150 472

42.0241 478

42.0332 429

42.0423 476

42.0514 452

42.0604 432

42.0695 490

42.0786 414

42.0877 458

42.0968 463

42.1059 446

42.1149 476

42.1240 475

42.1331 431

42.1422 432

42.1513 466

42.1604 444

42.1695 446

42.1785 457

42.1876 438

42.1967 472

42.2058 449

42.2149 422

42.2240 451

42.2330 459

42.2421 456

42.2512 509

42.2603 456

42.2694 427

42.2785 460

42.2876 438

42.2966 454

42.3057 474

42.3148 440

42.3239 437

42.3330 435

42.3421 437

42.3512 435

42.3602 404

42.3693 447

42.3784 445

42.3875 452

42.3966 473

42.4057 432

42.4147 483

42.4238 417

42.4329 446

42.4420 473

42.4511 437

42.4602 444

42.4693 475

42.4783 469

42.4874 483

42.4965 472

42.5056 473

42.5147 483

42.5238 476

42.5328 477

42.5419 410

42.5510 444

42.5601 437

42.5692 468

42.5783 418

42.5874 472

42.5964 446

42.6055 451

42.6146 467

42.6237 428

42.6328 425

42.6419 460

42.6510 402

42.6600 471

42.6691 483

42.6782 413

42.6873 465

42.6964 434

42.7055 437

42.7145 460

42.7236 449

42.7327 453

42.7418 457

42.7509 419

42.7600 421

42.7691 444

42.7781 434

42.7872 494

42.7963 461

42.8054 455

42.8145 454

42.8236 416

42.8326 418

42.8417 440

42.8508 433

42.8599 457

42.8690 443

42.8781 413

42.8872 431

42.8962 416

42.9053 465

42.9144 409

42.9235 432

42.9326 429

42.9417 422

42.9508 472

42.9598 482

42.9689 451

42.9780 463

42.9871 442

42.9962 457

43.0053 436

43.0143 427

43.0234 416

43.0325 436

43.0416 473

43.0507 436

43.0598 420

43.0689 444

43.0779 467

43.0870 411

43.0961 468

43.1052 471

43.1143 425

43.1234 470

43.1324 483

43.1415 450

43.1506 443

43.1597 484

43.1688 447

43.1779 420

43.1870 430

43.1960 479

43.2051 459

43.2142 409

43.2233 423

43.2324 422

43.2415 430

43.2506 461

43.2596 430

43.2687 460

43.2778 468

43.2869 447

43.2960 449

43.3051 447

43.3141 441

43.3232 474

43.3323 427

43.3414 440

43.3505 458

43.3596 443

43.3687 435

43.3777 463

43.3868 448

43.3959 434

43.4050 445

43.4141 430

43.4232 439

43.4322 417

43.4413 434

43.4504 459

43.4595 450

43.4686 439

43.4777 472

43.4868 417

43.4958 457

43.5049 439

43.5140 443

43.5231 426

43.5322 492

43.5413 456

43.5504 433

43.5594 477

43.5685 408

43.5776 482

43.5867 426

43.5958 439

43.6049 449

43.6139 424

43.6230 465

43.6321 451

43.6412 476

43.6503 419

43.6594 430

43.6685 434

43.6775 435

43.6866 414

43.6957 446

43.7048 442

43.7139 443

43.7230 446

43.7320 457

43.7411 477

43.7502 458

43.7593 416

43.7684 430

43.7775 443

43.7866 435

43.7956 462

43.8047 437

43.8138 445

43.8229 428

43.8320 406

43.8411 426

43.8502 467

43.8592 482

43.8683 481

43.8774 401

43.8865 455

43.8956 448

43.9047 437

43.9137 460

43.9228 437

43.9319 461

43.9410 439

43.9501 436

43.9592 444

43.9683 418

43.9773 448

43.9864 445

43.9955 437

44.0046 425

44.0137 455

44.0228 472

44.0318 422

44.0409 433

44.0500 433

44.0591 404

44.0682 425

44.0773 457

44.0864 415

44.0954 430

44.1045 418

44.1136 418

44.1227 438

44.1318 439

44.1409 427

44.1500 447

44.1590 470

44.1681 452

44.1772 405

44.1863 452

44.1954 443

44.2045 481

44.2135 442

44.2226 467

44.2317 456

44.2408 428

44.2499 430

44.2590 445

44.2681 428

44.2771 422

44.2862 442

44.2953 449

44.3044 421

44.3135 428

44.3226 439

44.3316 449

44.3407 433

44.3498 460

44.3589 416

44.3680 442

44.3771 414

44.3862 398

44.3952 454

44.4043 421

44.4134 445

44.4225 445

44.4316 437

44.4407 460

44.4498 451

44.4588 419

44.4679 431

44.4770 434

44.4861 455

44.4952 461

44.5043 478

44.5133 450

44.5224 405

44.5315 458

44.5406 449

44.5497 440

44.5588 424

44.5679 443

44.5769 421

44.5860 438

44.5951 438

44.6042 426

44.6133 389

44.6224 455

44.6314 407

44.6405 453

44.6496 439

44.6587 465

44.6678 471

44.6769 488

44.6860 454

44.6950 452

44.7041 410

44.7132 416

44.7223 408

44.7314 404

44.7405 423

44.7496 446

44.7586 456

44.7677 427

44.7768 464

44.7859 444

44.7950 394

44.8041 442

44.8131 397

44.8222 420

44.8313 461

44.8404 411

44.8495 458

44.8586 419

44.8677 402

44.8767 461

44.8858 423

44.8949 436

44.9040 408

44.9131 467

44.9222 435

44.9312 461

44.9403 459

44.9494 423

44.9585 461

44.9676 427

44.9767 417

44.9858 442

44.9948 417

45.0039 441

45.0130 491

45.0221 462

45.0312 423

45.0403 421

45.0494 412

45.0584 437

45.0675 407

45.0766 450

45.0857 423

45.0948 469

45.1039 426

45.1129 422

45.1220 432

45.1311 412

45.1402 433

45.1493 457

45.1584 402

45.1675 393

45.1765 434

45.1856 436

45.1947 446

45.2038 412

45.2129 432

45.2220 454

45.2310 456

45.2401 431

45.2492 455

45.2583 407

45.2674 448

45.2765 435

45.2856 426

45.2946 409

45.3037 418

45.3128 403

45.3219 387

45.3310 436

45.3401 435

45.3492 426

45.3582 443

45.3673 416

45.3764 443

45.3855 440

45.3946 399

45.4037 461

45.4127 434

45.4218 414

45.4309 464

45.4400 415

45.4491 453

45.4582 403

45.4673 401

45.4763 422

45.4854 466

45.4945 435

45.5036 401

45.5127 443

45.5218 437

45.5308 439

45.5399 407

45.5490 425

45.5581 421

45.5672 446

45.5763 436

45.5854 442

45.5944 425

45.6035 399

45.6126 446

45.6217 441

45.6308 408

45.6399 425

45.6490 385

45.6580 425

45.6671 416

45.6762 409

45.6853 402

45.6944 385

45.7035 412

45.7125 433

45.7216 448

45.7307 405

45.7398 417

45.7489 412

45.7580 415

45.7671 430

45.7761 417

45.7852 422

45.7943 433

45.8034 425

45.8125 460

45.8216 450

45.8306 407

45.8397 391

45.8488 415

45.8579 442

45.8670 416

45.8761 401

45.8852 467

45.8942 415

45.9033 408

45.9124 420

45.9215 451

45.9306 430

45.9397 425

45.9488 438

45.9578 423

45.9669 431

45.9760 420

45.9851 401

45.9942 410

46.0033 409

46.0123 391

46.0214 370

46.0305 406

46.0396 380

46.0487 416

46.0578 428

46.0669 424

46.0759 410

46.0850 419

46.0941 421

46.1032 423

46.1123 385

46.1214 422

46.1304 453

46.1395 415

46.1486 450

46.1577 437

46.1668 465

46.1759 410

46.1850 400

46.1940 385

46.2031 432

46.2122 375

46.2213 423

46.2304 433

46.2395 405

46.2486 447

46.2576 428

46.2667 420

46.2758 438

46.2849 415

46.2940 427

46.3031 373

46.3121 414

46.3212 426

46.3303 436

46.3394 402

46.3485 443

46.3576 423

46.3667 364

46.3757 397

46.3848 409

46.3939 421

46.4030 408

46.4121 435

46.4212 400

46.4302 413

46.4393 431

46.4484 420

46.4575 415

46.4666 444

46.4757 443

46.4848 456

46.4938 444

46.5029 419

46.5120 421

46.5211 400

46.5302 438

46.5393 426

46.5484 395

46.5574 403

46.5665 402

46.5756 420

46.5847 417

46.5938 446

46.6029 419

46.6119 392

46.6210 423

46.6301 442

46.6392 373

46.6483 440

46.6574 428

46.6665 434

46.6755 396

46.6846 428

46.6937 427

46.7028 376

46.7119 394

46.7210 437

46.7300 399

46.7391 445

46.7482 393

46.7573 426

46.7664 403

46.7755 421

46.7846 442

46.7936 447

46.8027 396

46.8118 425

46.8209 420

46.8300 421

46.8391 390

46.8482 367

46.8572 399

46.8663 448

46.8754 370

46.8845 401

46.8936 419

46.9027 438

46.9117 404

46.9208 398

46.9299 444

46.9390 420

46.9481 415

46.9572 399

46.9663 421

46.9753 405

46.9844 422

46.9935 432

47.0026 389

47.0117 400

47.0208 396

47.0298 432

47.0389 407

47.0480 383

47.0571 401

47.0662 433

47.0753 397

47.0844 436

47.0934 402

47.1025 400

47.1116 408

47.1207 440

47.1298 425

47.1389 409

47.1480 424

47.1570 434

47.1661 423

47.1752 392

47.1843 397

47.1934 409

47.2025 410

47.2115 433

47.2206 439

47.2297 404

47.2388 436

47.2479 418

47.2570 402

47.2661 419

47.2751 424

47.2842 420

47.2933 425

47.3024 421

47.3115 439

47.3206 399

47.3296 435

47.3387 404

47.3478 429

47.3569 398

47.3660 400

47.3751 418

47.3842 380

47.3932 405

47.4023 414

47.4114 382

47.4205 410

47.4296 439

47.4387 419

47.4478 422

47.4568 383

47.4659 395

47.4750 418

47.4841 394

47.4932 419

47.5023 413

47.5113 386

47.5204 379

47.5295 402

47.5386 411

47.5477 429

47.5568 402

47.5659 418

47.5749 424

47.5840 396

47.5931 398

47.6022 391

47.6113 408

47.6204 469

47.6294 418

47.6385 382

47.6476 428

47.6567 392

47.6658 408

47.6749 379

47.6840 406

47.6930 410

47.7021 386

47.7112 413

47.7203 382

47.7294 417

47.7385 425

47.7476 378

47.7566 417

47.7657 429

47.7748 391

47.7839 406

47.7930 408

47.8021 412

47.8111 367

47.8202 419

47.8293 409

47.8384 457

47.8475 426

47.8566 370

47.8657 366

47.8747 399

47.8838 420

47.8929 401

47.9020 442

47.9111 400

47.9202 388

47.9292 422

47.9383 403

47.9474 404

47.9565 376

47.9656 388

47.9747 400

47.9838 366

47.9928 445

48.0019 405

48.0110 394

48.0201 375

48.0292 405

48.0383 405

48.0474 429

48.0564 415

48.0655 394

48.0746 409

48.0837 419

48.0928 398

48.1019 436

48.1109 394

48.1200 419

48.1291 401

48.1382 381

48.1473 408

48.1564 408

48.1655 404

48.1745 398

48.1836 389

48.1927 370

48.2018 391

48.2109 405

48.2200 397

48.2290 399

48.2381 407

48.2472 381

48.2563 403

48.2654 370

48.2745 385

48.2836 392

48.2926 399

48.3017 433

48.3108 379

48.3199 397

48.3290 422

48.3381 415

48.3472 426

48.3562 406

48.3653 403

48.3744 400

48.3835 370

48.3926 384

48.4017 423

48.4107 379

48.4198 390

48.4289 367

48.4380 386

48.4471 416

48.4562 400

48.4653 411

48.4743 416

48.4834 387

48.4925 441

48.5016 422

48.5107 396

48.5198 392

48.5288 438

48.5379 394

48.5470 351

48.5561 407

48.5652 376

48.5743 393

48.5834 409

48.5924 389

48.6015 431

48.6106 404

48.6197 365

48.6288 422

48.6379 386

48.6470 393

48.6560 374

48.6651 367

48.6742 376

48.6833 376

48.6924 418

48.7015 436

48.7105 399

48.7196 386

48.7287 418

48.7378 389

48.7469 368

48.7560 370

48.7651 423

48.7741 396

48.7832 398

48.7923 395

48.8014 412

48.8105 408

48.8196 413

48.8286 379

48.8377 425

48.8468 394

48.8559 383

48.8650 400

48.8741 418

48.8832 370

48.8922 400

48.9013 381

48.9104 391

48.9195 391

48.9286 413

48.9377 390

48.9468 407

48.9558 389

48.9649 406

48.9740 419

48.9831 417

48.9922 361

49.0013 414

49.0103 415

49.0194 427

49.0285 456

49.0376 405

49.0467 409

49.0558 386

49.0649 373

49.0739 428

49.0830 426

49.0921 408

49.1012 368

49.1103 396

49.1194 390

49.1284 410

49.1375 433

49.1466 410

49.1557 386

49.1648 432

49.1739 386

49.1830 378

49.1920 406

49.2011 429

49.2102 360

49.2193 412

49.2284 378

49.2375 384

49.2466 426

49.2556 399

49.2647 388

49.2738 381

49.2829 384

49.2920 351

49.3011 389

49.3101 401

49.3192 367

49.3283 370

49.3374 389

49.3465 407

49.3556 359

49.3647 386

49.3737 441

49.3828 428

49.3919 412

49.4010 427

49.4101 382

49.4192 433

49.4282 386

49.4373 407

49.4464 405

49.4555 367

49.4646 396

49.4737 392

49.4828 419

49.4918 383

49.5009 387

49.5100 400

49.5191 375

49.5282 393

49.5373 391

49.5464 397

49.5554 379

49.5645 402

49.5736 400

49.5827 377

49.5918 400

49.6009 418

49.6099 376

49.6190 404

49.6281 355

49.6372 392

49.6463 383

49.6554 397

49.6645 427

49.6735 410

49.6826 387

49.6917 367

49.7008 400

49.7099 380

49.7190 403

49.7280 353

49.7371 405

49.7462 386

49.7553 419

49.7644 408

49.7735 379

49.7826 407

49.7916 433

49.8007 407

49.8098 440

49.8189 386

49.8280 409

49.8371 390

49.8462 422

49.8552 383

49.8643 375

49.8734 401

49.8825 416

49.8916 370

49.9007 391

49.9097 381

49.9188 378

49.9279 396

49.9370 357

49.9461 389

49.9552 407

49.9643 404

49.9733 385

49.9824 393

49.9915 370

50.0006 424

50.0097 369

50.0188 383

50.0278 394

50.0369 396

50.0460 418

50.0551 404

50.0642 364

50.0733 415

50.0824 371

50.0914 357

50.1005 395

50.1096 388

50.1187 360

50.1278 380

50.1369 384

50.1460 392

50.1550 375

50.1641 395

50.1732 391

50.1823 404

50.1914 368

50.2005 370

50.2095 348

50.2186 388

50.2277 369

50.2368 352

50.2459 392

50.2550 389

50.2641 383

50.2731 395

50.2822 341

50.2913 387

50.3004 380

50.3095 380

50.3186 393

50.3276 382

50.3367 370

50.3458 378

50.3549 381

50.3640 420

50.3731 387

50.3822 383

50.3912 368

50.4003 371

50.4094 397

50.4185 380

50.4276 433

50.4367 374

50.4458 392

50.4548 366

50.4639 369

50.4730 409

50.4821 356

50.4912 386

50.5003 380

50.5093 376

50.5184 429

50.5275 354

50.5366 373

50.5457 403

50.5548 363

50.5639 389

50.5729 388

50.5820 360

50.5911 421

50.6002 372

50.6093 369

50.6184 425

50.6274 377

50.6365 424

50.6456 390

50.6547 372

50.6638 369

50.6729 410

50.6820 388

50.6910 372

50.7001 390

50.7092 421

50.7183 389

50.7274 385

50.7365 400

50.7456 386

50.7546 390

50.7637 370

50.7728 384

50.7819 433

50.7910 393

50.8001 410

50.8091 405

50.8182 373

50.8273 328

50.8364 407

50.8455 361

50.8546 403

50.8637 358

50.8727 359

50.8818 390

50.8909 412

50.9000 389

50.9091 376

50.9182 402

50.9272 385

50.9363 408

50.9454 388

50.9545 350

50.9636 397

50.9727 402

50.9818 432

50.9908 385

50.9999 364

51.0090 400

51.0181 425

51.0272 377

51.0363 377

51.0454 418

51.0544 393

51.0635 334

51.0726 436

51.0817 385

51.0908 380

51.0999 394

51.1089 378

51.1180 369

51.1271 385

51.1362 377

51.1453 386

51.1544 398

51.1635 371

51.1725 385

51.1816 400

51.1907 397

51.1998 371

51.2089 376

51.2180 394

51.2270 408

51.2361 372

51.2452 363

51.2543 347

51.2634 349

51.2725 371

51.2816 392

51.2906 398

51.2997 411

51.3088 359

51.3179 387

51.3270 410

51.3361 370

51.3452 384

51.3542 368

51.3633 377

51.3724 341

51.3815 371

51.3906 389

51.3997 380

51.4087 381

51.4178 395

51.4269 386

51.4360 412

51.4451 405

51.4542 383

51.4633 420

51.4723 385

51.4814 376

51.4905 354

51.4996 418

51.5087 349

51.5178 415

51.5268 357

51.5359 355

51.5450 361

51.5541 373

51.5632 364

51.5723 376

51.5814 369

51.5904 385

51.5995 403

51.6086 385

51.6177 389

51.6268 361

51.6359 388

51.6450 375

51.6540 406

51.6631 364

51.6722 346

51.6813 401

51.6904 403

51.6995 372

51.7085 393

51.7176 371

51.7267 347

51.7358 357

51.7449 378

51.7540 372

51.7631 373

51.7721 419

51.7812 368

51.7903 410

51.7994 384

51.8085 355

51.8176 379

51.8266 386

51.8357 385

51.8448 410

51.8539 398

51.8630 388

51.8721 379

51.8812 380

51.8902 398

51.8993 375

51.9084 380

51.9175 375

51.9266 381

51.9357 368

51.9448 375

51.9538 380

51.9629 426

51.9720 411

51.9811 378

51.9902 405

51.9993 376

52.0083 368

52.0174 383

52.0265 354

52.0356 394

52.0447 334

52.0538 389

52.0629 380

52.0719 400

52.0810 392

52.0901 346

52.0992 382

52.1083 373

52.1174 325

52.1264 386

52.1355 381

52.1446 363

52.1537 394

52.1628 408

52.1719 394

52.1810 351

52.1900 347

52.1991 369

52.2082 373

52.2173 376

52.2264 357

52.2355 371

52.2446 394

52.2536 379

52.2627 401

52.2718 357

52.2809 385

52.2900 389

52.2991 372

52.3081 371

52.3172 389

52.3263 363

52.3354 411

52.3445 363

52.3536 397

52.3627 379

52.3717 387

52.3808 360

52.3899 349

52.3990 376

52.4081 355

52.4172 338

52.4262 377

52.4353 366

52.4444 384

52.4535 356

52.4626 389

52.4717 395

52.4808 404

52.4898 398

52.4989 365

52.5080 374

52.5171 383

52.5262 381

52.5353 395

52.5444 389

52.5534 381

52.5625 372

52.5716 362

52.5807 335

52.5898 360

52.5989 383

52.6079 384

52.6170 351

52.6261 397

52.6352 361

52.6443 387

52.6534 393

52.6625 356

52.6715 349

52.6806 383

52.6897 385

52.6988 360

52.7079 401

52.7170 387

52.7260 399

52.7351 372

52.7442 367

52.7533 365

52.7624 381

52.7715 407

52.7806 386

52.7896 393

52.7987 379

52.8078 387

52.8169 373

52.8260 369

52.8351 369

52.8442 374

52.8532 362

52.8623 358

52.8714 366

52.8805 374

52.8896 355

52.8987 397

52.9077 367

52.9168 387

52.9259 391

52.9350 385

52.9441 375

52.9532 359

52.9623 386

52.9713 392

52.9804 391

52.9895 354

52.9986 355

53.0077 373

53.0168 351

53.0258 403

53.0349 369

53.0440 359

53.0531 386

53.0622 396

53.0713 395

53.0804 367

53.0894 363

53.0985 365

53.1076 365

53.1167 381

53.1258 358

53.1349 404

53.1440 359

53.1530 369

53.1621 394

53.1712 380

53.1803 365

53.1894 348

53.1985 370

53.2075 350

53.2166 374

53.2257 350

53.2348 370

53.2439 349

53.2530 372

53.2621 413

53.2711 358

53.2802 377

53.2893 346

53.2984 374

53.3075 374

53.3166 366

53.3256 369

53.3347 385

53.3438 358

53.3529 360

53.3620 360

53.3711 369

53.3802 380

53.3892 420

53.3983 362

53.4074 395

53.4165 369

53.4256 377

53.4347 379

53.4438 380

53.4528 361

53.4619 339

53.4710 387

53.4801 375

53.4892 381

53.4983 355

53.5073 386

53.5164 387

53.5255 385

53.5346 413

53.5437 341

53.5528 420

53.5619 378

53.5709 379

53.5800 388

53.5891 369

53.5982 407

53.6073 394

53.6164 375

53.6254 359

53.6345 377

53.6436 361

53.6527 376

53.6618 390

53.6709 412

53.6800 333

53.6890 354

53.6981 383

53.7072 367

53.7163 370

53.7254 407

53.7345 352

53.7436 388

53.7526 359

53.7617 383

53.7708 391

53.7799 378

53.7890 374

53.7981 399

53.8071 374

53.8162 342

53.8253 382

53.8344 392

53.8435 416

53.8526 395

53.8617 394

53.8707 334

53.8798 379

53.8889 381

53.8980 371

53.9071 392

53.9162 343

53.9252 356

53.9343 355

53.9434 384

53.9525 371

53.9616 364

53.9707 370

53.9798 363

53.9888 406

53.9979 373

54.0070 367

54.0161 375

54.0252 361

54.0343 391

54.0434 356

54.0524 341

54.0615 352

54.0706 368

54.0797 388

54.0888 353

54.0979 371

54.1069 364

54.1160 349

54.1251 377

54.1342 382

54.1433 392

54.1524 379

54.1615 366

54.1705 366

54.1796 370

54.1887 371

54.1978 382

54.2069 384

54.2160 344

54.2250 335

54.2341 366

54.2432 354

54.2523 329

54.2614 345

54.2705 402

54.2796 333

54.2886 414

54.2977 366

54.3068 381

54.3159 367

54.3250 365

54.3341 382

54.3432 382

54.3522 356

54.3613 363

54.3704 364

54.3795 331

54.3886 381

54.3977 387

54.4067 353

54.4158 395

54.4249 365

54.4340 359

54.4431 391

54.4522 364

54.4613 390

54.4703 309

54.4794 371

54.4885 374

54.4976 340

54.5067 353

54.5158 366

54.5248 383

54.5339 365

54.5430 347

54.5521 387

54.5612 340

54.5703 373

54.5794 356

54.5884 372

54.5975 375

54.6066 355

54.6157 328

54.6248 363

54.6339 370

54.6430 393

54.6520 340

54.6611 397

54.6702 417

54.6793 384

54.6884 352

54.6975 365

54.7065 368

54.7156 368

54.7247 382

54.7338 384

54.7429 379

54.7520 369

54.7611 357

54.7701 396

54.7792 344

54.7883 386

54.7974 388

54.8065 344

54.8156 321

54.8246 356

54.8337 355

54.8428 393

54.8519 333

54.8610 341

54.8701 314

54.8792 352

54.8882 375

54.8973 362

54.9064 342

54.9155 337

54.9246 380

54.9337 362

54.9428 323

54.9518 368

54.9609 360

54.9700 396

54.9791 350

54.9882 335

54.9973 362

55.0063 367

55.0154 371

55.0245 381

55.0336 389

55.0427 405

55.0518 348

55.0609 347

55.0699 390

55.0790 361

55.0881 337

55.0972 378

55.1063 336

55.1154 370

55.1244 366

55.1335 335

55.1426 333

55.1517 409

55.1608 319

55.1699 347

55.1790 372

55.1880 337

55.1971 392

55.2062 420

55.2153 380

55.2244 383

55.2335 360

55.2426 377

55.2516 349

55.2607 368

55.2698 393

55.2789 381

55.2880 367

55.2971 351

55.3061 364

55.3152 365

55.3243 392

55.3334 365

55.3425 363

55.3516 325

55.3607 397

55.3697 382

55.3788 376

55.3879 351

55.3970 375

55.4061 359

55.4152 363

55.4243 385

55.4333 372

55.4424 399

55.4515 391

55.4606 389

55.4697 346

55.4788 362

55.4878 369

55.4969 389

55.5060 389

55.5151 351

55.5242 369

55.5333 371

55.5424 369

55.5514 375

55.5605 355

55.5696 326

55.5787 327

55.5878 346

55.5969 377

55.6059 370

55.6150 369

55.6241 398

55.6332 335

55.6423 384

55.6514 345

55.6605 335

55.6695 398

55.6786 348

55.6877 357

55.6968 338

55.7059 355

55.7150 353

55.7241 364

55.7331 349

55.7422 358

55.7513 350

55.7604 322

55.7695 353

55.7786 338

55.7876 364

55.7967 381

55.8058 387

55.8149 360

55.8240 343

55.8331 383

55.8422 332

55.8512 359

55.8603 348

55.8694 360

55.8785 364

55.8876 336

55.8967 353

55.9057 355

55.9148 356

55.9239 348

55.9330 342

55.9421 324

55.9512 334

55.9603 324

55.9693 339

55.9784 345

55.9875 376

55.9966 364

56.0057 332

56.0148 371

56.0239 387

56.0329 368

56.0420 375

56.0511 387

56.0602 384

56.0693 345

56.0784 346

56.0874 353

56.0965 342

56.1056 371

56.1147 340

56.1238 353

56.1329 332

56.1420 366

56.1510 356

56.1601 387

56.1692 362

56.1783 361

56.1874 372

56.1965 354

56.2055 345

56.2146 377

56.2237 384

56.2328 355

56.2419 354

56.2510 346

56.2601 354

56.2691 350

56.2782 365

56.2873 368

56.2964 364

56.3055 347

56.3146 362

56.3237 334

56.3327 405

56.3418 376

56.3509 371

56.3600 326

56.3691 345

56.3782 366

56.3872 377

56.3963 367

56.4054 365

56.4145 373

56.4236 358

56.4327 385

56.4418 336

56.4508 356

56.4599 360

56.4690 369

56.4781 357

56.4872 378

56.4963 360

56.5053 364

56.5144 342

56.5235 336

56.5326 394

56.5417 350

56.5508 336

56.5599 368

56.5689 365

56.5780 350

56.5871 354

56.5962 372

56.6053 351

56.6144 350

56.6235 359

56.6325 357

56.6416 385

56.6507 353

56.6598 374

56.6689 367

56.6780 370

56.6870 367

56.6961 325

56.7052 351

56.7143 320

56.7234 394

56.7325 363

56.7416 322

56.7506 383

56.7597 340

56.7688 319

56.7779 384

56.7870 376

56.7961 327

56.8051 325

56.8142 339

56.8233 359

56.8324 349

56.8415 364

56.8506 370

56.8597 345

56.8687 356

56.8778 368

56.8869 365

56.8960 362

56.9051 357

56.9142 368

56.9233 304

56.9323 359

56.9414 382

56.9505 367

56.9596 356

56.9687 366

56.9778 350

56.9868 338

56.9959 376

57.0050 355

57.0141 371

57.0232 353

57.0323 359

57.0414 378

57.0504 362

57.0595 353

57.0686 355

57.0777 337

57.0868 386

57.0959 354

57.1049 342

57.1140 359

57.1231 342

57.1322 306

57.1413 344

57.1504 352

57.1595 351

57.1685 354

57.1776 339

57.1867 368

57.1958 341

57.2049 363

57.2140 378

57.2231 363

57.2321 387

57.2412 350

57.2503 348

57.2594 335

57.2685 347

57.2776 349

57.2866 351

57.2957 356

57.3048 334

57.3139 323

57.3230 362

57.3321 376

57.3412 379

57.3502 338

57.3593 366

57.3684 346

57.3775 368

57.3866 347

57.3957 329

57.4047 352

57.4138 364

57.4229 371

57.4320 375

57.4411 334

57.4502 362

57.4593 347

57.4683 349

57.4774 365

57.4865 340

57.4956 345

57.5047 353

57.5138 345

57.5229 327

57.5319 361

57.5410 374

57.5501 345

57.5592 347

57.5683 397

57.5774 367

57.5864 382

57.5955 344

57.6046 363

57.6137 367

57.6228 346

57.6319 352

57.6410 356

57.6500 340

57.6591 357

57.6682 330

57.6773 350

57.6864 341

57.6955 367

57.7045 368

57.7136 352

57.7227 390

57.7318 358

57.7409 363

57.7500 354

57.7591 352

57.7681 349

57.7772 344

57.7863 322

57.7954 356

57.8045 338

57.8136 392

57.8227 345

57.8317 334

57.8408 377

57.8499 338

57.8590 370

57.8681 370

57.8772 357

57.8862 357

57.8953 362

57.9044 361

57.9135 361

57.9226 335

57.9317 345

57.9408 356

57.9498 405

57.9589 347

57.9680 355

57.9771 331

57.9862 358

57.9953 328

58.0043 387

58.0134 369

58.0225 363

58.0316 335

58.0407 379

58.0498 342

58.0589 333

58.0679 350

58.0770 357

58.0861 410

58.0952 360

58.1043 324

58.1134 356

58.1225 365

58.1315 346

58.1406 365

58.1497 339

58.1588 345

58.1679 362

58.1770 398

58.1860 339

58.1951 337

58.2042 340

58.2133 335

58.2224 348

58.2315 382

58.2406 374

58.2496 358

58.2587 323

58.2678 326

58.2769 352

58.2860 333

58.2951 359

58.3041 346

58.3132 306

58.3223 359

58.3314 345

58.3405 373

58.3496 335

58.3587 364

58.3677 341

58.3768 356

58.3859 392

58.3950 345

58.4041 362

58.4132 367

58.4223 361

58.4313 353

58.4404 344

58.4495 349

58.4586 378

58.4677 354

58.4768 302

58.4858 355

58.4949 362

58.5040 379

58.5131 367

58.5222 345

58.5313 348

58.5404 358

58.5494 346

58.5585 401

58.5676 363

58.5767 344

58.5858 356

58.5949 357

58.6039 331

58.6130 366

58.6221 335

58.6312 355

58.6403 361

58.6494 333

58.6585 366

58.6675 324

58.6766 366

58.6857 378

58.6948 371

58.7039 327

58.7130 341

58.7221 335

58.7311 357

58.7402 341

58.7493 360

58.7584 329

58.7675 337

58.7766 341

58.7856 366

58.7947 348

58.8038 349

58.8129 343

58.8220 376

58.8311 367

58.8402 347

58.8492 341

58.8583 385

58.8674 345

58.8765 336

58.8856 355

58.8947 342

58.9037 339

58.9128 356

58.9219 325

58.9310 348

58.9401 347

58.9492 333

58.9583 332

58.9673 344

58.9764 364

58.9855 382

58.9946 356

59.0037 366

59.0128 355

59.0219 319

59.0309 359

59.0400 398

59.0491 320

59.0582 341

59.0673 371

59.0764 360

59.0854 368

59.0945 340

59.1036 369

59.1127 374

59.1218 367

59.1309 357

59.1400 354

59.1490 353

59.1581 361

59.1672 379

59.1763 359

59.1854 353

59.1945 392

59.2035 358

59.2126 352

59.2217 340

59.2308 354

59.2399 370

59.2490 350

59.2581 359

59.2671 385

59.2762 335

59.2853 361

59.2944 377

59.3035 370

59.3126 360

59.3217 370

59.3307 354

59.3398 338

59.3489 360

59.3580 363

59.3671 332

59.3762 370

59.3852 370

59.3943 342

59.4034 355

59.4125 367

59.4216 383

59.4307 344

59.4398 355

59.4488 335

59.4579 357

59.4670 358

59.4761 383

59.4852 368

59.4943 344

59.5033 329

59.5124 364

59.5215 370

59.5306 362

59.5397 354

59.5488 341

59.5579 333

59.5669 373

59.5760 353

59.5851 343

59.5942 347

59.6033 343

59.6124 359

59.6215 354

59.6305 339

59.6396 349

59.6487 329

59.6578 374

59.6669 368

59.6760 334

59.6850 349

59.6941 300

59.7032 353

59.7123 345

59.7214 354

59.7305 355

59.7396 353

59.7486 354

59.7577 352

59.7668 351

59.7759 329

59.7850 395

59.7941 333

59.8031 376

59.8122 359

59.8213 349

59.8304 327

59.8395 378

59.8486 348

59.8577 332

59.8667 369

59.8758 360

59.8849 332

59.8940 378

59.9031 356

59.9122 357

59.9213 362

59.9303 370

59.9394 364

59.9485 373

59.9576 331

59.9667 321

59.9758 315

59.9848 372

59.9939 331

60.0030 359

60.0121 372

60.0212 329

60.0303 380

60.0394 301

60.0484 322

60.0575 333

60.0666 401

60.0757 343

60.0848 336

60.0939 328

60.1029 342

60.1120 366

60.1211 357

60.1302 376

60.1393 343

60.1484 337

60.1575 323

60.1665 357

60.1756 345

60.1847 361

60.1938 339

60.2029 351

60.2120 376

60.2211 356

60.2301 396

60.2392 323

60.2483 355

60.2574 358

60.2665 342

60.2756 349

60.2846 403

60.2937 346

60.3028 353

60.3119 315

60.3210 322

60.3301 335

60.3392 322

60.3482 363

60.3573 351

60.3664 330

60.3755 327

60.3846 377

60.3937 330

60.4027 357

60.4118 351

60.4209 315

60.4300 389

60.4391 331

60.4482 324

60.4573 382

60.4663 321

60.4754 338

60.4845 334

60.4936 360

60.5027 365

60.5118 353

60.5209 395

60.5299 364

60.5390 332

60.5481 369

60.5572 358

60.5663 371

60.5754 368

60.5844 321

60.5935 328

60.6026 350

60.6117 346

60.6208 337

60.6299 341

60.6390 359

60.6480 336

60.6571 371

60.6662 316

60.6753 334

60.6844 330

60.6935 336

60.7025 368

60.7116 355

60.7207 357

60.7298 366

60.7389 357

60.7480 341

60.7571 340

60.7661 352

60.7752 333

60.7843 354

60.7934 330

60.8025 351

60.8116 376

60.8207 340

60.8297 300

60.8388 347

60.8479 341

60.8570 342

60.8661 315

60.8752 366

60.8842 342

60.8933 323

60.9024 335

60.9115 327

60.9206 322

60.9297 353

60.9388 291

60.9478 364

60.9569 338

60.9660 352

60.9751 326

60.9842 347

60.9933 325

61.0023 371

61.0114 302

61.0205 347

61.0296 339

61.0387 352

61.0478 354

61.0569 344

61.0659 353

61.0750 355

61.0841 335

61.0932 301

61.1023 321

61.1114 332

61.1205 346

61.1295 334

61.1386 312

61.1477 317

61.1568 343

61.1659 390

61.1750 343

61.1840 324

61.1931 338

61.2022 350

61.2113 366

61.2204 362

61.2295 343

61.2386 369

61.2476 323

61.2567 321

61.2658 372

61.2749 330

61.2840 364

61.2931 300

61.3021 360

61.3112 350

61.3203 339

61.3294 325

61.3385 363

61.3476 328

61.3567 338

61.3657 326

61.3748 340

61.3839 386

61.3930 340

61.4021 339

61.4112 328

61.4203 326

61.4293 365

61.4384 364

61.4475 341

61.4566 336

61.4657 343

61.4748 324

61.4838 383

61.4929 357

61.5020 377

61.5111 308

61.5202 335

61.5293 341

61.5384 333

61.5474 351

61.5565 353

61.5656 315

61.5747 347

61.5838 354

61.5929 359

61.6019 364

61.6110 341

61.6201 361

61.6292 319

61.6383 348

61.6474 332

61.6565 330

61.6655 356

61.6746 349

61.6837 319

61.6928 347

61.7019 343

61.7110 323

61.7201 330

61.7291 338

61.7382 337

61.7473 343

61.7564 347

61.7655 357

61.7746 306

61.7836 347

61.7927 315

61.8018 309

61.8109 324

61.8200 358

61.8291 350

61.8382 327

61.8472 331

61.8563 371

61.8654 312

61.8745 324

61.8836 335

61.8927 354

61.9017 332

61.9108 379

61.9199 344

61.9290 295

61.9381 365

61.9472 355

61.9563 365

61.9653 341

61.9744 345

61.9835 323

61.9926 335

62.0017 349

62.0108 353

62.0199 364

62.0289 375

62.0380 342

62.0471 327

62.0562 316

62.0653 352

62.0744 376

62.0834 339

62.0925 368

62.1016 353

62.1107 336

62.1198 326

62.1289 348

62.1380 298

62.1470 338

62.1561 310

62.1652 359

62.1743 331

62.1834 350

62.1925 332

62.2015 331

62.2106 338

62.2197 360

62.2288 326

62.2379 315

62.2470 350

62.2561 338

62.2651 357

62.2742 381

62.2833 319

62.2924 329

62.3015 374

62.3106 351

62.3197 356

62.3287 301

62.3378 325

62.3469 320

62.3560 340

62.3651 308

62.3742 345

62.3832 338

62.3923 315

62.4014 325

62.4105 327

62.4196 337

62.4287 337

62.4378 345

62.4468 309

62.4559 317

62.4650 316

62.4741 347

62.4832 331

62.4923 351

62.5013 327

62.5104 357

62.5195 325

62.5286 288

62.5377 329

62.5468 327

62.5559 327

62.5649 336

62.5740 324

62.5831 343

62.5922 345

62.6013 360

62.6104 347

62.6195 353

62.6285 336

62.6376 342

62.6467 326

62.6558 356

62.6649 369

62.6740 334

62.6830 350

62.6921 301

62.7012 364

62.7103 327

62.7194 314

62.7285 369

62.7376 367

62.7466 314

62.7557 384

62.7648 336

62.7739 318

62.7830 345

62.7921 322

62.8011 317

62.8102 330

62.8193 342

62.8284 299

62.8375 342

62.8466 361

62.8557 376

62.8647 348

62.8738 355

62.8829 331

62.8920 311

62.9011 365

62.9102 321

62.9193 334

62.9283 332

62.9374 355

62.9465 349

62.9556 316

62.9647 314

62.9738 342

62.9828 327

62.9919 348

63.0010 318

63.0101 327

63.0192 357

63.0283 294

63.0374 344

63.0464 342

63.0555 298

63.0646 327

63.0737 346

63.0828 334

63.0919 354

63.1009 322

63.1100 330

63.1191 336

63.1282 345

63.1373 358

63.1464 302

63.1555 351

63.1645 357

63.1736 347

63.1827 314

63.1918 363

63.2009 307

63.2100 335

63.2191 357

63.2281 339

63.2372 326

63.2463 375

63.2554 341

63.2645 359

63.2736 317

63.2826 338

63.2917 355

63.3008 323

63.3099 370

63.3190 312

63.3281 336

63.3372 330

63.3462 314

63.3553 314

63.3644 343

63.3735 323

63.3826 349

63.3917 314

63.4007 336

63.4098 372

63.4189 346

63.4280 367

63.4371 326

63.4462 296

63.4553 347

63.4643 341

63.4734 354

63.4825 331

63.4916 357

63.5007 334

63.5098 363

63.5189 327

63.5279 359

63.5370 373

63.5461 355

63.5552 311

63.5643 355

63.5734 314

63.5824 345

63.5915 327

63.6006 310

63.6097 346

63.6188 331

63.6279 356

63.6370 320

63.6460 352

63.6551 319

63.6642 337

63.6733 329

63.6824 335

63.6915 336

63.7005 361

63.7096 341

63.7187 350

63.7278 284

63.7369 307

63.7460 328

63.7551 348

63.7641 345

63.7732 334

63.7823 299

63.7914 312

63.8005 299

63.8096 322

63.8187 311

63.8277 328

63.8368 326

63.8459 349

63.8550 347

63.8641 340

63.8732 328

63.8822 369

63.8913 294

63.9004 342

63.9095 313

63.9186 323

63.9277 357

63.9368 356

63.9458 356

63.9549 334

63.9640 340

63.9731 339

63.9822 357

63.9913 336

64.0003 335

64.0094 355

64.0185 334

64.0276 344

64.0367 358

64.0458 318

64.0549 303

64.0639 343

64.0730 352

64.0821 343

64.0912 319

64.1003 334

64.1094 337

64.1185 297

64.1275 297

64.1366 296

64.1457 312

64.1548 335

64.1639 342

64.1730 351

64.1820 332

64.1911 327

64.2002 353

64.2093 297

64.2184 325

64.2275 307

64.2366 325

64.2456 317

64.2547 309

64.2638 336

64.2729 313

64.2820 348

64.2911 323

64.3001 318

64.3092 327

64.3183 337

64.3274 330

64.3365 312

64.3456 307

64.3547 365

64.3637 347

64.3728 337

64.3819 317

64.3910 317

64.4001 325

64.4092 331

64.4183 307

64.4273 330

64.4364 329

64.4455 328

64.4546 348

64.4637 344

64.4728 364

64.4818 348

64.4909 327

64.5000 334

64.5091 327

64.5182 318

64.5273 304

64.5364 334

64.5454 343

64.5545 314

64.5636 335

64.5727 324

64.5818 338

64.5909 348

64.5999 294

64.6090 313

64.6181 324

64.6272 316

64.6363 331

64.6454 333

64.6545 388

64.6635 321

64.6726 328

64.6817 333

64.6908 346

64.6999 312

64.7090 318

64.7181 314

64.7271 359

64.7362 353

64.7453 328

64.7544 339

64.7635 316

64.7726 317

64.7816 323

64.7907 335

64.7998 342

64.8089 328

64.8180 328

64.8271 336

64.8362 324

64.8452 311

64.8543 348

64.8634 325

64.8725 324

64.8816 345

64.8907 339

64.8997 337

64.9088 325

64.9179 374

64.9270 315

64.9361 330

64.9452 311

64.9543 301

64.9633 312

64.9724 349

64.9815 341

64.9906 311

64.9997 352

65.0088 326

65.0179 321

65.0269 294

65.0360 305

65.0451 347

65.0542 349

65.0633 318

65.0724 352

65.0814 326

65.0905 328

65.0996 309

65.1087 305

65.1178 349

65.1269 325

65.1360 334

65.1450 324

65.1541 334

65.1632 310

65.1723 297

65.1814 314

65.1905 316

65.1995 367

65.2086 305

65.2177 307

65.2268 326

65.2359 347

65.2450 320

65.2541 330

65.2631 332

65.2722 374

65.2813 331

65.2904 298

65.2995 342

65.3086 323

65.3177 323

65.3267 319

65.3358 309

65.3449 340

65.3540 372

65.3631 342

65.3722 291

65.3812 309

65.3903 308

65.3994 331

65.4085 310

65.4176 324

65.4267 320

65.4358 341

65.4448 325

65.4539 308

65.4630 341

65.4721 337

65.4812 356

65.4903 323

65.4993 296

65.5084 334

65.5175 339

65.5266 332

65.5357 302

65.5448 333

65.5539 318

65.5629 307

65.5720 355

65.5811 371

65.5902 340

65.5993 337

65.6084 349

65.6175 299

65.6265 312

65.6356 316

65.6447 358

65.6538 342

65.6629 300

65.6720 351

65.6810 329

65.6901 325

65.6992 325

65.7083 346

65.7174 331

65.7265 323

65.7356 336

65.7446 331

65.7537 308

65.7628 324

65.7719 325

65.7810 326

65.7901 337

65.7991 346

65.8082 329

65.8173 322

65.8264 327

65.8355 321

65.8446 344

65.8537 304

65.8627 331

65.8718 305

65.8809 330

65.8900 340

65.8991 328

65.9082 335

65.9173 323

65.9263 310

65.9354 349

65.9445 340

65.9536 308

65.9627 318

65.9718 313

65.9808 356

65.9899 307

65.9990 315

66.0081 296

66.0172 350

66.0263 326

66.0354 349

66.0444 297

66.0535 290

66.0626 331

66.0717 344

66.0808 330

66.0899 296

66.0989 317

66.1080 321

66.1171 343

66.1262 316

66.1353 346

66.1444 327

66.1535 303

66.1625 324

66.1716 327

66.1807 337

66.1898 299

66.1989 333

66.2080 326

66.2171 319

66.2261 297

66.2352 334

66.2443 317

66.2534 321

66.2625 339

66.2716 324

66.2806 338

66.2897 315

66.2988 293

66.3079 327

66.3170 312

66.3261 290

66.3352 324

66.3442 332

66.3533 353

66.3624 333

66.3715 328

66.3806 298

66.3897 339

66.3987 329

66.4078 299

66.4169 327

66.4260 352

66.4351 334

66.4442 313

66.4533 281

66.4623 322

66.4714 337

66.4805 323

66.4896 331

66.4987 340

66.5078 335

66.5169 318

66.5259 314

66.5350 324

66.5441 322

66.5532 307

66.5623 326

66.5714 330

66.5804 335

66.5895 322

66.5986 314

66.6077 322

66.6168 286

66.6259 304

66.6350 311

66.6440 295

66.6531 360

66.6622 302

66.6713 304

66.6804 288

66.6895 321

66.6985 339

66.7076 336

66.7167 328

66.7258 302

66.7349 312

66.7440 338

66.7531 291

66.7621 320

66.7712 289

66.7803 275

66.7894 306

66.7985 314

66.8076 323

66.8167 322

66.8257 295

66.8348 276

66.8439 300

66.8530 358

66.8621 327

66.8712 314

66.8802 272

66.8893 303

66.8984 284

66.9075 297

66.9166 320

66.9257 345

66.9348 315

66.9438 280

66.9529 325

66.9620 305

66.9711 302

66.9802 300

66.9893 304

66.9983 309

67.0074 306

67.0165 311

67.0256 323

67.0347 304

67.0438 282

67.0529 283

67.0619 303

67.0710 282

67.0801 315

67.0892 326

67.0983 309

67.1074 325

67.1165 335

67.1255 298

67.1346 315

67.1437 307

67.1528 312

67.1619 324

67.1710 280

67.1800 322

67.1891 339

67.1982 301

67.2073 297

67.2164 301

67.2255 288

67.2346 301

67.2436 320

67.2527 317

67.2618 294

67.2709 298

67.2800 308

67.2891 324

67.2981 328

67.3072 331

67.3163 286

67.3254 333

67.3345 327

67.3436 296

67.3527 324

67.3617 317

67.3708 312

67.3799 357

67.3890 336

67.3981 321

67.4072 321

67.4163 288

67.4253 307

67.4344 286

67.4435 295

67.4526 335

67.4617 294

67.4708 339

67.4798 317

67.4889 312

67.4980 317

67.5071 329

67.5162 321

67.5253 310

67.5344 313

67.5434 273

67.5525 310

67.5616 314

67.5707 307

67.5798 314

67.5889 304

67.5979 320

67.6070 300

67.6161 324

67.6252 320

67.6343 321

67.6434 309

67.6525 320

67.6615 308

67.6706 300

67.6797 314

67.6888 318

67.6979 310

67.7070 311

67.7161 332

67.7251 278

67.7342 303

67.7433 327

67.7524 320

67.7615 343

67.7706 297

67.7796 317

67.7887 310

67.7978 323

67.8069 298

67.8160 327

67.8251 322

67.8342 333

67.8432 331

67.8523 314

67.8614 326

67.8705 336

67.8796 300

67.8887 299

67.8977 314

67.9068 305

67.9159 313

67.9250 295

67.9341 325

67.9432 325

67.9523 300

67.9613 343

67.9704 313

67.9795 293

67.9886 299

67.9977 300

68.0068 302

68.0159 299

68.0249 314

68.0340 330

68.0431 318

68.0522 321

68.0613 304

68.0704 304

68.0794 281

68.0885 302

68.0976 303

68.1067 328

68.1158 328

68.1249 347

68.1340 318

68.1430 327

68.1521 352

68.1612 332

68.1703 308

68.1794 332

68.1885 311

68.1975 307

68.2066 344

68.2157 309

68.2248 307

68.2339 320

68.2430 302

68.2521 304

68.2611 282

68.2702 317

68.2793 323

68.2884 300

68.2975 305

68.3066 326

68.3157 315

68.3247 343

68.3338 305

68.3429 299

68.3520 296

68.3611 282

68.3702 302

68.3792 309

68.3883 294

68.3974 333

68.4065 328

68.4156 295

68.4247 299

68.4338 328

68.4428 298

68.4519 313

68.4610 288

68.4701 286

68.4792 317

68.4883 319

68.4973 316

68.5064 284

68.5155 328

68.5246 332

68.5337 274

68.5428 316

68.5519 307

68.5609 287

68.5700 309

68.5791 272

68.5882 332

68.5973 295

68.6064 315

68.6155 279

68.6245 342

68.6336 309

68.6427 274

68.6518 322

68.6609 332

68.6700 355

68.6790 315

68.6881 353

68.6972 308

68.7063 307

68.7154 275

68.7245 329

68.7336 317

68.7426 322

68.7517 338

68.7608 335

68.7699 330

68.7790 279

68.7881 336

68.7971 308

68.8062 315

68.8153 292

68.8244 329

68.8335 299

68.8426 325

68.8517 293

68.8607 351

68.8698 301

68.8789 341

68.8880 315

68.8971 314

68.9062 296

68.9153 320

68.9243 303

68.9334 291

68.9425 329

68.9516 311

68.9607 334

68.9698 321

68.9788 313

68.9879 316

68.9970 299

69.0061 284

69.0152 320

69.0243 285

69.0334 310

69.0424 292

69.0515 318

69.0606 276

69.0697 308

69.0788 319

69.0879 285

69.0969 311

69.1060 297

69.1151 315

69.1242 300

69.1333 315

69.1424 336

69.1515 304

69.1605 320

69.1696 323

69.1787 310

69.1878 292

69.1969 323

69.2060 332

69.2151 301

69.2241 323

69.2332 244

69.2423 307

69.2514 305

69.2605 323

69.2696 305

69.2786 305

69.2877 328

69.2968 319

69.3059 306

69.3150 321

69.3241 303

69.3332 308

69.3422 332

69.3513 300

69.3604 330

69.3695 281

69.3786 328

69.3877 309

69.3967 319

69.4058 288

69.4149 331

69.4240 351

69.4331 308

69.4422 331

69.4513 338

69.4603 321

69.4694 306

69.4785 302

69.4876 337

69.4967 305

69.5058 320

69.5149 292

69.5239 285

69.5330 355

69.5421 302

69.5512 322

69.5603 325

69.5694 317

69.5784 292

69.5875 330

69.5966 320

69.6057 304

69.6148 297

69.6239 263

69.6330 275

69.6420 293

69.6511 325

69.6602 309

69.6693 292

69.6784 263

69.6875 309

69.6965 315

69.7056 299

69.7147 310

69.7238 274

69.7329 321

69.7420 308

69.7511 333

69.7601 322

69.7692 292

69.7783 286

69.7874 320

69.7965 294

69.8056 306

69.8147 319

69.8237 274

69.8328 320

69.8419 309

69.8510 325

69.8601 298

69.8692 302

69.8782 304

69.8873 317

69.8964 309

69.9055 313

69.9146 318

69.9237 333

69.9328 316

69.9418 324

69.9509 295

69.9600 304

69.9691 344

69.9782 304

69.9873 321

69.9963 317

70.0054 281

70.0145 297

70.0236 295

70.0327 303

70.0418 305

70.0509 285

70.0599 333

70.0690 307

70.0781 289

70.0872 316

70.0963 306

70.1054 301

70.1145 297

70.1235 285

70.1326 301

70.1417 295

70.1508 291

70.1599 265

70.1690 319

70.1780 290

70.1871 280

70.1962 304

70.2053 318

70.2144 289

70.2235 348

70.2326 306

70.2416 307

70.2507 301

70.2598 317

70.2689 320

70.2780 319

70.2871 296

70.2961 332

70.3052 326

70.3143 309

70.3234 316

70.3325 291

70.3416 298

70.3507 299

70.3597 317

70.3688 295

70.3779 277

70.3870 295

70.3961 322

70.4052 294

70.4143 305

70.4233 315

70.4324 282

70.4415 308

70.4506 304

70.4597 299

70.4688 325

70.4778 297

70.4869 315

70.4960 297

70.5051 284

70.5142 293

70.5233 294

70.5324 301

70.5414 299

70.5505 297

70.5596 313

70.5687 306

70.5778 252

70.5869 292

70.5959 303

70.6050 282

70.6141 315

70.6232 280

70.6323 293

70.6414 346

70.6505 293

70.6595 288

70.6686 293

70.6777 290

70.6868 333

70.6959 276

70.7050 275

70.7141 295

70.7231 289

70.7322 337

70.7413 314

70.7504 320

70.7595 315

70.7686 303

70.7776 293

70.7867 280

70.7958 309

70.8049 306

70.8140 314

70.8231 348

70.8322 302

70.8412 302

70.8503 286

70.8594 294

70.8685 323

70.8776 304

70.8867 298

70.8957 291

70.9048 289

70.9139 303

70.9230 331

70.9321 326

70.9412 323

70.9503 279

70.9593 313

70.9684 290

70.9775 339

70.9866 272

70.9957 301

71.0048 307

71.0139 297

71.0229 274

71.0320 313

71.0411 329

71.0502 314

71.0593 283

71.0684 291

71.0774 316

71.0865 284

71.0956 256

71.1047 329

71.1138 292

71.1229 318

71.1320 306

71.1410 288

71.1501 278

71.1592 290

71.1683 312

71.1774 299

71.1865 329

71.1955 306

71.2046 316

71.2137 310

71.2228 281

71.2319 294

71.2410 293

71.2501 317

71.2591 304

71.2682 314

71.2773 282

71.2864 285

71.2955 297

71.3046 285

71.3137 274

71.3227 316

71.3318 350

71.3409 320

71.3500 305

71.3591 277

71.3682 318

71.3772 308

71.3863 298

71.3954 284

71.4045 330

71.4136 313

71.4227 311

71.4318 309

71.4408 293

71.4499 287

71.4590 299

71.4681 248

71.4772 333

71.4863 289

71.4953 273

71.5044 327

71.5135 302

71.5226 300

71.5317 316

71.5408 315

71.5499 311

71.5589 301

71.5680 272

71.5771 338

71.5862 314

71.5953 302

71.6044 295

71.6135 279

71.6225 288

71.6316 275

71.6407 311

71.6498 280

71.6589 282

71.6680 329

71.6770 305

71.6861 284

71.6952 297

71.7043 320

71.7134 314

71.7225 308

71.7316 297

71.7406 300

71.7497 295

71.7588 311

71.7679 291

71.7770 286

71.7861 285

71.7951 270

71.8042 278

71.8133 298

71.8224 275

71.8315 299

71.8406 275

71.8497 285

71.8587 313

71.8678 339

71.8769 304

71.8860 318

71.8951 306

71.9042 290

71.9133 284

71.9223 268

71.9314 314

71.9405 314

71.9496 320

71.9587 323

71.9678 328

71.9768 290

71.9859 291

71.9950 274

72.0041 308

72.0132 330

72.0223 291

72.0314 305

72.0404 286

72.0495 301

72.0586 297

72.0677 313

72.0768 321

72.0859 296

72.0949 290

72.1040 330

72.1131 313

72.1222 330

72.1313 304

72.1404 294

72.1495 280

72.1585 309

72.1676 279

72.1767 325

72.1858 265

72.1949 286

72.2040 293

72.2131 298

72.2221 306

72.2312 316

72.2403 303

72.2494 302

72.2585 299

72.2676 289

72.2766 296

72.2857 273

72.2948 311

72.3039 288

72.3130 304

72.3221 289

72.3312 284

72.3402 309

72.3493 291

72.3584 299

72.3675 309

72.3766 330

72.3857 314

72.3947 263

72.4038 267

72.4129 264

72.4220 298

72.4311 324

72.4402 287

72.4493 310

72.4583 294

72.4674 298

72.4765 303

72.4856 298

72.4947 309

72.5038 318

72.5129 270

72.5219 319

72.5310 284

72.5401 270

72.5492 281

72.5583 321

72.5674 285

72.5764 317

72.5855 279

72.5946 275

72.6037 286

72.6128 291

72.6219 284

72.6310 299

72.6400 284

72.6491 296

72.6582 286

72.6673 293

72.6764 292

72.6855 282

72.6945 301

72.7036 305

72.7127 302

72.7218 278

72.7309 290

72.7400 272

72.7491 282

72.7581 281

72.7672 300

72.7763 283

72.7854 297

72.7945 273

72.8036 306

72.8127 317

72.8217 274

72.8308 278

72.8399 279

72.8490 281

72.8581 274

72.8672 294

72.8762 274

72.8853 278

72.8944 290

72.9035 293

72.9126 282

72.9217 296

72.9308 280

72.9398 306

72.9489 304

72.9580 318

72.9671 313

72.9762 315

72.9853 279

72.9943 293

73.0034 280

73.0125 300

73.0216 306

73.0307 306

73.0398 325

73.0489 305

73.0579 294

73.0670 296

73.0761 299

73.0852 293

73.0943 269

73.1034 271

73.1125 304

73.1215 278

73.1306 320

73.1397 291

73.1488 294

73.1579 305

73.1670 303

73.1760 296

73.1851 301

73.1942 305

73.2033 299

73.2124 271

73.2215 286

73.2306 286

73.2396 300

73.2487 282

73.2578 331

73.2669 293

73.2760 305

73.2851 312

73.2941 319

73.3032 287

73.3123 310

73.3214 302

73.3305 285

73.3396 280

73.3487 280

73.3577 275

73.3668 287

73.3759 293

73.3850 276

73.3941 305

73.4032 284

73.4123 304

73.4213 306

73.4304 298

73.4395 293

73.4486 305

73.4577 299

73.4668 286

73.4758 293

73.4849 290

73.4940 288

73.5031 339

73.5122 293

73.5213 309

73.5304 300

73.5394 309

73.5485 279

73.5576 261

73.5667 272

73.5758 273

73.5849 281

73.5940 286

73.6030 273

73.6121 287

73.6212 281

73.6303 291

73.6394 278

73.6485 301

73.6575 291

73.6666 275

73.6757 299

73.6848 289

73.6939 301

73.7030 283

73.7121 284

73.7211 285

73.7302 294

73.7393 267

73.7484 324

73.7575 278

73.7666 275

73.7756 273

73.7847 288

73.7938 272

73.8029 295

73.8120 291

73.8211 291

73.8302 316

73.8392 342

73.8483 309

73.8574 295

73.8665 279

73.8756 294

73.8847 297

73.8938 300

73.9028 318

73.9119 282

73.9210 313

73.9301 274

73.9392 281

73.9483 295

73.9573 272

73.9664 275

73.9755 310

73.9846 280

73.9937 271

74.0028 297

74.0119 302

74.0209 258

74.0300 287

74.0391 301

74.0482 286

74.0573 298

74.0664 317

74.0754 279

74.0845 291

74.0936 269

74.1027 274

74.1118 295

74.1209 269

74.1300 292

74.1390 282

74.1481 280

74.1572 317

74.1663 271

74.1754 295

74.1845 291

74.1936 280

74.2026 256

74.2117 292

74.2208 304

74.2299 263

74.2390 308

74.2481 290

74.2571 301

74.2662 280

74.2753 288

74.2844 338

74.2935 273

74.3026 303

74.3117 300

74.3207 281

74.3298 299

74.3389 309

74.3480 266

74.3571 311

74.3662 323

74.3752 304

74.3843 283

74.3934 289

74.4025 275

74.4116 313

74.4207 295

74.4298 281

74.4388 279

74.4479 289

74.4570 299

74.4661 314

74.4752 279

74.4843 282

74.4934 304

74.5024 294

74.5115 254

74.5206 281

74.5297 290

74.5388 294

74.5479 265

74.5569 279

74.5660 289

74.5751 277

74.5842 280

74.5933 311

74.6024 267

74.6115 302

74.6205 298

74.6296 247

74.6387 296

74.6478 295

74.6569 293

74.6660 275

74.6750 300

74.6841 297

74.6932 268

74.7023 299

74.7114 302

74.7205 294

74.7296 265

74.7386 280

74.7477 327

74.7568 264

74.7659 296

74.7750 286

74.7841 301

74.7932 309

74.8022 290

74.8113 270

74.8204 309

74.8295 271

74.8386 282

74.8477 330

74.8567 293

74.8658 262

74.8749 281

74.8840 287

74.8931 303

74.9022 282

74.9113 297

74.9203 276

74.9294 283

74.9385 306

74.9476 302

74.9567 263

74.9658 273

74.9748 304

74.9839 295

74.9930 312

75.0021 267

75.0112 273

75.0203 303

75.0294 296

75.0384 292

75.0475 293

75.0566 294

75.0657 319

75.0748 303

75.0839 280

75.0930 298

75.1020 296

75.1111 284

75.1202 272

75.1293 280

75.1384 291

75.1475 269

75.1565 282

75.1656 243

75.1747 274

75.1838 304

75.1929 275

75.2020 298

75.2111 300

75.2201 293

75.2292 316

75.2383 300

75.2474 269

75.2565 267

75.2656 307

75.2746 266

75.2837 285

75.2928 281

75.3019 268

75.3110 308

75.3201 278

75.3292 265

75.3382 288

75.3473 281

75.3564 312

75.3655 266

75.3746 299

75.3837 298

75.3928 313

75.4018 297

75.4109 304

75.4200 277

75.4291 287

75.4382 269

75.4473 267

75.4563 290

75.4654 292

75.4745 278

75.4836 289

75.4927 294

75.5018 265

75.5109 285

75.5199 295

75.5290 294

75.5381 292

75.5472 314

75.5563 275

75.5654 265

75.5744 282

75.5835 308

75.5926 312

75.6017 280

75.6108 298

75.6199 281

75.6290 268

75.6380 294

75.6471 322

75.6562 283

75.6653 287

75.6744 285

75.6835 267

75.6926 300

75.7016 293

75.7107 312

75.7198 295

75.7289 301

75.7380 282

75.7471 303

75.7561 286

75.7652 307

75.7743 290

75.7834 279

75.7925 297

75.8016 300

75.8107 280

75.8197 295

75.8288 287

75.8379 287

75.8470 266

75.8561 304

75.8652 274

75.8742 264

75.8833 288

75.8924 268

75.9015 281

75.9106 276

75.9197 258

75.9288 300

75.9378 284

75.9469 268

75.9560 282

75.9651 288

75.9742 291

75.9833 291

75.9924 280

76.0014 298

76.0105 277

76.0196 314

76.0287 301

76.0378 276

76.0469 264

76.0559 259

76.0650 293

76.0741 290

76.0832 296

76.0923 343

76.1014 305

76.1105 287

76.1195 285

76.1286 229

76.1377 285

76.1468 287

76.1559 304

76.1650 296

76.1740 268

76.1831 297

76.1922 288

76.2013 281

76.2104 276

76.2195 259

76.2286 292

76.2376 254

76.2467 258

76.2558 287

76.2649 278

76.2740 270

76.2831 278

76.2922 307

76.3012 283

76.3103 276

76.3194 302

76.3285 287

76.3376 248

76.3467 315

76.3557 289

76.3648 312

76.3739 301

76.3830 279

76.3921 300

76.4012 271

76.4103 255

76.4193 309

76.4284 292

76.4375 293

76.4466 277

76.4557 287

76.4648 277

76.4738 276

76.4829 274

76.4920 256

76.5011 302

76.5102 270

76.5193 298

76.5284 309

76.5374 275

76.5465 306

76.5556 318

76.5647 307

76.5738 268

76.5829 281

76.5920 296

76.6010 272

76.6101 245

76.6192 275

76.6283 307

76.6374 260

76.6465 288

76.6555 308

76.6646 281

76.6737 266

76.6828 316

76.6919 292

76.7010 278

76.7101 262

76.7191 288

76.7282 306

76.7373 297

76.7464 300

76.7555 304

76.7646 270

76.7736 323

76.7827 283

76.7918 266

76.8009 280

76.8100 282

76.8191 274

76.8282 270

76.8372 257

76.8463 285

76.8554 292

76.8645 290

76.8736 281

76.8827 280

76.8918 291

76.9008 296

76.9099 266

76.9190 284

76.9281 279

76.9372 286

76.9463 277

76.9553 282

76.9644 273

76.9735 272

76.9826 289

76.9917 278

77.0008 282

77.0099 282

77.0189 289

77.0280 289

77.0371 290

77.0462 275

77.0553 278

77.0644 272

77.0734 310

77.0825 265

77.0916 314

77.1007 304

77.1098 285

77.1189 305

77.1280 279

77.1370 257

77.1461 291

77.1552 281

77.1643 247

77.1734 275

77.1825 272

77.1916 282

77.2006 291

77.2097 286

77.2188 263

77.2279 265

77.2370 284

77.2461 280

77.2551 299

77.2642 299

77.2733 276

77.2824 255

77.2915 281

77.3006 310

77.3097 286

77.3187 290

77.3278 280

77.3369 303

77.3460 301

77.3551 278

77.3642 276

77.3732 265

77.3823 290

77.3914 290

77.4005 277

77.4096 319

77.4187 258

77.4278 292

77.4368 273

77.4459 284

77.4550 283

77.4641 257

77.4732 274

77.4823 249

77.4914 261

77.5004 308

77.5095 278

77.5186 271

77.5277 241

77.5368 290

77.5459 283

77.5549 312

77.5640 270

77.5731 286

77.5822 305

77.5913 295

77.6004 274

77.6095 304

77.6185 325

77.6276 235

77.6367 287

77.6458 283

77.6549 293

77.6640 269

77.6730 311

77.6821 279

77.6912 295

77.7003 295

77.7094 286

77.7185 288

77.7276 276

77.7366 284

77.7457 271

77.7548 288

77.7639 278

77.7730 281

77.7821 282

77.7912 297

77.8002 259

77.8093 298

77.8184 292

77.8275 293

77.8366 302

77.8457 287

77.8547 252

77.8638 267

77.8729 296

77.8820 243

77.8911 279

77.9002 281

77.9093 295

77.9183 316

77.9274 272

77.9365 295

77.9456 295

77.9547 307

77.9638 255

77.9728 277

77.9819 297

77.9910 283

78.0001 282

78.0092 274

78.0183 275

78.0274 254

78.0364 277

78.0455 284

78.0546 284

78.0637 262

78.0728 261

78.0819 273

78.0910 275

78.1000 307

78.1091 262

78.1182 309

78.1273 297

78.1364 295

78.1455 317

78.1545 292

78.1636 289

78.1727 312

78.1818 289

78.1909 280

78.2000 289

78.2091 253

78.2181 252

78.2272 283

78.2363 260

78.2454 285

78.2545 260

78.2636 284

78.2726 287

78.2817 271

78.2908 288

78.2999 253

78.3090 246

78.3181 274

78.3272 279

78.3362 294

78.3453 288

78.3544 281

78.3635 278

78.3726 270

78.3817 273

78.3908 304

78.3998 298

78.4089 271

78.4180 274

78.4271 267

78.4362 286

78.4453 272

78.4543 262

78.4634 271

78.4725 265

78.4816 270

78.4907 309

78.4998 302

78.5089 300

78.5179 331

78.5270 269

78.5361 255

78.5452 278

78.5543 295

78.5634 313

78.5724 255

78.5815 262

78.5906 268

78.5997 256

78.6088 286

78.6179 263

78.6270 269

78.6360 272

78.6451 262

78.6542 273

78.6633 247

78.6724 290

78.6815 272

78.6906 298

78.6996 291

78.7087 296

78.7178 245

78.7269 279

78.7360 299

78.7451 274

78.7541 296

78.7632 282

78.7723 265

78.7814 249

78.7905 290

78.7996 279

78.8087 262

78.8177 270

78.8268 299

78.8359 263

78.8450 269

78.8541 296

78.8632 272

78.8722 307

78.8813 258

78.8904 281

78.8995 265

78.9086 283

78.9177 271

78.9268 267

78.9358 284

78.9449 265

78.9540 318

78.9631 281

78.9722 272

78.9813 285

78.9904 263

78.9994 271

79.0085 298

79.0176 281

79.0267 304

79.0358 274

79.0449 272

79.0539 276

79.0630 285

79.0721 280

79.0812 282

79.0903 285

79.0994 298

79.1085 275

79.1175 257

79.1266 296

79.1357 257

79.1448 265

79.1539 279

79.1630 275

79.1720 274

79.1811 287

79.1902 285

79.1993 266

79.2084 271

79.2175 267

79.2266 247

79.2356 267

79.2447 253

79.2538 294

79.2629 254

79.2720 256

79.2811 267

79.2902 267

79.2992 284

79.3083 266

79.3174 304

79.3265 270

79.3356 286

79.3447 270

79.3537 291

79.3628 284

79.3719 281

79.3810 275

79.3901 262

79.3992 288

79.4083 240

79.4173 267

79.4264 289

79.4355 293

79.4446 262

79.4537 278

79.4628 272

79.4718 279

79.4809 290

79.4900 265

79.4991 276

79.5082 289

79.5173 282

79.5264 258

79.5354 268

79.5445 287

79.5536 251

79.5627 293

79.5718 248

79.5809 258

79.5900 268

79.5990 288

79.6081 278

79.6172 251

79.6263 276

79.6354 288

79.6445 283

79.6535 312

79.6626 271

79.6717 260

79.6808 286

79.6899 273

79.6990 286

79.7081 274

79.7171 269

79.7262 298

79.7353 262

79.7444 292

79.7535 262

79.7626 275

79.7716 262

79.7807 278

79.7898 272

79.7989 286

79.8080 308

79.8171 279

79.8262 261

79.8352 265

79.8443 254

79.8534 237

79.8625 297

79.8716 293

79.8807 297

79.8898 314

79.8988 274

79.9079 308

79.9170 266

79.9261 275

79.9352 264

79.9443 293

79.9533 273

79.9624 246

79.9715 279

79.9806 282

79.9897 278

79.9988 288

80.0079 238

80.0169 255

80.0260 286

80.0351 294

80.0442 301

80.0533 257

80.0624 255

80.0714 292

80.0805 273

80.0896 275

80.0987 261

80.1078 296

80.1169 266

80.1260 290

80.1350 274

80.1441 276

80.1532 265

80.1623 262

80.1714 257

80.1805 288

80.1896 294

80.1986 307

80.2077 294

80.2168 281

80.2259 271

80.2350 262

80.2441 235

80.2531 290

80.2622 277

80.2713 295

80.2804 294

80.2895 246

80.2986 256

80.3077 248

80.3167 264

80.3258 263

80.3349 273

80.3440 293

80.3531 288

80.3622 277

80.3712 300

80.3803 273

80.3894 283

80.3985 255

80.4076 277

80.4167 295

80.4258 303

80.4348 269

80.4439 263

80.4530 282

80.4621 281

80.4712 278

80.4803 263

80.4894 278

80.4984 276

80.5075 268

80.5166 264

80.5257 256

80.5348 242

80.5439 294

80.5529 254

80.5620 266

80.5711 253

80.5802 296

80.5893 242

80.5984 273

80.6075 277

80.6165 284

80.6256 274

80.6347 262

80.6438 273

80.6529 282

80.6620 268

80.6710 290

80.6801 264

80.6892 289

80.6983 255

80.7074 277

80.7165 274

80.7256 285

80.7346 250

80.7437 274

80.7528 316

80.7619 280

80.7710 282

80.7801 279

80.7892 301

80.7982 256

80.8073 240

80.8164 283

80.8255 296

80.8346 259

80.8437 293

80.8527 284

80.8618 243

80.8709 259

80.8800 282

80.8891 261

80.8982 260

80.9073 273

80.9163 294

80.9254 290

80.9345 288

80.9436 293

80.9527 287

80.9618 287

80.9708 247

80.9799 261

80.9890 227

80.9981 273

81.0072 302

81.0163 258

81.0254 285

81.0344 288

81.0435 271

81.0526 268

81.0617 265

81.0708 290

81.0799 236

81.0890 262

81.0980 278

81.1071 290

81.1162 286

81.1253 269

81.1344 245

81.1435 287

81.1525 271

81.1616 277

81.1707 299

81.1798 278

81.1889 259

81.1980 291

81.2071 280

81.2161 299

81.2252 266

81.2343 269

81.2434 273

81.2525 301

81.2616 246

81.2706 268

81.2797 243

81.2888 293

81.2979 263

81.3070 260

81.3161 280

81.3252 272

81.3342 249

81.3433 283

81.3524 296

81.3615 273

81.3706 272

81.3797 262

81.3888 236

81.3978 269

81.4069 287

81.4160 254

81.4251 265

81.4342 278

81.4433 279

81.4523 241

81.4614 256

81.4705 255

81.4796 277

81.4887 275

81.4978 286

81.5069 284

81.5159 280

81.5250 257

81.5341 261

81.5432 250

81.5523 270

81.5614 253

81.5704 270

81.5795 258

81.5886 234

81.5977 267

81.6068 240

81.6159 281

81.6250 290

81.6340 271

81.6431 261

81.6522 270

81.6613 312

81.6704 248

81.6795 266

81.6886 266

81.6976 258

81.7067 234

81.7158 237

81.7249 254

81.7340 290

81.7431 278

81.7521 259

81.7612 266

81.7703 262

81.7794 257

81.7885 265

81.7976 242

81.8067 260

81.8157 259

81.8248 252

81.8339 283

81.8430 261

81.8521 264

81.8612 265

81.8702 265

81.8793 267

81.8884 271

81.8975 273

81.9066 281

81.9157 271

81.9248 275

81.9338 283

81.9429 261

81.9520 271

81.9611 286

81.9702 269

81.9793 255

81.9884 265

81.9974 268

82.0065 265

82.0156 258

82.0247 265

82.0338 285

82.0429 263

82.0519 297

82.0610 268

82.0701 242

82.0792 282

82.0883 248

82.0974 244

82.1065 239

82.1155 274

82.1246 261

82.1337 236

82.1428 298

82.1519 275

82.1610 263

82.1700 262

82.1791 235

82.1882 255

82.1973 303

82.2064 261

82.2155 265

82.2246 311

82.2336 270

82.2427 261

82.2518 278

82.2609 238

82.2700 270

82.2791 282

82.2882 267

82.2972 271

82.3063 264

82.3154 266

82.3245 260

82.3336 263

82.3427 273

82.3517 253

82.3608 272

82.3699 282

82.3790 266

82.3881 281

82.3972 301

82.4063 273

82.4153 276

82.4244 257

82.4335 298

82.4426 319

82.4517 272

82.4608 257

82.4698 259

82.4789 264

82.4880 294

82.4971 261

82.5062 275

82.5153 264

82.5244 275

82.5334 284

82.5425 274

82.5516 291

82.5607 262

82.5698 261

82.5789 276

82.5880 282

82.5970 282

82.6061 269

82.6152 283

82.6243 293

82.6334 255

82.6425 262

82.6515 241

82.6606 284

82.6697 247

82.6788 271

82.6879 251

82.6970 285

82.7061 277

82.7151 273

82.7242 267

82.7333 279

82.7424 272

82.7515 303

82.7606 284

82.7696 218

82.7787 259

82.7878 267

82.7969 255

82.8060 274

82.8151 257

82.8242 278

82.8332 290

82.8423 272

82.8514 274

82.8605 254

82.8696 241

82.8787 254

82.8878 286

82.8968 268

82.9059 279

82.9150 265

82.9241 267

82.9332 286

82.9423 263

82.9513 280

82.9604 256

82.9695 252

82.9786 266

82.9877 261

82.9968 254

83.0059 262

83.0149 249

83.0240 256

83.0331 262

83.0422 275

83.0513 259

83.0604 275

83.0694 279

83.0785 255

83.0876 263

83.0967 242

83.1058 282

83.1149 268

83.1240 250

83.1330 267

83.1421 289

83.1512 249

83.1603 278

83.1694 298

83.1785 241

83.1876 278

83.1966 257

83.2057 276

83.2148 293

83.2239 263

83.2330 264

83.2421 270

83.2511 283

83.2602 271

83.2693 262

83.2784 284

83.2875 243

83.2966 240

83.3057 271

83.3147 250

83.3238 242

83.3329 263

83.3420 258

83.3511 277

83.3602 251

83.3692 280

83.3783 255

83.3874 255

83.3965 256

83.4056 270

83.4147 287

83.4238 246

83.4328 278

83.4419 289

83.4510 244

83.4601 283

83.4692 263

83.4783 245

83.4874 249

83.4964 280

83.5055 284

83.5146 263

83.5237 265

83.5328 262

83.5419 285

83.5509 283

83.5600 258

83.5691 266

83.5782 253

83.5873 270

83.5964 266

83.6055 293

83.6145 261

83.6236 281

83.6327 265

83.6418 286

83.6509 272

83.6600 242

83.6690 263

83.6781 261

83.6872 256

83.6963 272

83.7054 301

83.7145 285

83.7236 262

83.7326 272

83.7417 246

83.7508 260

83.7599 255

83.7690 300

83.7781 234

83.7872 290

83.7962 290

83.8053 271

83.8144 274

83.8235 262

83.8326 276

83.8417 280

83.8507 256

83.8598 251

83.8689 281

83.8780 265

83.8871 294

83.8962 248

83.9053 272

83.9143 239

83.9234 244

83.9325 262

83.9416 238

83.9507 262

83.9598 258

83.9688 270

83.9779 287

83.9870 261

83.9961 262

84.0052 234

84.0143 249

84.0234 277

84.0324 252

84.0415 261

84.0506 244

84.0597 252

84.0688 275

84.0779 258

84.0870 262

84.0960 272

84.1051 269

84.1142 267

84.1233 275

84.1324 266

84.1415 283

84.1505 236

84.1596 266

84.1687 277

84.1778 259

84.1869 263

84.1960 267

84.2051 258

84.2141 263

84.2232 274

84.2323 260

84.2414 297

84.2505 260

84.2596 259

84.2686 266

84.2777 267

84.2868 255

84.2959 272

84.3050 243

84.3141 218

84.3232 287

84.3322 259

84.3413 257

84.3504 269

84.3595 260

84.3686 283

84.3777 277

84.3868 268

84.3958 260

84.4049 266

84.4140 282

84.4231 250

84.4322 268

84.4413 266

84.4503 275

84.4594 229

84.4685 244

84.4776 267

84.4867 265

84.4958 289

84.5049 264

84.5139 306

84.5230 277

84.5321 260

84.5412 267

84.5503 225

84.5594 284

84.5684 285

84.5775 231

84.5866 266

84.5957 268

84.6048 271

84.6139 283

84.6230 251

84.6320 264

84.6411 244

84.6502 266

84.6593 255

84.6684 288

84.6775 277

84.6866 229

84.6956 255

84.7047 278

84.7138 270

84.7229 277

84.7320 281

84.7411 277

84.7501 268

84.7592 262

84.7683 275

84.7774 278

84.7865 258

84.7956 249

84.8047 265

84.8137 263

84.8228 262

84.8319 260

84.8410 292

84.8501 286

84.8592 269

84.8682 271

84.8773 246

84.8864 255

84.8955 247

84.9046 262

84.9137 283

84.9228 256

84.9318 273

84.9409 264

84.9500 293

84.9591 247

84.9682 242

84.9773 255

84.9864 268

84.9954 232

85.0045 248

85.0136 275

85.0227 277

85.0318 274

85.0409 257

85.0499 264

85.0590 247

85.0681 250

85.0772 258

85.0863 279

85.0954 252

85.1045 273

85.1135 276

85.1226 280

85.1317 244

85.1408 274

85.1499 284

85.1590 274

85.1680 259

85.1771 244

85.1862 269

85.1953 270

85.2044 261

85.2135 244

85.2226 269

85.2316 241

85.2407 263

85.2498 274

85.2589 285

85.2680 230

85.2771 282

85.2862 250

85.2952 281

85.3043 242

85.3134 248

85.3225 239

85.3316 257

85.3407 243

85.3497 279

85.3588 240

85.3679 246

85.3770 238

85.3861 260

85.3952 255

85.4043 248

85.4133 275

85.4224 279

85.4315 285

85.4406 272

85.4497 314

85.4588 253

85.4678 270

85.4769 263

85.4860 238

85.4951 224

85.5042 292

85.5133 246

85.5224 287

85.5314 256

85.5405 288

85.5496 280

85.5587 282

85.5678 277

85.5769 265

85.5860 244

85.5950 267

85.6041 270

85.6132 274

85.6223 234

85.6314 230

85.6405 243

85.6495 279

85.6586 221

85.6677 271

85.6768 283

85.6859 243

85.6950 252

85.7041 254

85.7131 232

85.7222 215

85.7313 275

85.7404 269

85.7495 248

85.7586 269

85.7676 275

85.7767 300

85.7858 252

85.7949 281

85.8040 265

85.8131 234

85.8222 261

85.8312 260

85.8403 255

85.8494 250

85.8585 230

85.8676 249

85.8767 295

85.8858 255

85.8948 265

85.9039 260

85.9130 233

85.9221 243

85.9312 260

85.9403 272

85.9493 276

85.9584 263

85.9675 278

85.9766 252

85.9857 258

85.9948 265

86.0039 226

86.0129 254

86.0220 277

86.0311 272

86.0402 255

86.0493 269

86.0584 230

86.0674 235

86.0765 239

86.0856 271

86.0947 232

86.1038 245

86.1129 292

86.1220 252

86.1310 273

86.1401 252

86.1492 281

86.1583 256

86.1674 267

86.1765 268

86.1856 267

86.1946 271

86.2037 249

86.2128 288

86.2219 293

86.2310 266

86.2401 275

86.2491 271

86.2582 244

86.2673 270

86.2764 256

86.2855 272

86.2946 245

86.3037 258

86.3127 253

86.3218 263

86.3309 243

86.3400 294

86.3491 252

86.3582 264

86.3672 279

86.3763 260

86.3854 263

86.3945 269

86.4036 271

86.4127 259

86.4218 271

86.4308 271

86.4399 248

86.4490 286

86.4581 240

86.4672 257

86.4763 234

86.4854 230

86.4944 280

86.5035 252

86.5126 257

86.5217 258

86.5308 257

86.5399 254

86.5489 243

86.5580 247

86.5671 264

86.5762 265

86.5853 274

86.5944 262

86.6035 259

86.6125 271

86.6216 273

86.6307 241

86.6398 255

86.6489 266

86.6580 266

86.6670 237

86.6761 249

86.6852 250

86.6943 248

86.7034 288

86.7125 270

86.7216 223

86.7306 238

86.7397 267

86.7488 243

86.7579 262

86.7670 256

86.7761 271

86.7852 257

86.7942 260

86.8033 245

86.8124 240

86.8215 242

86.8306 243

86.8397 241

86.8487 273

86.8578 248

86.8669 262

86.8760 257

86.8851 248

86.8942 267

86.9033 286

86.9123 258

86.9214 251

86.9305 258

86.9396 239

86.9487 249

86.9578 277

86.9668 265

86.9759 244

86.9850 274

86.9941 297

87.0032 251

87.0123 249

87.0214 263

87.0304 284

87.0395 248

87.0486 224

87.0577 282

87.0668 242

87.0759 248

87.0850 248

87.0940 247

87.1031 251

87.1122 232

87.1213 266

87.1304 277

87.1395 270

87.1485 271

87.1576 242

87.1667 296

87.1758 249

87.1849 287

87.1940 260

87.2031 271

87.2121 269

87.2212 285

87.2303 264

87.2394 271

87.2485 253

87.2576 224

87.2666 248

87.2757 233

87.2848 254

87.2939 242

87.3030 256

87.3121 245

87.3212 267

87.3302 250

87.3393 247

87.3484 261

87.3575 248

87.3666 252

87.3757 267

87.3848 253

87.3938 272

87.4029 236

87.4120 256

87.4211 254

87.4302 238

87.4393 242

87.4483 239

87.4574 238

87.4665 257

87.4756 235

87.4847 243

87.4938 255

87.5029 269

87.5119 257

87.5210 244

87.5301 259

87.5392 258

87.5483 256

87.5574 273

87.5664 226

87.5755 242

87.5846 245

87.5937 268

87.6028 258

87.6119 246

87.6210 270

87.6300 270

87.6391 222

87.6482 245

87.6573 268

87.6664 224

87.6755 237

87.6846 290

87.6936 239

87.7027 224

87.7118 286

87.7209 266

87.7300 250

87.7391 262

87.7481 295

87.7572 257

87.7663 238

87.7754 280

87.7845 286

87.7936 260

87.8027 253

87.8117 247

87.8208 263

87.8299 292

87.8390 284

87.8481 260

87.8572 276

87.8662 260

87.8753 265

87.8844 282

87.8935 257

87.9026 254

87.9117 262

87.9208 278

87.9298 264

87.9389 265

87.9480 255

87.9571 271

87.9662 258

87.9753 249

87.9844 248

87.9934 251

88.0025 267

88.0116 242

88.0207 258

88.0298 256

88.0389 254

88.0479 266

88.0570 249

88.0661 241

88.0752 260

88.0843 259

88.0934 269

88.1025 238

88.1115 262

88.1206 268

88.1297 238

88.1388 245

88.1479 239

88.1570 252

88.1660 245

88.1751 220

88.1842 248

88.1933 276

88.2024 245

88.2115 276

88.2206 245

88.2296 246

88.2387 236

88.2478 254

88.2569 239

88.2660 247

88.2751 264

88.2842 261

88.2932 238

88.3023 268

88.3114 247

88.3205 239

88.3296 260

88.3387 267

88.3477 268

88.3568 270

88.3659 226

88.3750 235

88.3841 233

88.3932 250

88.4023 239

88.4113 250

88.4204 262

88.4295 220

88.4386 257

88.4477 280

88.4568 257

88.4658 283

88.4749 261

88.4840 258

88.4931 232

88.5022 253

88.5113 249

88.5204 243

88.5294 215

88.5385 250

88.5476 257

88.5567 275

88.5658 210

88.5749 252

88.5840 238

88.5930 283

88.6021 264

88.6112 266

88.6203 262

88.6294 251

88.6385 277

88.6475 236

88.6566 247

88.6657 261

88.6748 278

88.6839 257

88.6930 253

88.7021 272

88.7111 252

88.7202 284

88.7293 246

88.7384 241

88.7475 272

88.7566 265

88.7656 241

88.7747 234

88.7838 274

88.7929 248

88.8020 227

88.8111 250

88.8202 261

88.8292 247

88.8383 250

88.8474 271

88.8565 265

88.8656 273

88.8747 259

88.8838 238

88.8928 254

88.9019 279

88.9110 267

88.9201 231

88.9292 252

88.9383 222

88.9473 227

88.9564 228

88.9655 268

88.9746 259

88.9837 249

88.9928 259

89.0019 255

89.0109 251

89.0200 251

89.0291 240

89.0382 282

89.0473 255

89.0564 256

89.0654 271

89.0745 243

89.0836 276

89.0927 223

89.1018 257

89.1109 247

89.1200 265

89.1290 258

89.1381 224

89.1472 262

89.1563 244

89.1654 236

89.1745 241

89.1836 246

89.1926 242

89.2017 226

89.2108 247

89.2199 257

89.2290 256

89.2381 209

89.2471 254

89.2562 214

89.2653 273

89.2744 247

89.2835 266

89.2926 252

89.3017 248

89.3107 250

89.3198 250

89.3289 252

89.3380 237

89.3471 258

89.3562 253

89.3652 260

89.3743 254

89.3834 251

89.3925 248

89.4016 262

89.4107 238

89.4198 267

89.4288 253

89.4379 252

89.4470 253

89.4561 234

89.4652 253

89.4743 246

89.4834 259

89.4924 259

89.5015 256

89.5106 246

89.5197 237

89.5288 237

89.5379 242

89.5469 262

89.5560 225

89.5651 283

89.5742 258

89.5833 214

89.5924 250

89.6015 245

89.6105 262

89.6196 249

89.6287 250

89.6378 258

89.6469 262

89.6560 239

89.6650 247

89.6741 259

89.6832 237

89.6923 245

89.7014 248

89.7105 265

89.7196 227

89.7286 247

89.7377 237

89.7468 262

89.7559 247

89.7650 261

89.7741 263

89.7832 251

89.7922 234

89.8013 269

89.8104 241

89.8195 259

89.8286 284

89.8377 240

89.8467 260

89.8558 254

89.8649 249

89.8740 241

89.8831 241

89.8922 244

89.9013 246

89.9103 239

89.9194 258

89.9285 268

89.9376 231

89.9467 236

89.9558 252

89.9648 249

89.9739 238

89.9830 241

89.9921 234

90.0012 218

90.0103 240

90.0194 235

90.0284 218

90.0375 264

90.0466 271

90.0557 239

90.0648 248

90.0739 254

90.0830 251

90.0920 237

90.1011 255

90.1102 245

90.1193 239

90.1284 246

90.1375 272

90.1465 251

90.1556 235

90.1647 257

90.1738 233

90.1829 274

90.1920 234

90.2011 235

90.2101 236

90.2192 217

90.2283 264

90.2374 264

90.2465 238

90.2556 268

90.2646 256

90.2737 229

90.2828 250

90.2919 269

90.3010 239

90.3101 265

90.3192 249

90.3282 257

90.3373 248

90.3464 281

90.3555 274

90.3646 257

90.3737 233

90.3828 263

90.3918 242

90.4009 240

90.4100 256

90.4191 246

90.4282 242

90.4373 277

90.4463 233

90.4554 227

90.4645 228

90.4736 267

90.4827 284

90.4918 256

90.5009 259

90.5099 237

90.5190 266

90.5281 253

90.5372 250

**Raw data 2**. XRD raw data of microsphere obtained spray pyrolysis at 550 ℃.

; (content of file C:\DATA\CBNU_IN\ENG-Chemical\JoJungSang\Oh Sehwan\190104\Ni-Co_550C.raw)

_FILEVERSION = 2

_SAMPLE =

_+SAMPLE =

_SITE = Korea

_USER = "Chungbuk UNI."

_GONIOMETER_CODE = 21

; Goniometer : D8 theta/theta, stage : Unknown

_SAMPLE_CHANGER_CODE = 0

_ATTACHMENTS_CODE = 0

_GONIOMETER_RADIUS = 250

_FIXED_DIVSLIT = 0.6

_FIXED_SAMPLESLIT = 0

_FIXED_DETSLIT = 12.21

_MONOCHROMATOR = 0

; Incident beam monochromator : None

_THIN_FILM = N

_BETA_FILTER = N

_FIXED_ANTISLIT = 8.46

_ANALYZER_CODE = 4

; Received beam analyzer : Gobel mirror

_DATEMEASURED = "04-Jan-2019 15:04:41"

_WL_UNIT = A

_WL1 = 1.5406

_WL2 = 1.54439

_WL3 = 1.39222

_WLRATIO = 0.5

_ANODE = Cu

; Data for range 1

_DRIVE = COUPLED

_STEPTIME = 35.8

_STEPSIZE = 0.00908485

_STEPMODE = C

_START = 10

_THETA = 5

_2THETA = 10

_KHI = 0

_PHI = 0

_X = 0

_Y = 0

_Z = 0

_DETECTOR = 5

; Detector type : Unknown

_DETECTORSLIT = out

_AUX1 = 0

_AUX2 = 0

_AUX3 = 0

_TIMESTARTED = 12

_TEMP_RATE = -1

_TEMP_DELAY = -1

_KV = 40

_MA = 40

_RANGE_WL = 1.5406

_3DPLANE = 0

_V4_COUNTERS_MASK = 4096

_V4_DRIVES_MASK = 0

_V4_ENCODERS_MASK = 0

_2THETACOUNTS = 1

; 2THETA PSD

10.0000 1159

10.0091 1105

10.0182 1148

10.0273 1150

10.0363 1163

10.0454 1179

10.0545 1167

10.0636 1118

10.0727 1190

10.0818 1171

10.0908 1211

10.0999 1132

10.1090 1136

10.1181 1106

10.1272 1163

10.1363 1185

10.1454 1152

10.1544 1193

10.1635 1136

10.1726 1120

10.1817 1164

10.1908 1208

10.1999 1163

10.2090 1155

10.2180 1181

10.2271 1167

10.2362 1137

10.2453 1193

10.2544 1203

10.2635 1151

10.2725 1160

10.2816 1185

10.2907 1217

10.2998 1145

10.3089 1148

10.3180 1133

10.3271 1166

10.3361 1118

10.3452 1189

10.3543 1100

10.3634 1114

10.3725 1162

10.3816 1179

10.3906 1117

10.3997 1121

10.4088 1119

10.4179 1134

10.4270 1152

10.4361 1074

10.4452 1122

10.4542 1214

10.4633 1165

10.4724 1147

10.4815 1095

10.4906 1199

10.4997 1184

10.5088 1156

10.5178 1113

10.5269 1121

10.5360 1219

10.5451 1145

10.5542 1162

10.5633 1154

10.5723 1183

10.5814 1159

10.5905 1161

10.5996 1151

10.6087 1146

10.6178 1142

10.6269 1185

10.6359 1112

10.6450 1160

10.6541 1124

10.6632 1199

10.6723 1109

10.6814 1105

10.6904 1160

10.6995 1134

10.7086 1158

10.7177 1133

10.7268 1077

10.7359 1136

10.7450 1133

10.7540 1132

10.7631 1123

10.7722 1094

10.7813 1187

10.7904 1091

10.7995 1184

10.8086 1149

10.8176 1143

10.8267 1140

10.8358 1154

10.8449 1136

10.8540 1113

10.8631 1123

10.8721 1139

10.8812 1098

10.8903 1089

10.8994 1137

10.9085 1160

10.9176 1091

10.9267 1161

10.9357 1123

10.9448 1114

10.9539 1144

10.9630 988

10.9721 1142

10.9812 1178

10.9902 1083

10.9993 1166

11.0084 1130

11.0175 1118

11.0266 1117

11.0357 1129

11.0448 1137

11.0538 1101

11.0629 1097

11.0720 1090

11.0811 1119

11.0902 1106

11.0993 1105

11.1084 1146

11.1174 1177

11.1265 1126

11.1356 1070

11.1447 1157

11.1538 1127

11.1629 1148

11.1719 1094

11.1810 1062

11.1901 1144

11.1992 1032

11.2083 1075

11.2174 1084

11.2265 1130

11.2355 1155

11.2446 1167

11.2537 1121

11.2628 1160

11.2719 1091

11.2810 1130

11.2900 1082

11.2991 1109

11.3082 1087

11.3173 1097

11.3264 1123

11.3355 1097

11.3446 1138

11.3536 1109

11.3627 1144

11.3718 1015

11.3809 1171

11.3900 1034

11.3991 1137

11.4082 1139

11.4172 1128

11.4263 1125

11.4354 1068

11.4445 1097

11.4536 1049

11.4627 1070

11.4717 1048

11.4808 1098

11.4899 1016

11.4990 1149

11.5081 1093

11.5172 1102

11.5263 1088

11.5353 1104

11.5444 1041

11.5535 1135

11.5626 1110

11.5717 1045

11.5808 1145

11.5898 1057

11.5989 1096

11.6080 1113

11.6171 1078

11.6262 1071

11.6353 1069

11.6444 1170

11.6534 1025

11.6625 1064

11.6716 1059

11.6807 1113

11.6898 1086

11.6989 1098

11.7080 1078

11.7170 1113

11.7261 1171

11.7352 1037

11.7443 1065

11.7534 1097

11.7625 1080

11.7715 1126

11.7806 1044

11.7897 1133

11.7988 1088

11.8079 1145

11.8170 1068

11.8261 1107

11.8351 1113

11.8442 1109

11.8533 1095

11.8624 1108

11.8715 1066

11.8806 1025

11.8896 1118

11.8987 1088

11.9078 1055

11.9169 1105

11.9260 1081

11.9351 1070

11.9442 1111

11.9532 1079

11.9623 1022

11.9714 1077

11.9805 1076

11.9896 1135

11.9987 1089

12.0078 1084

12.0168 1073

12.0259 1068

12.0350 1070

12.0441 1089

12.0532 1104

12.0623 1116

12.0713 1119

12.0804 1061

12.0895 1067

12.0986 1097

12.1077 1014

12.1168 1066

12.1259 1143

12.1349 1034

12.1440 1054

12.1531 1134

12.1622 1016

12.1713 1127

12.1804 1120

12.1894 1121

12.1985 1112

12.2076 1012

12.2167 1017

12.2258 1034

12.2349 1036

12.2440 1119

12.2530 1075

12.2621 1081

12.2712 1067

12.2803 1058

12.2894 1076

12.2985 1073

12.3076 1086

12.3166 1092

12.3257 1068

12.3348 1063

12.3439 1061

12.3530 1060

12.3621 1090

12.3711 1038

12.3802 1097

12.3893 1115

12.3984 1062

12.4075 1115

12.4166 1029

12.4257 1074

12.4347 1035

12.4438 1055

12.4529 1024

12.4620 1069

12.4711 1025

12.4802 1073

12.4892 1022

12.4983 1021

12.5074 1065

12.5165 1058

12.5256 1037

12.5347 1050

12.5438 1091

12.5528 1069

12.5619 1062

12.5710 1062

12.5801 1058

12.5892 1075

12.5983 1078

12.6074 1054

12.6164 1073

12.6255 1029

12.6346 1087

12.6437 1020

12.6528 1079

12.6619 1028

12.6709 1043

12.6800 1119

12.6891 1064

12.6982 1111

12.7073 1057

12.7164 1115

12.7255 1001

12.7345 1074

12.7436 1043

12.7527 1046

12.7618 1011

12.7709 1092

12.7800 1071

12.7890 1064

12.7981 1118

12.8072 1031

12.8163 1068

12.8254 1109

12.8345 1061

12.8436 1089

12.8526 1089

12.8617 1057

12.8708 1033

12.8799 1041

12.8890 1061

12.8981 1010

12.9072 1076

12.9162 1028

12.9253 1006

12.9344 1076

12.9435 1079

12.9526 1008

12.9617 1037

12.9707 1096

12.9798 990

12.9889 1046

12.9980 1020

13.0071 1094

13.0162 993

13.0253 1001

13.0343 1082

13.0434 973

13.0525 1068

13.0616 1049

13.0707 1045

13.0798 1068

13.0888 1037

13.0979 1100

13.1070 1103

13.1161 1065

13.1252 1060

13.1343 1008

13.1434 1069

13.1524 1067

13.1615 1023

13.1706 1131

13.1797 1012

13.1888 1050

13.1979 1052

13.2070 1017

13.2160 1024

13.2251 1036

13.2342 1050

13.2433 1039

13.2524 999

13.2615 1034

13.2705 1018

13.2796 1033

13.2887 1058

13.2978 1027

13.3069 1032

13.3160 1082

13.3251 1074

13.3341 1053

13.3432 1005

13.3523 1069

13.3614 1072

13.3705 1055

13.3796 1092

13.3886 1049

13.3977 1055

13.4068 1004

13.4159 1006

13.4250 1032

13.4341 1075

13.4432 1043

13.4522 1032

13.4613 1067

13.4704 1076

13.4795 1106

13.4886 1052

13.4977 1048

13.5068 1038

13.5158 1013

13.5249 1101

13.5340 982

13.5431 1064

13.5522 994

13.5613 1038

13.5703 980

13.5794 1082

13.5885 1044

13.5976 1002

13.6067 1016

13.6158 1038

13.6249 1071

13.6339 1048

13.6430 1071

13.6521 1061

13.6612 1014

13.6703 1067

13.6794 1058

13.6884 1034

13.6975 1004

13.7066 1035

13.7157 1008

13.7248 1011

13.7339 1098

13.7430 1084

13.7520 1100

13.7611 1017

13.7702 1066

13.7793 974

13.7884 1067

13.7975 990

13.8066 960

13.8156 1022

13.8247 1051

13.8338 1049

13.8429 1018

13.8520 1057

13.8611 1043

13.8701 1051

13.8792 978

13.8883 1065

13.8974 1072

13.9065 993

13.9156 1038

13.9247 1039

13.9337 1051

13.9428 1035

13.9519 1093

13.9610 949

13.9701 1082

13.9792 1067

13.9882 1057

13.9973 1035

14.0064 1028

14.0155 1017

14.0246 1071

14.0337 1074

14.0428 1040

14.0518 1028

14.0609 1056

14.0700 1010

14.0791 924

14.0882 1128

14.0973 1040

14.1064 1055

14.1154 1043

14.1245 997

14.1336 1050

14.1427 1056

14.1518 1008

14.1609 1105

14.1699 1056

14.1790 1004

14.1881 1033

14.1972 1028

14.2063 1036

14.2154 1054

14.2245 1056

14.2335 1015

14.2426 1069

14.2517 1040

14.2608 1058

14.2699 1000

14.2790 1030

14.2880 1049

14.2971 1056

14.3062 1040

14.3153 1020

14.3244 1048

14.3335 1054

14.3426 1008

14.3516 992

14.3607 994

14.3698 1026

14.3789 1071

14.3880 1058

14.3971 1017

14.4062 1075

14.4152 1016

14.4243 1014

14.4334 1067

14.4425 1015

14.4516 1049

14.4607 1005

14.4697 1059

14.4788 1000

14.4879 976

14.4970 1002

14.5061 1042

14.5152 1080

14.5243 1050

14.5333 1043

14.5424 1019

14.5515 1025

14.5606 1066

14.5697 1095

14.5788 1013

14.5878 987

14.5969 1054

14.6060 1016

14.6151 1064

14.6242 1057

14.6333 1050

14.6424 1057

14.6514 1075

14.6605 1079

14.6696 1016

14.6787 1041

14.6878 965

14.6969 1031

14.7060 1070

14.7150 1013

14.7241 973

14.7332 1046

14.7423 1068

14.7514 1051

14.7605 1040

14.7695 1019

14.7786 1019

14.7877 1012

14.7968 1019

14.8059 1065

14.8150 1069

14.8241 1018

14.8331 1007

14.8422 1045

14.8513 1056

14.8604 1090

14.8695 1042

14.8786 1019

14.8876 994

14.8967 1065

14.9058 1063

14.9149 1046

14.9240 1076

14.9331 1026

14.9422 1018

14.9512 1000

14.9603 1000

14.9694 1023

14.9785 1061

14.9876 1037

14.9967 1044

15.0058 1046

15.0148 1032

15.0239 1026

15.0330 973

15.0421 1052

15.0512 1039

15.0603 1062

15.0693 1074

15.0784 1060

15.0875 1042

15.0966 1011

15.1057 1101

15.1148 1076

15.1239 1006

15.1329 1010

15.1420 1029

15.1511 951

15.1602 1055

15.1693 1069

15.1784 1062

15.1874 985

15.1965 1073

15.2056 1046

15.2147 997

15.2238 1054

15.2329 1034

15.2420 1001

15.2510 1042

15.2601 1032

15.2692 987

15.2783 1008

15.2874 1106

15.2965 1009

15.3056 1049

15.3146 1017

15.3237 987

15.3328 1039

15.3419 1047

15.3510 1048

15.3601 996

15.3691 980

15.3782 1039

15.3873 1071

15.3964 1109

15.4055 1018

15.4146 1040

15.4237 1053

15.4327 998

15.4418 1095

15.4509 1046

15.4600 991

15.4691 996

15.4782 1082

15.4872 1034

15.4963 966

15.5054 975

15.5145 1022

15.5236 1114

15.5327 1032

15.5418 986

15.5508 1013

15.5599 1050

15.5690 1003

15.5781 1044

15.5872 1056

15.5963 999

15.6054 1030

15.6144 1039

15.6235 1074

15.6326 1026

15.6417 1031

15.6508 1015

15.6599 1077

15.6689 1019

15.6780 1036

15.6871 997

15.6962 990

15.7053 1037

15.7144 1051

15.7235 1027

15.7325 1070

15.7416 1014

15.7507 983

15.7598 1006

15.7689 1034

15.7780 965

15.7870 1034

15.7961 1047

15.8052 1064

15.8143 952

15.8234 1077

15.8325 971

15.8416 1012

15.8506 1030

15.8597 988

15.8688 1100

15.8779 1028

15.8870 1027

15.8961 1036

15.9052 1099

15.9142 1043

15.9233 1030

15.9324 1039

15.9415 1031

15.9506 1003

15.9597 1051

15.9687 1033

15.9778 1039

15.9869 1025

15.9960 1038

16.0051 1031

16.0142 1019

16.0233 1009

16.0323 1034

16.0414 1015

16.0505 1022

16.0596 985

16.0687 978

16.0778 1045

16.0868 1007

16.0959 1024

16.1050 982

16.1141 1040

16.1232 1008

16.1323 1013

16.1414 1062

16.1504 1028

16.1595 1028

16.1686 1031

16.1777 1070

16.1868 1044

16.1959 1024

16.2050 1073

16.2140 1042

16.2231 999

16.2322 1064

16.2413 1040

16.2504 1039

16.2595 969

16.2685 998

16.2776 1009

16.2867 1003

16.2958 1033

16.3049 997

16.3140 1043

16.3231 1008

16.3321 992

16.3412 1054

16.3503 975

16.3594 988

16.3685 974

16.3776 1001

16.3866 1030

16.3957 1002

16.4048 1031

16.4139 999

16.4230 1078

16.4321 969

16.4412 1002

16.4502 1019

16.4593 1025

16.4684 1048

16.4775 1015

16.4866 1078

16.4957 1052

16.5048 1038

16.5138 1040

16.5229 1040

16.5320 1017

16.5411 1025

16.5502 1035

16.5593 1069

16.5683 1022

16.5774 1063

16.5865 1030

16.5956 1016

16.6047 1025

16.6138 974

16.6229 1032

16.6319 1003

16.6410 1077

16.6501 1023

16.6592 1030

16.6683 1067

16.6774 1086

16.6864 1007

16.6955 1058

16.7046 1006

16.7137 997

16.7228 1030

16.7319 967

16.7410 1033

16.7500 996

16.7591 1021

16.7682 1058

16.7773 1047

16.7864 1069

16.7955 1042

16.8046 1051

16.8136 1017

16.8227 1059

16.8318 1017

16.8409 1036

16.8500 1054

16.8591 999

16.8681 1017

16.8772 1026

16.8863 1066

16.8954 1041

16.9045 965

16.9136 1048

16.9227 990

16.9317 999

16.9408 1030

16.9499 992

16.9590 967

16.9681 939

16.9772 1053

16.9862 1045

16.9953 1015

17.0044 993

17.0135 1067

17.0226 981

17.0317 1020

17.0408 1045

17.0498 1021

17.0589 990

17.0680 1052

17.0771 998

17.0862 1014

17.0953 1058

17.1044 1109

17.1134 1033

17.1225 996

17.1316 1026

17.1407 1064

17.1498 952

17.1589 1008

17.1679 1019

17.1770 1003

17.1861 1039

17.1952 986

17.2043 1023

17.2134 1038

17.2225 981

17.2315 1057

17.2406 952

17.2497 988

17.2588 1017

17.2679 1057

17.2770 955

17.2860 1045

17.2951 967

17.3042 1005

17.3133 1000

17.3224 1007

17.3315 1031

17.3406 1046

17.3496 1010

17.3587 996

17.3678 1021

17.3769 1037

17.3860 1006

17.3951 1035

17.4042 1028

17.4132 1003

17.4223 978

17.4314 986

17.4405 968

17.4496 1049

17.4587 1027

17.4677 1020

17.4768 1031

17.4859 1067

17.4950 999

17.5041 1027

17.5132 1046

17.5223 1077

17.5313 1057

17.5404 1025

17.5495 1032

17.5586 957

17.5677 986

17.5768 979

17.5858 1049

17.5949 1024

17.6040 1032

17.6131 1023

17.6222 966

17.6313 954

17.6404 1075

17.6494 1050

17.6585 1051

17.6676 993

17.6767 1010

17.6858 1015

17.6949 1059

17.7040 1076

17.7130 1081

17.7221 1040

17.7312 1009

17.7403 1047

17.7494 1038

17.7585 1068

17.7675 1007

17.7766 1014

17.7857 1023

17.7948 1052

17.8039 989

17.8130 1025

17.8221 1021

17.8311 1041

17.8402 1037

17.8493 987

17.8584 1020

17.8675 959

17.8766 1046

17.8856 1038

17.8947 1046

17.9038 963

17.9129 960

17.9220 998

17.9311 974

17.9402 1009

17.9492 994

17.9583 1014

17.9674 1023

17.9765 1073

17.9856 1019

17.9947 967

18.0038 1037

18.0128 1032

18.0219 1008

18.0310 998

18.0401 1001

18.0492 1018

18.0583 1013

18.0673 987

18.0764 1030

18.0855 999

18.0946 962

18.1037 985

18.1128 1034

18.1219 992

18.1309 1023

18.1400 992

18.1491 998

18.1582 1024

18.1673 1059

18.1764 1027

18.1854 941

18.1945 1074

18.2036 981

18.2127 1002

18.2218 954

18.2309 1022

18.2400 1021

18.2490 1037

18.2581 1026

18.2672 1048

18.2763 1004

18.2854 1055

18.2945 1056

18.3036 1015

18.3126 987

18.3217 994

18.3308 1061

18.3399 1047

18.3490 1042

18.3581 1054

18.3671 1019

18.3762 1070

18.3853 1017

18.3944 1025

18.4035 1028

18.4126 997

18.4217 995

18.4307 1054

18.4398 1016

18.4489 1040

18.4580 972

18.4671 994

18.4762 1031

18.4852 1057

18.4943 1040

18.5034 1021

18.5125 1027

18.5216 1012

18.5307 993

18.5398 1003

18.5488 1027

18.5579 1028

18.5670 1053

18.5761 1010

18.5852 1029

18.5943 1025

18.6034 1039

18.6124 1024

18.6215 982

18.6306 1024

18.6397 991

18.6488 1011

18.6579 1022

18.6669 916

18.6760 1013

18.6851 1078

18.6942 1081

18.7033 996

18.7124 1016

18.7215 1055

18.7305 953

18.7396 947

18.7487 1026

18.7578 1027

18.7669 1020

18.7760 1004

18.7850 999

18.7941 1048

18.8032 1026

18.8123 1018

18.8214 1040

18.8305 1063

18.8396 978

18.8486 1032

18.8577 965

18.8668 979

18.8759 1008

18.8850 961

18.8941 992

18.9032 987

18.9122 1014

18.9213 1012

18.9304 942

18.9395 981

18.9486 1001

18.9577 1073

18.9667 999

18.9758 1022

18.9849 1015

18.9940 1074

19.0031 979

19.0122 989

19.0213 962

19.0303 956

19.0394 964

19.0485 968

19.0576 977

19.0667 1040

19.0758 952

19.0848 1030

19.0939 1006

19.1030 1029

19.1121 1027

19.1212 1015

19.1303 1055

19.1394 972

19.1484 1026

19.1575 972

19.1666 1005

19.1757 1004

19.1848 1014

19.1939 951

19.2030 1047

19.2120 1012

19.2211 966

19.2302 1032

19.2393 996

19.2484 954

19.2575 1064

19.2665 980

19.2756 1037

19.2847 1031

19.2938 1026

19.3029 984

19.3120 989

19.3211 1085

19.3301 1029

19.3392 989

19.3483 1047

19.3574 1054

19.3665 1013

19.3756 1013

19.3847 996

19.3937 1039

19.4028 993

19.4119 989

19.4210 1046

19.4301 975

19.4392 1017

19.4482 926

19.4573 1039

19.4664 1039

19.4755 963

19.4846 1016

19.4937 969

19.5028 985

19.5118 1038

19.5209 949

19.5300 1050

19.5391 961

19.5482 1053

19.5573 1022

19.5663 1023

19.5754 1028

19.5845 1044

19.5936 1002

19.6027 982

19.6118 936

19.6209 1005

19.6299 1037

19.6390 1025

19.6481 988

19.6572 991

19.6663 967

19.6754 1031

19.6845 994

19.6935 1050

19.7026 1001

19.7117 1003

19.7208 1003

19.7299 1022

19.7390 975

19.7480 999

19.7571 1021

19.7662 969

19.7753 979

19.7844 1023

19.7935 966

19.8026 994

19.8116 990

19.8207 1016

19.8298 1003

19.8389 1001

19.8480 953

19.8571 925

19.8661 959

19.8752 978

19.8843 1032

19.8934 991

19.9025 1056

19.9116 983

19.9207 1020

19.9297 961

19.9388 969

19.9479 1086

19.9570 1057

19.9661 972

19.9752 1008

19.9843 1014

19.9933 938

20.0024 997

20.0115 981

20.0206 991

20.0297 1025

20.0388 980

20.0478 988

20.0569 966

20.0660 946

20.0751 973

20.0842 1014

20.0933 988

20.1024 973

20.1114 976

20.1205 938

20.1296 961

20.1387 1034

20.1478 957

20.1569 998

20.1659 1015

20.1750 1002

20.1841 997

20.1932 976

20.2023 974

20.2114 984

20.2205 914

20.2295 982

20.2386 983

20.2477 1013

20.2568 1042

20.2659 977

20.2750 1004

20.2841 1010

20.2931 992

20.3022 985

20.3113 1012

20.3204 996

20.3295 975

20.3386 1009

20.3476 1013

20.3567 940

20.3658 1002

20.3749 1061

20.3840 979

20.3931 1028

20.4022 970

20.4112 997

20.4203 1014

20.4294 965

20.4385 1006

20.4476 985

20.4567 968

20.4657 979

20.4748 959

20.4839 970

20.4930 968

20.5021 975

20.5112 994

20.5203 968

20.5293 999

20.5384 980

20.5475 1057

20.5566 1029

20.5657 969

20.5748 990

20.5839 1020

20.5929 995

20.6020 988

20.6111 964

20.6202 1067

20.6293 946

20.6384 948

20.6474 1049

20.6565 1023

20.6656 960

20.6747 947

20.6838 937

20.6929 996

20.7020 1028

20.7110 989

20.7201 955

20.7292 970

20.7383 980

20.7474 967

20.7565 979

20.7655 975

20.7746 999

20.7837 988

20.7928 1022

20.8019 1007

20.8110 998

20.8201 978

20.8291 1002

20.8382 1014

20.8473 994

20.8564 999

20.8655 957

20.8746 994

20.8837 995

20.8927 976

20.9018 992

20.9109 958

20.9200 958

20.9291 953

20.9382 946

20.9472 1041

20.9563 950

20.9654 937

20.9745 961

20.9836 978

20.9927 1030

21.0018 955

21.0108 1022

21.0199 973

21.0290 962

21.0381 1032

21.0472 921

21.0563 975

21.0653 991

21.0744 1016

21.0835 1000

21.0926 979

21.1017 1003

21.1108 1045

21.1199 978

21.1289 1014

21.1380 987

21.1471 971

21.1562 979

21.1653 952

21.1744 977

21.1835 966

21.1925 1005

21.2016 946

21.2107 1006

21.2198 926

21.2289 1016

21.2380 964

21.2470 984

21.2561 991

21.2652 1018

21.2743 985

21.2834 940

21.2925 924

21.3016 900

21.3106 959

21.3197 954

21.3288 931

21.3379 979

21.3470 963

21.3561 948

21.3651 1027

21.3742 1007

21.3833 869

21.3924 941

21.4015 988

21.4106 974

21.4197 922

21.4287 976

21.4378 957

21.4469 957

21.4560 927

21.4651 960

21.4742 927

21.4833 943

21.4923 972

21.5014 980

21.5105 1025

21.5196 891

21.5287 979

21.5378 986

21.5468 998

21.5559 960

21.5650 977

21.5741 1001

21.5832 981

21.5923 991

21.6014 898

21.6104 947

21.6195 946

21.6286 941

21.6377 949

21.6468 997

21.6559 979

21.6649 969

21.6740 993

21.6831 993

21.6922 941

21.7013 978

21.7104 991

21.7195 933

21.7285 918

21.7376 944

21.7467 980

21.7558 995

21.7649 986

21.7740 961

21.7831 961

21.7921 1014

21.8012 947

21.8103 962

21.8194 1008

21.8285 980

21.8376 940

21.8466 939

21.8557 960

21.8648 917

21.8739 917

21.8830 959

21.8921 925

21.9012 954

21.9102 981

21.9193 1005

21.9284 954

21.9375 1001

21.9466 970

21.9557 1003

21.9647 996

21.9738 988

21.9829 993

21.9920 912

22.0011 927

22.0102 979

22.0193 983

22.0283 929

22.0374 961

22.0465 944

22.0556 937

22.0647 936

22.0738 976

22.0829 933

22.0919 999

22.1010 957

22.1101 936

22.1192 943

22.1283 989

22.1374 925

22.1464 989

22.1555 1009

22.1646 948

22.1737 901

22.1828 1033

22.1919 924

22.2010 1006

22.2100 929

22.2191 976

22.2282 1024

22.2373 968

22.2464 1003

22.2555 949

22.2645 992

22.2736 964

22.2827 961

22.2918 931

22.3009 920

22.3100 933

22.3191 934

22.3281 946

22.3372 951

22.3463 964

22.3554 924

22.3645 1001

22.3736 986

22.3827 1023

22.3917 971

22.4008 960

22.4099 994

22.4190 976

22.4281 986

22.4372 1024

22.4462 969

22.4553 938

22.4644 860

22.4735 950

22.4826 993

22.4917 997

22.5008 989

22.5098 968

22.5189 895

22.5280 972

22.5371 919

22.5462 993

22.5553 972

22.5643 991

22.5734 872

22.5825 961

22.5916 938

22.6007 930

22.6098 968

22.6189 956

22.6279 944

22.6370 927

22.6461 943

22.6552 970

22.6643 945

22.6734 955

22.6825 1013

22.6915 932

22.7006 964

22.7097 955

22.7188 1004

22.7279 1021

22.7370 932

22.7460 936

22.7551 956

22.7642 935

22.7733 919

22.7824 964

22.7915 987

22.8006 959

22.8096 922

22.8187 934

22.8278 975

22.8369 964

22.8460 1008

22.8551 927

22.8641 873

22.8732 904

22.8823 972

22.8914 920

22.9005 936

22.9096 942

22.9187 931

22.9277 954

22.9368 900

22.9459 1000

22.9550 934

22.9641 949

22.9732 987

22.9823 987

22.9913 926

23.0004 963

23.0095 920

23.0186 965

23.0277 930

23.0368 964

23.0458 962

23.0549 937

23.0640 914

23.0731 959

23.0822 959

23.0913 951

23.1004 1027

23.1094 932

23.1185 903

23.1276 919

23.1367 934

23.1458 952

23.1549 954

23.1639 889

23.1730 941

23.1821 964

23.1912 946

23.2003 971

23.2094 928

23.2185 895

23.2275 927

23.2366 914

23.2457 891

23.2548 960

23.2639 958

23.2730 958

23.2821 969

23.2911 1019

23.3002 927

23.3093 935

23.3184 931

23.3275 940

23.3366 994

23.3456 972

23.3547 943

23.3638 977

23.3729 936

23.3820 946

23.3911 964

23.4002 941

23.4092 944

23.4183 890

23.4274 981

23.4365 935

23.4456 939

23.4547 956

23.4637 941

23.4728 901

23.4819 939

23.4910 985

23.5001 998

23.5092 922

23.5183 945

23.5273 918

23.5364 919

23.5455 928

23.5546 957

23.5637 911

23.5728 895

23.5819 935

23.5909 956

23.6000 973

23.6091 940

23.6182 935

23.6273 966

23.6364 1026

23.6454 927

23.6545 952

23.6636 951

23.6727 963

23.6818 891

23.6909 947

23.7000 911

23.7090 966

23.7181 888

23.7272 895

23.7363 905

23.7454 957

23.7545 931

23.7635 963

23.7726 935

23.7817 892

23.7908 913

23.7999 927

23.8090 922

23.8181 942

23.8271 942

23.8362 943

23.8453 941

23.8544 897

23.8635 966

23.8726 960

23.8817 938

23.8907 948

23.8998 953

23.9089 964

23.9180 892

23.9271 885

23.9362 949

23.9452 933

23.9543 998

23.9634 950

23.9725 918

23.9816 925

23.9907 879

23.9998 933

24.0088 891

24.0179 906

24.0270 939

24.0361 958

24.0452 891

24.0543 932

24.0633 911

24.0724 894

24.0815 888

24.0906 884

24.0997 939

24.1088 900

24.1179 892

24.1269 951

24.1360 894

24.1451 892

24.1542 971

24.1633 934

24.1724 903

24.1815 951

24.1905 954

24.1996 901

24.2087 901

24.2178 910

24.2269 859

24.2360 960

24.2450 890

24.2541 918

24.2632 968

24.2723 897

24.2814 977

24.2905 908

24.2996 929

24.3086 905

24.3177 971

24.3268 947

24.3359 908

24.3450 931

24.3541 926

24.3631 910

24.3722 945

24.3813 885

24.3904 926

24.3995 911

24.4086 937

24.4177 930

24.4267 965

24.4358 914

24.4449 940

24.4540 960

24.4631 887

24.4722 931

24.4813 907

24.4903 953

24.4994 910

24.5085 938

24.5176 908

24.5267 920

24.5358 929

24.5448 878

24.5539 906

24.5630 891

24.5721 903

24.5812 941

24.5903 927

24.5994 920

24.6084 936

24.6175 910

24.6266 935

24.6357 901

24.6448 903

24.6539 886

24.6629 956

24.6720 919

24.6811 939

24.6902 892

24.6993 920

24.7084 928

24.7175 908

24.7265 873

24.7356 884

24.7447 941

24.7538 955

24.7629 855

24.7720 890

24.7811 930

24.7901 890

24.7992 911

24.8083 937

24.8174 954

24.8265 900

24.8356 923

24.8446 930

24.8537 926

24.8628 897

24.8719 894

24.8810 966

24.8901 956

24.8992 908

24.9082 957

24.9173 848

24.9264 908

24.9355 892

24.9446 967

24.9537 923

24.9627 873

24.9718 916

24.9809 892

24.9900 936

24.9991 911

25.0082 926

25.0173 974

25.0263 889

25.0354 877

25.0445 880

25.0536 919

25.0627 933

25.0718 922

25.0809 936

25.0899 903

25.0990 881

25.1081 945

25.1172 891

25.1263 966

25.1354 892

25.1444 965

25.1535 908

25.1626 916

25.1717 885

25.1808 943

25.1899 884

25.1990 880

25.2080 936

25.2171 895

25.2262 849

25.2353 934

25.2444 941

25.2535 932

25.2625 876

25.2716 882

25.2807 860

25.2898 869

25.2989 903

25.3080 918

25.3171 912

25.3261 929

25.3352 884

25.3443 860

25.3534 878

25.3625 903

25.3716 895

25.3807 954

25.3897 934

25.3988 930

25.4079 870

25.4170 964

25.4261 926

25.4352 876

25.4442 987

25.4533 955

25.4624 859

25.4715 916

25.4806 967

25.4897 884

25.4988 929

25.5078 949

25.5169 861

25.5260 875

25.5351 885

25.5442 898

25.5533 837

25.5623 918

25.5714 857

25.5805 912

25.5896 883

25.5987 921

25.6078 913

25.6169 884

25.6259 836

25.6350 912

25.6441 955

25.6532 852

25.6623 926

25.6714 912

25.6805 857

25.6895 896

25.6986 890

25.7077 941

25.7168 916

25.7259 875

25.7350 902

25.7440 886

25.7531 891

25.7622 864

25.7713 904

25.7804 927

25.7895 874

25.7986 923

25.8076 886

25.8167 898

25.8258 907

25.8349 902

25.8440 868

25.8531 900

25.8621 939

25.8712 915

25.8803 873

25.8894 916

25.8985 897

25.9076 907

25.9167 887

25.9257 893

25.9348 841

25.9439 934

25.9530 876

25.9621 891

25.9712 924

25.9803 845

25.9893 894

25.9984 836

26.0075 888

26.0166 898

26.0257 874

26.0348 930

26.0438 869

26.0529 928

26.0620 890

26.0711 924

26.0802 890

26.0893 886

26.0984 918

26.1074 881

26.1165 888

26.1256 899

26.1347 914

26.1438 850

26.1529 938

26.1619 945

26.1710 887

26.1801 909

26.1892 910

26.1983 864

26.2074 906

26.2165 899

26.2255 892

26.2346 860

26.2437 867

26.2528 850

26.2619 867

26.2710 828

26.2801 856

26.2891 823

26.2982 831

26.3073 863

26.3164 873

26.3255 857

26.3346 877

26.3436 893

26.3527 839

26.3618 847

26.3709 853

26.3800 863

26.3891 923

26.3982 885

26.4072 921

26.4163 896

26.4254 857

26.4345 833

26.4436 897

26.4527 945

26.4617 952

26.4708 870

26.4799 891

26.4890 892

26.4981 878

26.5072 885

26.5163 858

26.5253 861

26.5344 855

26.5435 872

26.5526 902

26.5617 900

26.5708 850

26.5799 891

26.5889 915

26.5980 888

26.6071 875

26.6162 918

26.6253 898

26.6344 913

26.6434 897

26.6525 878

26.6616 856

26.6707 867

26.6798 935

26.6889 899

26.6980 856

26.7070 914

26.7161 835

26.7252 920

26.7343 896

26.7434 855

26.7525 884

26.7615 914

26.7706 889

26.7797 904

26.7888 877

26.7979 853

26.8070 895

26.8161 908

26.8251 904

26.8342 868

26.8433 889

26.8524 849

26.8615 928

26.8706 881

26.8797 869

26.8887 901

26.8978 905

26.9069 871

26.9160 849

26.9251 910

26.9342 903

26.9432 896

26.9523 855

26.9614 886

26.9705 865

26.9796 877

26.9887 896

26.9978 899

27.0068 864

27.0159 865

27.0250 900

27.0341 904

27.0432 799

27.0523 918

27.0613 873

27.0704 862

27.0795 858

27.0886 908

27.0977 873

27.1068 893

27.1159 832

27.1249 929

27.1340 856

27.1431 907

27.1522 869

27.1613 844

27.1704 838

27.1795 856

27.1885 888

27.1976 895

27.2067 910

27.2158 795

27.2249 903

27.2340 916

27.2430 832

27.2521 860

27.2612 839

27.2703 824

27.2794 805

27.2885 865

27.2976 833

27.3066 859

27.3157 885

27.3248 839

27.3339 847

27.3430 789

27.3521 835

27.3611 926

27.3702 903

27.3793 860

27.3884 908

27.3975 833

27.4066 875

27.4157 841

27.4247 872

27.4338 907

27.4429 877

27.4520 854

27.4611 822

27.4702 854

27.4793 872

27.4883 876

27.4974 846

27.5065 811

27.5156 895

27.5247 873

27.5338 880

27.5428 879

27.5519 858

27.5610 830

27.5701 857

27.5792 834

27.5883 847

27.5974 862

27.6064 819

27.6155 841

27.6246 894

27.6337 852

27.6428 850

27.6519 893

27.6609 914

27.6700 843

27.6791 834

27.6882 910

27.6973 858

27.7064 855

27.7155 834

27.7245 870

27.7336 833

27.7427 838

27.7518 911

27.7609 862

27.7700 892

27.7791 821

27.7881 859

27.7972 847

27.8063 885

27.8154 842

27.8245 825

27.8336 835

27.8426 915

27.8517 869

27.8608 879

27.8699 843

27.8790 848

27.8881 884

27.8972 841

27.9062 825

27.9153 848

27.9244 836

27.9335 867

27.9426 846

27.9517 850

27.9607 839

27.9698 878

27.9789 867

27.9880 892

27.9971 871

28.0062 905

28.0153 834

28.0243 836

28.0334 879

28.0425 828

28.0516 863

28.0607 825

28.0698 889

28.0789 874

28.0879 892

28.0970 865

28.1061 866

28.1152 828

28.1243 888

28.1334 838

28.1424 860

28.1515 911

28.1606 852

28.1697 908

28.1788 856

28.1879 867

28.1970 873

28.2060 897

28.2151 866

28.2242 853

28.2333 839

28.2424 901

28.2515 846

28.2605 859

28.2696 812

28.2787 836

28.2878 794

28.2969 860

28.3060 836

28.3151 847

28.3241 863

28.3332 864

28.3423 844

28.3514 865

28.3605 823

28.3696 881

28.3787 876

28.3877 904

28.3968 853

28.4059 876

28.4150 829

28.4241 817

28.4332 868

28.4422 866

28.4513 907

28.4604 938

28.4695 851

28.4786 875

28.4877 878

28.4968 861

28.5058 857

28.5149 885

28.5240 834

28.5331 857

28.5422 888

28.5513 892

28.5603 818

28.5694 877

28.5785 883

28.5876 920

28.5967 835

28.6058 849

28.6149 845

28.6239 819

28.6330 859

28.6421 858

28.6512 843

28.6603 886

28.6694 843

28.6785 867

28.6875 835

28.6966 896

28.7057 834

28.7148 873

28.7239 832

28.7330 838

28.7420 837

28.7511 872

28.7602 870

28.7693 830

28.7784 853

28.7875 909

28.7966 832

28.8056 831

28.8147 885

28.8238 857

28.8329 881

28.8420 842

28.8511 847

28.8601 869

28.8692 872

28.8783 848

28.8874 869

28.8965 855

28.9056 864

28.9147 851

28.9237 846

28.9328 917

28.9419 894

28.9510 874

28.9601 838

28.9692 800

28.9783 903

28.9873 862

28.9964 827

29.0055 871

29.0146 820

29.0237 879

29.0328 840

29.0418 834

29.0509 842

29.0600 857

29.0691 828

29.0782 832

29.0873 867

29.0964 879

29.1054 819

29.1145 860

29.1236 821

29.1327 855

29.1418 820

29.1509 900

29.1599 876

29.1690 872

29.1781 851

29.1872 803

29.1963 851

29.2054 847

29.2145 825

29.2235 831

29.2326 799

29.2417 841

29.2508 842

29.2599 794

29.2690 867

29.2781 863

29.2871 885

29.2962 867

29.3053 875

29.3144 877

29.3235 894

29.3326 849

29.3416 854

29.3507 804

29.3598 853

29.3689 844

29.3780 837

29.3871 842

29.3962 807

29.4052 839

29.4143 837

29.4234 817

29.4325 856

29.4416 807

29.4507 794

29.4597 839

29.4688 848

29.4779 843

29.4870 829

29.4961 835

29.5052 842

29.5143 853

29.5233 821

29.5324 819

29.5415 917

29.5506 808

29.5597 891

29.5688 825

29.5779 810

29.5869 879

29.5960 802

29.6051 838

29.6142 869

29.6233 788

29.6324 813

29.6414 825

29.6505 809

29.6596 886

29.6687 853

29.6778 851

29.6869 835

29.6960 806

29.7050 840

29.7141 812

29.7232 810

29.7323 802

29.7414 830

29.7505 894

29.7595 858

29.7686 807

29.7777 876

29.7868 855

29.7959 812

29.8050 820

29.8141 860

29.8231 850

29.8322 827

29.8413 848

29.8504 825

29.8595 893

29.8686 826

29.8777 835

29.8867 804

29.8958 818

29.9049 916

29.9140 844

29.9231 823

29.9322 845

29.9412 875

29.9503 838

29.9594 827

29.9685 828

29.9776 805

29.9867 853

29.9958 863

30.0048 875

30.0139 857

30.0230 861

30.0321 845

30.0412 788

30.0503 804

30.0593 797

30.0684 772

30.0775 786

30.0866 812

30.0957 849

30.1048 823

30.1139 818

30.1229 885

30.1320 850

30.1411 854

30.1502 802

30.1593 838

30.1684 823

30.1775 839

30.1865 819

30.1956 806

30.2047 798

30.2138 826

30.2229 816

30.2320 805

30.2410 860

30.2501 817

30.2592 799

30.2683 842

30.2774 825

30.2865 827

30.2956 843

30.3046 893

30.3137 828

30.3228 821

30.3319 813

30.3410 817

30.3501 837

30.3591 824

30.3682 803

30.3773 854

30.3864 806

30.3955 839

30.4046 826

30.4137 829

30.4227 846

30.4318 823

30.4409 841

30.4500 894

30.4591 877

30.4682 837

30.4773 847

30.4863 820

30.4954 841

30.5045 814

30.5136 866

30.5227 768

30.5318 816

30.5408 777

30.5499 855

30.5590 815

30.5681 808

30.5772 797

30.5863 841

30.5954 782

30.6044 821

30.6135 860

30.6226 840

30.6317 861

30.6408 839

30.6499 848

30.6589 861

30.6680 837

30.6771 790

30.6862 816

30.6953 829

30.7044 843

30.7135 782

30.7225 796

30.7316 844

30.7407 819

30.7498 849

30.7589 845

30.7680 828

30.7771 829

30.7861 837

30.7952 785

30.8043 844

30.8134 862

30.8225 749

30.8316 862

30.8406 808

30.8497 785

30.8588 825

30.8679 809

30.8770 821

30.8861 828

30.8952 810

30.9042 774

30.9133 845

30.9224 793

30.9315 848

30.9406 850

30.9497 824

30.9587 803

30.9678 888

30.9769 881

30.9860 771

30.9951 816

31.0042 803

31.0133 834

31.0223 844

31.0314 870

31.0405 815

31.0496 799

31.0587 797

31.0678 829

31.0769 785

31.0859 816

31.0950 841

31.1041 844

31.1132 825

31.1223 787

31.1314 808

31.1404 839

31.1495 821

31.1586 837

31.1677 812

31.1768 845

31.1859 823

31.1950 801

31.2040 828

31.2131 803

31.2222 822

31.2313 823

31.2404 780

31.2495 828

31.2585 779

31.2676 817

31.2767 809

31.2858 768

31.2949 828

31.3040 860

31.3131 898

31.3221 832

31.3312 818

31.3403 794

31.3494 853

31.3585 823

31.3676 801

31.3767 803

31.3857 819

31.3948 840

31.4039 837

31.4130 844

31.4221 811

31.4312 877

31.4402 849

31.4493 848

31.4584 897

31.4675 865

31.4766 847

31.4857 819

31.4948 818

31.5038 838

31.5129 861

31.5220 843

31.5311 828

31.5402 799

31.5493 775

31.5583 879

31.5674 861

31.5765 817

31.5856 822

31.5947 803

31.6038 855

31.6129 822

31.6219 843

31.6310 805

31.6401 787

31.6492 863

31.6583 847

31.6674 776

31.6765 851

31.6855 790

31.6946 819

31.7037 794

31.7128 787

31.7219 825

31.7310 794

31.7400 788

31.7491 808

31.7582 789

31.7673 824

31.7764 808

31.7855 788

31.7946 888

31.8036 815

31.8127 798

31.8218 781

31.8309 783

31.8400 766

31.8491 747

31.8581 856

31.8672 806

31.8763 848

31.8854 757

31.8945 825

31.9036 800

31.9127 806

31.9217 772

31.9308 794

31.9399 775

31.9490 851

31.9581 814

31.9672 802

31.9763 842

31.9853 765

31.9944 848

32.0035 852

32.0126 762

32.0217 842

32.0308 852

32.0398 831

32.0489 829

32.0580 800

32.0671 828

32.0762 813

32.0853 790

32.0944 849

32.1034 825

32.1125 768

32.1216 865

32.1307 798

32.1398 805

32.1489 806

32.1579 838

32.1670 777

32.1761 751

32.1852 824

32.1943 840

32.2034 800

32.2125 789

32.2215 848

32.2306 789

32.2397 762

32.2488 826

32.2579 784

32.2670 844

32.2761 781

32.2851 848

32.2942 792

32.3033 860

32.3124 800

32.3215 835

32.3306 773

32.3396 786

32.3487 847

32.3578 850

32.3669 800

32.3760 838

32.3851 819

32.3942 797

32.4032 814

32.4123 791

32.4214 770

32.4305 820

32.4396 767

32.4487 819

32.4577 838

32.4668 755

32.4759 855

32.4850 788

32.4941 775

32.5032 743

32.5123 826

32.5213 813

32.5304 804

32.5395 832

32.5486 789

32.5577 786

32.5668 818

32.5759 796

32.5849 842

32.5940 811

32.6031 873

32.6122 791

32.6213 826

32.6304 804

32.6394 777

32.6485 761

32.6576 786

32.6667 803

32.6758 866

32.6849 794

32.6940 764

32.7030 832

32.7121 814

32.7212 821

32.7303 795

32.7394 787

32.7485 778

32.7575 825

32.7666 789

32.7757 803

32.7848 801

32.7939 857

32.8030 791

32.8121 755

32.8211 798

32.8302 790

32.8393 780

32.8484 817

32.8575 781

32.8666 831

32.8757 736

32.8847 834

32.8938 841

32.9029 795

32.9120 789

32.9211 808

32.9302 812

32.9392 790

32.9483 793

32.9574 768

32.9665 837

32.9756 809

32.9847 818

32.9938 811

33.0028 749

33.0119 780

33.0210 771

33.0301 807

33.0392 898

33.0483 776

33.0573 842

33.0664 849

33.0755 822

33.0846 778

33.0937 842

33.1028 882

33.1119 827

33.1209 802

33.1300 811

33.1391 828

33.1482 788

33.1573 829

33.1664 831

33.1755 782

33.1845 789

33.1936 808

33.2027 796

33.2118 815

33.2209 755

33.2300 820

33.2390 799

33.2481 813

33.2572 792

33.2663 800

33.2754 764

33.2845 825

33.2936 842

33.3026 811

33.3117 777

33.3208 801

33.3299 816

33.3390 812

33.3481 786

33.3571 795

33.3662 804

33.3753 785

33.3844 810

33.3935 793

33.4026 773

33.4117 832

33.4207 805

33.4298 793

33.4389 771

33.4480 761

33.4571 808

33.4662 791

33.4753 786

33.4843 826

33.4934 787

33.5025 828

33.5116 797

33.5207 777

33.5298 815

33.5388 824

33.5479 781

33.5570 783

33.5661 830

33.5752 773

33.5843 817

33.5934 830

33.6024 819

33.6115 836

33.6206 805

33.6297 795

33.6388 824

33.6479 810

33.6569 816

33.6660 796

33.6751 842

33.6842 845

33.6933 802

33.7024 772

33.7115 819

33.7205 817

33.7296 789

33.7387 791

33.7478 795

33.7569 801

33.7660 779

33.7751 763

33.7841 799

33.7932 743

33.8023 748

33.8114 836

33.8205 806

33.8296 771

33.8386 777

33.8477 762

33.8568 783

33.8659 808

33.8750 790

33.8841 797

33.8932 781

33.9022 796

33.9113 779

33.9204 756

33.9295 795

33.9386 840

33.9477 812

33.9567 838

33.9658 846

33.9749 802

33.9840 762

33.9931 796

34.0022 793

34.0113 820

34.0203 801

34.0294 854

34.0385 839

34.0476 783

34.0567 797

34.0658 836

34.0749 767

34.0839 764

34.0930 758

34.1021 766

34.1112 791

34.1203 751

34.1294 820

34.1384 788

34.1475 759

34.1566 808

34.1657 755

34.1748 787

34.1839 812

34.1930 808

34.2020 858

34.2111 760

34.2202 797

34.2293 788

34.2384 811

34.2475 794

34.2565 785

34.2656 730

34.2747 865

34.2838 777

34.2929 788

34.3020 750

34.3111 770

34.3201 762

34.3292 820

34.3383 780

34.3474 812

34.3565 775

34.3656 805

34.3747 789

34.3837 788

34.3928 783

34.4019 816

34.4110 854

34.4201 779

34.4292 758

34.4382 796

34.4473 753

34.4564 776

34.4655 800

34.4746 795

34.4837 807

34.4928 820

34.5018 838

34.5109 807

34.5200 783

34.5291 779

34.5382 803

34.5473 801

34.5563 754

34.5654 831

34.5745 708

34.5836 791

34.5927 786

34.6018 792

34.6109 797

34.6199 750

34.6290 845

34.6381 825

34.6472 812

34.6563 752

34.6654 818

34.6745 785

34.6835 769

34.6926 770

34.7017 782

34.7108 774

34.7199 829

34.7290 770

34.7380 806

34.7471 785

34.7562 803

34.7653 834

34.7744 803

34.7835 837

34.7926 812

34.8016 780

34.8107 795

34.8198 757

34.8289 785

34.8380 768

34.8471 800

34.8561 790

34.8652 818

34.8743 801

34.8834 759

34.8925 796

34.9016 805

34.9107 788

34.9197 722

34.9288 770

34.9379 780

34.9470 862

34.9561 797

34.9652 825

34.9743 817

34.9833 756

34.9924 750

35.0015 779

35.0106 714

35.0197 774

35.0288 797

35.0378 746

35.0469 814

35.0560 848

35.0651 831

35.0742 774

35.0833 784

35.0924 806

35.1014 852

35.1105 795

35.1196 785

35.1287 784

35.1378 737

35.1469 836

35.1559 784

35.1650 825

35.1741 793

35.1832 781

35.1923 814

35.2014 825

35.2105 770

35.2195 841

35.2286 758

35.2377 747

35.2468 830

35.2559 812

35.2650 740

35.2741 782

35.2831 808

35.2922 774

35.3013 799

35.3104 730

35.3195 798

35.3286 819

35.3376 790

35.3467 815

35.3558 814

35.3649 781

35.3740 809

35.3831 838

35.3922 849

35.4012 736

35.4103 785

35.4194 842

35.4285 811

35.4376 788

35.4467 793

35.4557 800

35.4648 827

35.4739 801

35.4830 815

35.4921 860

35.5012 811

35.5103 810

35.5193 801

35.5284 859

35.5375 805

35.5466 777

35.5557 799

35.5648 846

35.5739 807

35.5829 817

35.5920 817

35.6011 845

35.6102 790

35.6193 840

35.6284 814

35.6374 769

35.6465 797

35.6556 817

35.6647 845

35.6738 797

35.6829 796

35.6920 780

35.7010 853

35.7101 804

35.7192 779

35.7283 809

35.7374 812

35.7465 837

35.7555 840

35.7646 804

35.7737 816

35.7828 811

35.7919 764

35.8010 784

35.8101 795

35.8191 796

35.8282 785

35.8373 839

35.8464 828

35.8555 808

35.8646 799

35.8737 818

35.8827 795

35.8918 822

35.9009 805

35.9100 788

35.9191 814

35.9282 835

35.9372 793

35.9463 808

35.9554 827

35.9645 834

35.9736 801

35.9827 853

35.9918 783

36.0008 774

36.0099 800

36.0190 877

36.0281 824

36.0372 831

36.0463 798

36.0553 861

36.0644 801

36.0735 825

36.0826 809

36.0917 847

36.1008 849

36.1099 875

36.1189 781

36.1280 815

36.1371 789

36.1462 797

36.1553 840

36.1644 826

36.1735 833

36.1825 770

36.1916 887

36.2007 808

36.2098 810

36.2189 773

36.2280 886

36.2370 818

36.2461 840

36.2552 824

36.2643 844

36.2734 824

36.2825 871

36.2916 827

36.3006 825

36.3097 819

36.3188 846

36.3279 807

36.3370 795

36.3461 889

36.3551 810

36.3642 823

36.3733 840

36.3824 793

36.3915 842

36.4006 791

36.4097 781

36.4187 809

36.4278 831

36.4369 806

36.4460 794

36.4551 826

36.4642 855

36.4733 804

36.4823 869

36.4914 836

36.5005 834

36.5096 825

36.5187 805

36.5278 816

36.5368 842

36.5459 846

36.5550 892

36.5641 871

36.5732 807

36.5823 808

36.5914 791

36.6004 817

36.6095 856

36.6186 876

36.6277 819

36.6368 762

36.6459 819

36.6549 787

36.6640 825

36.6731 836

36.6822 788

36.6913 835

36.7004 838

36.7095 852

36.7185 834

36.7276 844

36.7367 806

36.7458 867

36.7549 889

36.7640 821

36.7731 854

36.7821 795

36.7912 834

36.8003 835

36.8094 862

36.8185 813

36.8276 819

36.8366 840

36.8457 822

36.8548 825

36.8639 790

36.8730 815

36.8821 786

36.8912 837

36.9002 851

36.9093 828

36.9184 870

36.9275 809

36.9366 839

36.9457 837

36.9547 823

36.9638 835

36.9729 801

36.9820 788

36.9911 802

37.0002 867

37.0093 807

37.0183 789

37.0274 824

37.0365 783

37.0456 775

37.0547 831

37.0638 828

37.0729 788

37.0819 834

37.0910 868

37.1001 774

37.1092 820

37.1183 843

37.1274 811

37.1364 796

37.1455 802

37.1546 773

37.1637 833

37.1728 821

37.1819 830

37.1910 828

37.2000 810

37.2091 842

37.2182 868

37.2273 866

37.2364 840

37.2455 818

37.2546 826

37.2636 828

37.2727 833

37.2818 764

37.2909 827

37.3000 813

37.3091 788

37.3181 791

37.3272 812

37.3363 801

37.3454 798

37.3545 833

37.3636 773

37.3727 808

37.3817 789

37.3908 834

37.3999 824

37.4090 783

37.4181 858

37.4272 839

37.4362 762

37.4453 828

37.4544 814

37.4635 765

37.4726 848

37.4817 800

37.4908 792

37.4998 821

37.5089 791

37.5180 777

37.5271 795

37.5362 797

37.5453 808

37.5544 761

37.5634 763

37.5725 806

37.5816 758

37.5907 831

37.5998 787

37.6089 798

37.6179 840

37.6270 744

37.6361 847

37.6452 818

37.6543 786

37.6634 805

37.6725 765

37.6815 774

37.6906 824

37.6997 801

37.7088 800

37.7179 756

37.7270 784

37.7360 776

37.7451 790

37.7542 778

37.7633 762

37.7724 814

37.7815 778

37.7906 799

37.7996 766

37.8087 773

37.8178 814

37.8269 774

37.8360 700

37.8451 764

37.8542 784

37.8632 758

37.8723 761

37.8814 811

37.8905 822

37.8996 807

37.9087 752

37.9177 795

37.9268 759

37.9359 778

37.9450 755

37.9541 825

37.9632 790

37.9723 773

37.9813 798

37.9904 737

37.9995 776

38.0086 768

38.0177 734

38.0268 778

38.0358 753

38.0449 748

38.0540 764

38.0631 778

38.0722 781

38.0813 756

38.0904 775

38.0994 778

38.1085 767

38.1176 779

38.1267 799

38.1358 749

38.1449 786

38.1540 755

38.1630 755

38.1721 829

38.1812 754

38.1903 775

38.1994 756

38.2085 734

38.2175 786

38.2266 803

38.2357 781

38.2448 730

38.2539 772

38.2630 747

38.2721 753

38.2811 738

38.2902 746

38.2993 836

38.3084 782

38.3175 767

38.3266 742

38.3356 789

38.3447 768

38.3538 804

38.3629 774

38.3720 704

38.3811 779

38.3902 794

38.3992 765

38.4083 822

38.4174 728

38.4265 806

38.4356 772

38.4447 784

38.4538 789

38.4628 750

38.4719 725

38.4810 740

38.4901 704

38.4992 784

38.5083 711

38.5173 779

38.5264 749

38.5355 746

38.5446 766

38.5537 761

38.5628 808

38.5719 728

38.5809 734

38.5900 758

38.5991 765

38.6082 759

38.6173 769

38.6264 735

38.6354 779

38.6445 715

38.6536 788

38.6627 762

38.6718 762

38.6809 706

38.6900 755

38.6990 786

38.7081 725

38.7172 729

38.7263 754

38.7354 801

38.7445 783

38.7536 732

38.7626 811

38.7717 752

38.7808 799

38.7899 772

38.7990 744

38.8081 788

38.8171 781

38.8262 737

38.8353 786

38.8444 717

38.8535 786

38.8626 711

38.8717 755

38.8807 728

38.8898 816

38.8989 751

38.9080 769

38.9171 749

38.9262 736

38.9352 744

38.9443 732

38.9534 736

38.9625 744

38.9716 757

38.9807 733

38.9898 812

38.9988 762

39.0079 757

39.0170 713

39.0261 756

39.0352 777

39.0443 734

39.0534 757

39.0624 752

39.0715 785

39.0806 764

39.0897 725

39.0988 711

39.1079 713

39.1169 742

39.1260 784

39.1351 747

39.1442 795

39.1533 723

39.1624 737

39.1715 708

39.1805 756

39.1896 772

39.1987 783

39.2078 746

39.2169 762

39.2260 825

39.2350 737

39.2441 755

39.2532 737

39.2623 742

39.2714 753

39.2805 808

39.2896 713

39.2986 739

39.3077 681

39.3168 734

39.3259 749

39.3350 711

39.3441 764

39.3532 757

39.3622 723

39.3713 750

39.3804 725

39.3895 793

39.3986 787

39.4077 730

39.4167 740

39.4258 763

39.4349 746

39.4440 741

39.4531 777

39.4622 699

39.4713 777

39.4803 847

39.4894 745

39.4985 777

39.5076 738

39.5167 757

39.5258 790

39.5348 724

39.5439 760

39.5530 761

39.5621 772

39.5712 766

39.5803 757

39.5894 706

39.5984 748

39.6075 725

39.6166 799

39.6257 745

39.6348 758

39.6439 769

39.6530 747

39.6620 765

39.6711 799

39.6802 733

39.6893 793

39.6984 783

39.7075 748

39.7165 794

39.7256 756

39.7347 737

39.7438 751

39.7529 762

39.7620 757

39.7711 740

39.7801 752

39.7892 740

39.7983 771

39.8074 772

39.8165 811

39.8256 747

39.8346 724

39.8437 724

39.8528 762

39.8619 738

39.8710 751

39.8801 759

39.8892 756

39.8982 721

39.9073 720

39.9164 735

39.9255 736

39.9346 754

39.9437 744

39.9528 777

39.9618 729

39.9709 770

39.9800 768

39.9891 787

39.9982 728

40.0073 734

40.0163 739

40.0254 778

40.0345 737

40.0436 767

40.0527 727

40.0618 749

40.0709 728

40.0799 759

40.0890 783

40.0981 735

40.1072 801

40.1163 722

40.1254 734

40.1344 769

40.1435 746

40.1526 744

40.1617 717

40.1708 700

40.1799 762

40.1890 746

40.1980 725

40.2071 769

40.2162 751

40.2253 708

40.2344 762

40.2435 755

40.2526 804

40.2616 750

40.2707 712

40.2798 799

40.2889 795

40.2980 720

40.3071 796

40.3161 772

40.3252 718

40.3343 705

40.3434 779

40.3525 827

40.3616 749

40.3707 735

40.3797 720

40.3888 704

40.3979 747

40.4070 688

40.4161 733

40.4252 778

40.4342 733

40.4433 688

40.4524 724

40.4615 751

40.4706 802

40.4797 753

40.4888 770

40.4978 775

40.5069 774

40.5160 684

40.5251 777

40.5342 757

40.5433 730

40.5524 749

40.5614 773

40.5705 765

40.5796 718

40.5887 738

40.5978 717

40.6069 753

40.6159 729

40.6250 715

40.6341 734

40.6432 793

40.6523 762

40.6614 767

40.6705 734

40.6795 788

40.6886 762

40.6977 742

40.7068 767

40.7159 740

40.7250 746

40.7340 704

40.7431 772

40.7522 732

40.7613 752

40.7704 764

40.7795 742

40.7886 744

40.7976 730

40.8067 796

40.8158 728

40.8249 728

40.8340 743

40.8431 757

40.8522 784

40.8612 752

40.8703 712

40.8794 765

40.8885 747

40.8976 775

40.9067 741

40.9157 734

40.9248 726

40.9339 774

40.9430 757

40.9521 773

40.9612 740

40.9703 736

40.9793 760

40.9884 812

40.9975 790

41.0066 697

41.0157 780

41.0248 755

41.0338 766

41.0429 770

41.0520 760

41.0611 768

41.0702 665

41.0793 763

41.0884 806

41.0974 822

41.1065 752

41.1156 716

41.1247 746

41.1338 749

41.1429 768

41.1520 761

41.1610 708

41.1701 783

41.1792 757

41.1883 745

41.1974 751

41.2065 769

41.2155 790

41.2246 773

41.2337 774

41.2428 734

41.2519 762

41.2610 740

41.2701 781

41.2791 755

41.2882 771

41.2973 757

41.3064 768

41.3155 739

41.3246 800

41.3336 748

41.3427 760

41.3518 768

41.3609 739

41.3700 776

41.3791 780

41.3882 782

41.3972 733

41.4063 784

41.4154 792

41.4245 742

41.4336 778

41.4427 777

41.4518 746

41.4608 805

41.4699 767

41.4790 697

41.4881 761

41.4972 788

41.5063 724

41.5153 745

41.5244 769

41.5335 798

41.5426 817

41.5517 784

41.5608 742

41.5699 802

41.5789 743

41.5880 806

41.5971 750

41.6062 808

41.6153 785

41.6244 766

41.6334 726

41.6425 746

41.6516 745

41.6607 753

41.6698 764

41.6789 761

41.6880 793

41.6970 786

41.7061 782

41.7152 769

41.7243 777

41.7334 788

41.7425 777

41.7516 816

41.7606 756

41.7697 764

41.7788 746

41.7879 774

41.7970 706

41.8061 801

41.8151 772

41.8242 819

41.8333 768

41.8424 736

41.8515 812

41.8606 786

41.8697 761

41.8787 775

41.8878 789

41.8969 767

41.9060 772

41.9151 757

41.9242 794

41.9332 848

41.9423 805

41.9514 775

41.9605 783

41.9696 809

41.9787 768

41.9878 792

41.9968 801

42.0059 787

42.0150 806

42.0241 745

42.0332 821

42.0423 852

42.0514 759

42.0604 772

42.0695 794

42.0786 814

42.0877 815

42.0968 824

42.1059 775

42.1149 789

42.1240 797

42.1331 823

42.1422 779

42.1513 848

42.1604 771

42.1695 837

42.1785 762

42.1876 836

42.1967 816

42.2058 802

42.2149 798

42.2240 814

42.2330 782

42.2421 801

42.2512 819

42.2603 805

42.2694 748

42.2785 756

42.2876 830

42.2966 803

42.3057 809

42.3148 773

42.3239 758

42.3330 842

42.3421 803

42.3512 848

42.3602 809

42.3693 853

42.3784 811

42.3875 834

42.3966 819

42.4057 827

42.4147 827

42.4238 812

42.4329 847

42.4420 787

42.4511 812

42.4602 830

42.4693 828

42.4783 860

42.4874 805

42.4965 860

42.5056 862

42.5147 823

42.5238 822

42.5328 811

42.5419 804

42.5510 791

42.5601 833

42.5692 802

42.5783 785

42.5874 861

42.5964 779

42.6055 830

42.6146 814

42.6237 849

42.6328 837

42.6419 756

42.6510 878

42.6600 817

42.6691 786

42.6782 772

42.6873 791

42.6964 830

42.7055 813

42.7145 807

42.7236 846

42.7327 841

42.7418 800

42.7509 863

42.7600 836

42.7691 837

42.7781 838

42.7872 843

42.7963 848

42.8054 842

42.8145 903

42.8236 822

42.8326 825

42.8417 797

42.8508 840

42.8599 876

42.8690 820

42.8781 811

42.8872 818

42.8962 822

42.9053 806

42.9144 807

42.9235 822

42.9326 773

42.9417 815

42.9508 890

42.9598 832

42.9689 739

42.9780 791

42.9871 815

42.9962 777

43.0053 844

43.0143 821

43.0234 803

43.0325 911

43.0416 825

43.0507 773

43.0598 817

43.0689 848

43.0779 792

43.0870 778

43.0961 830

43.1052 797

43.1143 760

43.1234 818

43.1324 772

43.1415 800

43.1506 808

43.1597 777

43.1688 773

43.1779 776

43.1870 777

43.1960 779

43.2051 766

43.2142 779

43.2233 770

43.2324 815

43.2415 758

43.2506 809

43.2596 831

43.2687 834

43.2778 800

43.2869 793

43.2960 771

43.3051 764

43.3141 786

43.3232 779

43.3323 788

43.3414 777

43.3505 722

43.3596 787

43.3687 830

43.3777 789

43.3868 769

43.3959 764

43.4050 829

43.4141 753

43.4232 780

43.4322 789

43.4413 816

43.4504 832

43.4595 783

43.4686 802

43.4777 766

43.4868 758

43.4958 791

43.5049 815

43.5140 747

43.5231 801

43.5322 775

43.5413 801

43.5504 780

43.5594 752

43.5685 780

43.5776 724

43.5867 689

43.5958 769

43.6049 821

43.6139 758

43.6230 786

43.6321 789

43.6412 804

43.6503 798

43.6594 770

43.6685 783

43.6775 744

43.6866 719

43.6957 766

43.7048 714

43.7139 803

43.7230 778

43.7320 722

43.7411 748

43.7502 763

43.7593 766

43.7684 707

43.7775 749

43.7866 755

43.7956 717

43.8047 785

43.8138 785

43.8229 739

43.8320 746

43.8411 760

43.8502 709

43.8592 765

43.8683 779

43.8774 755

43.8865 775

43.8956 729

43.9047 725

43.9137 705

43.9228 741

43.9319 751

43.9410 720

43.9501 763

43.9592 768

43.9683 737

43.9773 763

43.9864 757

43.9955 709

44.0046 732

44.0137 756

44.0228 773

44.0318 830

44.0409 736

44.0500 754

44.0591 767

44.0682 707

44.0773 750

44.0864 698

44.0954 779

44.1045 720

44.1136 751

44.1227 716

44.1318 748

44.1409 775

44.1500 746

44.1590 790

44.1681 757

44.1772 794

44.1863 728

44.1954 737

44.2045 718

44.2135 774

44.2226 796

44.2317 707

44.2408 689

44.2499 731

44.2590 726

44.2681 716

44.2771 725

44.2862 705

44.2953 680

44.3044 761

44.3135 719

44.3226 736

44.3316 739

44.3407 735

44.3498 736

44.3589 743

44.3680 728

44.3771 755

44.3862 727

44.3952 710

44.4043 776

44.4134 656

44.4225 678

44.4316 724

44.4407 743

44.4498 733

44.4588 743

44.4679 707

44.4770 732

44.4861 711

44.4952 741

44.5043 714

44.5133 741

44.5224 740

44.5315 740

44.5406 699

44.5497 743

44.5588 734

44.5679 710

44.5769 754

44.5860 710

44.5951 717

44.6042 683

44.6133 726

44.6224 751

44.6314 714

44.6405 740

44.6496 748

44.6587 712

44.6678 695

44.6769 759

44.6860 675

44.6950 726

44.7041 703

44.7132 693

44.7223 702

44.7314 787

44.7405 746

44.7496 737

44.7586 731

44.7677 729

44.7768 722

44.7859 678

44.7950 696

44.8041 701

44.8131 673

44.8222 691

44.8313 706

44.8404 748

44.8495 698

44.8586 690

44.8677 749

44.8767 699

44.8858 712

44.8949 698

44.9040 734

44.9131 739

44.9222 753

44.9312 675

44.9403 746

44.9494 730

44.9585 694

44.9676 690

44.9767 713

44.9858 724

44.9948 759

45.0039 655

45.0130 670

45.0221 707

45.0312 660

45.0403 713

45.0494 739

45.0584 698

45.0675 644

45.0766 655

45.0857 687

45.0948 704

45.1039 733

45.1129 727

45.1220 706

45.1311 697

45.1402 709

45.1493 712

45.1584 712

45.1675 727

45.1765 665

45.1856 686

45.1947 752

45.2038 712

45.2129 723

45.2220 678

45.2310 675

45.2401 701

45.2492 693

45.2583 750

45.2674 667

45.2765 684

45.2856 717

45.2946 686

45.3037 710

45.3128 690

45.3219 687

45.3310 684

45.3401 720

45.3492 671

45.3582 706

45.3673 712

45.3764 725

45.3855 654

45.3946 670

45.4037 724

45.4127 680

45.4218 711

45.4309 714

45.4400 709

45.4491 700

45.4582 670

45.4673 778

45.4763 708

45.4854 689

45.4945 715

45.5036 700

45.5127 675

45.5218 692

45.5308 660

45.5399 709

45.5490 759

45.5581 722

45.5672 712

45.5763 703

45.5854 682

45.5944 703

45.6035 725

45.6126 727

45.6217 696

45.6308 678

45.6399 683

45.6490 670

45.6580 682

45.6671 669

45.6762 741

45.6853 725

45.6944 701

45.7035 689

45.7125 738

45.7216 695

45.7307 756

45.7398 685

45.7489 719

45.7580 731

45.7671 679

45.7761 708

45.7852 656

45.7943 691

45.8034 711

45.8125 684

45.8216 727

45.8306 707

45.8397 718

45.8488 704

45.8579 693

45.8670 707

45.8761 692

45.8852 683

45.8942 726

45.9033 676

45.9124 695

45.9215 704

45.9306 715

45.9397 718

45.9488 688

45.9578 698

45.9669 681

45.9760 771

45.9851 661

45.9942 692

46.0033 689

46.0123 679

46.0214 701

46.0305 681

46.0396 701

46.0487 670

46.0578 720

46.0669 701

46.0759 677

46.0850 700

46.0941 664

46.1032 727

46.1123 707

46.1214 703

46.1304 703

46.1395 686

46.1486 661

46.1577 745

46.1668 680

46.1759 655

46.1850 712

46.1940 656

46.2031 701

46.2122 670

46.2213 707

46.2304 689

46.2395 725

46.2486 672

46.2576 647

46.2667 693

46.2758 641

46.2849 662

46.2940 703

46.3031 663

46.3121 678

46.3212 688

46.3303 695

46.3394 695

46.3485 722

46.3576 674

46.3667 676

46.3757 668

46.3848 668

46.3939 623

46.4030 707

46.4121 668

46.4212 670

46.4302 657

46.4393 649

46.4484 635

46.4575 695

46.4666 666

46.4757 648

46.4848 670

46.4938 689

46.5029 686

46.5120 693

46.5211 687

46.5302 664

46.5393 697

46.5484 647

46.5574 702

46.5665 667

46.5756 674

46.5847 679

46.5938 656

46.6029 641

46.6119 638

46.6210 694

46.6301 712

46.6392 661

46.6483 723

46.6574 682

46.6665 677

46.6755 685

46.6846 698

46.6937 700

46.7028 706

46.7119 684

46.7210 669

46.7300 690

46.7391 703

46.7482 666

46.7573 681

46.7664 656

46.7755 665

46.7846 709

46.7936 647

46.8027 666

46.8118 698

46.8209 619

46.8300 654

46.8391 698

46.8482 726

46.8572 690

46.8663 650

46.8754 703

46.8845 682

46.8936 651

46.9027 671

46.9117 713

46.9208 670

46.9299 675

46.9390 684

46.9481 669

46.9572 687

46.9663 718

46.9753 712

46.9844 699

46.9935 670

47.0026 658

47.0117 626

47.0208 665

47.0298 696

47.0389 662

47.0480 678

47.0571 691

47.0662 686

47.0753 667

47.0844 668

47.0934 645

47.1025 691

47.1116 700

47.1207 707

47.1298 681

47.1389 643

47.1480 699

47.1570 640

47.1661 695

47.1752 641

47.1843 670

47.1934 677

47.2025 675

47.2115 675

47.2206 696

47.2297 686

47.2388 659

47.2479 670

47.2570 660

47.2661 696

47.2751 668

47.2842 676

47.2933 739

47.3024 686

47.3115 634

47.3206 683

47.3296 695

47.3387 655

47.3478 683

47.3569 663

47.3660 688

47.3751 674

47.3842 695

47.3932 704

47.4023 668

47.4114 645

47.4205 649

47.4296 643

47.4387 648

47.4478 702

47.4568 667

47.4659 685

47.4750 670

47.4841 679

47.4932 699

47.5023 659

47.5113 642

47.5204 694

47.5295 672

47.5386 642

47.5477 689

47.5568 663

47.5659 651

47.5749 712

47.5840 637

47.5931 684

47.6022 689

47.6113 680

47.6204 633

47.6294 650

47.6385 687

47.6476 662

47.6567 667

47.6658 710

47.6749 684

47.6840 650

47.6930 643

47.7021 715

47.7112 656

47.7203 709

47.7294 686

47.7385 702

47.7476 684

47.7566 701

47.7657 700

47.7748 670

47.7839 695

47.7930 691

47.8021 653

47.8111 677

47.8202 657

47.8293 689

47.8384 670

47.8475 644

47.8566 636

47.8657 721

47.8747 637

47.8838 654

47.8929 669

47.9020 712

47.9111 691

47.9202 649

47.9292 696

47.9383 688

47.9474 672

47.9565 636

47.9656 658

47.9747 682

47.9838 666

47.9928 672

48.0019 629

48.0110 669

48.0201 685

48.0292 662

48.0383 652

48.0474 664

48.0564 649

48.0655 674

48.0746 664

48.0837 629

48.0928 671

48.1019 638

48.1109 687

48.1200 660

48.1291 703

48.1382 625

48.1473 697

48.1564 662

48.1655 671

48.1745 631

48.1836 654

48.1927 612

48.2018 659

48.2109 684

48.2200 637

48.2290 654

48.2381 681

48.2472 655

48.2563 664

48.2654 665

48.2745 643

48.2836 644

48.2926 642

48.3017 715

48.3108 689

48.3199 699

48.3290 642

48.3381 646

48.3472 654

48.3562 655

48.3653 636

48.3744 641

48.3835 626

48.3926 692

48.4017 691

48.4107 646

48.4198 616

48.4289 670

48.4380 645

48.4471 675

48.4562 654

48.4653 674

48.4743 679

48.4834 699

48.4925 638

48.5016 642

48.5107 679

48.5198 642

48.5288 634

48.5379 722

48.5470 622

48.5561 626

48.5652 655

48.5743 681

48.5834 616

48.5924 659

48.6015 648

48.6106 684

48.6197 616

48.6288 656

48.6379 624

48.6470 663

48.6560 658

48.6651 637

48.6742 673

48.6833 659

48.6924 664

48.7015 650

48.7105 733

48.7196 655

48.7287 655

48.7378 640

48.7469 737

48.7560 676

48.7651 670

48.7741 644

48.7832 632

48.7923 675

48.8014 706

48.8105 606

48.8196 634

48.8286 725

48.8377 623

48.8468 649

48.8559 682

48.8650 648

48.8741 692

48.8832 661

48.8922 691

48.9013 639

48.9104 680

48.9195 689

48.9286 678

48.9377 694

48.9468 672

48.9558 638

48.9649 632

48.9740 647

48.9831 684

48.9922 666

49.0013 640

49.0103 646

49.0194 655

49.0285 645

49.0376 668

49.0467 656

49.0558 614

49.0649 633

49.0739 676

49.0830 633

49.0921 672

49.1012 668

49.1103 666

49.1194 669

49.1284 658

49.1375 723

49.1466 649

49.1557 682

49.1648 699

49.1739 656

49.1830 674

49.1920 610

49.2011 647

49.2102 671

49.2193 665

49.2284 639

49.2375 660

49.2466 682

49.2556 669

49.2647 645

49.2738 669

49.2829 632

49.2920 671

49.3011 676

49.3101 620

49.3192 661

49.3283 668

49.3374 632

49.3465 689

49.3556 697

49.3647 684

49.3737 676

49.3828 687

49.3919 671

49.4010 639

49.4101 662

49.4192 695

49.4282 705

49.4373 645

49.4464 684

49.4555 619

49.4646 629

49.4737 659

49.4828 673

49.4918 677

49.5009 652

49.5100 613

49.5191 655

49.5282 613

49.5373 638

49.5464 648

49.5554 626

49.5645 664

49.5736 651

49.5827 701

49.5918 716

49.6009 644

49.6099 664

49.6190 666

49.6281 619

49.6372 635

49.6463 655

49.6554 668

49.6645 670

49.6735 666

49.6826 640

49.6917 643

49.7008 700

49.7099 631

49.7190 675

49.7280 663

49.7371 633

49.7462 664

49.7553 625

49.7644 613

49.7735 638

49.7826 630

49.7916 662

49.8007 639

49.8098 653

49.8189 629

49.8280 669

49.8371 626

49.8462 640

49.8552 640

49.8643 628

49.8734 663

49.8825 603

49.8916 716

49.9007 667

49.9097 688

49.9188 649

49.9279 659

49.9370 639

49.9461 631

49.9552 654

49.9643 648

49.9733 645

49.9824 658

49.9915 571

50.0006 646

50.0097 674

50.0188 649

50.0278 631

50.0369 631

50.0460 655

50.0551 653

50.0642 672

50.0733 709

50.0824 664

50.0914 657

50.1005 649

50.1096 658

50.1187 643

50.1278 634

50.1369 626

50.1460 655

50.1550 649

50.1641 691

50.1732 630

50.1823 617

50.1914 622

50.2005 685

50.2095 631

50.2186 617

50.2277 681

50.2368 644

50.2459 626

50.2550 639

50.2641 633

50.2731 648

50.2822 654

50.2913 637

50.3004 681

50.3095 690

50.3186 634

50.3276 673

50.3367 588

50.3458 640

50.3549 652

50.3640 637

50.3731 625

50.3822 679

50.3912 658

50.4003 674

50.4094 638

50.4185 621

50.4276 622

50.4367 670

50.4458 673

50.4548 681

50.4639 663

50.4730 695

50.4821 617

50.4912 677

50.5003 657

50.5093 656

50.5184 676

50.5275 676

50.5366 639

50.5457 640

50.5548 705

50.5639 652

50.5729 656

50.5820 636

50.5911 684

50.6002 658

50.6093 645

50.6184 618

50.6274 642

50.6365 638

50.6456 612

50.6547 676

50.6638 609

50.6729 671

50.6820 653

50.6910 652

50.7001 617

50.7092 666

50.7183 665

50.7274 674

50.7365 663

50.7456 677

50.7546 640

50.7637 647

50.7728 671

50.7819 639

50.7910 661

50.8001 625

50.8091 651

50.8182 596

50.8273 661

50.8364 622

50.8455 642

50.8546 667

50.8637 660

50.8727 697

50.8818 696

50.8909 620

50.9000 673

50.9091 627

50.9182 672

50.9272 615

50.9363 665

50.9454 643

50.9545 647

50.9636 649

50.9727 605

50.9818 642

50.9908 690

50.9999 623

51.0090 698

51.0181 689

51.0272 629

51.0363 680

51.0454 680

51.0544 607

51.0635 663

51.0726 591

51.0817 608

51.0908 640

51.0999 652

51.1089 625

51.1180 613

51.1271 646

51.1362 642

51.1453 667

51.1544 635

51.1635 685

51.1725 637

51.1816 667

51.1907 651

51.1998 615

51.2089 647

51.2180 636

51.2270 678

51.2361 588

51.2452 651

51.2543 644

51.2634 587

51.2725 664

51.2816 613

51.2906 675

51.2997 657

51.3088 635

51.3179 627

51.3270 597

51.3361 668

51.3452 670

51.3542 647

51.3633 633

51.3724 639

51.3815 642

51.3906 622

51.3997 629

51.4087 624

51.4178 610

51.4269 637

51.4360 660

51.4451 681

51.4542 648

51.4633 621

51.4723 636

51.4814 691

51.4905 626

51.4996 645

51.5087 660

51.5178 656

51.5268 647

51.5359 650

51.5450 677

51.5541 648

51.5632 637

51.5723 643

51.5814 627

51.5904 645

51.5995 669

51.6086 641

51.6177 598

51.6268 636

51.6359 664

51.6450 633

51.6540 617

51.6631 642

51.6722 627

51.6813 644

51.6904 575

51.6995 642

51.7085 643

51.7176 628

51.7267 589

51.7358 639

51.7449 636

51.7540 631

51.7631 647

51.7721 640

51.7812 637

51.7903 640

51.7994 680

51.8085 624

51.8176 664

51.8266 625

51.8357 644

51.8448 637

51.8539 647

51.8630 585

51.8721 664

51.8812 653

51.8902 610

51.8993 669

51.9084 634

51.9175 608

51.9266 614

51.9357 642

51.9448 621

51.9538 644

51.9629 620

51.9720 651

51.9811 642

51.9902 659

51.9993 595

52.0083 589

52.0174 624

52.0265 680

52.0356 616

52.0447 647

52.0538 571

52.0629 627

52.0719 645

52.0810 622

52.0901 641

52.0992 608

52.1083 649

52.1174 641

52.1264 619

52.1355 625

52.1446 641

52.1537 647

52.1628 631

52.1719 654

52.1810 634

52.1900 650

52.1991 626

52.2082 661

52.2173 659

52.2264 654

52.2355 646

52.2446 652

52.2536 649

52.2627 575

52.2718 624

52.2809 642

52.2900 655

52.2991 651

52.3081 617

52.3172 657

52.3263 653

52.3354 618

52.3445 608

52.3536 656

52.3627 642

52.3717 651

52.3808 634

52.3899 594

52.3990 624

52.4081 604

52.4172 647

52.4262 640

52.4353 649

52.4444 636

52.4535 631

52.4626 613

52.4717 611

52.4808 609

52.4898 647

52.4989 638

52.5080 572

52.5171 682

52.5262 635

52.5353 591

52.5444 639

52.5534 645

52.5625 622

52.5716 640

52.5807 659

52.5898 654

52.5989 596

52.6079 639

52.6170 678

52.6261 636

52.6352 657

52.6443 614

52.6534 601

52.6625 665

52.6715 644

52.6806 630

52.6897 589

52.6988 607

52.7079 662

52.7170 571

52.7260 643

52.7351 640

52.7442 611

52.7533 648

52.7624 681

52.7715 573

52.7806 646

52.7896 644

52.7987 606

52.8078 634

52.8169 678

52.8260 619

52.8351 594

52.8442 617

52.8532 633

52.8623 637

52.8714 623

52.8805 605

52.8896 629

52.8987 648

52.9077 651

52.9168 647

52.9259 625

52.9350 590

52.9441 642

52.9532 633

52.9623 648

52.9713 611

52.9804 658

52.9895 651

52.9986 653

53.0077 638

53.0168 669

53.0258 645

53.0349 619

53.0440 620

53.0531 625

53.0622 617

53.0713 642

53.0804 640

53.0894 649

53.0985 651

53.1076 651

53.1167 563

53.1258 611

53.1349 623

53.1440 610

53.1530 608

53.1621 617

53.1712 596

53.1803 605

53.1894 620

53.1985 587

53.2075 621

53.2166 631

53.2257 622

53.2348 630

53.2439 617

53.2530 629

53.2621 613

53.2711 598

53.2802 647

53.2893 655

53.2984 633

53.3075 646

53.3166 596

53.3256 650

53.3347 639

53.3438 637

53.3529 573

53.3620 623

53.3711 622

53.3802 620

53.3892 622

53.3983 640

53.4074 657

53.4165 615

53.4256 603

53.4347 643

53.4438 653

53.4528 604

53.4619 656

53.4710 601

53.4801 623

53.4892 646

53.4983 628

53.5073 612

53.5164 585

53.5255 591

53.5346 597

53.5437 632

53.5528 643

53.5619 617

53.5709 633

53.5800 615

53.5891 615

53.5982 606

53.6073 573

53.6164 632

53.6254 641

53.6345 627

53.6436 629

53.6527 639

53.6618 642

53.6709 616

53.6800 613

53.6890 627

53.6981 585

53.7072 659

53.7163 632

53.7254 598

53.7345 645

53.7436 602

53.7526 568

53.7617 618

53.7708 575

53.7799 668

53.7890 593

53.7981 624

53.8071 642

53.8162 664

53.8253 602

53.8344 586

53.8435 644

53.8526 628

53.8617 614

53.8707 656

53.8798 629

53.8889 605

53.8980 544

53.9071 607

53.9162 609

53.9252 649

53.9343 643

53.9434 623

53.9525 591

53.9616 593

53.9707 600

53.9798 625

53.9888 637

53.9979 658

54.0070 632

54.0161 628

54.0252 604

54.0343 632

54.0434 633

54.0524 631

54.0615 640

54.0706 623

54.0797 619

54.0888 644

54.0979 625

54.1069 606

54.1160 634

54.1251 589

54.1342 651

54.1433 590

54.1524 598

54.1615 650

54.1705 593

54.1796 624

54.1887 598

54.1978 587

54.2069 611

54.2160 629

54.2250 646

54.2341 667

54.2432 610

54.2523 645

54.2614 599

54.2705 610

54.2796 638

54.2886 597

54.2977 621

54.3068 583

54.3159 612

54.3250 580

54.3341 626

54.3432 555

54.3522 661

54.3613 630

54.3704 606

54.3795 598

54.3886 615

54.3977 632

54.4067 587

54.4158 632

54.4249 581

54.4340 620

54.4431 618

54.4522 608

54.4613 591

54.4703 660

54.4794 651

54.4885 629

54.4976 601

54.5067 636

54.5158 623

54.5248 626

54.5339 629

54.5430 626

54.5521 620

54.5612 611

54.5703 613

54.5794 595

54.5884 646

54.5975 611

54.6066 601

54.6157 599

54.6248 594

54.6339 610

54.6430 610

54.6520 586

54.6611 658

54.6702 592

54.6793 639

54.6884 630

54.6975 628

54.7065 617

54.7156 600

54.7247 599

54.7338 609

54.7429 617

54.7520 633

54.7611 632

54.7701 627

54.7792 590

54.7883 603

54.7974 610

54.8065 619

54.8156 605

54.8246 640

54.8337 619

54.8428 581

54.8519 602

54.8610 582

54.8701 618

54.8792 622

54.8882 625

54.8973 640

54.9064 643

54.9155 628

54.9246 564

54.9337 656

54.9428 593

54.9518 613

54.9609 619

54.9700 656

54.9791 617

54.9882 565

54.9973 587

55.0063 619

55.0154 597

55.0245 602

55.0336 638

55.0427 618

55.0518 655

55.0609 632

55.0699 593

55.0790 581

55.0881 578

55.0972 548

55.1063 603

55.1154 612

55.1244 600

55.1335 629

55.1426 597

55.1517 629

55.1608 662

55.1699 601

55.1790 612

55.1880 616

55.1971 606

55.2062 592

55.2153 585

55.2244 632

55.2335 615

55.2426 605

55.2516 638

55.2607 632

55.2698 559

55.2789 619

55.2880 608

55.2971 623

55.3061 596

55.3152 609

55.3243 601

55.3334 629

55.3425 586

55.3516 614

55.3607 595

55.3697 606

55.3788 598

55.3879 572

55.3970 631

55.4061 622

55.4152 600

55.4243 611

55.4333 612

55.4424 682

55.4515 579

55.4606 654

55.4697 619

55.4788 650

55.4878 612

55.4969 654

55.5060 574

55.5151 617

55.5242 636

55.5333 577

55.5424 569

55.5514 604

55.5605 624

55.5696 577

55.5787 616

55.5878 611

55.5969 631

55.6059 610

55.6150 601

55.6241 588

55.6332 614

55.6423 604

55.6514 643

55.6605 602

55.6695 643

55.6786 602

55.6877 627

55.6968 616

55.7059 618

55.7150 617

55.7241 652

55.7331 620

55.7422 587

55.7513 596

55.7604 606

55.7695 600

55.7786 627

55.7876 612

55.7967 610

55.8058 585

55.8149 585

55.8240 617

55.8331 606

55.8422 600

55.8512 606

55.8603 594

55.8694 612

55.8785 622

55.8876 611

55.8967 612

55.9057 638

55.9148 632

55.9239 582

55.9330 614

55.9421 590

55.9512 584

55.9603 550

55.9693 635

55.9784 604

55.9875 604

55.9966 629

56.0057 570

56.0148 590

56.0239 587

56.0329 592

56.0420 650

56.0511 618

56.0602 600

56.0693 620

56.0784 634

56.0874 593

56.0965 583

56.1056 582

56.1147 604

56.1238 619

56.1329 618

56.1420 604

56.1510 597

56.1601 577

56.1692 602

56.1783 635

56.1874 620

56.1965 617

56.2055 613

56.2146 554

56.2237 611

56.2328 656

56.2419 592

56.2510 604

56.2601 604

56.2691 590

56.2782 612

56.2873 583

56.2964 625

56.3055 633

56.3146 593

56.3237 618

56.3327 593

56.3418 602

56.3509 591

56.3600 606

56.3691 621

56.3782 632

56.3872 618

56.3963 636

56.4054 614

56.4145 570

56.4236 580

56.4327 623

56.4418 555

56.4508 598

56.4599 634

56.4690 590

56.4781 615

56.4872 638

56.4963 612

56.5053 610

56.5144 636

56.5235 601

56.5326 583

56.5417 597

56.5508 614

56.5599 584

56.5689 644

56.5780 582

56.5871 659

56.5962 589

56.6053 567

56.6144 625

56.6235 610

56.6325 641

56.6416 639

56.6507 594

56.6598 602

56.6689 589

56.6780 618

56.6870 624

56.6961 608

56.7052 580

56.7143 600

56.7234 617

56.7325 583

56.7416 627

56.7506 605

56.7597 648

56.7688 582

56.7779 599

56.7870 553

56.7961 581

56.8051 544

56.8142 616

56.8233 593

56.8324 597

56.8415 618

56.8506 670

56.8597 563

56.8687 588

56.8778 611

56.8869 591

56.8960 613

56.9051 555

56.9142 599

56.9233 631

56.9323 588

56.9414 635

56.9505 575

56.9596 618

56.9687 544

56.9778 581

56.9868 573

56.9959 595

57.0050 647

57.0141 611

57.0232 602

57.0323 610

57.0414 550

57.0504 599

57.0595 627

57.0686 647

57.0777 601

57.0868 612

57.0959 644

57.1049 634

57.1140 642

57.1231 548

57.1322 611

57.1413 620

57.1504 618

57.1595 640

57.1685 608

57.1776 603

57.1867 587

57.1958 587

57.2049 617

57.2140 595

57.2231 625

57.2321 642

57.2412 555

57.2503 603

57.2594 605

57.2685 542

57.2776 605

57.2866 629

57.2957 593

57.3048 590

57.3139 596

57.3230 586

57.3321 594

57.3412 594

57.3502 574

57.3593 622

57.3684 638

57.3775 575

57.3866 667

57.3957 624

57.4047 613

57.4138 650

57.4229 584

57.4320 559

57.4411 573

57.4502 641

57.4593 625

57.4683 591

57.4774 555

57.4865 600

57.4956 581

57.5047 595

57.5138 623

57.5229 593

57.5319 543

57.5410 562

57.5501 559

57.5592 619

57.5683 593

57.5774 579

57.5864 608

57.5955 583

57.6046 593

57.6137 611

57.6228 574

57.6319 579

57.6410 607

57.6500 546

57.6591 627

57.6682 588

57.6773 636

57.6864 609

57.6955 556

57.7045 567

57.7136 608

57.7227 646

57.7318 579

57.7409 654

57.7500 613

57.7591 624

57.7681 564

57.7772 598

57.7863 582

57.7954 585

57.8045 561

57.8136 605

57.8227 594

57.8317 610

57.8408 573

57.8499 618

57.8590 618

57.8681 587

57.8772 612

57.8862 584

57.8953 624

57.9044 587

57.9135 584

57.9226 589

57.9317 625

57.9408 598

57.9498 594

57.9589 582

57.9680 613

57.9771 588

57.9862 551

57.9953 593

58.0043 593

58.0134 584

58.0225 571

58.0316 603

58.0407 549

58.0498 540

58.0589 601

58.0679 581

58.0770 600

58.0861 591

58.0952 579

58.1043 605

58.1134 596

58.1225 604

58.1315 600

58.1406 610

58.1497 596

58.1588 604

58.1679 582

58.1770 563

58.1860 579

58.1951 588

58.2042 652

58.2133 604

58.2224 598

58.2315 578

58.2406 591

58.2496 587

58.2587 562

58.2678 580

58.2769 583

58.2860 610

58.2951 635

58.3041 565

58.3132 593

58.3223 519

58.3314 610

58.3405 590

58.3496 591

58.3587 620

58.3677 579

58.3768 645

58.3859 610

58.3950 560

58.4041 548

58.4132 596

58.4223 601

58.4313 563

58.4404 572

58.4495 545

58.4586 579

58.4677 620

58.4768 589

58.4858 601

58.4949 603

58.5040 588

58.5131 666

58.5222 573

58.5313 617

58.5404 601

58.5494 620

58.5585 616

58.5676 626

58.5767 594

58.5858 573

58.5949 607

58.6039 605

58.6130 611

58.6221 589

58.6312 567

58.6403 651

58.6494 574

58.6585 599

58.6675 572

58.6766 607

58.6857 593

58.6948 579

58.7039 609

58.7130 520

58.7221 618

58.7311 587

58.7402 599

58.7493 606

58.7584 566

58.7675 639

58.7766 622

58.7856 580

58.7947 579

58.8038 613

58.8129 606

58.8220 595

58.8311 602

58.8402 591

58.8492 622

58.8583 634

58.8674 606

58.8765 611

58.8856 602

58.8947 625

58.9037 597

58.9128 578

58.9219 566

58.9310 631

58.9401 608

58.9492 588

58.9583 596

58.9673 553

58.9764 626

58.9855 598

58.9946 586

59.0037 608

59.0128 590

59.0219 572

59.0309 579

59.0400 611

59.0491 591

59.0582 641

59.0673 615

59.0764 597

59.0854 613

59.0945 622

59.1036 603

59.1127 569

59.1218 574

59.1309 614

59.1400 592

59.1490 563

59.1581 561

59.1672 623

59.1763 566

59.1854 575

59.1945 577

59.2035 616

59.2126 597

59.2217 608

59.2308 573

59.2399 606

59.2490 567

59.2581 589

59.2671 577

59.2762 609

59.2853 557

59.2944 544

59.3035 628

59.3126 597

59.3217 611

59.3307 592

59.3398 580

59.3489 619

59.3580 584

59.3671 570

59.3762 557

59.3852 563

59.3943 574

59.4034 571

59.4125 624

59.4216 646

59.4307 580

59.4398 576

59.4488 551

59.4579 613

59.4670 586

59.4761 598

59.4852 589

59.4943 573

59.5033 627

59.5124 572

59.5215 591

59.5306 575

59.5397 549

59.5488 611

59.5579 597

59.5669 560

59.5760 552

59.5851 588

59.5942 598

59.6033 573

59.6124 631

59.6215 632

59.6305 602

59.6396 584

59.6487 594

59.6578 605

59.6669 539

59.6760 549

59.6850 608

59.6941 606

59.7032 605

59.7123 556

59.7214 602

59.7305 616

59.7396 593

59.7486 622

59.7577 587

59.7668 543

59.7759 618

59.7850 545

59.7941 591

59.8031 630

59.8122 577

59.8213 600

59.8304 600

59.8395 598

59.8486 611

59.8577 570

59.8667 587

59.8758 605

59.8849 625

59.8940 606

59.9031 619

59.9122 609

59.9213 603

59.9303 607

59.9394 590

59.9485 613

59.9576 573

59.9667 629

59.9758 596

59.9848 626

59.9939 616

60.0030 551

60.0121 612

60.0212 628

60.0303 620

60.0394 585

60.0484 566

60.0575 572

60.0666 603

60.0757 593

60.0848 606

60.0939 589

60.1029 595

60.1120 584

60.1211 601

60.1302 605

60.1393 607

60.1484 573

60.1575 562

60.1665 575

60.1756 599

60.1847 586

60.1938 562

60.2029 540

60.2120 600

60.2211 544

60.2301 621

60.2392 597

60.2483 553

60.2574 644

60.2665 611

60.2756 593

60.2846 583

60.2937 614

60.3028 610

60.3119 580

60.3210 571

60.3301 589

60.3392 584

60.3482 616

60.3573 632

60.3664 605

60.3755 601

60.3846 584

60.3937 576

60.4027 592

60.4118 582

60.4209 573

60.4300 595

60.4391 584

60.4482 571

60.4573 595

60.4663 614

60.4754 581

60.4845 620

60.4936 585

60.5027 616

60.5118 550

60.5209 615

60.5299 575

60.5390 647

60.5481 581

60.5572 585

60.5663 549

60.5754 583

60.5844 570

60.5935 564

60.6026 595

60.6117 619

60.6208 567

60.6299 583

60.6390 587

60.6480 597

60.6571 623

60.6662 603

60.6753 595

60.6844 597

60.6935 629

60.7025 618

60.7116 559

60.7207 581

60.7298 600

60.7389 567

60.7480 616

60.7571 604

60.7661 588

60.7752 606

60.7843 582

60.7934 590

60.8025 602

60.8116 616

60.8207 603

60.8297 599

60.8388 626

60.8479 608

60.8570 597

60.8661 612

60.8752 609

60.8842 586

60.8933 605

60.9024 637

60.9115 556

60.9206 571

60.9297 624

60.9388 588

60.9478 594

60.9569 615

60.9660 622

60.9751 656

60.9842 567

60.9933 603

61.0023 580

61.0114 597

61.0205 630

61.0296 587

61.0387 600

61.0478 574

61.0569 627

61.0659 615

61.0750 611

61.0841 610

61.0932 594

61.1023 582

61.1114 633

61.1205 606

61.1295 591

61.1386 631

61.1477 571

61.1568 621

61.1659 577

61.1750 581

61.1840 602

61.1931 605

61.2022 577

61.2113 583

61.2204 633

61.2295 628

61.2386 554

61.2476 590

61.2567 611

61.2658 581

61.2749 610

61.2840 624

61.2931 649

61.3021 572

61.3112 621

61.3203 590

61.3294 635

61.3385 565

61.3476 600

61.3567 606

61.3657 605

61.3748 558

61.3839 617

61.3930 605

61.4021 621

61.4112 577

61.4203 586

61.4293 590

61.4384 610

61.4475 622

61.4566 580

61.4657 567

61.4748 589

61.4838 639

61.4929 619

61.5020 641

61.5111 611

61.5202 631

61.5293 593

61.5384 619

61.5474 625

61.5565 580

61.5656 603

61.5747 641

61.5838 600

61.5929 620

61.6019 576

61.6110 607

61.6201 674

61.6292 593

61.6383 605

61.6474 611

61.6565 601

61.6655 590

61.6746 588

61.6837 578

61.6928 596

61.7019 576

61.7110 622

61.7201 619

61.7291 617

61.7382 566

61.7473 643

61.7564 629

61.7655 661

61.7746 593

61.7836 563

61.7927 630

61.8018 576

61.8109 623

61.8200 601

61.8291 562

61.8382 632

61.8472 571

61.8563 641

61.8654 620

61.8745 566

61.8836 613

61.8927 615

61.9017 630

61.9108 617

61.9199 617

61.9290 581

61.9381 610

61.9472 615

61.9563 610

61.9653 637

61.9744 631

61.9835 616

61.9926 599

62.0017 618

62.0108 561

62.0199 573

62.0289 599

62.0380 604

62.0471 588

62.0562 631

62.0653 612

62.0744 610

62.0834 631

62.0925 619

62.1016 585

62.1107 590

62.1198 635

62.1289 628

62.1380 580

62.1470 602

62.1561 601

62.1652 578

62.1743 616

62.1834 668

62.1925 605

62.2015 597

62.2106 610

62.2197 626

62.2288 632

62.2379 592

62.2470 559

62.2561 599

62.2651 623

62.2742 592

62.2833 654

62.2924 616

62.3015 585

62.3106 627

62.3197 577

62.3287 590

62.3378 634

62.3469 608

62.3560 605

62.3651 613

62.3742 569

62.3832 645

62.3923 589

62.4014 605

62.4105 562

62.4196 588

62.4287 613

62.4378 610

62.4468 573

62.4559 594

62.4650 579

62.4741 583

62.4832 562

62.4923 625

62.5013 594

62.5104 634

62.5195 634

62.5286 567

62.5377 562

62.5468 583

62.5559 599

62.5649 626

62.5740 600

62.5831 604

62.5922 593

62.6013 598

62.6104 579

62.6195 580

62.6285 635

62.6376 537

62.6467 595

62.6558 591

62.6649 613

62.6740 572

62.6830 605

62.6921 575

62.7012 597

62.7103 624

62.7194 638

62.7285 598

62.7376 558

62.7466 568

62.7557 615

62.7648 573

62.7739 601

62.7830 575

62.7921 573

62.8011 642

62.8102 544

62.8193 594

62.8284 608

62.8375 548

62.8466 579

62.8557 599

62.8647 562

62.8738 573

62.8829 574

62.8920 605

62.9011 555

62.9102 583

62.9193 569

62.9283 581

62.9374 590

62.9465 593

62.9556 586

62.9647 596

62.9738 609

62.9828 616

62.9919 578

63.0010 542

63.0101 587

63.0192 612

63.0283 611

63.0374 571

63.0464 645

63.0555 583

63.0646 597

63.0737 581

63.0828 591

63.0919 602

63.1009 576

63.1100 593

63.1191 570

63.1282 556

63.1373 590

63.1464 586

63.1555 600

63.1645 595

63.1736 544

63.1827 549

63.1918 579

63.2009 619

63.2100 638

63.2191 567

63.2281 587

63.2372 583

63.2463 593

63.2554 555

63.2645 610

63.2736 595

63.2826 613

63.2917 587

63.3008 582

63.3099 549

63.3190 603

63.3281 624

63.3372 541

63.3462 541

63.3553 573

63.3644 607

63.3735 543

63.3826 602

63.3917 538

63.4007 576

63.4098 567

63.4189 587

63.4280 588

63.4371 575

63.4462 554

63.4553 560

63.4643 566

63.4734 572

63.4825 526

63.4916 547

63.5007 547

63.5098 583

63.5189 566

63.5279 591

63.5370 570

63.5461 560

63.5552 577

63.5643 583

63.5734 554

63.5824 573

63.5915 559

63.6006 551

63.6097 547

63.6188 569

63.6279 592

63.6370 597

63.6460 601

63.6551 608

63.6642 580

63.6733 597

63.6824 566

63.6915 560

63.7005 578

63.7096 584

63.7187 557

63.7278 586

63.7369 563

63.7460 556

63.7551 592

63.7641 598

63.7732 585

63.7823 554

63.7914 566

63.8005 604

63.8096 551

63.8187 581

63.8277 559

63.8368 565

63.8459 507

63.8550 618

63.8641 566

63.8732 608

63.8822 601

63.8913 568

63.9004 547

63.9095 513

63.9186 588

63.9277 593

63.9368 581

63.9458 580

63.9549 573

63.9640 586

63.9731 543

63.9822 578

63.9913 562

64.0003 561

64.0094 584

64.0185 560

64.0276 518

64.0367 582

64.0458 544

64.0549 602

64.0639 563

64.0730 557

64.0821 540

64.0912 568

64.1003 564

64.1094 590

64.1185 555

64.1275 523

64.1366 535

64.1457 525

64.1548 560

64.1639 548

64.1730 533

64.1820 528

64.1911 603

64.2002 528

64.2093 564

64.2184 547

64.2275 557

64.2366 544

64.2456 578

64.2547 570

64.2638 540

64.2729 596

64.2820 560

64.2911 565

64.3001 563

64.3092 542

64.3183 583

64.3274 543

64.3365 542

64.3456 589

64.3547 582

64.3637 572

64.3728 562

64.3819 559

64.3910 534

64.4001 553

64.4092 570

64.4183 540

64.4273 583

64.4364 562

64.4455 587

64.4546 560

64.4637 574

64.4728 543

64.4818 546

64.4909 576

64.5000 544

64.5091 568

64.5182 581

64.5273 562

64.5364 572

64.5454 567

64.5545 587

64.5636 607

64.5727 593

64.5818 606

64.5909 607

64.5999 548

64.6090 562

64.6181 554

64.6272 576

64.6363 544

64.6454 551

64.6545 534

64.6635 584

64.6726 574

64.6817 571

64.6908 568

64.6999 529

64.7090 523

64.7181 561

64.7271 573

64.7362 570

64.7453 491

64.7544 532

64.7635 545

64.7726 577

64.7816 578

64.7907 547

64.7998 592

64.8089 551

64.8180 569

64.8271 548

64.8362 551

64.8452 533

64.8543 549

64.8634 541

64.8725 536

64.8816 553

64.8907 586

64.8997 602

64.9088 553

64.9179 541

64.9270 607

64.9361 553

64.9452 571

64.9543 564

64.9633 549

64.9724 547

64.9815 574

64.9906 550

64.9997 544

65.0088 590

65.0179 572

65.0269 547

65.0360 540

65.0451 552

65.0542 588

65.0633 575

65.0724 564

65.0814 553

65.0905 533

65.0996 580

65.1087 574

65.1178 536

65.1269 573

65.1360 540

65.1450 559

65.1541 554

65.1632 592

65.1723 536

65.1814 606

65.1905 559

65.1995 567

65.2086 534

65.2177 541

65.2268 576

65.2359 628

65.2450 536

65.2541 557

65.2631 540

65.2722 583

65.2813 544

65.2904 553

65.2995 576

65.3086 539

65.3177 567

65.3267 547

65.3358 547

65.3449 506

65.3540 579

65.3631 573

65.3722 540

65.3812 527

65.3903 553

65.3994 552

65.4085 517

65.4176 563

65.4267 553

65.4358 571

65.4448 563

65.4539 563

65.4630 551

65.4721 537

65.4812 565

65.4903 530

65.4993 559

65.5084 553

65.5175 558

65.5266 519

65.5357 578

65.5448 535

65.5539 549

65.5629 578

65.5720 606

65.5811 583

65.5902 526

65.5993 531

65.6084 547

65.6175 548

65.6265 540

65.6356 534

65.6447 524

65.6538 559

65.6629 579

65.6720 522

65.6810 529

65.6901 527

65.6992 564

65.7083 558

65.7174 560

65.7265 593

65.7356 553

65.7446 583

65.7537 584

65.7628 569

65.7719 528

65.7810 555

65.7901 553

65.7991 544

65.8082 599

65.8173 521

65.8264 518

65.8355 534

65.8446 578

65.8537 528

65.8627 534

65.8718 556

65.8809 563

65.8900 528

65.8991 531

65.9082 553

65.9173 533

65.9263 584

65.9354 587

65.9445 586

65.9536 545

65.9627 527

65.9718 573

65.9808 561

65.9899 557

65.9990 574

66.0081 531

66.0172 571

66.0263 518

66.0354 546

66.0444 513

66.0535 529

66.0626 583

66.0717 530

66.0808 508

66.0899 576

66.0989 528

66.1080 545

66.1171 557

66.1262 563

66.1353 572

66.1444 571

66.1535 559

66.1625 577

66.1716 552

66.1807 562

66.1898 561

66.1989 579

66.2080 537

66.2171 608

66.2261 542

66.2352 553

66.2443 549

66.2534 548

66.2625 544

66.2716 598

66.2806 521

66.2897 572

66.2988 536

66.3079 543

66.3170 546

66.3261 527

66.3352 543

66.3442 554

66.3533 536

66.3624 514

66.3715 539

66.3806 545

66.3897 536

66.3987 543

66.4078 571

66.4169 566

66.4260 534

66.4351 567

66.4442 572

66.4533 575

66.4623 546

66.4714 522

66.4805 586

66.4896 545

66.4987 556

66.5078 574

66.5169 531

66.5259 603

66.5350 525

66.5441 549

66.5532 508

66.5623 562

66.5714 561

66.5804 542

66.5895 536

66.5986 585

66.6077 522

66.6168 582

66.6259 551

66.6350 529

66.6440 544

66.6531 555

66.6622 546

66.6713 547

66.6804 572

66.6895 556

66.6985 544

66.7076 550

66.7167 509

66.7258 514

66.7349 574

66.7440 561

66.7531 573

66.7621 569

66.7712 548

66.7803 548

66.7894 540

66.7985 530

66.8076 550

66.8167 518

66.8257 501

66.8348 527

66.8439 586

66.8530 565

66.8621 521

66.8712 517

66.8802 565

66.8893 537

66.8984 551

66.9075 525

66.9166 560

66.9257 543

66.9348 549

66.9438 574

66.9529 528

66.9620 551

66.9711 518

66.9802 517

66.9893 541

66.9983 512

67.0074 558

67.0165 535

67.0256 531

67.0347 483

67.0438 531

67.0529 576

67.0619 540

67.0710 555

67.0801 520

67.0892 488

67.0983 531

67.1074 539

67.1165 545

67.1255 540

67.1346 532

67.1437 554

67.1528 566

67.1619 550

67.1710 566

67.1800 527

67.1891 551

67.1982 573

67.2073 552

67.2164 537

67.2255 585

67.2346 538

67.2436 531

67.2527 501

67.2618 545

67.2709 566

67.2800 535

67.2891 538

67.2981 557

67.3072 568

67.3163 543

67.3254 552

67.3345 542

67.3436 530

67.3527 565

67.3617 571

67.3708 581

67.3799 537

67.3890 525

67.3981 572

67.4072 587

67.4163 538

67.4253 499

67.4344 549

67.4435 524

67.4526 538

67.4617 585

67.4708 537

67.4798 535

67.4889 575

67.4980 515

67.5071 465

67.5162 555

67.5253 502

67.5344 561

67.5434 573

67.5525 562

67.5616 542

67.5707 560

67.5798 512

67.5889 521

67.5979 498

67.6070 537

67.6161 538

67.6252 554

67.6343 531

67.6434 514

67.6525 576

67.6615 528

67.6706 583

67.6797 567

67.6888 510

67.6979 528

67.7070 540

67.7161 545

67.7251 529

67.7342 580

67.7433 548

67.7524 517

67.7615 521

67.7706 512

67.7796 559

67.7887 529

67.7978 541

67.8069 538

67.8160 562

67.8251 572

67.8342 569

67.8432 556

67.8523 567

67.8614 528

67.8705 481

67.8796 587

67.8887 540

67.8977 561

67.9068 526

67.9159 556

67.9250 530

67.9341 544

67.9432 513

67.9523 554

67.9613 532

67.9704 517

67.9795 566

67.9886 554

67.9977 522

68.0068 512

68.0159 493

68.0249 520

68.0340 509

68.0431 534

68.0522 552

68.0613 543

68.0704 521

68.0794 523

68.0885 565

68.0976 551

68.1067 515

68.1158 534

68.1249 561

68.1340 526

68.1430 555

68.1521 588

68.1612 543

68.1703 544

68.1794 547

68.1885 513

68.1975 495

68.2066 523

68.2157 549

68.2248 552

68.2339 514

68.2430 549

68.2521 518

68.2611 558

68.2702 520

68.2793 551

68.2884 573

68.2975 550

68.3066 522

68.3157 560

68.3247 557

68.3338 520

68.3429 557

68.3520 535

68.3611 549

68.3702 535

68.3792 556

68.3883 538

68.3974 524

68.4065 510

68.4156 520

68.4247 536

68.4338 535

68.4428 499

68.4519 554

68.4610 545

68.4701 555

68.4792 519

68.4883 545

68.4973 557

68.5064 528

68.5155 536

68.5246 535

68.5337 519

68.5428 518

68.5519 551

68.5609 547

68.5700 539

68.5791 576

68.5882 521

68.5973 542

68.6064 546

68.6155 565

68.6245 557

68.6336 526

68.6427 532

68.6518 491

68.6609 540

68.6700 506

68.6790 512

68.6881 516

68.6972 538

68.7063 514

68.7154 514

68.7245 583

68.7336 500

68.7426 488

68.7517 535

68.7608 546

68.7699 538

68.7790 517

68.7881 568

68.7971 519

68.8062 498

68.8153 482

68.8244 530

68.8335 545

68.8426 519

68.8517 521

68.8607 512

68.8698 535

68.8789 485

68.8880 527

68.8971 546

68.9062 565

68.9153 520

68.9243 558

68.9334 571

68.9425 507

68.9516 533

68.9607 514

68.9698 550

68.9788 556

68.9879 541

68.9970 525

69.0061 521

69.0152 503

69.0243 526

69.0334 515

69.0424 493

69.0515 579

69.0606 551

69.0697 481

69.0788 539

69.0879 545

69.0969 523

69.1060 564

69.1151 516

69.1242 551

69.1333 537

69.1424 533

69.1515 565

69.1605 532

69.1696 514

69.1787 561

69.1878 542

69.1969 579

69.2060 546

69.2151 557

69.2241 524

69.2332 510

69.2423 493

69.2514 548

69.2605 556

69.2696 533

69.2786 564

69.2877 529

69.2968 519

69.3059 570

69.3150 484

69.3241 495

69.3332 539

69.3422 510

69.3513 549

69.3604 516

69.3695 562

69.3786 501

69.3877 512

69.3967 538

69.4058 536

69.4149 523

69.4240 531

69.4331 542

69.4422 527

69.4513 504

69.4603 538

69.4694 543

69.4785 542

69.4876 536

69.4967 569

69.5058 530

69.5149 568

69.5239 505

69.5330 552

69.5421 541

69.5512 508

69.5603 532

69.5694 491

69.5784 546

69.5875 548

69.5966 498

69.6057 525

69.6148 505

69.6239 510

69.6330 515

69.6420 543

69.6511 533

69.6602 557

69.6693 548

69.6784 510

69.6875 525

69.6965 533

69.7056 506

69.7147 528

69.7238 532

69.7329 505

69.7420 508

69.7511 519

69.7601 505

69.7692 477

69.7783 510

69.7874 540

69.7965 507

69.8056 570

69.8147 547

69.8237 553

69.8328 543

69.8419 520

69.8510 499

69.8601 519

69.8692 497

69.8782 537

69.8873 548

69.8964 523

69.9055 555

69.9146 556

69.9237 570

69.9328 557

69.9418 552

69.9509 570

69.9600 521

69.9691 504

69.9782 523

69.9873 552

69.9963 489

70.0054 537

70.0145 544

70.0236 504

70.0327 506

70.0418 505

70.0509 520

70.0599 564

70.0690 513

70.0781 545

70.0872 539

70.0963 535

70.1054 558

70.1145 530

70.1235 516

70.1326 533

70.1417 529

70.1508 560

70.1599 526

70.1690 517

70.1780 530

70.1871 521

70.1962 511

70.2053 543

70.2144 540

70.2235 534

70.2326 556

70.2416 565

70.2507 557

70.2598 538

70.2689 530

70.2780 598

70.2871 547

70.2961 506

70.3052 522

70.3143 537

70.3234 530

70.3325 570

70.3416 506

70.3507 513

70.3597 546

70.3688 501

70.3779 490

70.3870 533

70.3961 518

70.4052 525

70.4143 567

70.4233 514

70.4324 517

70.4415 500

70.4506 512

70.4597 597

70.4688 543

70.4778 502

70.4869 550

70.4960 498

70.5051 523

70.5142 536

70.5233 558

70.5324 533

70.5414 549

70.5505 542

70.5596 535

70.5687 495

70.5778 501

70.5869 513

70.5959 548

70.6050 537

70.6141 562

70.6232 548

70.6323 541

70.6414 512

70.6505 519

70.6595 538

70.6686 524

70.6777 518

70.6868 552

70.6959 523

70.7050 545

70.7141 537

70.7231 500

70.7322 504

70.7413 531

70.7504 541

70.7595 470

70.7686 541

70.7776 542

70.7867 519

70.7958 520

70.8049 492

70.8140 490

70.8231 519

70.8322 549

70.8412 512

70.8503 507

70.8594 524

70.8685 534

70.8776 519

70.8867 572

70.8957 508

70.9048 523

70.9139 543

70.9230 541

70.9321 532

70.9412 468

70.9503 545

70.9593 565

70.9684 528

70.9775 502

70.9866 524

70.9957 534

71.0048 541

71.0139 534

71.0229 491

71.0320 561

71.0411 528

71.0502 499

71.0593 513

71.0684 515

71.0774 495

71.0865 555

71.0956 537

71.1047 510

71.1138 524

71.1229 531

71.1320 502

71.1410 542

71.1501 521

71.1592 494

71.1683 513

71.1774 539

71.1865 507

71.1955 536

71.2046 508

71.2137 497

71.2228 540

71.2319 559

71.2410 504

71.2501 524

71.2591 520

71.2682 531

71.2773 525

71.2864 531

71.2955 504

71.3046 483

71.3137 496

71.3227 512

71.3318 527

71.3409 526

71.3500 503

71.3591 555

71.3682 501

71.3772 500

71.3863 513

71.3954 532

71.4045 516

71.4136 481

71.4227 514

71.4318 461

71.4408 551

71.4499 532

71.4590 530

71.4681 495

71.4772 505

71.4863 556

71.4953 517

71.5044 491

71.5135 523

71.5226 527

71.5317 492

71.5408 503

71.5499 507

71.5589 515

71.5680 498

71.5771 560

71.5862 508

71.5953 509

71.6044 539

71.6135 540

71.6225 506

71.6316 510

71.6407 523

71.6498 548

71.6589 513

71.6680 516

71.6770 543

71.6861 499

71.6952 521

71.7043 517

71.7134 539

71.7225 533

71.7316 528

71.7406 542

71.7497 493

71.7588 552

71.7679 495

71.7770 519

71.7861 545

71.7951 510

71.8042 542

71.8133 496

71.8224 528

71.8315 532

71.8406 528

71.8497 555

71.8587 520

71.8678 515

71.8769 530

71.8860 488

71.8951 546

71.9042 575

71.9133 533

71.9223 520

71.9314 496

71.9405 519

71.9496 543

71.9587 518

71.9678 582

71.9768 495

71.9859 536

71.9950 531

72.0041 530

72.0132 519

72.0223 491

72.0314 487

72.0404 496

72.0495 510

72.0586 489

72.0677 509

72.0768 521

72.0859 521

72.0949 538

72.1040 495

72.1131 531

72.1222 538

72.1313 529

72.1404 510

72.1495 551

72.1585 524

72.1676 522

72.1767 536

72.1858 511

72.1949 502

72.2040 535

72.2131 489

72.2221 519

72.2312 547

72.2403 497

72.2494 517

72.2585 488

72.2676 557

72.2766 523

72.2857 543

72.2948 519

72.3039 540

72.3130 484

72.3221 528

72.3312 551

72.3402 550

72.3493 537

72.3584 511

72.3675 503

72.3766 501

72.3857 505

72.3947 551

72.4038 532

72.4129 497

72.4220 510

72.4311 488

72.4402 537

72.4493 486

72.4583 491

72.4674 508

72.4765 526

72.4856 528

72.4947 602

72.5038 517

72.5129 495

72.5219 518

72.5310 572

72.5401 510

72.5492 503

72.5583 504

72.5674 576

72.5764 482

72.5855 497

72.5946 505

72.6037 564

72.6128 507

72.6219 507

72.6310 568

72.6400 551

72.6491 539

72.6582 490

72.6673 537

72.6764 529

72.6855 527

72.6945 519

72.7036 508

72.7127 545

72.7218 509

72.7309 497

72.7400 509

72.7491 497

72.7581 526

72.7672 530

72.7763 540

72.7854 531

72.7945 513

72.8036 523

72.8127 505

72.8217 508

72.8308 524

72.8399 498

72.8490 566

72.8581 562

72.8672 516

72.8762 515

72.8853 513

72.8944 546

72.9035 525

72.9126 516

72.9217 509

72.9308 529

72.9398 522

72.9489 557

72.9580 547

72.9671 458

72.9762 469

72.9853 541

72.9943 561

73.0034 544

73.0125 511

73.0216 504

73.0307 527

73.0398 580

73.0489 492

73.0579 550

73.0670 496

73.0761 565

73.0852 503

73.0943 580

73.1034 535

73.1125 536

73.1215 525

73.1306 529

73.1397 506

73.1488 558

73.1579 520

73.1670 525

73.1760 490

73.1851 487

73.1942 506

73.2033 538

73.2124 535

73.2215 534

73.2306 523

73.2396 542

73.2487 489

73.2578 515

73.2669 506

73.2760 467

73.2851 517

73.2941 516

73.3032 522

73.3123 506

73.3214 506

73.3305 526

73.3396 516

73.3487 515

73.3577 542

73.3668 530

73.3759 523

73.3850 536

73.3941 495

73.4032 504

73.4123 530

73.4213 512

73.4304 528

73.4395 525

73.4486 516

73.4577 523

73.4668 533

73.4758 534

73.4849 502

73.4940 525

73.5031 539

73.5122 527

73.5213 487

73.5304 505

73.5394 532

73.5485 530

73.5576 517

73.5667 514

73.5758 519

73.5849 506

73.5940 519

73.6030 483

73.6121 516

73.6212 480

73.6303 506

73.6394 558

73.6485 486

73.6575 528

73.6666 531

73.6757 543

73.6848 523

73.6939 479

73.7030 539

73.7121 487

73.7211 524

73.7302 533

73.7393 523

73.7484 532

73.7575 545

73.7666 522

73.7756 511

73.7847 544

73.7938 537

73.8029 539

73.8120 512

73.8211 509

73.8302 540

73.8392 539

73.8483 503

73.8574 512

73.8665 512

73.8756 517

73.8847 561

73.8938 550

73.9028 516

73.9119 550

73.9210 536

73.9301 511

73.9392 511

73.9483 535

73.9573 536

73.9664 495

73.9755 505

73.9846 512

73.9937 490

74.0028 517

74.0119 514

74.0209 514

74.0300 510

74.0391 533

74.0482 530

74.0573 515

74.0664 495

74.0754 548

74.0845 527

74.0936 543

74.1027 527

74.1118 521

74.1209 547

74.1300 504

74.1390 526

74.1481 489

74.1572 525

74.1663 524

74.1754 568

74.1845 536

74.1936 527

74.2026 524

74.2117 524

74.2208 510

74.2299 508

74.2390 527

74.2481 531

74.2571 533

74.2662 528

74.2753 525

74.2844 484

74.2935 538

74.3026 497

74.3117 532

74.3207 491

74.3298 542

74.3389 507

74.3480 576

74.3571 554

74.3662 549

74.3752 513

74.3843 525

74.3934 524

74.4025 513

74.4116 537

74.4207 480

74.4298 511

74.4388 513

74.4479 537

74.4570 513

74.4661 526

74.4752 518

74.4843 525

74.4934 516

74.5024 504

74.5115 542

74.5206 526

74.5297 545

74.5388 565

74.5479 498

74.5569 516

74.5660 516

74.5751 549

74.5842 558

74.5933 530

74.6024 517

74.6115 524

74.6205 528

74.6296 507

74.6387 561

74.6478 506

74.6569 533

74.6660 545

74.6750 535

74.6841 519

74.6932 542

74.7023 485

74.7114 549

74.7205 501

74.7296 500

74.7386 524

74.7477 494

74.7568 511

74.7659 499

74.7750 556

74.7841 560

74.7932 518

74.8022 571

74.8113 542

74.8204 509

74.8295 536

74.8386 554

74.8477 509

74.8567 525

74.8658 509

74.8749 497

74.8840 496

74.8931 504

74.9022 490

74.9113 520

74.9203 483

74.9294 532

74.9385 502

74.9476 507

74.9567 497

74.9658 488

74.9748 519

74.9839 576

74.9930 508

75.0021 477

75.0112 496

75.0203 548

75.0294 539

75.0384 543

75.0475 513

75.0566 454

75.0657 499

75.0748 546

75.0839 529

75.0930 515

75.1020 532

75.1111 493

75.1202 504

75.1293 529

75.1384 553

75.1475 528

75.1565 507

75.1656 518

75.1747 505

75.1838 474

75.1929 542

75.2020 508

75.2111 491

75.2201 496

75.2292 507

75.2383 483

75.2474 506

75.2565 507

75.2656 534

75.2746 513

75.2837 470

75.2928 501

75.3019 491

75.3110 506

75.3201 514

75.3292 513

75.3382 564

75.3473 519

75.3564 535

75.3655 515

75.3746 544

75.3837 511

75.3928 496

75.4018 575

75.4109 490

75.4200 537

75.4291 518

75.4382 534

75.4473 539

75.4563 518

75.4654 495

75.4745 500

75.4836 528

75.4927 513

75.5018 545

75.5109 507

75.5199 491

75.5290 536

75.5381 478

75.5472 510

75.5563 500

75.5654 499

75.5744 482

75.5835 516

75.5926 514

75.6017 541

75.6108 487

75.6199 467

75.6290 518

75.6380 473

75.6471 517

75.6562 474

75.6653 493

75.6744 550

75.6835 551

75.6926 504

75.7016 510

75.7107 531

75.7198 510

75.7289 514

75.7380 522

75.7471 494

75.7561 516

75.7652 478

75.7743 524

75.7834 550

75.7925 472

75.8016 484

75.8107 518

75.8197 530

75.8288 499

75.8379 515

75.8470 511

75.8561 483

75.8652 519

75.8742 536

75.8833 500

75.8924 525

75.9015 529

75.9106 500

75.9197 502

75.9288 517

75.9378 540

75.9469 550

75.9560 493

75.9651 507

75.9742 490

75.9833 509

75.9924 508

76.0014 535

76.0105 515

76.0196 520

76.0287 486

76.0378 537

76.0469 494

76.0559 547

76.0650 508

76.0741 470

76.0832 510

76.0923 525

76.1014 514

76.1105 509

76.1195 490

76.1286 473

76.1377 535

76.1468 522

76.1559 491

76.1650 503

76.1740 521

76.1831 508

76.1922 454

76.2013 532

76.2104 521

76.2195 550

76.2286 547

76.2376 532

76.2467 476

76.2558 519

76.2649 483

76.2740 479

76.2831 529

76.2922 513

76.3012 471

76.3103 469

76.3194 481

76.3285 499

76.3376 545

76.3467 516

76.3557 533

76.3648 483

76.3739 482

76.3830 534

76.3921 494

76.4012 528

76.4103 474

76.4193 479

76.4284 503

76.4375 500

76.4466 494

76.4557 522

76.4648 484

76.4738 469

76.4829 502

76.4920 493

76.5011 518

76.5102 489

76.5193 479

76.5284 502

76.5374 487

76.5465 510

76.5556 539

76.5647 492

76.5738 539

76.5829 498

76.5920 486

76.6010 468

76.6101 497

76.6192 455

76.6283 496

76.6374 529

76.6465 532

76.6555 508

76.6646 521

76.6737 532

76.6828 505

76.6919 512

76.7010 490

76.7101 517

76.7191 531

76.7282 491

76.7373 503

76.7464 485

76.7555 544

76.7646 516

76.7736 525

76.7827 481

76.7918 497

76.8009 515

76.8100 486

76.8191 527

76.8282 501

76.8372 516

76.8463 483

76.8554 518

76.8645 491

76.8736 499

76.8827 515

76.8918 512

76.9008 511

76.9099 483

76.9190 483

76.9281 546

76.9372 491

76.9463 465

76.9553 500

76.9644 505

76.9735 519

76.9826 482

76.9917 546

77.0008 521

77.0099 491

77.0189 529

77.0280 537

77.0371 481

77.0462 490

77.0553 533

77.0644 508

77.0734 520

77.0825 500

77.0916 503

77.1007 490

77.1098 514

77.1189 517

77.1280 496

77.1370 497

77.1461 507

77.1552 538

77.1643 484

77.1734 453

77.1825 478

77.1916 456

77.2006 475

77.2097 489

77.2188 505

77.2279 531

77.2370 533

77.2461 528

77.2551 514

77.2642 503

77.2733 515

77.2824 509

77.2915 526

77.3006 512

77.3097 513

77.3187 469

77.3278 525

77.3369 497

77.3460 501

77.3551 507

77.3642 484

77.3732 462

77.3823 486

77.3914 482

77.4005 532

77.4096 507

77.4187 515

77.4278 509

77.4368 496

77.4459 500

77.4550 491

77.4641 499

77.4732 520

77.4823 471

77.4914 500

77.5004 504

77.5095 502

77.5186 521

77.5277 512

77.5368 482

77.5459 524

77.5549 531

77.5640 484

77.5731 497

77.5822 511

77.5913 484

77.6004 520

77.6095 487

77.6185 481

77.6276 494

77.6367 526

77.6458 520

77.6549 486

77.6640 507

77.6730 487

77.6821 536

77.6912 496

77.7003 506

77.7094 520

77.7185 477

77.7276 530

77.7366 529

77.7457 493

77.7548 546

77.7639 495

77.7730 483

77.7821 500

77.7912 491

77.8002 519

77.8093 504

77.8184 542

77.8275 501

77.8366 507

77.8457 506

77.8547 509

77.8638 541

77.8729 515

77.8820 544

77.8911 502

77.9002 466

77.9093 497

77.9183 508

77.9274 506

77.9365 495

77.9456 514

77.9547 491

77.9638 504

77.9728 462

77.9819 495

77.9910 508

78.0001 541

78.0092 549

78.0183 548

78.0274 483

78.0364 521

78.0455 491

78.0546 495

78.0637 483

78.0728 499

78.0819 469

78.0910 503

78.1000 496

78.1091 512

78.1182 500

78.1273 471

78.1364 524

78.1455 523

78.1545 526

78.1636 503

78.1727 515

78.1818 462

78.1909 491

78.2000 503

78.2091 507

78.2181 489

78.2272 498

78.2363 502

78.2454 468

78.2545 519

78.2636 538

78.2726 517

78.2817 496

78.2908 492

78.2999 513

78.3090 545

78.3181 469

78.3272 527

78.3362 500

78.3453 471

78.3544 456

78.3635 495

78.3726 489

78.3817 508

78.3908 463

78.3998 518

78.4089 513

78.4180 540

78.4271 504

78.4362 505

78.4453 541

78.4543 513

78.4634 504

78.4725 496

78.4816 480

78.4907 525

78.4998 545

78.5089 510

78.5179 535

78.5270 498

78.5361 492

78.5452 493

78.5543 504

78.5634 520

78.5724 558

78.5815 502

78.5906 524

78.5997 490

78.6088 502

78.6179 540

78.6270 533

78.6360 468

78.6451 518

78.6542 489

78.6633 521

78.6724 519

78.6815 546

78.6906 494

78.6996 500

78.7087 498

78.7178 510

78.7269 497

78.7360 516

78.7451 497

78.7541 507

78.7632 473

78.7723 455

78.7814 533

78.7905 504

78.7996 492

78.8087 490

78.8177 518

78.8268 526

78.8359 499

78.8450 482

78.8541 512

78.8632 493

78.8722 501

78.8813 515

78.8904 497

78.8995 486

78.9086 480

78.9177 481

78.9268 477

78.9358 426

78.9449 513

78.9540 460

78.9631 470

78.9722 508

78.9813 476

78.9904 495

78.9994 488

79.0085 473

79.0176 526

79.0267 476

79.0358 475

79.0449 509

79.0539 472

79.0630 502

79.0721 497

79.0812 470

79.0903 497

79.0994 483

79.1085 465

79.1175 449

79.1266 485

79.1357 485

79.1448 528

79.1539 501

79.1630 478

79.1720 463

79.1811 476

79.1902 525

79.1993 454

79.2084 488

79.2175 491

79.2266 446

79.2356 508

79.2447 511

79.2538 548

79.2629 473

79.2720 492

79.2811 493

79.2902 506

79.2992 493

79.3083 486

79.3174 462

79.3265 493

79.3356 490

79.3447 479

79.3537 496

79.3628 517

79.3719 461

79.3810 449

79.3901 525

79.3992 461

79.4083 489

79.4173 472

79.4264 467

79.4355 463

79.4446 501

79.4537 464

79.4628 527

79.4718 489

79.4809 497

79.4900 465

79.4991 456

79.5082 465

79.5173 493

79.5264 500

79.5354 498

79.5445 500

79.5536 493

79.5627 481

79.5718 459

79.5809 515

79.5900 492

79.5990 516

79.6081 504

79.6172 503

79.6263 479

79.6354 479

79.6445 480

79.6535 511

79.6626 436

79.6717 465

79.6808 498

79.6899 486

79.6990 543

79.7081 475

79.7171 467

79.7262 514

79.7353 479

79.7444 497

79.7535 429

79.7626 471

79.7716 440

79.7807 492

79.7898 521

79.7989 447

79.8080 516

79.8171 487

79.8262 507

79.8352 474

79.8443 478

79.8534 516

79.8625 495

79.8716 504

79.8807 494

79.8898 484

79.8988 525

79.9079 492

79.9170 510

79.9261 487

79.9352 515

79.9443 495

79.9533 468

79.9624 459

79.9715 484

79.9806 450

79.9897 483

79.9988 452

80.0079 498

80.0169 475

80.0260 476

80.0351 463

80.0442 470

80.0533 480

80.0624 485

80.0714 486

80.0805 525

80.0896 442

80.0987 473

80.1078 518

80.1169 482

80.1260 481

80.1350 475

80.1441 458

80.1532 509

80.1623 498

80.1714 473

80.1805 466

80.1896 537

80.1986 472

80.2077 468

80.2168 490

80.2259 463

80.2350 497

80.2441 494

80.2531 505

80.2622 457

80.2713 466

80.2804 481

80.2895 481

80.2986 490

80.3077 537

80.3167 482

80.3258 492

80.3349 477

80.3440 483

80.3531 497

80.3622 490

80.3712 450

80.3803 491

80.3894 482

80.3985 505

80.4076 462

80.4167 520

80.4258 489

80.4348 498

80.4439 456

80.4530 528

80.4621 460

80.4712 472

80.4803 473

80.4894 470

80.4984 466

80.5075 469

80.5166 443

80.5257 488

80.5348 469

80.5439 507

80.5529 466

80.5620 465

80.5711 509

80.5802 517

80.5893 489

80.5984 518

80.6075 494

80.6165 497

80.6256 461

80.6347 487

80.6438 472

80.6529 527

80.6620 509

80.6710 507

80.6801 482

80.6892 464

80.6983 506

80.7074 491

80.7165 456

80.7256 479

80.7346 501

80.7437 495

80.7528 440

80.7619 454

80.7710 478

80.7801 474

80.7892 517

80.7982 452

80.8073 506

80.8164 504

80.8255 471

80.8346 506

80.8437 486

80.8527 496

80.8618 475

80.8709 500

80.8800 539

80.8891 471

80.8982 526

80.9073 482

80.9163 489

80.9254 490

80.9345 532

80.9436 517

80.9527 480

80.9618 495

80.9708 483

80.9799 449

80.9890 478

80.9981 467

81.0072 505

81.0163 502

81.0254 508

81.0344 466

81.0435 503

81.0526 508

81.0617 482

81.0708 462

81.0799 516

81.0890 494

81.0980 455

81.1071 479

81.1162 473

81.1253 466

81.1344 449

81.1435 505

81.1525 443

81.1616 504

81.1707 489

81.1798 480

81.1889 426

81.1980 483

81.2071 483

81.2161 433

81.2252 485

81.2343 525

81.2434 534

81.2525 506

81.2616 504

81.2706 483

81.2797 493

81.2888 454

81.2979 468

81.3070 498

81.3161 469

81.3252 509

81.3342 510

81.3433 478

81.3524 463

81.3615 485

81.3706 457

81.3797 488

81.3888 483

81.3978 455

81.4069 480

81.4160 495

81.4251 443

81.4342 469

81.4433 470

81.4523 522

81.4614 490

81.4705 467

81.4796 452

81.4887 504

81.4978 484

81.5069 508

81.5159 489

81.5250 504

81.5341 488

81.5432 463

81.5523 498

81.5614 471

81.5704 498

81.5795 478

81.5886 476

81.5977 489

81.6068 475

81.6159 471

81.6250 512

81.6340 487

81.6431 494

81.6522 487

81.6613 490

81.6704 490

81.6795 478

81.6886 454

81.6976 454

81.7067 465

81.7158 469

81.7249 468

81.7340 445

81.7431 505

81.7521 467

81.7612 503

81.7703 497

81.7794 460

81.7885 454

81.7976 457

81.8067 467

81.8157 446

81.8248 460

81.8339 480

81.8430 502

81.8521 455

81.8612 487

81.8702 488

81.8793 489

81.8884 497

81.8975 490

81.9066 490

81.9157 466

81.9248 471

81.9338 503

81.9429 472

81.9520 471

81.9611 468

81.9702 489

81.9793 458

81.9884 503

81.9974 493

82.0065 489

82.0156 489

82.0247 450

82.0338 447

82.0429 479

82.0519 462

82.0610 494

82.0701 490

82.0792 451

82.0883 476

82.0974 456

82.1065 479

82.1155 462

82.1246 486

82.1337 473

82.1428 505

82.1519 478

82.1610 463

82.1700 517

82.1791 480

82.1882 468

82.1973 492

82.2064 514

82.2155 452

82.2246 474

82.2336 460

82.2427 456

82.2518 481

82.2609 480

82.2700 459

82.2791 477

82.2882 450

82.2972 492

82.3063 472

82.3154 455

82.3245 512

82.3336 490

82.3427 490

82.3517 482

82.3608 497

82.3699 476

82.3790 466

82.3881 495

82.3972 475

82.4063 495

82.4153 490

82.4244 481

82.4335 483

82.4426 449

82.4517 477

82.4608 455

82.4698 464

82.4789 445

82.4880 467

82.4971 472

82.5062 466

82.5153 480

82.5244 460

82.5334 495

82.5425 512

82.5516 478

82.5607 528

82.5698 469

82.5789 474

82.5880 484

82.5970 469

82.6061 455

82.6152 449

82.6243 455

82.6334 461

82.6425 466

82.6515 469

82.6606 484

82.6697 470

82.6788 479

82.6879 444

82.6970 471

82.7061 490

82.7151 486

82.7242 491

82.7333 437

82.7424 451

82.7515 463

82.7606 458

82.7696 514

82.7787 477

82.7878 445

82.7969 460

82.8060 455

82.8151 476

82.8242 429

82.8332 506

82.8423 502

82.8514 474

82.8605 463

82.8696 441

82.8787 452

82.8878 474

82.8968 489

82.9059 475

82.9150 460

82.9241 473

82.9332 500

82.9423 465

82.9513 495

82.9604 450

82.9695 448

82.9786 439

82.9877 478

82.9968 439

83.0059 471

83.0149 493

83.0240 471

83.0331 468

83.0422 491

83.0513 531

83.0604 534

83.0694 480

83.0785 494

83.0876 499

83.0967 459

83.1058 525

83.1149 475

83.1240 487

83.1330 467

83.1421 462

83.1512 492

83.1603 479

83.1694 491

83.1785 469

83.1876 499

83.1966 479

83.2057 459

83.2148 468

83.2239 497

83.2330 483

83.2421 491

83.2511 497

83.2602 495

83.2693 513

83.2784 496

83.2875 474

83.2966 463

83.3057 473

83.3147 454

83.3238 484

83.3329 481

83.3420 466

83.3511 455

83.3602 488

83.3692 484

83.3783 437

83.3874 493

83.3965 491

83.4056 481

83.4147 511

83.4238 466

83.4328 484

83.4419 470

83.4510 466

83.4601 474

83.4692 482

83.4783 463

83.4874 442

83.4964 487

83.5055 491

83.5146 490

83.5237 456

83.5328 450

83.5419 485

83.5509 442

83.5600 504

83.5691 450

83.5782 507

83.5873 473

83.5964 453

83.6055 451

83.6145 503

83.6236 473

83.6327 480

83.6418 473

83.6509 473

83.6600 517

83.6690 486

83.6781 465

83.6872 477

83.6963 449

83.7054 466

83.7145 454

83.7236 465

83.7326 495

83.7417 516

83.7508 463

83.7599 473

83.7690 503

83.7781 450

83.7872 469

83.7962 468

83.8053 467

83.8144 443

83.8235 449

83.8326 484

83.8417 455

83.8507 464

83.8598 471

83.8689 480

83.8780 513

83.8871 459

83.8962 414

83.9053 445

83.9143 463

83.9234 468

83.9325 479

83.9416 457

83.9507 470

83.9598 471

83.9688 434

83.9779 459

83.9870 458

83.9961 451

84.0052 477

84.0143 463

84.0234 460

84.0324 482

84.0415 485

84.0506 491

84.0597 519

84.0688 465

84.0779 491

84.0870 470

84.0960 458

84.1051 492

84.1142 463

84.1233 457

84.1324 484

84.1415 459

84.1505 468

84.1596 493

84.1687 464

84.1778 453

84.1869 463

84.1960 474

84.2051 479

84.2141 436

84.2232 497

84.2323 468

84.2414 487

84.2505 447

84.2596 473

84.2686 428

84.2777 507

84.2868 492

84.2959 512

84.3050 441

84.3141 474

84.3232 462

84.3322 491

84.3413 463

84.3504 458

84.3595 468

84.3686 469

84.3777 490

84.3868 476

84.3958 474

84.4049 481

84.4140 488

84.4231 490

84.4322 438

84.4413 486

84.4503 496

84.4594 474

84.4685 463

84.4776 461

84.4867 468

84.4958 452

84.5049 500

84.5139 460

84.5230 495

84.5321 457

84.5412 474

84.5503 464

84.5594 465

84.5684 432

84.5775 481

84.5866 499

84.5957 467

84.6048 495

84.6139 475

84.6230 477

84.6320 457

84.6411 487

84.6502 467

84.6593 496

84.6684 480

84.6775 478

84.6866 396

84.6956 443

84.7047 483

84.7138 484

84.7229 516

84.7320 464

84.7411 494

84.7501 470

84.7592 493

84.7683 460

84.7774 444

84.7865 463

84.7956 483

84.8047 463

84.8137 445

84.8228 487

84.8319 431

84.8410 473

84.8501 496

84.8592 438

84.8682 469

84.8773 459

84.8864 452

84.8955 444

84.9046 482

84.9137 459

84.9228 465

84.9318 463

84.9409 436

84.9500 474

84.9591 462

84.9682 468

84.9773 463

84.9864 501

84.9954 458

85.0045 463

85.0136 454

85.0227 485

85.0318 475

85.0409 461

85.0499 465

85.0590 471

85.0681 462

85.0772 486

85.0863 499

85.0954 477

85.1045 472

85.1135 459

85.1226 446

85.1317 486

85.1408 446

85.1499 448

85.1590 423

85.1680 456

85.1771 476

85.1862 431

85.1953 468

85.2044 464

85.2135 446

85.2226 466

85.2316 476

85.2407 477

85.2498 462

85.2589 462

85.2680 483

85.2771 464

85.2862 474

85.2952 454

85.3043 469

85.3134 442

85.3225 453

85.3316 484

85.3407 487

85.3497 450

85.3588 481

85.3679 441

85.3770 455

85.3861 485

85.3952 491

85.4043 434

85.4133 465

85.4224 459

85.4315 517

85.4406 478

85.4497 454

85.4588 463

85.4678 496

85.4769 439

85.4860 492

85.4951 478

85.5042 446

85.5133 485

85.5224 482

85.5314 429

85.5405 466

85.5496 482

85.5587 452

85.5678 459

85.5769 470

85.5860 438

85.5950 470

85.6041 457

85.6132 475

85.6223 469

85.6314 491

85.6405 463

85.6495 437

85.6586 452

85.6677 455

85.6768 485

85.6859 485

85.6950 486

85.7041 470

85.7131 429

85.7222 461

85.7313 488

85.7404 460

85.7495 461

85.7586 498

85.7676 469

85.7767 466

85.7858 433

85.7949 462

85.8040 473

85.8131 491

85.8222 442

85.8312 458

85.8403 476

85.8494 458

85.8585 451

85.8676 477

85.8767 491

85.8858 463

85.8948 427

85.9039 466

85.9130 463

85.9221 516

85.9312 460

85.9403 463

85.9493 479

85.9584 512

85.9675 438

85.9766 481

85.9857 434

85.9948 468

86.0039 458

86.0129 452

86.0220 448

86.0311 448

86.0402 479

86.0493 473

86.0584 487

86.0674 444

86.0765 467

86.0856 472

86.0947 429

86.1038 448

86.1129 462

86.1220 473

86.1310 494

86.1401 492

86.1492 459

86.1583 456

86.1674 462

86.1765 448

86.1856 466

86.1946 457

86.2037 496

86.2128 473

86.2219 455

86.2310 467

86.2401 465

86.2491 488

86.2582 500

86.2673 437

86.2764 390

86.2855 455

86.2946 486

86.3037 475

86.3127 449

86.3218 500

86.3309 446

86.3400 465

86.3491 491

86.3582 481

86.3672 414

86.3763 456

86.3854 446

86.3945 458

86.4036 437

86.4127 472

86.4218 436

86.4308 448

86.4399 480

86.4490 485

86.4581 482

86.4672 433

86.4763 466

86.4854 451

86.4944 471

86.5035 455

86.5126 433

86.5217 462

86.5308 472

86.5399 415

86.5489 450

86.5580 445

86.5671 436

86.5762 470

86.5853 459

86.5944 446

86.6035 479

86.6125 422

86.6216 449

86.6307 459

86.6398 474

86.6489 447

86.6580 508

86.6670 463

86.6761 447

86.6852 474

86.6943 469

86.7034 480

86.7125 476

86.7216 397

86.7306 496

86.7397 450

86.7488 446

86.7579 473

86.7670 477

86.7761 468

86.7852 460

86.7942 450

86.8033 444

86.8124 434

86.8215 477

86.8306 497

86.8397 433

86.8487 461

86.8578 446

86.8669 439

86.8760 439

86.8851 471

86.8942 490

86.9033 440

86.9123 460

86.9214 474

86.9305 444

86.9396 454

86.9487 462

86.9578 454

86.9668 482

86.9759 452

86.9850 458

86.9941 470

87.0032 446

87.0123 471

87.0214 467

87.0304 425

87.0395 459

87.0486 454

87.0577 468

87.0668 462

87.0759 421

87.0850 443

87.0940 455

87.1031 408

87.1122 471

87.1213 463

87.1304 451

87.1395 441

87.1485 465

87.1576 450

87.1667 466

87.1758 445

87.1849 427

87.1940 466

87.2031 478

87.2121 445

87.2212 464

87.2303 461

87.2394 435

87.2485 447

87.2576 469

87.2666 460

87.2757 444

87.2848 481

87.2939 463

87.3030 472

87.3121 440

87.3212 466

87.3302 490

87.3393 508

87.3484 520

87.3575 472

87.3666 457

87.3757 462

87.3848 457

87.3938 473

87.4029 456

87.4120 442

87.4211 441

87.4302 450

87.4393 447

87.4483 448

87.4574 443

87.4665 431

87.4756 410

87.4847 463

87.4938 480

87.5029 490

87.5119 439

87.5210 462

87.5301 439

87.5392 427

87.5483 463

87.5574 485

87.5664 449

87.5755 448

87.5846 434

87.5937 456

87.6028 460

87.6119 436

87.6210 447

87.6300 468

87.6391 459

87.6482 473

87.6573 452

87.6664 430

87.6755 464

87.6846 442

87.6936 471

87.7027 486

87.7118 489

87.7209 445

87.7300 426

87.7391 504

87.7481 464

87.7572 482

87.7663 464

87.7754 492

87.7845 457

87.7936 454

87.8027 407

87.8117 465

87.8208 473

87.8299 473

87.8390 464

87.8481 476

87.8572 423

87.8662 452

87.8753 469

87.8844 449

87.8935 464

87.9026 430

87.9117 458

87.9208 460

87.9298 443

87.9389 444

87.9480 457

87.9571 457

87.9662 449

87.9753 471

87.9844 476

87.9934 466

88.0025 441

88.0116 418

88.0207 429

88.0298 465

88.0389 445

88.0479 431

88.0570 488

88.0661 465

88.0752 500

88.0843 467

88.0934 449

88.1025 501

88.1115 442

88.1206 445

88.1297 437

88.1388 496

88.1479 488

88.1570 464

88.1660 449

88.1751 446

88.1842 459

88.1933 462

88.2024 445

88.2115 417

88.2206 474

88.2296 465

88.2387 456

88.2478 460

88.2569 466

88.2660 458

88.2751 413

88.2842 454

88.2932 481

88.3023 493

88.3114 437

88.3205 447

88.3296 469

88.3387 491

88.3477 481

88.3568 421

88.3659 465

88.3750 484

88.3841 450

88.3932 491

88.4023 469

88.4113 449

88.4204 457

88.4295 449

88.4386 457

88.4477 446

88.4568 437

88.4658 443

88.4749 468

88.4840 452

88.4931 465

88.5022 432

88.5113 456

88.5204 491

88.5294 484

88.5385 472

88.5476 459

88.5567 433

88.5658 451

88.5749 436

88.5840 478

88.5930 445

88.6021 472

88.6112 478

88.6203 443

88.6294 485

88.6385 431

88.6475 422

88.6566 464

88.6657 459

88.6748 475

88.6839 442

88.6930 474

88.7021 486

88.7111 455

88.7202 420

88.7293 462

88.7384 436

88.7475 457

88.7566 481

88.7656 472

88.7747 456

88.7838 500

88.7929 458

88.8020 449

88.8111 424

88.8202 485

88.8292 468

88.8383 439

88.8474 467

88.8565 431

88.8656 464

88.8747 481

88.8838 471

88.8928 429

88.9019 463

88.9110 453

88.9201 456

88.9292 445

88.9383 450

88.9473 436

88.9564 470

88.9655 481

88.9746 444

88.9837 453

88.9928 462

89.0019 465

89.0109 459

89.0200 470

89.0291 483

89.0382 479

89.0473 458

89.0564 424

89.0654 467

89.0745 462

89.0836 447

89.0927 422

89.1018 454

89.1109 456

89.1200 463

89.1290 484

89.1381 478

89.1472 436

89.1563 450

89.1654 468

89.1745 418

89.1836 451

89.1926 451

89.2017 443

89.2108 447

89.2199 409

89.2290 468

89.2381 455

89.2471 470

89.2562 442

89.2653 487

89.2744 484

89.2835 523

89.2926 485

89.3017 460

89.3107 413

89.3198 467

89.3289 449

89.3380 442

89.3471 435

89.3562 435

89.3652 436

89.3743 445

89.3834 459

89.3925 489

89.4016 420

89.4107 477

89.4198 446

89.4288 429

89.4379 436

89.4470 425

89.4561 464

89.4652 435

89.4743 494

89.4834 441

89.4924 456

89.5015 461

89.5106 445

89.5197 444

89.5288 476

89.5379 451

89.5469 456

89.5560 477

89.5651 442

89.5742 465

89.5833 418

89.5924 484

89.6015 425

89.6105 475

89.6196 445

89.6287 418

89.6378 446

89.6469 449

89.6560 470

89.6650 467

89.6741 450

89.6832 417

89.6923 416

89.7014 461

89.7105 432

89.7196 474

89.7286 410

89.7377 439

89.7468 456

89.7559 450

89.7650 444

89.7741 469

89.7832 446

89.7922 454

89.8013 473

89.8104 475

89.8195 437

89.8286 418

89.8377 447

89.8467 447

89.8558 442

89.8649 461

89.8740 449

89.8831 436

89.8922 407

89.9013 391

89.9103 446

89.9194 491

89.9285 455

89.9376 471

89.9467 446

89.9558 433

89.9648 415

89.9739 448

89.9830 433

89.9921 490

90.0012 473

90.0103 442

90.0194 433

90.0284 457

90.0375 430

90.0466 485

90.0557 423

90.0648 436

90.0739 440

90.0830 453

90.0920 494

90.1011 454

90.1102 468

90.1193 460

90.1284 455

90.1375 448

90.1465 442

90.1556 463

90.1647 427

90.1738 464

90.1829 435

90.1920 459

90.2011 455

90.2101 452

90.2192 442

90.2283 450

90.2374 444

90.2465 426

90.2556 464

90.2646 402

90.2737 448

90.2828 412

90.2919 446

90.3010 464

90.3101 460

90.3192 442

90.3282 466

90.3373 435

90.3464 435

90.3555 475

90.3646 424

90.3737 454

90.3828 418

90.3918 489

90.4009 446

90.4100 446

90.4191 477

90.4282 451

90.4373 431

90.4463 440

90.4554 435

90.4645 420

90.4736 446

90.4827 452

90.4918 479

90.5009 436

90.5099 439

90.5190 474

90.5281 439

90.5372 428

**Raw data 3**. XRD raw data of microsphere obtained spray pyrolysis at 700 ℃.

; (content of file C:\DATA\CBNU_IN\ENG-Chemical\JoJungSang\Oh Sehwan\171127\sample.raw)

_FILEVERSION = 2

_SAMPLE = JKI

_+SAMPLE = ZnO-GaO(ADD)

_SITE = Korea

_USER = "Chungbuk UNI."

_GONIOMETER_CODE = 21

; Goniometer : D8 theta/theta, stage : Unknown

_SAMPLE_CHANGER_CODE = 0

_ATTACHMENTS_CODE = 0

_GONIOMETER_RADIUS = 250

_FIXED_DIVSLIT = 0.6

_FIXED_SAMPLESLIT = 0

_FIXED_DETSLIT = 12.21

_MONOCHROMATOR = 0

; Incident beam monochromator : None

_THIN_FILM = N

_BETA_FILTER = N

_FIXED_ANTISLIT = 8.46

_ANALYZER_CODE = 4

; Received beam analyzer : Gobel mirror

_DATEMEASURED = "27-Nov-2017 13:10:31"

_WL_UNIT = A

_WL1 = 1.5406

_WL2 = 1.54439

_WL3 = 1.39222

_WLRATIO = 0.5

_ANODE = Cu

; Data for range 1

_DRIVE = COUPLED

_STEPTIME = 35.8

_STEPSIZE = 0.0090856

_STEPMODE = C

_START = 5

_THETA = 2.5

_2THETA = 5

_KHI = 0

_PHI = 0

_X = 0

_Y = 0

_Z = 0

_DETECTOR = 5

; Detector type : Unknown

_DETECTORSLIT = out

_AUX1 = 0

_AUX2 = 0

_AUX3 = 0

_TIMESTARTED = 12

_TEMP_RATE = -1

_TEMP_DELAY = -1

_KV = 40

_MA = 40

_RANGE_WL = 1.5406

_3DPLANE = 0

_V4_COUNTERS_MASK = 4096

_V4_DRIVES_MASK = 0

_V4_ENCODERS_MASK = 0

_2THETACOUNTS = 1

; 2THETA PSD

5.0000 0

5.0091 -0

5.0182 4

5.0273 -32

5.0363 66

5.0454 55

5.0545 64

5.0636 91

5.0727 59

5.0818 35

5.0909 89

5.0999 31

5.1090 23

5.1181 49

5.1272 131

5.1363 39

5.1454 27

5.1545 67

5.1635 37

5.1726 17

5.1817 125

5.1908 25

5.1999 135

5.2090 63

5.2181 46

5.2271 140

5.2362 68

5.2453 -16

5.2544 10

5.2635 75

5.2726 79

5.2817 80

5.2907 108

5.2998 117

5.3089 114

5.3180 11

5.3271 64

5.3362 123

5.3453 111

5.3543 84

5.3634 138

5.3725 35

5.3816 60

5.3907 97

5.3998 79

5.4089 133

5.4179 -20

5.4270 99

5.4361 122

5.4452 50

5.4543 69

5.4634 49

5.4725 144

5.4815 107

5.4906 -7

5.4997 76

5.5088 76

5.5179 63

5.5270 102

5.5361 94

5.5451 93

5.5542 70

5.5633 21

5.5724 123

5.5815 87

5.5906 7

5.5996 70

5.6087 67

5.6178 95

5.6269 77

5.6360 103

5.6451 75

5.6542 67

5.6632 104

5.6723 -2

5.6814 -11

5.6905 93

5.6996 97

5.7087 127

5.7178 30

5.7268 75

5.7359 118

5.7450 59

5.7541 85

5.7632 116

5.7723 41

5.7814 5

5.7904 114

5.7995 141

5.8086 92

5.8177 84

5.8268 3

5.8359 29

5.8450 37

5.8540 87

5.8631 87

5.8722 30

5.8813 55

5.8904 64

5.8995 51

5.9086 53

5.9176 102

5.9267 76

5.9358 93

5.9449 45

5.9540 97

5.9631 28

5.9722 39

5.9812 68

5.9903 -6

5.9994 40

6.0085 8

6.0176 1

6.0267 117

6.0358 52

6.0448 40

6.0539 36

6.0630 36

6.0721 56

6.0812 60

6.0903 29

6.0994 84

6.1084 8

6.1175 1

6.1266 -4

6.1357 77

6.1448 37

6.1539 35

6.1630 88

6.1720 60

6.1811 71

6.1902 2

6.1993 53

6.2084 103

6.2175 57

6.2266 19

6.2356 26

6.2447 -41

6.2538 61

6.2629 84

6.2720 42

6.2811 -11

6.2902 108

6.2992 48

6.3083 49

6.3174 33

6.3265 45

6.3356 55

6.3447 52

6.3538 55

6.3628 93

6.3719 50

6.3810 74

6.3901 45

6.3992 62

6.4083 44

6.4174 83

6.4264 47

6.4355 20

6.4446 53

6.4537 98

6.4628 56

6.4719 105

6.4810 45

6.4900 18

6.4991 91

6.5082 4

6.5173 39

6.5264 68

6.5355 21

6.5446 -5

6.5536 78

6.5627 99

6.5718 -45

6.5809 17

6.5900 25

6.5991 93

6.6082 140

6.6172 77

6.6263 62

6.6354 57

6.6445 88

6.6536 34

6.6627 66

6.6718 73

6.6808 128

6.6899 116

6.6990 41

6.7081 19

6.7172 56

6.7263 25

6.7353 48

6.7444 91

6.7535 45

6.7626 85

6.7717 123

6.7808 14

6.7899 124

6.7989 73

6.8080 72

6.8171 83

6.8262 111

6.8353 80

6.8444 48

6.8535 69

6.8625 85

6.8716 74

6.8807 92

6.8898 45

6.8989 48

6.9080 51

6.9171 36

6.9261 9

6.9352 34

6.9443 110

6.9534 34

6.9625 8

6.9716 72

6.9807 54

6.9897 86

6.9988 38

7.0079 38

7.0170 118

7.0261 87

7.0352 86

7.0443 103

7.0533 149

7.0624 94

7.0715 58

7.0806 57

7.0897 29

7.0988 91

7.1079 37

7.1169 80

7.1260 91

7.1351 48

7.1442 106

7.1533 122

7.1624 84

7.1715 84

7.1805 65

7.1896 32

7.1987 75

7.2078 46

7.2169 -2

7.2260 66

7.2351 47

7.2441 91

7.2532 17

7.2623 92

7.2714 104

7.2805 85

7.2896 127

7.2987 80

7.3077 57

7.3168 119

7.3259 58

7.3350 109

7.3441 84

7.3532 22

7.3623 94

7.3713 63

7.3804 68

7.3895 98

7.3986 -9

7.4077 30

7.4168 150

7.4259 90

7.4349 16

7.4440 87

7.4531 82

7.4622 54

7.4713 -1

7.4804 137

7.4895 52

7.4985 24

7.5076 48

7.5167 53

7.5258 95

7.5349 61

7.5440 48

7.5531 88

7.5621 116

7.5712 39

7.5803 97

7.5894 81

7.5985 1

7.6076 113

7.6167 21

7.6257 99

7.6348 55

7.6439 79

7.6530 18

7.6621 81

7.6712 28

7.6803 76

7.6893 -1

7.6984 61

7.7075 96

7.7166 -23

7.7257 34

7.7348 -0

7.7439 32

7.7529 7

7.7620 105

7.7711 150

7.7802 109

7.7893 36

7.7984 -0

7.8075 97

7.8165 72

7.8256 98

7.8347 17

7.8438 113

7.8529 -7

7.8620 128

7.8710 92

7.8801 43

7.8892 57

7.8983 56

7.9074 25

7.9165 76

7.9256 24

7.9346 64

7.9437 72

7.9528 81

7.9619 96

7.9710 57

7.9801 112

7.9892 31

7.9982 58

8.0073 -20

8.0164 47

8.0255 76

8.0346 1

8.0437 90

8.0528 58

8.0618 72

8.0709 89

8.0800 43

8.0891 73

8.0982 109

8.1073 92

8.1164 120

8.1254 37

8.1345 94

8.1436 116

8.1527 86

8.1618 119

8.1709 11

8.1800 140

8.1890 71

8.1981 144

8.2072 88

8.2163 77

8.2254 71

8.2345 98

8.2436 91

8.2526 74

8.2617 67

8.2708 64

8.2799 83

8.2890 68

8.2981 95

8.3072 78

8.3162 127

8.3253 28

8.3344 79

8.3435 28

8.3526 95

8.3617 -7

8.3708 65

8.3798 37

8.3889 75

8.3980 62

8.4071 16

8.4162 46

8.4253 88

8.4344 49

8.4434 54

8.4525 75

8.4616 53

8.4707 49

8.4798 96

8.4889 71

8.4980 15

8.5070 77

8.5161 116

8.5252 60

8.5343 84

8.5434 18

8.5525 11

8.5616 73

8.5706 133

8.5797 105

8.5888 39

8.5979 58

8.6070 128

8.6161 110

8.6252 119

8.6342 81

8.6433 119

8.6524 50

8.6615 54

8.6706 86

8.6797 110

8.6888 83

8.6978 129

8.7069 90

8.7160 54

8.7251 103

8.7342 88

8.7433 108

8.7524 63

8.7614 73

8.7705 141

8.7796 113

8.7887 102

8.7978 1

8.8069 69

8.8160 79

8.8250 125

8.8341 41

8.8432 113

8.8523 36

8.8614 89

8.8705 13

8.8796 31

8.8886 60

8.8977 98

8.9068 -18

8.9159 87

8.9250 53

8.9341 55

8.9432 71

8.9522 55

8.9613 87

8.9704 -7

8.9795 53

8.9886 34

8.9977 13

9.0067 82

9.0158 21

9.0249 89

9.0340 84

9.0431 56

9.0522 120

9.0613 41

9.0703 76

9.0794 52

9.0885 -13

9.0976 40

9.1067 100

9.1158 110

9.1249 72

9.1339 61

9.1430 9

9.1521 108

9.1612 73

9.1703 105

9.1794 20

9.1885 56

9.1975 104

9.2066 69

9.2157 31

9.2248 32

9.2339 43

9.2430 25

9.2521 84

9.2611 68

9.2702 74

9.2793 75

9.2884 53

9.2975 93

9.3066 103

9.3157 82

9.3247 47

9.3338 50

9.3429 116

9.3520 65

9.3611 7

9.3702 119

9.3793 60

9.3883 53

9.3974 128

9.4065 -19

9.4156 53

9.4247 30

9.4338 75

9.4429 97

9.4519 27

9.4610 76

9.4701 38

9.4792 61

9.4883 20

9.4974 101

9.5065 39

9.5155 11

9.5246 64

9.5337 65

9.5428 42

9.5519 67

9.5610 99

9.5701 66

9.5791 62

9.5882 -8

9.5973 33

9.6064 52

9.6155 177

9.6246 42

9.6337 29

9.6427 31

9.6518 89

9.6609 79

9.6700 43

9.6791 52

9.6882 61

9.6973 97

9.7063 85

9.7154 62

9.7245 52

9.7336 35

9.7427 -12

9.7518 80

9.7609 53

9.7699 41

9.7790 72

9.7881 79

9.7972 111

9.8063 59

9.8154 77

9.8245 117

9.8335 82

9.8426 48

9.8517 33

9.8608 73

9.8699 74

9.8790 66

9.8881 132

9.8971 37

9.9062 -22

9.9153 42

9.9244 86

9.9335 73

9.9426 89

9.9517 63

9.9607 76

9.9698 73

9.9789 63

9.9880 113

9.9971 56

10.0062 31

10.0153 64

10.0243 98

10.0334 138

10.0425 78

10.0516 -15

10.0607 51

10.0698 2

10.0789 -9

10.0879 65

10.0970 97

10.1061 31

10.1152 31

10.1243 28

10.1334 -11

10.1424 114

10.1515 33

10.1606 142

10.1697 45

10.1788 90

10.1879 123

10.1970 40

10.2060 109

10.2151 77

10.2242 71

10.2333 76

10.2424 130

10.2515 8

10.2606 48

10.2696 60

10.2787 23

10.2878 13

10.2969 94

10.3060 80

10.3151 32

10.3242 9

10.3332 82

10.3423 102

10.3514 55

10.3605 87

10.3696 70

10.3787 101

10.3878 61

10.3968 50

10.4059 -9

10.4150 17

10.4241 -39

10.4332 53

10.4423 123

10.4514 13

10.4604 39

10.4695 101

10.4786 103

10.4877 43

10.4968 75

10.5059 101

10.5150 27

10.5240 13

10.5331 51

10.5422 56

10.5513 155

10.5604 65

10.5695 54

10.5786 63

10.5876 15

10.5967 80

10.6058 73

10.6149 118

10.6240 55

10.6331 4

10.6422 80

10.6512 34

10.6603 46

10.6694 32

10.6785 70

10.6876 76

10.6967 88

10.7058 27

10.7148 112

10.7239 62

10.7330 75

10.7421 79

10.7512 13

10.7603 19

10.7694 41

10.7784 110

10.7875 79

10.7966 93

10.8057 36

10.8148 19

10.8239 94

10.8330 23

10.8420 56

10.8511 71

10.8602 9

10.8693 72

10.8784 115

10.8875 55

10.8966 83

10.9056 72

10.9147 17

10.9238 61

10.9329 72

10.9420 116

10.9511 31

10.9602 63

10.9692 -1

10.9783 88

10.9874 43

10.9965 54

11.0056 37

11.0147 70

11.0238 46

11.0328 44

11.0419 89

11.0510 29

11.0601 40

11.0692 26

11.0783 109

11.0874 109

11.0964 72

11.1055 42

11.1146 28

11.1237 24

11.1328 24

11.1419 -38

11.1510 123

11.1600 66

11.1691 7

11.1782 19

11.1873 27

11.1964 44

11.2055 27

11.2146 85

11.2236 98

11.2327 54

11.2418 47

11.2509 28

11.2600 32

11.2691 66

11.2781 47

11.2872 81

11.2963 57

11.3054 109

11.3145 -25

11.3236 12

11.3327 129

11.3417 13

11.3508 23

11.3599 94

11.3690 18

11.3781 33

11.3872 41

11.3963 42

11.4053 -5

11.4144 88

11.4235 85

11.4326 45

11.4417 57

11.4508 116

11.4599 63

11.4689 115

11.4780 123

11.4871 28

11.4962 34

11.5053 88

11.5144 62

11.5235 81

11.5325 35

11.5416 31

11.5507 53

11.5598 84

11.5689 47

11.5780 66

11.5871 2

11.5961 40

11.6052 37

11.6143 -8

11.6234 94

11.6325 49

11.6416 30

11.6507 43

11.6597 95

11.6688 50

11.6779 8

11.6870 93

11.6961 88

11.7052 110

11.7143 58

11.7233 12

11.7324 15

11.7415 55

11.7506 66

11.7597 102

11.7688 61

11.7779 125

11.7869 80

11.7960 27

11.8051 65

11.8142 72

11.8233 41

11.8324 22

11.8415 26

11.8505 49

11.8596 77

11.8687 32

11.8778 44

11.8869 122

11.8960 55

11.9051 39

11.9141 26

11.9232 11

11.9323 93

11.9414 44

11.9505 -3

11.9596 58

11.9687 106

11.9777 26

11.9868 13

11.9959 49

12.0050 68

12.0141 58

12.0232 97

12.0323 11

12.0413 68

12.0504 27

12.0595 61

12.0686 46

12.0777 68

12.0868 101

12.0959 39

12.1049 39

12.1140 74

12.1231 68

12.1322 21

12.1413 79

12.1504 105

12.1595 78

12.1685 -3

12.1776 47

12.1867 147

12.1958 53

12.2049 4

12.2140 55

12.2231 -2

12.2321 57

12.2412 96

12.2503 85

12.2594 50

12.2685 39

12.2776 86

12.2867 146

12.2957 37

12.3048 45

12.3139 31

12.3230 38

12.3321 45

12.3412 35

12.3503 69

12.3593 28

12.3684 65

12.3775 149

12.3866 63

12.3957 44

12.4048 -7

12.4138 21

12.4229 12

12.4320 59

12.4411 53

12.4502 37

12.4593 110

12.4684 44

12.4774 55

12.4865 116

12.4956 59

12.5047 101

12.5138 87

12.5229 70

12.5320 84

12.5410 62

12.5501 70

12.5592 92

12.5683 88

12.5774 76

12.5865 107

12.5956 60

12.6046 -17

12.6137 76

12.6228 119

12.6319 53

12.6410 0

12.6501 29

12.6592 74

12.6682 105

12.6773 61

12.6864 56

12.6955 27

12.7046 60

12.7137 129

12.7228 71

12.7318 46

12.7409 83

12.7500 162

12.7591 75

12.7682 83

12.7773 2

12.7864 45

12.7954 46

12.8045 77

12.8136 81

12.8227 10

12.8318 109

12.8409 78

12.8500 67

12.8590 15

12.8681 119

12.8772 30

12.8863 12

12.8954 110

12.9045 121

12.9136 50

12.9226 105

12.9317 46

12.9408 20

12.9499 30

12.9590 47

12.9681 16

12.9772 -19

12.9862 83

12.9953 44

13.0044 76

13.0135 8

13.0226 75

13.0317 11

13.0408 109

13.0498 -2

13.0589 43

13.0680 34

13.0771 98

13.0862 8

13.0953 18

13.1044 7

13.1134 40

13.1225 31

13.1316 -2

13.1407 59

13.1498 118

13.1589 36

13.1680 7

13.1770 -22

13.1861 115

13.1952 37

13.2043 23

13.2134 109

13.2225 67

13.2316 28

13.2406 48

13.2497 17

13.2588 93

13.2679 47

13.2770 107

13.2861 56

13.2952 30

13.3042 35

13.3133 31

13.3224 23

13.3315 50

13.3406 66

13.3497 72

13.3588 19

13.3678 50

13.3769 37

13.3860 10

13.3951 48

13.4042 32

13.4133 56

13.4224 11

13.4314 72

13.4405 58

13.4496 33

13.4587 82

13.4678 16

13.4769 -3

13.4860 69

13.4950 47

13.5041 53

13.5132 41

13.5223 29

13.5314 -13

13.5405 77

13.5495 71

13.5586 73

13.5677 107

13.5768 64

13.5859 11

13.5950 27

13.6041 47

13.6131 0

13.6222 82

13.6313 83

13.6404 84

13.6495 75

13.6586 70

13.6677 53

13.6767 38

13.6858 29

13.6949 94

13.7040 35

13.7131 47

13.7222 13

13.7313 48

13.7403 28

13.7494 98

13.7585 80

13.7676 -12

13.7767 71

13.7858 97

13.7949 26

13.8039 84

13.8130 36

13.8221 22

13.8312 80

13.8403 72

13.8494 120

13.8585 40

13.8675 132

13.8766 69

13.8857 96

13.8948 38

13.9039 59

13.9130 65

13.9221 74

13.9311 15

13.9402 128

13.9493 83

13.9584 73

13.9675 23

13.9766 -19

13.9857 111

13.9947 121

14.0038 76

14.0129 90

14.0220 46

14.0311 57

14.0402 62

14.0493 91

14.0583 33

14.0674 111

14.0765 68

14.0856 82

14.0947 80

14.1038 122

14.1129 47

14.1219 90

14.1310 62

14.1401 108

14.1492 54

14.1583 46

14.1674 56

14.1765 27

14.1855 97

14.1946 21

14.2037 40

14.2128 67

14.2219 88

14.2310 46

14.2401 78

14.2491 77

14.2582 115

14.2673 100

14.2764 49

14.2855 37

14.2946 89

14.3037 78

14.3127 35

14.3218 110

14.3309 61

14.3400 79

14.3491 106

14.3582 22

14.3673 84

14.3763 60

14.3854 88

14.3945 73

14.4036 111

14.4127 18

14.4218 40

14.4309 75

14.4399 62

14.4490 32

14.4581 -51

14.4672 66

14.4763 129

14.4854 95

14.4945 93

14.5035 44

14.5126 154

14.5217 49

14.5308 44

14.5399 11

14.5490 55

14.5581 67

14.5671 69

14.5762 97

14.5853 51

14.5944 105

14.6035 38

14.6126 105

14.6217 49

14.6307 109

14.6398 80

14.6489 73

14.6580 97

14.6671 141

14.6762 110

14.6852 75

14.6943 60

14.7034 131

14.7125 42

14.7216 26

14.7307 62

14.7398 61

14.7488 150

14.7579 78

14.7670 47

14.7761 57

14.7852 91

14.7943 66

14.8034 65

14.8124 78

14.8215 3

14.8306 59

14.8397 97

14.8488 106

14.8579 85

14.8670 61

14.8760 115

14.8851 11

14.8942 -0

14.9033 81

14.9124 70

14.9215 43

14.9306 77

14.9396 4

14.9487 85

14.9578 77

14.9669 34

14.9760 71

14.9851 19

14.9942 89

15.0032 28

15.0123 92

15.0214 112

15.0305 92

15.0396 86

15.0487 100

15.0578 70

15.0668 65

15.0759 90

15.0850 70

15.0941 95

15.1032 81

15.1123 89

15.1214 72

15.1304 61

15.1395 104

15.1486 44

15.1577 110

15.1668 56

15.1759 59

15.1850 54

15.1940 141

15.2031 71

15.2122 39

15.2213 142

15.2304 68

15.2395 117

15.2486 55

15.2576 57

15.2667 110

15.2758 86

15.2849 74

15.2940 39

15.3031 123

15.3122 80

15.3212 82

15.3303 58

15.3394 146

15.3485 92

15.3576 105

15.3667 78

15.3758 53

15.3848 108

15.3939 39

15.4030 54

15.4121 9

15.4212 67

15.4303 66

15.4394 43

15.4484 175

15.4575 92

15.4666 3

15.4757 52

15.4848 19

15.4939 109

15.5030 112

15.5120 114

15.5211 106

15.5302 46

15.5393 95

15.5484 44

15.5575 95

15.5666 76

15.5756 128

15.5847 107

15.5938 118

15.6029 136

15.6120 56

15.6211 96

15.6302 79

15.6392 95

15.6483 78

15.6574 134

15.6665 129

15.6756 99

15.6847 162

15.6938 146

15.7028 64

15.7119 110

15.7210 107

15.7301 59

15.7392 45

15.7483 124

15.7574 45

15.7664 85

15.7755 54

15.7846 116

15.7937 58

15.8028 122

15.8119 97

15.8209 58

15.8300 36

15.8391 65

15.8482 49

15.8573 85

15.8664 116

15.8755 112

15.8845 37

15.8936 104

15.9027 85

15.9118 31

15.9209 54

15.9300 45

15.9391 71

15.9481 160

15.9572 72

15.9663 111

15.9754 95

15.9845 105

15.9936 157

16.0027 57

16.0117 106

16.0208 90

16.0299 161

16.0390 47

16.0481 90

16.0572 101

16.0663 17

16.0753 112

16.0844 74

16.0935 81

16.1026 56

16.1117 48

16.1208 53

16.1299 95

16.1389 89

16.1480 65

16.1571 61

16.1662 35

16.1753 125

16.1844 50

16.1935 132

16.2025 57

16.2116 57

16.2207 89

16.2298 106

16.2389 96

16.2480 85

16.2571 77

16.2661 108

16.2752 117

16.2843 89

16.2934 101

16.3025 42

16.3116 41

16.3207 121

16.3297 80

16.3388 96

16.3479 58

16.3570 46

16.3661 158

16.3752 147

16.3843 80

16.3933 99

16.4024 90

16.4115 135

16.4206 105

16.4297 25

16.4388 122

16.4479 97

16.4569 35

16.4660 103

16.4751 122

16.4842 117

16.4933 98

16.5024 102

16.5115 110

16.5205 53

16.5296 36

16.5387 80

16.5478 123

16.5569 136

16.5660 65

16.5751 70

16.5841 183

16.5932 157

16.6023 65

16.6114 126

16.6205 118

16.6296 89

16.6387 77

16.6477 76

16.6568 132

16.6659 55

16.6750 119

16.6841 108

16.6932 90

16.7023 113

16.7113 96

16.7204 104

16.7295 82

16.7386 77

16.7477 33

16.7568 123

16.7659 139

16.7749 148

16.7840 148

16.7931 134

16.8022 104

16.8113 136

16.8204 148

16.8295 23

16.8385 56

16.8476 103

16.8567 98

16.8658 103

16.8749 101

16.8840 91

16.8931 115

16.9021 122

16.9112 129

16.9203 83

16.9294 172

16.9385 82

16.9476 158

16.9566 68

16.9657 95

16.9748 106

16.9839 59

16.9930 161

17.0021 162

17.0112 69

17.0202 120

17.0293 124

17.0384 110

17.0475 104

17.0566 103

17.0657 122

17.0748 78

17.0838 94

17.0929 86

17.1020 -3

17.1111 111

17.1202 41

17.1293 131

17.1384 143

17.1474 111

17.1565 117

17.1656 145

17.1747 118

17.1838 27

17.1929 154

17.2020 100

17.2110 97

17.2201 53

17.2292 100

17.2383 111

17.2474 133

17.2565 71

17.2656 109

17.2746 47

17.2837 68

17.2928 138

17.3019 122

17.3110 94

17.3201 118

17.3292 153

17.3382 103

17.3473 150

17.3564 87

17.3655 90

17.3746 134

17.3837 83

17.3928 79

17.4018 51

17.4109 140

17.4200 123

17.4291 124

17.4382 129

17.4473 132

17.4564 69

17.4654 116

17.4745 49

17.4836 125

17.4927 -5

17.5018 64

17.5109 118

17.5200 92

17.5290 -4

17.5381 110

17.5472 140

17.5563 67

17.5654 62

17.5745 87

17.5836 109

17.5926 112

17.6017 83

17.6108 106

17.6199 95

17.6290 138

17.6381 55

17.6472 103

17.6562 87

17.6653 104

17.6744 108

17.6835 113

17.6926 94

17.7017 98

17.7108 108

17.7198 80

17.7289 98

17.7380 116

17.7471 111

17.7562 90

17.7653 54

17.7744 81

17.7834 187

17.7925 81

17.8016 94

17.8107 84

17.8198 141

17.8289 125

17.8380 99

17.8470 123

17.8561 77

17.8652 95

17.8743 48

17.8834 87

17.8925 85

17.9016 72

17.9106 1

17.9197 65

17.9288 55

17.9379 100

17.9470 120

17.9561 91

17.9652 83

17.9742 72

17.9833 107

17.9924 86

18.0015 169

18.0106 81

18.0197 64

18.0288 67

18.0378 203

18.0469 121

18.0560 67

18.0651 61

18.0742 172

18.0833 66

18.0923 94

18.1014 33

18.1105 52

18.1196 107

18.1287 91

18.1378 57

18.1469 116

18.1559 96

18.1650 -4

18.1741 87

18.1832 80

18.1923 85

18.2014 76

18.2105 120

18.2195 35

18.2286 128

18.2377 101

18.2468 128

18.2559 117

18.2650 77

18.2741 123

18.2831 111

18.2922 133

18.3013 68

18.3104 41

18.3195 89

18.3286 87

18.3377 61

18.3467 42

18.3558 125

18.3649 117

18.3740 79

18.3831 149

18.3922 106

18.4013 32

18.4103 95

18.4194 58

18.4285 78

18.4376 98

18.4467 29

18.4558 91

18.4649 40

18.4739 114

18.4830 45

18.4921 125

18.5012 68

18.5103 110

18.5194 128

18.5285 77

18.5375 96

18.5466 76

18.5557 131

18.5648 96

18.5739 70

18.5830 48

18.5921 62

18.6011 83

18.6102 97

18.6193 141

18.6284 94

18.6375 90

18.6466 139

18.6557 28

18.6647 55

18.6738 60

18.6829 123

18.6920 70

18.7011 59

18.7102 86

18.7193 114

18.7283 100

18.7374 84

18.7465 86

18.7556 102

18.7647 61

18.7738 119

18.7829 121

18.7919 73

18.8010 157

18.8101 121

18.8192 97

18.8283 110

18.8374 95

18.8465 112

18.8555 61

18.8646 69

18.8737 58

18.8828 11

18.8919 78

18.9010 108

18.9101 119

18.9191 137

18.9282 118

18.9373 95

18.9464 61

18.9555 66

18.9646 63

18.9737 134

18.9827 77

18.9918 114

19.0009 109

19.0100 82

19.0191 109

19.0282 54

19.0373 141

19.0463 63

19.0554 97

19.0645 84

19.0736 88

19.0827 66

19.0918 92

19.1009 61

19.1099 112

19.1190 68

19.1281 121

19.1372 34

19.1463 80

19.1554 95

19.1645 108

19.1735 50

19.1826 88

19.1917 90

19.2008 122

19.2099 54

19.2190 74

19.2280 111

19.2371 96

19.2462 90

19.2553 30

19.2644 113

19.2735 99

19.2826 83

19.2916 133

19.3007 76

19.3098 44

19.3189 80

19.3280 67

19.3371 73

19.3462 96

19.3552 61

19.3643 56

19.3734 53

19.3825 23

19.3916 97

19.4007 45

19.4098 122

19.4188 78

19.4279 44

19.4370 116

19.4461 9

19.4552 84

19.4643 93

19.4734 122

19.4824 73

19.4915 97

19.5006 135

19.5097 48

19.5188 89

19.5279 104

19.5370 55

19.5460 48

19.5551 71

19.5642 34

19.5733 50

19.5824 26

19.5915 85

19.6006 37

19.6096 81

19.6187 55

19.6278 119

19.6369 27

19.6460 59

19.6551 70

19.6642 123

19.6732 31

19.6823 70

19.6914 83

19.7005 67

19.7096 95

19.7187 83

19.7278 81

19.7368 98

19.7459 39

19.7550 39

19.7641 49

19.7732 109

19.7823 83

19.7914 121

19.8004 10

19.8095 56

19.8186 88

19.8277 51

19.8368 78

19.8459 76

19.8550 154

19.8640 125

19.8731 83

19.8822 100

19.8913 -7

19.9004 71

19.9095 26

19.9186 86

19.9276 78

19.9367 67

19.9458 92

19.9549 24

19.9640 152

19.9731 91

19.9822 147

19.9912 55

20.0003 95

20.0094 89

20.0185 77

20.0276 95

20.0367 61

20.0458 69

20.0548 59

20.0639 49

20.0730 41

20.0821 115

20.0912 103

20.1003 104

20.1094 56

20.1184 112

20.1275 71

20.1366 88

20.1457 33

20.1548 44

20.1639 10

20.1730 138

20.1820 26

20.1911 36

20.2002 104

20.2093 102

20.2184 85

20.2275 85

20.2366 91

20.2456 44

20.2547 53

20.2638 54

20.2729 63

20.2820 111

20.2911 96

20.3002 106

20.3092 93

20.3183 11

20.3274 98

20.3365 92

20.3456 61

20.3547 85

20.3637 115

20.3728 107

20.3819 78

20.3910 81

20.4001 77

20.4092 91

20.4183 101

20.4273 90

20.4364 126

20.4455 68

20.4546 70

20.4637 38

20.4728 41

20.4819 65

20.4909 54

20.5000 51

20.5091 90

20.5182 152

20.5273 99

20.5364 70

20.5455 28

20.5545 74

20.5636 64

20.5727 94

20.5818 32

20.5909 54

20.6000 62

20.6091 77

20.6181 106

20.6272 69

20.6363 36

20.6454 78

20.6545 44

20.6636 90

20.6727 96

20.6817 77

20.6908 22

20.6999 57

20.7090 28

20.7181 57

20.7272 103

20.7363 78

20.7453 48

20.7544 71

20.7635 88

20.7726 71

20.7817 75

20.7908 86

20.7999 51

20.8089 55

20.8180 76

20.8271 83

20.8362 66

20.8453 66

20.8544 100

20.8635 16

20.8725 67

20.8816 71

20.8907 87

20.8998 61

20.9089 91

20.9180 116

20.9271 77

20.9361 51

20.9452 55

20.9543 121

20.9634 52

20.9725 76

20.9816 108

20.9907 100

20.9997 69

21.0088 126

21.0179 20

21.0270 131

21.0361 59

21.0452 99

21.0543 0

21.0633 30

21.0724 102

21.0815 82

21.0906 111

21.0997 71

21.1088 13

21.1179 98

21.1269 84

21.1360 82

21.1451 84

21.1542 128

21.1633 56

21.1724 54

21.1815 135

21.1905 77

21.1996 51

21.2087 127

21.2178 43

21.2269 87

21.2360 93

21.2451 25

21.2541 79

21.2632 109

21.2723 69

21.2814 105

21.2905 69

21.2996 71

21.3087 28

21.3177 88

21.3268 37

21.3359 95

21.3450 84

21.3541 36

21.3632 46

21.3723 35

21.3813 46

21.3904 71

21.3995 100

21.4086 96

21.4177 145

21.4268 76

21.4359 83

21.4449 81

21.4540 82

21.4631 101

21.4722 69

21.4813 60

21.4904 86

21.4994 20

21.5085 46

21.5176 66

21.5267 122

21.5358 16

21.5449 71

21.5540 22

21.5630 27

21.5721 73

21.5812 24

21.5903 129

21.5994 74

21.6085 85

21.6176 75

21.6266 52

21.6357 70

21.6448 69

21.6539 42

21.6630 52

21.6721 50

21.6812 65

21.6902 3

21.6993 92

21.7084 81

21.7175 92

21.7266 41

21.7357 91

21.7448 52

21.7538 47

21.7629 74

21.7720 64

21.7811 56

21.7902 124

21.7993 83

21.8084 102

21.8174 53

21.8265 45

21.8356 77

21.8447 64

21.8538 71

21.8629 78

21.8720 107

21.8810 73

21.8901 19

21.8992 61

21.9083 3

21.9174 50

21.9265 52

21.9356 52

21.9446 99

21.9537 85

21.9628 49

21.9719 41

21.9810 81

21.9901 92

21.9992 103

22.0082 13

22.0173 64

22.0264 75

22.0355 4

22.0446 18

22.0537 40

22.0628 101

22.0718 82

22.0809 59

22.0900 59

22.0991 45

22.1082 102

22.1173 40

22.1264 81

22.1354 74

22.1445 38

22.1536 -8

22.1627 102

22.1718 61

22.1809 79

22.1900 127

22.1990 73

22.2081 40

22.2172 39

22.2263 46

22.2354 63

22.2445 95

22.2536 77

22.2626 114

22.2717 29

22.2808 -19

22.2899 84

22.2990 81

22.3081 107

22.3172 7

22.3262 72

22.3353 102

22.3444 109

22.3535 112

22.3626 32

22.3717 81

22.3808 58

22.3898 61

22.3989 46

22.4080 125

22.4171 81

22.4262 41

22.4353 66

22.4444 82

22.4534 77

22.4625 98

22.4716 56

22.4807 -2

22.4898 125

22.4989 88

22.5080 101

22.5170 61

22.5261 88

22.5352 80

22.5443 152

22.5534 116

22.5625 104

22.5716 38

22.5806 69

22.5897 42

22.5988 75

22.6079 113

22.6170 51

22.6261 82

22.6351 45

22.6442 67

22.6533 11

22.6624 52

22.6715 156

22.6806 87

22.6897 56

22.6987 53

22.7078 23

22.7169 102

22.7260 67

22.7351 24

22.7442 36

22.7533 82

22.7623 40

22.7714 101

22.7805 108

22.7896 31

22.7987 53

22.8078 -26

22.8169 12

22.8259 58

22.8350 83

22.8441 108

22.8532 93

22.8623 111

22.8714 35

22.8805 100

22.8895 122

22.8986 122

22.9077 113

22.9168 93

22.9259 72

22.9350 96

22.9441 99

22.9531 120

22.9622 155

22.9713 105

22.9804 98

22.9895 50

22.9986 142

23.0077 84

23.0167 51

23.0258 71

23.0349 43

23.0440 15

23.0531 -10

23.0622 61

23.0713 130

23.0803 9

23.0894 105

23.0985 -10

23.1076 23

23.1167 17

23.1258 42

23.1349 83

23.1439 90

23.1530 31

23.1621 72

23.1712 77

23.1803 90

23.1894 125

23.1985 47

23.2075 24

23.2166 68

23.2257 100

23.2348 78

23.2439 45

23.2530 89

23.2621 32

23.2711 55

23.2802 96

23.2893 35

23.2984 84

23.3075 96

23.3166 27

23.3257 57

23.3347 67

23.3438 25

23.3529 73

23.3620 67

23.3711 96

23.3802 108

23.3893 67

23.3983 37

23.4074 61

23.4165 42

23.4256 48

23.4347 90

23.4438 18

23.4529 104

23.4619 129

23.4710 88

23.4801 122

23.4892 47

23.4983 117

23.5074 101

23.5165 65

23.5255 47

23.5346 124

23.5437 97

23.5528 15

23.5619 139

23.5710 30

23.5801 116

23.5891 117

23.5982 108

23.6073 106

23.6164 39

23.6255 35

23.6346 51

23.6437 28

23.6527 152

23.6618 67

23.6709 31

23.6800 22

23.6891 55

23.6982 32

23.7073 60

23.7163 22

23.7254 91

23.7345 61

23.7436 90

23.7527 41

23.7618 59

23.7708 84

23.7799 101

23.7890 47

23.7981 148

23.8072 69

23.8163 43

23.8254 135

23.8344 65

23.8435 99

23.8526 70

23.8617 47

23.8708 77

23.8799 85

23.8890 96

23.8980 132

23.9071 64

23.9162 69

23.9253 62

23.9344 119

23.9435 73

23.9526 48

23.9616 116

23.9707 46

23.9798 71

23.9889 44

23.9980 18

24.0071 78

24.0162 67

24.0252 68

24.0343 56

24.0434 56

24.0525 80

24.0616 105

24.0707 94

24.0798 73

24.0888 17

24.0979 80

24.1070 70

24.1161 13

24.1252 55

24.1343 60

24.1434 56

24.1524 102

24.1615 31

24.1706 74

24.1797 90

24.1888 93

24.1979 61

24.2070 94

24.2160 119

24.2251 100

24.2342 101

24.2433 115

24.2524 54

24.2615 67

24.2706 88

24.2796 56

24.2887 67

24.2978 58

24.3069 92

24.3160 66

24.3251 47

24.3342 114

24.3432 102

24.3523 30

24.3614 56

24.3705 12

24.3796 75

24.3887 78

24.3978 52

24.4068 92

24.4159 49

24.4250 110

24.4341 72

24.4432 60

24.4523 77

24.4614 49

24.4704 42

24.4795 83

24.4886 57

24.4977 85

24.5068 61

24.5159 107

24.5250 79

24.5340 84

24.5431 93

24.5522 141

24.5613 54

24.5704 86

24.5795 96

24.5886 161

24.5976 61

24.6067 102

24.6158 67

24.6249 68

24.6340 118

24.6431 62

24.6522 77

24.6612 44

24.6703 106

24.6794 76

24.6885 18

24.6976 87

24.7067 81

24.7158 88

24.7248 113

24.7339 80

24.7430 93

24.7521 99

24.7612 132

24.7703 99

24.7794 46

24.7884 140

24.7975 42

24.8066 124

24.8157 51

24.8248 85

24.8339 88

24.8430 103

24.8520 43

24.8611 78

24.8702 91

24.8793 97

24.8884 84

24.8975 95

24.9065 63

24.9156 128

24.9247 92

24.9338 84

24.9429 33

24.9520 86

24.9611 90

24.9701 83

24.9792 107

24.9883 82

24.9974 84

25.0065 123

25.0156 45

25.0247 109

25.0337 111

25.0428 110

25.0519 65

25.0610 79

25.0701 115

25.0792 112

25.0883 21

25.0973 96

25.1064 124

25.1155 100

25.1246 105

25.1337 30

25.1428 135

25.1519 20

25.1609 77

25.1700 57

25.1791 125

25.1882 85

25.1973 57

25.2064 22

25.2155 68

25.2245 72

25.2336 16

25.2427 27

25.2518 93

25.2609 113

25.2700 78

25.2791 77

25.2881 85

25.2972 67

25.3063 128

25.3154 89

25.3245 114

25.3336 78

25.3427 81

25.3517 96

25.3608 124

25.3699 59

25.3790 63

25.3881 78

25.3972 63

25.4063 69

25.4153 106

25.4244 51

25.4335 77

25.4426 109

25.4517 84

25.4608 166

25.4699 83

25.4789 117

25.4880 49

25.4971 15

25.5062 61

25.5153 97

25.5244 103

25.5335 122

25.5425 119

25.5516 131

25.5607 168

25.5698 21

25.5789 95

25.5880 114

25.5971 91

25.6061 62

25.6152 114

25.6243 101

25.6334 70

25.6425 92

25.6516 79

25.6607 88

25.6697 69

25.6788 67

25.6879 95

25.6970 42

25.7061 137

25.7152 39

25.7243 69

25.7333 81

25.7424 49

25.7515 69

25.7606 72

25.7697 71

25.7788 97

25.7879 9

25.7969 0

25.8060 34

25.8151 59

25.8242 120

25.8333 110

25.8424 120

25.8515 159

25.8605 117

25.8696 111

25.8787 111

25.8878 52

25.8969 92

25.9060 112

25.9151 111

25.9241 104

25.9332 151

25.9423 131

25.9514 79

25.9605 84

25.9696 62

25.9787 52

25.9877 62

25.9968 40

26.0059 125

26.0150 65

26.0241 91

26.0332 98

26.0422 44

26.0513 84

26.0604 81

26.0695 94

26.0786 14

26.0877 93

26.0968 118

26.1058 113

26.1149 90

26.1240 115

26.1331 69

26.1422 69

26.1513 84

26.1604 63

26.1694 97

26.1785 75

26.1876 73

26.1967 78

26.2058 161

26.2149 124

26.2240 63

26.2330 28

26.2421 85

26.2512 63

26.2603 82

26.2694 88

26.2785 99

26.2876 92

26.2966 -5

26.3057 96

26.3148 123

26.3239 57

26.3330 181

26.3421 102

26.3512 99

26.3602 141

26.3693 113

26.3784 35

26.3875 97

26.3966 78

26.4057 70

26.4148 117

26.4238 93

26.4329 84

26.4420 125

26.4511 97

26.4602 124

26.4693 88

26.4784 98

26.4874 112

26.4965 69

26.5056 86

26.5147 70

26.5238 12

26.5329 57

26.5420 88

26.5510 123

26.5601 50

26.5692 80

26.5783 68

26.5874 93

26.5965 41

26.6056 62

26.6146 63

26.6237 73

26.6328 83

26.6419 132

26.6510 85

26.6601 67

26.6692 83

26.6782 129

26.6873 89

26.6964 123

26.7055 112

26.7146 152

26.7237 34

26.7328 75

26.7418 79

26.7509 53

26.7600 99

26.7691 68

26.7782 49

26.7873 80

26.7964 119

26.8054 32

26.8145 94

26.8236 52

26.8327 89

26.8418 116

26.8509 28

26.8600 103

26.8690 47

26.8781 103

26.8872 29

26.8963 53

26.9054 84

26.9145 129

26.9236 115

26.9326 68

26.9417 55

26.9508 127

26.9599 84

26.9690 92

26.9781 83

26.9872 105

26.9962 100

27.0053 142

27.0144 92

27.0235 55

27.0326 60

27.0417 112

27.0508 117

27.0598 82

27.0689 93

27.0780 100

27.0871 19

27.0962 54

27.1053 81

27.1144 83

27.1234 117

27.1325 69

27.1416 114

27.1507 98

27.1598 66

27.1689 47

27.1779 100

27.1870 30

27.1961 79

27.2052 131

27.2143 80

27.2234 79

27.2325 72

27.2415 30

27.2506 72

27.2597 11

27.2688 55

27.2779 105

27.2870 102

27.2961 21

27.3051 32

27.3142 112

27.3233 30

27.3324 119

27.3415 84

27.3506 94

27.3597 132

27.3687 68

27.3778 89

27.3869 52

27.3960 143

27.4051 107

27.4142 71

27.4233 123

27.4323 62

27.4414 115

27.4505 78

27.4596 46

27.4687 49

27.4778 152

27.4869 103

27.4959 105

27.5050 55

27.5141 66

27.5232 130

27.5323 66

27.5414 83

27.5505 103

27.5595 63

27.5686 68

27.5777 75

27.5868 43

27.5959 123

27.6050 78

27.6141 59

27.6231 78

27.6322 46

27.6413 111

27.6504 67

27.6595 30

27.6686 56

27.6777 72

27.6867 108

27.6958 101

27.7049 102

27.7140 39

27.7231 116

27.7322 39

27.7413 107

27.7503 40

27.7594 44

27.7685 100

27.7776 77

27.7867 63

27.7958 19

27.8049 80

27.8139 153

27.8230 57

27.8321 121

27.8412 105

27.8503 63

27.8594 126

27.8685 79

27.8775 16

27.8866 49

27.8957 108

27.9048 85

27.9139 96

27.9230 53

27.9321 48

27.9411 168

27.9502 106

27.9593 105

27.9684 83

27.9775 74

27.9866 105

27.9957 51

28.0047 112

28.0138 48

28.0229 27

28.0320 70

28.0411 89

28.0502 124

28.0593 58

28.0683 88

28.0774 59

28.0865 57

28.0956 39

28.1047 101

28.1138 75

28.1229 51

28.1319 118

28.1410 110

28.1501 142

28.1592 52

28.1683 4

28.1774 95

28.1865 85

28.1955 82

28.2046 95

28.2137 65

28.2228 72

28.2319 33

28.2410 138

28.2501 69

28.2591 103

28.2682 88

28.2773 75

28.2864 80

28.2955 39

28.3046 43

28.3136 105

28.3227 87

28.3318 97

28.3409 41

28.3500 98

28.3591 91

28.3682 -14

28.3772 38

28.3863 82

28.3954 112

28.4045 29

28.4136 57

28.4227 26

28.4318 67

28.4408 92

28.4499 65

28.4590 86

28.4681 50

28.4772 41

28.4863 37

28.4954 94

28.5044 84

28.5135 109

28.5226 82

28.5317 53

28.5408 127

28.5499 32

28.5590 134

28.5680 56

28.5771 135

28.5862 115

28.5953 139

28.6044 88

28.6135 115

28.6226 105

28.6316 71

28.6407 7

28.6498 68

28.6589 76

28.6680 50

28.6771 91

28.6862 69

28.6952 68

28.7043 -4

28.7134 48

28.7225 78

28.7316 58

28.7407 23

28.7498 61

28.7588 20

28.7679 141

28.7770 109

28.7861 14

28.7952 21

28.8043 8

28.8134 89

28.8224 103

28.8315 109

28.8406 96

28.8497 48

28.8588 73

28.8679 74

28.8770 113

28.8860 96

28.8951 84

28.9042 108

28.9133 68

28.9224 69

28.9315 95

28.9406 62

28.9496 56

28.9587 49

28.9678 110

28.9769 104

28.9860 96

28.9951 129

29.0042 120

29.0132 100

29.0223 106

29.0314 110

29.0405 47

29.0496 119

29.0587 69

29.0678 70

29.0768 101

29.0859 28

29.0950 60

29.1041 73

29.1132 137

29.1223 76

29.1314 59

29.1404 5

29.1495 83

29.1586 128

29.1677 79

29.1768 39

29.1859 77

29.1950 49

29.2040 94

29.2131 5

29.2222 82

29.2313 88

29.2404 18

29.2495 96

29.2586 72

29.2676 82

29.2767 78

29.2858 106

29.2949 59

29.3040 84

29.3131 81

29.3222 71

29.3312 66

29.3403 31

29.3494 49

29.3585 83

29.3676 108

29.3767 33

29.3858 73

29.3948 62

29.4039 71

29.4130 71

29.4221 78

29.4312 78

29.4403 79

29.4493 108

29.4584 124

29.4675 37

29.4766 125

29.4857 60

29.4948 54

29.5039 64

29.5129 77

29.5220 55

29.5311 67

29.5402 79

29.5493 77

29.5584 29

29.5675 110

29.5765 125

29.5856 110

29.5947 51

29.6038 26

29.6129 44

29.6220 58

29.6311 47

29.6401 42

29.6492 93

29.6583 73

29.6674 67

29.6765 117

29.6856 103

29.6947 96

29.7037 55

29.7128 12

29.7219 70

29.7310 134

29.7401 76

29.7492 75

29.7583 72

29.7673 73

29.7764 65

29.7855 54

29.7946 63

29.8037 72

29.8128 73

29.8219 76

29.8309 108

29.8400 45

29.8491 85

29.8582 90

29.8673 44

29.8764 111

29.8855 63

29.8945 78

29.9036 46

29.9127 98

29.9218 77

29.9309 101

29.9400 120

29.9491 82

29.9581 82

29.9672 53

29.9763 57

29.9854 122

29.9945 57

30.0036 55

30.0127 45

30.0217 41

30.0308 173

30.0399 120

30.0490 71

30.0581 80

30.0672 119

30.0763 33

30.0853 72

30.0944 88

30.1035 40

30.1126 25

30.1217 110

30.1308 51

30.1399 82

30.1489 32

30.1580 48

30.1671 122

30.1762 48

30.1853 65

30.1944 75

30.2035 120

30.2125 67

30.2216 68

30.2307 115

30.2398 118

30.2489 96

30.2580 47

30.2671 90

30.2761 112

30.2852 32

30.2943 33

30.3034 102

30.3125 69

30.3216 95

30.3307 83

30.3397 137

30.3488 77

30.3579 98

30.3670 29

30.3761 71

30.3852 100

30.3943 18

30.4033 84

30.4124 86

30.4215 75

30.4306 125

30.4397 70

30.4488 86

30.4579 45

30.4669 31

30.4760 50

30.4851 7

30.4942 65

30.5033 22

30.5124 45

30.5215 57

30.5305 47

30.5396 123

30.5487 86

30.5578 88

30.5669 109

30.5760 66

30.5850 122

30.5941 38

30.6032 78

30.6123 41

30.6214 22

30.6305 27

30.6396 49

30.6486 196

30.6577 5

30.6668 94

30.6759 70

30.6850 20

30.6941 121

30.7032 57

30.7122 110

30.7213 32

30.7304 40

30.7395 66

30.7486 35

30.7577 97

30.7668 75

30.7758 80

30.7849 80

30.7940 118

30.8031 61

30.8122 65

30.8213 62

30.8304 85

30.8394 6

30.8485 56

30.8576 107

30.8667 69

30.8758 0

30.8849 130

30.8940 88

30.9030 107

30.9121 74

30.9212 139

30.9303 119

30.9394 14

30.9485 75

30.9576 122

30.9666 53

30.9757 91

30.9848 123

30.9939 75

31.0030 111

31.0121 8

31.0212 112

31.0302 61

31.0393 43

31.0484 93

31.0575 41

31.0666 55

31.0757 72

31.0848 54

31.0938 85

31.1029 101

31.1120 71

31.1211 110

31.1302 33

31.1393 43

31.1484 105

31.1574 33

31.1665 93

31.1756 74

31.1847 49

31.1938 43

31.2029 42

31.2120 106

31.2210 52

31.2301 16

31.2392 112

31.2483 89

31.2574 69

31.2665 131

31.2756 105

31.2846 95

31.2937 50

31.3028 57

31.3119 144

31.3210 66

31.3301 91

31.3392 10

31.3482 94

31.3573 45

31.3664 37

31.3755 28

31.3846 95

31.3937 110

31.4028 151

31.4118 19

31.4209 35

31.4300 73

31.4391 108

31.4482 65

31.4573 13

31.4664 2

31.4754 114

31.4845 103

31.4936 68

31.5027 71

31.5118 40

31.5209 104

31.5300 61

31.5390 90

31.5481 84

31.5572 140

31.5663 90

31.5754 81

31.5845 99

31.5936 103

31.6026 95

31.6117 120

31.6208 42

31.6299 44

31.6390 101

31.6481 38

31.6572 97

31.6662 29

31.6753 129

31.6844 81

31.6935 28

31.7026 74

31.7117 61

31.7207 107

31.7298 111

31.7389 28

31.7480 101

31.7571 67

31.7662 26

31.7753 71

31.7843 34

31.7934 80

31.8025 12

31.8116 100

31.8207 72

31.8298 76

31.8389 76

31.8479 76

31.8570 98

31.8661 137

31.8752 65

31.8843 97

31.8934 71

31.9025 87

31.9115 41

31.9206 89

31.9297 83

31.9388 74

31.9479 128

31.9570 88

31.9661 59

31.9751 92

31.9842 94

31.9933 54

32.0024 -1

32.0115 14

32.0206 96

32.0297 107

32.0387 74

32.0478 17

32.0569 73

32.0660 62

32.0751 62

32.0842 77

32.0933 15

32.1023 95

32.1114 66

32.1205 50

32.1296 127

32.1387 52

32.1478 55

32.1569 68

32.1659 47

32.1750 140

32.1841 161

32.1932 94

32.2023 122

32.2114 71

32.2205 116

32.2295 147

32.2386 111

32.2477 109

32.2568 130

32.2659 96

32.2750 77

32.2841 63

32.2931 99

32.3022 66

32.3113 59

32.3204 60

32.3295 40

32.3386 125

32.3477 130

32.3567 88

32.3658 98

32.3749 40

32.3840 48

32.3931 134

32.4022 104

32.4113 65

32.4203 106

32.4294 51

32.4385 53

32.4476 85

32.4567 67

32.4658 75

32.4749 88

32.4839 109

32.4930 65

32.5021 80

32.5112 84

32.5203 82

32.5294 39

32.5385 85

32.5475 18

32.5566 62

32.5657 73

32.5748 64

32.5839 101

32.5930 83

32.6021 89

32.6111 60

32.6202 66

32.6293 56

32.6384 18

32.6475 96

32.6566 62

32.6657 91

32.6747 79

32.6838 35

32.6929 99

32.7020 46

32.7111 119

32.7202 69

32.7293 43

32.7383 144

32.7474 53

32.7565 59

32.7656 64

32.7747 142

32.7838 111

32.7929 100

32.8019 117

32.8110 90

32.8201 91

32.8292 124

32.8383 84

32.8474 60

32.8564 44

32.8655 113

32.8746 60

32.8837 71

32.8928 61

32.9019 127

32.9110 51

32.9200 86

32.9291 43

32.9382 49

32.9473 63

32.9564 83

32.9655 141

32.9746 67

32.9836 103

32.9927 104

33.0018 24

33.0109 36

33.0200 113

33.0291 43

33.0382 76

33.0472 80

33.0563 92

33.0654 44

33.0745 107

33.0836 74

33.0927 92

33.1018 -6

33.1108 82

33.1199 59

33.1290 104

33.1381 89

33.1472 34

33.1563 83

33.1654 51

33.1744 78

33.1835 154

33.1926 91

33.2017 107

33.2108 17

33.2199 84

33.2290 75

33.2380 57

33.2471 52

33.2562 103

33.2653 54

33.2744 96

33.2835 113

33.2926 110

33.3016 28

33.3107 60

33.3198 68

33.3289 147

33.3380 104

33.3471 123

33.3562 99

33.3652 61

33.3743 66

33.3834 99

33.3925 38

33.4016 30

33.4107 79

33.4198 90

33.4288 70

33.4379 142

33.4470 -0

33.4561 51

33.4652 27

33.4743 66

33.4834 91

33.4924 84

33.5015 128

33.5106 52

33.5197 77

33.5288 20

33.5379 106

33.5470 33

33.5560 68

33.5651 93

33.5742 96

33.5833 84

33.5924 34

33.6015 78

33.6106 96

33.6196 21

33.6287 118

33.6378 99

33.6469 44

33.6560 161

33.6651 68

33.6742 60

33.6832 49

33.6923 35

33.7014 93

33.7105 46

33.7196 72

33.7287 95

33.7378 64

33.7468 97

33.7559 99

33.7650 106

33.7741 38

33.7832 76

33.7923 85

33.8014 73

33.8104 62

33.8195 109

33.8286 144

33.8377 47

33.8468 86

33.8559 84

33.8650 135

33.8740 18

33.8831 114

33.8922 116

33.9013 85

33.9104 114

33.9195 63

33.9286 83

33.9376 55

33.9467 91

33.9558 39

33.9649 51

33.9740 53

33.9831 89

33.9921 15

34.0012 105

34.0103 110

34.0194 42

34.0285 52

34.0376 51

34.0467 69

34.0557 84

34.0648 113

34.0739 68

34.0830 99

34.0921 81

34.1012 94

34.1103 101

34.1193 89

34.1284 55

34.1375 46

34.1466 27

34.1557 74

34.1648 82

34.1739 92

34.1829 90

34.1920 98

34.2011 40

34.2102 107

34.2193 122

34.2284 75

34.2375 109

34.2465 69

34.2556 79

34.2647 93

34.2738 91

34.2829 102

34.2920 28

34.3011 50

34.3101 87

34.3192 134

34.3283 118

34.3374 60

34.3465 102

34.3556 99

34.3647 30

34.3737 39

34.3828 58

34.3919 45

34.4010 121

34.4101 93

34.4192 49

34.4283 25

34.4373 31

34.4464 60

34.4555 15

34.4646 87

34.4737 91

34.4828 89

34.4919 46

34.5009 26

34.5100 14

34.5191 -3

34.5282 81

34.5373 95

34.5464 97

34.5555 -13

34.5645 81

34.5736 97

34.5827 30

34.5918 129

34.6009 142

34.6100 94

34.6191 29

34.6281 120

34.6372 101

34.6463 81

34.6554 58

34.6645 40

34.6736 135

34.6827 97

34.6917 149

34.7008 103

34.7099 170

34.7190 47

34.7281 62

34.7372 73

34.7463 65

34.7553 46

34.7644 109

34.7735 65

34.7826 141

34.7917 105

34.8008 92

34.8099 63

34.8189 62

34.8280 60

34.8371 48

34.8462 124

34.8553 121

34.8644 1

34.8735 8

34.8825 44

34.8916 40

34.9007 101

34.9098 69

34.9189 57

34.9280 84

34.9371 66

34.9461 78

34.9552 95

34.9643 102

34.9734 146

34.9825 84

34.9916 104

35.0007 68

35.0097 90

35.0188 49

35.0279 76

35.0370 117

35.0461 62

35.0552 63

35.0643 83

35.0733 60

35.0824 71

35.0915 113

35.1006 62

35.1097 139

35.1188 54

35.1278 107

35.1369 76

35.1460 57

35.1551 140

35.1642 92

35.1733 106

35.1824 78

35.1914 107

35.2005 87

35.2096 63

35.2187 67

35.2278 147

35.2369 114

35.2460 106

35.2550 28

35.2641 196

35.2732 101

35.2823 157

35.2914 101

35.3005 89

35.3096 118

35.3186 120

35.3277 96

35.3368 114

35.3459 77

35.3550 94

35.3641 62

35.3732 134

35.3822 71

35.3913 92

35.4004 135

35.4095 122

35.4186 84

35.4277 106

35.4368 63

35.4458 105

35.4549 102

35.4640 47

35.4731 91

35.4822 186

35.4913 64

35.5004 74

35.5094 124

35.5185 98

35.5276 49

35.5367 130

35.5458 81

35.5549 138

35.5640 144

35.5730 102

35.5821 100

35.5912 94

35.6003 57

35.6094 64

35.6185 70

35.6276 80

35.6366 110

35.6457 65

35.6548 124

35.6639 105

35.6730 86

35.6821 141

35.6912 148

35.7002 131

35.7093 147

35.7184 117

35.7275 143

35.7366 81

35.7457 123

35.7548 108

35.7638 57

35.7729 149

35.7820 54

35.7911 118

35.8002 124

35.8093 130

35.8184 96

35.8274 85

35.8365 138

35.8456 112

35.8547 120

35.8638 99

35.8729 118

35.8820 109

35.8910 174

35.9001 131

35.9092 82

35.9183 178

35.9274 82

35.9365 118

35.9456 107

35.9546 69

35.9637 117

35.9728 138

35.9819 119

35.9910 102

36.0001 119

36.0092 72

36.0182 27

36.0273 153

36.0364 148

36.0455 130

36.0546 114

36.0637 110

36.0728 128

36.0818 182

36.0909 158

36.1000 102

36.1091 159

36.1182 71

36.1273 53

36.1364 152

36.1454 142

36.1545 131

36.1636 187

36.1727 121

36.1818 156

36.1909 139

36.2000 143

36.2090 138

36.2181 87

36.2272 143

36.2363 142

36.2454 160

36.2545 131

36.2635 90

36.2726 193

36.2817 180

36.2908 167

36.2999 148

36.3090 176

36.3181 129

36.3271 197

36.3362 175

36.3453 190

36.3544 181

36.3635 195

36.3726 152

36.3817 110

36.3907 123

36.3998 217

36.4089 214

36.4180 197

36.4271 160

36.4362 167

36.4453 188

36.4543 151

36.4634 180

36.4725 175

36.4816 215

36.4907 167

36.4998 142

36.5089 189

36.5179 168

36.5270 208

36.5361 201

36.5452 169

36.5543 209

36.5634 187

36.5725 228

36.5815 191

36.5906 195

36.5997 235

36.6088 238

36.6179 182

36.6270 276

36.6361 218

36.6451 172

36.6542 128

36.6633 200

36.6724 181

36.6815 248

36.6906 259

36.6997 217

36.7087 246

36.7178 216

36.7269 218

36.7360 233

36.7451 222

36.7542 197

36.7633 259

36.7723 228

36.7814 194

36.7905 248

36.7996 219

36.8087 224

36.8178 214

36.8269 256

36.8359 271

36.8450 184

36.8541 219

36.8632 196

36.8723 252

36.8814 129

36.8905 202

36.8995 158

36.9086 235

36.9177 239

36.9268 243

36.9359 194

36.9450 107

36.9541 178

36.9631 173

36.9722 227

36.9813 134

36.9904 166

36.9995 165

37.0086 256

37.0177 154

37.0267 181

37.0358 214

37.0449 199

37.0540 178

37.0631 221

37.0722 155

37.0813 145

37.0903 184

37.0994 219

37.1085 168

37.1176 131

37.1267 201

37.1358 246

37.1449 160

37.1539 194

37.1630 177

37.1721 159

37.1812 146

37.1903 124

37.1994 131

37.2085 130

37.2175 141

37.2266 177

37.2357 165

37.2448 65

37.2539 159

37.2630 167

37.2721 162

37.2811 195

37.2902 87

37.2993 183

37.3084 168

37.3175 110

37.3266 51

37.3357 109

37.3447 144

37.3538 153

37.3629 123

37.3720 105

37.3811 82

37.3902 149

37.3992 182

37.4083 140

37.4174 78

37.4265 110

37.4356 76

37.4447 127

37.4538 166

37.4628 158

37.4719 84

37.4810 111

37.4901 144

37.4992 74

37.5083 123

37.5174 126

37.5264 72

37.5355 70

37.5446 120

37.5537 91

37.5628 72

37.5719 86

37.5810 48

37.5900 31

37.5991 112

37.6082 164

37.6173 114

37.6264 117

37.6355 160

37.6446 52

37.6536 160

37.6627 158

37.6718 80

37.6809 96

37.6900 161

37.6991 176

37.7082 96

37.7172 105

37.7263 69

37.7354 30

37.7445 102

37.7536 125

37.7627 159

37.7718 88

37.7808 110

37.7899 101

37.7990 102

37.8081 102

37.8172 80

37.8263 97

37.8354 117

37.8444 99

37.8535 83

37.8626 117

37.8717 96

37.8808 95

37.8899 38

37.8990 128

37.9080 59

37.9171 85

37.9262 167

37.9353 135

37.9444 73

37.9535 112

37.9626 78

37.9716 72

37.9807 58

37.9898 53

37.9989 98

38.0080 52

38.0171 121

38.0262 50

38.0352 106

38.0443 102

38.0534 88

38.0625 61

38.0716 102

38.0807 77

38.0898 84

38.0988 123

38.1079 114

38.1170 91

38.1261 46

38.1352 113

38.1443 94

38.1534 95

38.1624 112

38.1715 79

38.1806 94

38.1897 118

38.1988 121

38.2079 84

38.2170 48

38.2260 166

38.2351 17

38.2442 82

38.2533 69

38.2624 34

38.2715 90

38.2806 133

38.2896 124

38.2987 103

38.3078 119

38.3169 112

38.3260 41

38.3351 117

38.3442 107

38.3532 131

38.3623 146

38.3714 47

38.3805 71

38.3896 151

38.3987 90

38.4078 52

38.4168 129

38.4259 118

38.4350 93

38.4441 137

38.4532 74

38.4623 102

38.4714 91

38.4804 65

38.4895 71

38.4986 61

38.5077 66

38.5168 58

38.5259 110

38.5349 10

38.5440 53

38.5531 61

38.5622 121

38.5713 85

38.5804 132

38.5895 154

38.5985 84

38.6076 174

38.6167 5

38.6258 89

38.6349 84

38.6440 116

38.6531 71

38.6621 63

38.6712 93

38.6803 122

38.6894 16

38.6985 97

38.7076 140

38.7167 76

38.7257 105

38.7348 98

38.7439 65

38.7530 44

38.7621 42

38.7712 79

38.7803 56

38.7893 47

38.7984 58

38.8075 99

38.8166 94

38.8257 118

38.8348 67

38.8439 100

38.8529 47

38.8620 83

38.8711 135

38.8802 91

38.8893 80

38.8984 92

38.9075 90

38.9165 63

38.9256 135

38.9347 119

38.9438 65

38.9529 105

38.9620 65

38.9711 90

38.9801 91

38.9892 64

38.9983 132

39.0074 91

39.0165 108

39.0256 129

39.0347 111

39.0437 80

39.0528 124

39.0619 123

39.0710 98

39.0801 86

39.0892 100

39.0983 100

39.1073 65

39.1164 123

39.1255 94

39.1346 141

39.1437 137

39.1528 170

39.1619 117

39.1709 101

39.1800 103

39.1891 26

39.1982 116

39.2073 135

39.2164 64

39.2255 52

39.2345 111

39.2436 112

39.2527 83

39.2618 81

39.2709 93

39.2800 87

39.2891 106

39.2981 37

39.3072 83

39.3163 65

39.3254 161

39.3345 69

39.3436 81

39.3527 34

39.3617 121

39.3708 117

39.3799 120

39.3890 37

39.3981 105

39.4072 107

39.4163 78

39.4253 112

39.4344 58

39.4435 69

39.4526 92

39.4617 88

39.4708 87

39.4799 125

39.4889 97

39.4980 79

39.5071 159

39.5162 135

39.5253 84

39.5344 64

39.5435 112

39.5525 53

39.5616 85

39.5707 64

39.5798 77

39.5889 41

39.5980 92

39.6071 63

39.6161 124

39.6252 70

39.6343 96

39.6434 54

39.6525 26

39.6616 131

39.6706 53

39.6797 104

39.6888 73

39.6979 88

39.7070 102

39.7161 110

39.7252 84

39.7342 111

39.7433 63

39.7524 68

39.7615 99

39.7706 79

39.7797 84

39.7888 69

39.7978 19

39.8069 95

39.8160 103

39.8251 137

39.8342 139

39.8433 62

39.8524 85

39.8614 84

39.8705 84

39.8796 118

39.8887 127

39.8978 58

39.9069 52

39.9160 128

39.9250 87

39.9341 88

39.9432 102

39.9523 99

39.9614 109

39.9705 87

39.9796 51

39.9886 110

39.9977 152

40.0068 66

40.0159 73

40.0250 49

40.0341 49

40.0432 105

40.0522 113

40.0613 89

40.0704 63

40.0795 130

40.0886 114

40.0977 107

40.1068 93

40.1158 94

40.1249 116

40.1340 101

40.1431 132

40.1522 122

40.1613 105

40.1704 112

40.1794 54

40.1885 105

40.1976 73

40.2067 41

40.2158 95

40.2249 88

40.2340 133

40.2430 63

40.2521 77

40.2612 149

40.2703 116

40.2794 45

40.2885 90

40.2976 108

40.3066 129

40.3157 113

40.3248 97

40.3339 114

40.3430 103

40.3521 76

40.3612 87

40.3702 133

40.3793 91

40.3884 107

40.3975 15

40.4066 142

40.4157 119

40.4248 50

40.4338 103

40.4429 78

40.4520 157

40.4611 80

40.4702 44

40.4793 135

40.4884 86

40.4974 75

40.5065 76

40.5156 92

40.5247 123

40.5338 133

40.5429 66

40.5520 70

40.5610 103

40.5701 107

40.5792 152

40.5883 46

40.5974 66

40.6065 97

40.6156 26

40.6246 127

40.6337 60

40.6428 92

40.6519 89

40.6610 73

40.6701 71

40.6792 89

40.6882 137

40.6973 149

40.7064 72

40.7155 155

40.7246 89

40.7337 91

40.7428 115

40.7518 67

40.7609 138

40.7700 90

40.7791 153

40.7882 94

40.7973 121

40.8063 27

40.8154 87

40.8245 61

40.8336 168

40.8427 53

40.8518 95

40.8609 126

40.8699 54

40.8790 195

40.8881 115

40.8972 121

40.9063 136

40.9154 117

40.9245 154

40.9335 128

40.9426 86

40.9517 126

40.9608 59

40.9699 91

40.9790 91

40.9881 67

40.9971 54

41.0062 95

41.0153 131

41.0244 163

41.0335 85

41.0426 83

41.0517 99

41.0607 111

41.0698 109

41.0789 124

41.0880 145

41.0971 87

41.1062 100

41.1153 121

41.1243 85

41.1334 116

41.1425 120

41.1516 187

41.1607 102

41.1698 101

41.1789 79

41.1879 99

41.1970 98

41.2061 92

41.2152 74

41.2243 150

41.2334 80

41.2425 104

41.2515 122

41.2606 72

41.2697 110

41.2788 153

41.2879 118

41.2970 121

41.3061 97

41.3151 83

41.3242 145

41.3333 143

41.3424 99

41.3515 149

41.3606 173

41.3697 172

41.3787 136

41.3878 90

41.3969 122

41.4060 123

41.4151 102

41.4242 129

41.4333 94

41.4423 111

41.4514 113

41.4605 156

41.4696 60

41.4787 143

41.4878 139

41.4969 147

41.5059 148

41.5150 79

41.5241 147

41.5332 119

41.5423 176

41.5514 143

41.5605 84

41.5695 154

41.5786 94

41.5877 107

41.5968 153

41.6059 153

41.6150 59

41.6241 97

41.6331 142

41.6422 143

41.6513 162

41.6604 108

41.6695 168

41.6786 158

41.6877 185

41.6967 132

41.7058 155

41.7149 132

41.7240 133

41.7331 105

41.7422 148

41.7513 66

41.7603 182

41.7694 151

41.7785 193

41.7876 186

41.7967 174

41.8058 155

41.8149 183

41.8239 153

41.8330 136

41.8421 157

41.8512 121

41.8603 190

41.8694 181

41.8785 136

41.8875 182

41.8966 89

41.9057 169

41.9148 157

41.9239 183

41.9330 189

41.9420 215

41.9511 164

41.9602 170

41.9693 152

41.9784 179

41.9875 116

41.9966 211

42.0056 214

42.0147 173

42.0238 186

42.0329 180

42.0420 190

42.0511 153

42.0602 183

42.0692 196

42.0783 219

42.0874 196

42.0965 233

42.1056 157

42.1147 156

42.1238 167

42.1328 212

42.1419 168

42.1510 93

42.1601 258

42.1692 193

42.1783 173

42.1874 196

42.1964 185

42.2055 205

42.2146 122

42.2237 186

42.2328 215

42.2419 176

42.2510 176

42.2600 189

42.2691 322

42.2782 255

42.2873 161

42.2964 215

42.3055 232

42.3146 243

42.3236 218

42.3327 237

42.3418 245

42.3509 209

42.3600 299

42.3691 227

42.3782 227

42.3872 289

42.3963 248

42.4054 251

42.4145 281

42.4236 233

42.4327 208

42.4418 245

42.4508 308

42.4599 278

42.4690 296

42.4781 288

42.4872 273

42.4963 285

42.5054 329

42.5144 268

42.5235 317

42.5326 333

42.5417 259

42.5508 358

42.5599 352

42.5690 318

42.5780 339

42.5871 315

42.5962 270

42.6053 314

42.6144 281

42.6235 368

42.6326 238

42.6416 348

42.6507 358

42.6598 358

42.6689 313

42.6780 351

42.6871 250

42.6962 345

42.7052 308

42.7143 250

42.7234 321

42.7325 330

42.7416 373

42.7507 352

42.7598 365

42.7688 384

42.7779 246

42.7870 326

42.7961 271

42.8052 270

42.8143 362

42.8234 322

42.8324 264

42.8415 264

42.8506 293

42.8597 281

42.8688 269

42.8779 354

42.8870 228

42.8960 296

42.9051 229

42.9142 315

42.9233 304

42.9324 270

42.9415 215

42.9506 277

42.9596 251

42.9687 235

42.9778 245

42.9869 221

42.9960 306

43.0051 264

43.0142 173

43.0232 186

43.0323 292

43.0414 247

43.0505 261

43.0596 296

43.0687 238

43.0777 274

43.0868 210

43.0959 223

43.1050 240

43.1141 218

43.1232 173

43.1323 204

43.1413 234

43.1504 183

43.1595 179

43.1686 259

43.1777 243

43.1868 269

43.1959 229

43.2049 175

43.2140 226

43.2231 223

43.2322 202

43.2413 259

43.2504 187

43.2595 220

43.2685 203

43.2776 221

43.2867 255

43.2958 164

43.3049 197

43.3140 220

43.3231 199

43.3321 232

43.3412 221

43.3503 214

43.3594 172

43.3685 188

43.3776 200

43.3867 193

43.3957 139

43.4048 181

43.4139 176

43.4230 218

43.4321 137

43.4412 174

43.4503 125

43.4593 190

43.4684 234

43.4775 171

43.4866 114

43.4957 75

43.5048 211

43.5139 169

43.5229 233

43.5320 97

43.5411 262

43.5502 198

43.5593 149

43.5684 112

43.5775 120

43.5865 143

43.5956 118

43.6047 167

43.6138 147

43.6229 209

43.6320 177

43.6411 120

43.6501 204

43.6592 212

43.6683 183

43.6774 158

43.6865 158

43.6956 189

43.7047 155

43.7137 107

43.7228 116

43.7319 191

43.7410 139

43.7501 114

43.7592 87

43.7683 112

43.7773 182

43.7864 110

43.7955 190

43.8046 152

43.8137 81

43.8228 72

43.8319 151

43.8409 172

43.8500 190

43.8591 162

43.8682 194

43.8773 159

43.8864 170

43.8955 131

43.9045 178

43.9136 168

43.9227 143

43.9318 169

43.9409 119

43.9500 140

43.9591 107

43.9681 130

43.9772 127

43.9863 164

43.9954 176

44.0045 184

44.0136 146

44.0227 162

44.0317 192

44.0408 118

44.0499 125

44.0590 202

44.0681 123

44.0772 136

44.0863 118

44.0953 155

44.1044 157

44.1135 146

44.1226 151

44.1317 116

44.1408 192

44.1499 202

44.1589 147

44.1680 157

44.1771 132

44.1862 173

44.1953 129

44.2044 89

44.2134 90

44.2225 159

44.2316 112

44.2407 186

44.2498 138

44.2589 165

44.2680 126

44.2770 114

44.2861 72

44.2952 104

44.3043 95

44.3134 150

44.3225 184

44.3316 81

44.3406 118

44.3497 153

44.3588 107

44.3679 95

44.3770 65

44.3861 166

44.3952 133

44.4042 150

44.4133 116

44.4224 138

44.4315 172

44.4406 112

44.4497 68

44.4588 217

44.4678 188

44.4769 150

44.4860 130

44.4951 32

44.5042 102

44.5133 120

44.5224 115

44.5314 144

44.5405 57

44.5496 114

44.5587 121

44.5678 63

44.5769 99

44.5860 171

44.5950 109

44.6041 118

44.6132 69

44.6223 110

44.6314 162

44.6405 167

44.6496 89

44.6586 77

44.6677 159

44.6768 80

44.6859 135

44.6950 186

44.7041 41

44.7132 93

44.7222 161

44.7313 187

44.7404 130

44.7495 68

44.7586 119

44.7677 101

44.7768 91

44.7858 148

44.7949 126

44.8040 122

44.8131 58

44.8222 74

44.8313 180

44.8404 120

44.8494 82

44.8585 134

44.8676 111

44.8767 63

44.8858 99

44.8949 130

44.9040 66

44.9130 84

44.9221 147

44.9312 112

44.9403 121

44.9494 135

44.9585 131

44.9676 91

44.9766 99

44.9857 139

44.9948 108

45.0039 81

45.0130 59

45.0221 128

45.0312 102

45.0402 102

45.0493 91

45.0584 47

45.0675 105

45.0766 162

45.0857 73

45.0948 93

45.1038 135

45.1129 131

45.1220 74

45.1311 81

45.1402 122

45.1493 78

45.1584 85

45.1674 16

45.1765 98

45.1856 67

45.1947 126

45.2038 130

45.2129 127

45.2220 73

45.2310 120

45.2401 59

45.2492 67

45.2583 67

45.2674 158

45.2765 113

45.2856 154

45.2946 82

45.3037 150

45.3128 126

45.3219 155

45.3310 125

45.3401 150

45.3491 136

45.3582 101

45.3673 113

45.3764 81

45.3855 123

45.3946 44

45.4037 82

45.4127 128

45.4218 111

45.4309 58

45.4400 167

45.4491 137

45.4582 89

45.4673 71

45.4763 164

45.4854 173

45.4945 87

45.5036 61

45.5127 142

45.5218 74

45.5309 91

45.5399 123

45.5490 75

45.5581 72

45.5672 114

45.5763 119

45.5854 68

45.5945 161

45.6035 84

45.6126 99

45.6217 128

45.6308 99

45.6399 62

45.6490 81

45.6581 99

45.6671 107

45.6762 100

45.6853 129

45.6944 57

45.7035 162

45.7126 143

45.7217 124

45.7307 85

45.7398 148

45.7489 112

45.7580 130

45.7671 53

45.7762 60

45.7853 127

45.7943 129

45.8034 81

45.8125 134

45.8216 85

45.8307 107

45.8398 88

45.8489 146

45.8579 77

45.8670 148

45.8761 124

45.8852 74

45.8943 51

45.9034 71

45.9125 171

45.9215 110

45.9306 54

45.9397 91

45.9488 89

45.9579 85

45.9670 159

45.9761 90

45.9851 73

45.9942 109

46.0033 157

46.0124 120

46.0215 81

46.0306 145

46.0397 95

46.0487 93

46.0578 123

46.0669 147

46.0760 110

46.0851 65

46.0942 100

46.1033 16

46.1123 74

46.1214 99

46.1305 128

46.1396 76

46.1487 143

46.1578 156

46.1669 141

46.1759 90

46.1850 100

46.1941 89

46.2032 102

46.2123 159

46.2214 76

46.2305 82

46.2395 62

46.2486 47

46.2577 148

46.2668 105

46.2759 130

46.2850 119

46.2941 130

46.3031 101

46.3122 28

46.3213 92

46.3304 102

46.3395 145

46.3486 135

46.3577 132

46.3667 138

46.3758 59

46.3849 81

46.3940 80

46.4031 106

46.4122 108

46.4213 104

46.4303 82

46.4394 120

46.4485 118

46.4576 128

46.4667 136

46.4758 140

46.4848 88

46.4939 101

46.5030 82

46.5121 44

46.5212 85

46.5303 188

46.5394 55

46.5484 125

46.5575 76

46.5666 56

46.5757 159

46.5848 67

46.5939 49

46.6030 98

46.6120 79

46.6211 173

46.6302 73

46.6393 146

46.6484 81

46.6575 87

46.6666 75

46.6756 145

46.6847 67

46.6938 101

46.7029 91

46.7120 119

46.7211 99

46.7302 192

46.7392 134

46.7483 109

46.7574 150

46.7665 105

46.7756 121

46.7847 158

46.7938 98

46.8028 75

46.8119 135

46.8210 146

46.8301 89

46.8392 46

46.8483 64

46.8574 88

46.8664 98

46.8755 102

46.8846 67

46.8937 77

46.9028 58

46.9119 123

46.9210 98

46.9300 152

46.9391 111

46.9482 99

46.9573 68

46.9664 179

46.9755 126

46.9846 60

46.9936 88

47.0027 154

47.0118 152

47.0209 93

47.0300 71

47.0391 53

47.0482 60

47.0572 85

47.0663 100

47.0754 128

47.0845 110

47.0936 173

47.1027 141

47.1118 85

47.1208 133

47.1299 162

47.1390 101

47.1481 96

47.1572 107

47.1663 74

47.1754 153

47.1844 116

47.1935 113

47.2026 122

47.2117 138

47.2208 81

47.2299 76

47.2390 58

47.2480 73

47.2571 56

47.2662 154

47.2753 83

47.2844 81

47.2935 157

47.3026 49

47.3116 67

47.3207 63

47.3298 126

47.3389 77

47.3480 95

47.3571 79

47.3662 103

47.3752 126

47.3843 119

47.3934 97

47.4025 114

47.4116 145

47.4207 77

47.4298 118

47.4388 121

47.4479 134

47.4570 114

47.4661 125

47.4752 28

47.4843 97

47.4934 154

47.5024 48

47.5115 126

47.5206 58

47.5297 54

47.5388 64

47.5479 99

47.5570 122

47.5660 127

47.5751 18

47.5842 117

47.5933 78

47.6024 57

47.6115 117

47.6205 102

47.6296 90

47.6387 138

47.6478 107

47.6569 73

47.6660 136

47.6751 81

47.6841 123

47.6932 69

47.7023 88

47.7114 70

47.7205 90

47.7296 106

47.7387 124

47.7477 107

47.7568 101

47.7659 123

47.7750 73

47.7841 109

47.7932 111

47.8023 90

47.8113 142

47.8204 134

47.8295 55

47.8386 116

47.8477 102

47.8568 115

47.8659 33

47.8749 58

47.8840 111

47.8931 90

47.9022 60

47.9113 115

47.9204 127

47.9295 146

47.9385 111

47.9476 49

47.9567 94

47.9658 118

47.9749 121

47.9840 92

47.9931 104

48.0021 85

48.0112 70

48.0203 107

48.0294 97

48.0385 58

48.0476 156

48.0567 72

48.0657 30

48.0748 95

48.0839 150

48.0930 69

48.1021 87

48.1112 79

48.1203 132

48.1293 64

48.1384 126

48.1475 74

48.1566 100

48.1657 80

48.1748 90

48.1839 136

48.1929 92

48.2020 163

48.2111 127

48.2202 98

48.2293 100

48.2384 111

48.2475 90

48.2565 177

48.2656 121

48.2747 85

48.2838 104

48.2929 83

48.3020 83

48.3111 65

48.3201 90

48.3292 75

48.3383 91

48.3474 110

48.3565 79

48.3656 102

48.3747 39

48.3837 131

48.3928 104

48.4019 74

48.4110 59

48.4201 167

48.4292 149

48.4383 121

48.4473 154

48.4564 61

48.4655 103

48.4746 129

48.4837 123

48.4928 98

48.5019 113

48.5109 124

48.5200 102

48.5291 14

48.5382 133

48.5473 81

48.5564 78

48.5655 121

48.5745 72

48.5836 37

48.5927 100

48.6018 122

48.6109 95

48.6200 123

48.6291 143

48.6381 100

48.6472 152

48.6563 66

48.6654 126

48.6745 87

48.6836 92

48.6927 119

48.7017 82

48.7108 65

48.7199 70

48.7290 174

48.7381 121

48.7472 72

48.7562 119

48.7653 185

48.7744 124

48.7835 125

48.7926 87

48.8017 62

48.8108 118

48.8198 122

48.8289 76

48.8380 83

48.8471 152

48.8562 60

48.8653 106

48.8744 61

48.8834 108

48.8925 45

48.9016 77

48.9107 98

48.9198 97

48.9289 95

48.9380 93

48.9470 156

48.9561 103

48.9652 93

48.9743 138

48.9834 127

48.9925 120

49.0016 135

49.0106 173

49.0197 122

49.0288 97

49.0379 104

49.0470 104

49.0561 108

49.0652 83

49.0742 138

49.0833 108

49.0924 69

49.1015 100

49.1106 131

49.1197 105

49.1288 120

49.1378 93

49.1469 90

49.1560 134

49.1651 98

49.1742 82

49.1833 91

49.1924 133

49.2014 76

49.2105 133

49.2196 125

49.2287 161

49.2378 82

49.2469 96

49.2560 153

49.2650 99

49.2741 68

49.2832 165

49.2923 76

49.3014 133

49.3105 123

49.3196 118

49.3286 145

49.3377 73

49.3468 75

49.3559 117

49.3650 180

49.3741 111

49.3832 104

49.3922 62

49.4013 85

49.4104 60

49.4195 97

49.4286 91

49.4377 130

49.4468 109

49.4558 106

49.4649 129

49.4740 140

49.4831 52

49.4922 114

49.5013 115

49.5104 61

49.5194 136

49.5285 123

49.5376 23

49.5467 33

49.5558 112

49.5649 108

49.5740 95

49.5830 116

49.5921 17

49.6012 84

49.6103 108

49.6194 148

49.6285 66

49.6376 129

49.6466 160

49.6557 78

49.6648 101

49.6739 92

49.6830 106

49.6921 139

49.7012 138

49.7102 58

49.7193 105

49.7284 128

49.7375 137

49.7466 94

49.7557 46

49.7648 64

49.7738 102

49.7829 72

49.7920 48

49.8011 158

49.8102 92

49.8193 87

49.8284 77

49.8374 37

49.8465 197

49.8556 88

49.8647 70

49.8738 93

49.8829 129

49.8919 90

49.9010 90

49.9101 146

49.9192 84

49.9283 129

49.9374 55

49.9465 49

49.9555 69

49.9646 175

49.9737 147

49.9828 111

49.9919 51

50.0010 62

50.0101 50

50.0191 108

50.0282 71

50.0373 129

50.0464 106

50.0555 138

50.0646 50

50.0737 92

50.0827 93

50.0918 128

50.1009 77

50.1100 122

50.1191 106

50.1282 124

50.1373 81

50.1463 112

50.1554 92

50.1645 130

50.1736 83

50.1827 80

50.1918 6

50.2009 74

50.2099 82

50.2190 103

50.2281 95

50.2372 62

50.2463 112

50.2554 143

50.2645 95

50.2735 48

50.2826 142

50.2917 98

50.3008 110

50.3099 51

50.3190 176

50.3281 69

50.3371 61

50.3462 97

50.3553 53

50.3644 80

50.3735 65

50.3826 107

50.3917 85

50.4007 87

50.4098 68

50.4189 59

50.4280 163

50.4371 133

50.4462 71

50.4553 83

50.4643 136

50.4734 75

50.4825 93

50.4916 72

50.5007 116

50.5098 113

50.5189 103

50.5279 119

50.5370 88

50.5461 37

50.5552 137

50.5643 114

50.5734 181

50.5825 59

50.5915 102

50.6006 73

50.6097 136

50.6188 208

50.6279 116

50.6370 121

50.6461 110

50.6551 63

50.6642 50

50.6733 115

50.6824 24

50.6915 69

50.7006 131

50.7097 82

50.7187 36

50.7278 115

50.7369 52

50.7460 62

50.7551 81

50.7642 110

50.7733 139

50.7823 131

50.7914 36

50.8005 62

50.8096 55

50.8187 83

50.8278 136

50.8369 121

50.8459 102

50.8550 84

50.8641 122

50.8732 10

50.8823 114

50.8914 74

50.9005 66

50.9095 67

50.9186 67

50.9277 112

50.9368 119

50.9459 39

50.9550 149

50.9641 129

50.9731 117

50.9822 84

50.9913 126

51.0004 93

51.0095 114

51.0186 151

51.0276 112

51.0367 139

51.0458 71

51.0549 159

51.0640 84

51.0731 78

51.0822 110

51.0912 171

51.1003 179

51.1094 111

51.1185 96

51.1276 84

51.1367 62

51.1458 168

51.1548 60

51.1639 85

51.1730 67

51.1821 85

51.1912 93

51.2003 132

51.2094 90

51.2184 77

51.2275 123

51.2366 138

51.2457 96

51.2548 60

51.2639 112

51.2730 154

51.2820 50

51.2911 169

51.3002 86

51.3093 97

51.3184 139

51.3275 63

51.3366 166

51.3456 95

51.3547 82

51.3638 144

51.3729 93

51.3820 69

51.3911 76

51.4002 71

51.4092 155

51.4183 42

51.4274 125

51.4365 61

51.4456 86

51.4547 130

51.4638 51

51.4728 91

51.4819 134

51.4910 105

51.5001 90

51.5092 133

51.5183 44

51.5274 124

51.5364 108

51.5455 73

51.5546 100

51.5637 106

51.5728 118

51.5819 152

51.5910 137

51.6000 117

51.6091 104

51.6182 144

51.6273 89

51.6364 96

51.6455 83

51.6546 112

51.6636 133

51.6727 111

51.6818 171

51.6909 81

51.7000 71

51.7091 74

51.7182 69

51.7272 88

51.7363 106

51.7454 133

51.7545 73

51.7636 69

51.7727 85

51.7818 82

51.7908 126

51.7999 79

51.8090 110

51.8181 134

51.8272 85

51.8363 59

51.8454 128

51.8544 46

51.8635 73

51.8726 122

51.8817 -2

51.8908 155

51.8999 87

51.9090 121

51.9180 66

51.9271 104

51.9362 129

51.9453 64

51.9544 74

51.9635 104

51.9726 52

51.9816 86

51.9907 137

51.9998 101

52.0089 29

52.0180 106

52.0271 122

52.0362 93

52.0452 134

52.0543 84

52.0634 105

52.0725 13

52.0816 104

52.0907 70

52.0998 73

52.1088 73

52.1179 79

52.1270 169

52.1361 91

52.1452 42

52.1543 70

52.1633 25

52.1724 74

52.1815 97

52.1906 84

52.1997 32

52.2088 48

52.2179 123

52.2269 140

52.2360 89

52.2451 101

52.2542 111

52.2633 19

52.2724 57

52.2815 150

52.2905 112

52.2996 106

52.3087 126

52.3178 118

52.3269 123

52.3360 92

52.3451 100

52.3541 89

52.3632 110

52.3723 61

52.3814 143

52.3905 51

52.3996 163

52.4087 117

52.4177 64

52.4268 115

52.4359 84

52.4450 152

52.4541 87

52.4632 64

52.4723 94

52.4813 72

52.4904 91

52.4995 83

52.5086 84

52.5177 55

52.5268 96

52.5359 113

52.5449 49

52.5540 55

52.5631 118

52.5722 144

52.5813 80

52.5904 107

52.5995 119

52.6085 28

52.6176 145

52.6267 124

52.6358 129

52.6449 110

52.6540 86

52.6631 118

52.6721 105

52.6812 165

52.6903 57

52.6994 59

52.7085 91

52.7176 84

52.7267 124

52.7357 104

52.7448 67

52.7539 101

52.7630 111

52.7721 83

52.7812 56

52.7903 130

52.7993 126

52.8084 119

52.8175 75

52.8266 105

52.8357 73

52.8448 43

52.8539 141

52.8629 96

52.8720 55

52.8811 58

52.8902 85

52.8993 77

52.9084 86

52.9175 26

52.9265 68

52.9356 93

52.9447 65

52.9538 68

52.9629 68

52.9720 59

52.9811 143

52.9901 45

52.9992 149

53.0083 85

53.0174 72

53.0265 115

53.0356 84

53.0447 151

53.0537 93

53.0628 93

53.0719 110

53.0810 83

53.0901 88

53.0992 27

53.1083 85

53.1173 78

53.1264 57

53.1355 86

53.1446 105

53.1537 103

53.1628 107

53.1719 138

53.1809 76

53.1900 59

53.1991 122

53.2082 104

53.2173 96

53.2264 112

53.2355 101

53.2445 124

53.2536 136

53.2627 109

53.2718 111

53.2809 101

53.2900 165

53.2990 109

53.3081 125

53.3172 66

53.3263 115

53.3354 64

53.3445 53

53.3536 101

53.3626 129

53.3717 80

53.3808 137

53.3899 98

53.3990 99

53.4081 59

53.4172 100

53.4262 69

53.4353 59

53.4444 140

53.4535 89

53.4626 117

53.4717 39

53.4808 66

53.4898 182

53.4989 87

53.5080 102

53.5171 90

53.5262 87

53.5353 94

53.5444 103

53.5534 131

53.5625 78

53.5716 141

53.5807 148

53.5898 80

53.5989 36

53.6080 102

53.6170 59

53.6261 60

53.6352 103

53.6443 98

53.6534 46

53.6625 61

53.6716 48

53.6806 89

53.6897 121

53.6988 75

53.7079 70

53.7170 68

53.7261 54

53.7352 77

53.7442 68

53.7533 109

53.7624 91

53.7715 98

53.7806 48

53.7897 100

53.7988 177

53.8078 153

53.8169 87

53.8260 70

53.8351 48

53.8442 52

53.8533 98

53.8624 107

53.8714 140

53.8805 101

53.8896 64

53.8987 125

53.9078 72

53.9169 78

53.9260 88

53.9350 84

53.9441 85

53.9532 103

53.9623 135

53.9714 65

53.9805 70

53.9896 113

53.9986 95

54.0077 117

54.0168 75

54.0259 109

54.0350 71

54.0441 77

54.0532 53

54.0622 118

54.0713 41

54.0804 65

54.0895 72

54.0986 75

54.1077 30

54.1168 101

54.1258 48

54.1349 72

54.1440 105

54.1531 33

54.1622 167

54.1713 44

54.1804 82

54.1894 127

54.1985 101

54.2076 100

54.2167 103

54.2258 121

54.2349 53

54.2440 101

54.2530 76

54.2621 51

54.2712 86

54.2803 55

54.2894 107

54.2985 64

54.3076 110

54.3166 129

54.3257 14

54.3348 109

54.3439 149

54.3530 133

54.3621 107

54.3712 78

54.3802 59

54.3893 90

54.3984 72

54.4075 59

54.4166 81

54.4257 69

54.4347 127

54.4438 87

54.4529 76

54.4620 112

54.4711 59

54.4802 83

54.4893 98

54.4983 36

54.5074 55

54.5165 96

54.5256 107

54.5347 87

54.5438 157

54.5529 108

54.5619 104

54.5710 67

54.5801 82

54.5892 96

54.5983 66

54.6074 168

54.6165 70

54.6255 117

54.6346 91

54.6437 47

54.6528 96

54.6619 125

54.6710 67

54.6801 41

54.6891 82

54.6982 89

54.7073 66

54.7164 88

54.7255 84

54.7346 148

54.7437 68

54.7527 168

54.7618 136

54.7709 96

54.7800 84

54.7891 63

54.7982 103

54.8073 96

54.8163 87

54.8254 59

54.8345 66

54.8436 95

54.8527 28

54.8618 101

54.8709 157

54.8799 101

54.8890 -6

54.8981 96

54.9072 99

54.9163 131

54.9254 96

54.9345 51

54.9435 97

54.9526 73

54.9617 128

54.9708 135

54.9799 34

54.9890 105

54.9981 40

55.0071 75

55.0162 96

55.0253 59

55.0344 83

55.0435 91

55.0526 123

55.0617 58

55.0707 55

55.0798 71

55.0889 99

55.0980 111

55.1071 23

55.1162 69

55.1253 65

55.1343 69

55.1434 118

55.1525 112

55.1616 51

55.1707 52

55.1798 77

55.1889 106

55.1979 71

55.2070 158

55.2161 89

55.2252 129

55.2343 83

55.2434 147

55.2525 128

55.2615 64

55.2706 52

55.2797 76

55.2888 140

55.2979 136

55.3070 57

55.3161 43

55.3251 103

55.3342 154

55.3433 31

55.3524 85

55.3615 68

55.3706 60

55.3797 109

55.3887 68

55.3978 61

55.4069 108

55.4160 80

55.4251 85

55.4342 111

55.4433 83

55.4523 114

55.4614 79

55.4705 66

55.4796 109

55.4887 32

55.4978 111

55.5069 108

55.5159 94

55.5250 78

55.5341 156

55.5432 102

55.5523 90

55.5614 53

55.5704 83

55.5795 71

55.5886 64

55.5977 112

55.6068 129

55.6159 33

55.6250 50

55.6340 86

55.6431 84

55.6522 90

55.6613 46

55.6704 85

55.6795 95

55.6886 57

55.6976 78

55.7067 76

55.7158 57

55.7249 112

55.7340 92

55.7431 101

55.7522 84

55.7612 48

55.7703 91

55.7794 133

55.7885 88

55.7976 153

55.8067 85

55.8158 87

55.8248 106

55.8339 93

55.8430 128

55.8521 27

55.8612 124

55.8703 115

55.8794 117

55.8884 44

55.8975 53

55.9066 102

55.9157 93

55.9248 27

55.9339 49

55.9430 94

55.9520 155

55.9611 115

55.9702 85

55.9793 102

55.9884 76

55.9975 146

56.0066 107

56.0156 66

56.0247 124

56.0338 136

56.0429 94

56.0520 68

56.0611 84

56.0702 123

56.0792 72

56.0883 67

56.0974 97

56.1065 129

56.1156 96

56.1247 67

56.1338 117

56.1428 103

56.1519 152

56.1610 95

56.1701 77

56.1792 105

56.1883 52

56.1974 86

56.2064 62

56.2155 100

56.2246 70

56.2337 54

56.2428 46

56.2519 49

56.2610 95

56.2700 111

56.2791 105

56.2882 80

56.2973 103

56.3064 97

56.3155 71

56.3246 131

56.3336 149

56.3427 110

56.3518 61

56.3609 80

56.3700 108

56.3791 67

56.3882 72

56.3972 112

56.4063 154

56.4154 88

56.4245 97

56.4336 50

56.4427 44

56.4518 19

56.4608 166

56.4699 97

56.4790 73

56.4881 97

56.4972 53

56.5063 139

56.5154 96

56.5244 105

56.5335 112

56.5426 102

56.5517 94

56.5608 107

56.5699 56

56.5790 98

56.5880 80

56.5971 77

56.6062 127

56.6153 134

56.6244 56

56.6335 71

56.6426 139

56.6516 71

56.6607 148

56.6698 156

56.6789 148

56.6880 71

56.6971 87

56.7061 123

56.7152 97

56.7243 88

56.7334 34

56.7425 69

56.7516 65

56.7607 76

56.7697 80

56.7788 107

56.7879 62

56.7970 107

56.8061 75

56.8152 74

56.8243 118

56.8333 123

56.8424 77

56.8515 96

56.8606 77

56.8697 71

56.8788 156

56.8879 82

56.8969 23

56.9060 78

56.9151 88

56.9242 96

56.9333 85

56.9424 87

56.9515 70

56.9605 121

56.9696 42

56.9787 144

56.9878 87

56.9969 91

57.0060 103

57.0151 23

57.0241 74

57.0332 75

57.0423 82

57.0514 133

57.0605 58

57.0696 66

57.0787 73

57.0877 92

57.0968 92

57.1059 50

57.1150 31

57.1241 94

57.1332 86

57.1423 99

57.1513 30

57.1604 80

57.1695 93

57.1786 90

57.1877 84

57.1968 122

57.2059 114

57.2149 83

57.2240 98

57.2331 109

57.2422 100

57.2513 96

57.2604 35

57.2695 92

57.2785 78

57.2876 71

57.2967 74

57.3058 88

57.3149 52

57.3240 138

57.3331 39

57.3421 125

57.3512 95

57.3603 158

57.3694 100

57.3785 89

57.3876 61

57.3967 140

57.4057 150

57.4148 85

57.4239 75

57.4330 110

57.4421 152

57.4512 49

57.4603 97

57.4693 111

57.4784 81

57.4875 104

57.4966 85

57.5057 107

57.5148 82

57.5239 58

57.5329 12

57.5420 66

57.5511 89

57.5602 73

57.5693 79

57.5784 109

57.5875 116

57.5965 60

57.6056 103

57.6147 135

57.6238 103

57.6329 121

57.6420 62

57.6511 33

57.6601 53

57.6692 70

57.6783 74

57.6874 74

57.6965 71

57.7056 130

57.7147 83

57.7237 143

57.7328 86

57.7419 110

57.7510 152

57.7601 104

57.7692 149

57.7783 104

57.7873 121

57.7964 85

57.8055 64

57.8146 45

57.8237 122

57.8328 101

57.8418 127

57.8509 91

57.8600 70

57.8691 99

57.8782 54

57.8873 71

57.8964 65

57.9054 84

57.9145 54

57.9236 57

57.9327 55

57.9418 90

57.9509 104

57.9600 70

57.9690 80

57.9781 116

57.9872 104

57.9963 70

58.0054 54

58.0145 46

58.0236 103

58.0326 121

58.0417 134

58.0508 74

58.0599 71

58.0690 69

58.0781 72

58.0872 70

58.0962 68

58.1053 80

58.1144 82

58.1235 69

58.1326 111

58.1417 118

58.1508 125

58.1598 104

58.1689 94

58.1780 66

58.1871 146

58.1962 72

58.2053 77

58.2144 63

58.2234 132

58.2325 99

58.2416 67

58.2507 165

58.2598 40

58.2689 60

58.2780 147

58.2870 67

58.2961 62

58.3052 112

58.3143 40

58.3234 36

58.3325 123

58.3416 64

58.3506 88

58.3597 96

58.3688 42

58.3779 107

58.3870 89

58.3961 116

58.4052 60

58.4142 73

58.4233 82

58.4324 74

58.4415 50

58.4506 104

58.4597 84

58.4688 77

58.4778 141

58.4869 118

58.4960 127

58.5051 41

58.5142 98

58.5233 59

58.5324 31

58.5414 125

58.5505 71

58.5596 102

58.5687 139

58.5778 115

58.5869 94

58.5960 119

58.6050 65

58.6141 97

58.6232 82

58.6323 73

58.6414 97

58.6505 147

58.6596 116

58.6686 70

58.6777 79

58.6868 85

58.6959 93

58.7050 138

58.7141 42

58.7232 95

58.7322 48

58.7413 106

58.7504 70

58.7595 119

58.7686 74

58.7777 58

58.7868 102

58.7958 123

58.8049 110

58.8140 44

58.8231 71

58.8322 15

58.8413 121

58.8504 59

58.8594 159

58.8685 136

58.8776 84

58.8867 93

58.8958 47

58.9049 156

58.9140 82

58.9230 95

58.9321 16

58.9412 78

58.9503 80

58.9594 49

58.9685 154

58.9775 99

58.9866 132

58.9957 86

59.0048 37

59.0139 63

59.0230 43

59.0321 110

59.0411 77

59.0502 132

59.0593 76

59.0684 95

59.0775 122

59.0866 97

59.0957 85

59.1047 63

59.1138 125

59.1229 17

59.1320 94

59.1411 46

59.1502 56

59.1593 55

59.1683 66

59.1774 81

59.1865 107

59.1956 67

59.2047 117

59.2138 96

59.2229 56

59.2319 58

59.2410 81

59.2501 103

59.2592 117

59.2683 63

59.2774 69

59.2865 83

59.2955 132

59.3046 83

59.3137 70

59.3228 92

59.3319 78

59.3410 69

59.3501 89

59.3591 97

59.3682 102

59.3773 77

59.3864 105

59.3955 114

59.4046 44

59.4137 133

59.4227 78

59.4318 98

59.4409 59

59.4500 81

59.4591 75

59.4682 102

59.4773 81

59.4863 145

59.4954 92

59.5045 67

59.5136 73

59.5227 145

59.5318 74

59.5409 112

59.5499 12

59.5590 97

59.5681 105

59.5772 79

59.5863 80

59.5954 61

59.6045 82

59.6135 75

59.6226 57

59.6317 109

59.6408 91

59.6499 6

59.6590 126

59.6681 131

59.6771 47

59.6862 133

59.6953 106

59.7044 146

59.7135 129

59.7226 81

59.7317 135

59.7407 68

59.7498 37

59.7589 69

59.7680 74

59.7771 163

59.7862 133

59.7953 68

59.8043 66

59.8134 135

59.8225 111

59.8316 87

59.8407 83

59.8498 156

59.8589 110

59.8679 85

59.8770 88

59.8861 87

59.8952 111

59.9043 105

59.9134 112

59.9225 42

59.9315 129

59.9406 118

59.9497 111

59.9588 136

59.9679 63

59.9770 113

59.9861 57

59.9951 98

60.0042 70

60.0133 82

60.0224 73

60.0315 55

60.0406 100

60.0497 148

60.0587 74

60.0678 112

60.0769 129

60.0860 102

60.0951 102

60.1042 94

60.1132 108

60.1223 114

60.1314 38

60.1405 130

60.1496 72

60.1587 121

60.1678 77

60.1768 122

60.1859 155

60.1950 83

60.2041 172

60.2132 84

60.2223 159

60.2314 126

60.2404 57

60.2495 69

60.2586 38

60.2677 117

60.2768 100

60.2859 67

60.2950 118

60.3040 55

60.3131 85

60.3222 85

60.3313 130

60.3404 112

60.3495 106

60.3586 68

60.3676 101

60.3767 125

60.3858 111

60.3949 123

60.4040 87

60.4131 182

60.4222 99

60.4312 82

60.4403 72

60.4494 76

60.4585 106

60.4676 148

60.4767 63

60.4858 117

60.4948 110

60.5039 141

60.5130 112

60.5221 111

60.5312 106

60.5403 81

60.5494 97

60.5584 105

60.5675 93

60.5766 71

60.5857 146

60.5948 99

60.6039 115

60.6130 93

60.6220 118

60.6311 113

60.6402 132

60.6493 135

60.6584 48

60.6675 117

60.6766 109

60.6856 115

60.6947 112

60.7038 96

60.7129 117

60.7220 102

60.7311 118

60.7402 117

60.7492 89

60.7583 190

60.7674 102

60.7765 92

60.7856 144

60.7947 108

60.8038 108

60.8128 76

60.8219 94

60.8310 113

60.8401 118

60.8492 88

60.8583 140

60.8674 92

60.8764 117

60.8855 136

60.8946 112

60.9037 65

60.9128 89

60.9219 117

60.9310 76

60.9400 137

60.9491 116

60.9582 164

60.9673 130

60.9764 94

60.9855 73

60.9946 57

61.0036 111

61.0127 138

61.0218 124

61.0309 100

61.0400 128

61.0491 102

61.0582 96

61.0672 133

61.0763 131

61.0854 77

61.0945 209

61.1036 110

61.1127 157

61.1218 40

61.1308 174

61.1399 117

61.1490 140

61.1581 46

61.1672 70

61.1763 98

61.1854 169

61.1944 98

61.2035 81

61.2126 67

61.2217 17

61.2308 85

61.2399 134

61.2489 115

61.2580 67

61.2671 85

61.2762 113

61.2853 142

61.2944 88

61.3035 152

61.3125 114

61.3216 133

61.3307 115

61.3398 146

61.3489 142

61.3580 119

61.3671 103

61.3761 107

61.3852 121

61.3943 60

61.4034 73

61.4125 153

61.4216 133

61.4307 149

61.4397 105

61.4488 117

61.4579 105

61.4670 175

61.4761 131

61.4852 149

61.4943 167

61.5033 128

61.5124 142

61.5215 164

61.5306 157

61.5397 159

61.5488 125

61.5579 167

61.5669 142

61.5760 120

61.5851 197

61.5942 168

61.6033 117

61.6124 144

61.6215 107

61.6305 211

61.6396 167

61.6487 189

61.6578 185

61.6669 149

61.6760 169

61.6851 120

61.6941 189

61.7032 169

61.7123 203

61.7214 192

61.7305 202

61.7396 204

61.7487 199

61.7577 62

61.7668 207

61.7759 208

61.7850 147

61.7941 146

61.8032 195

61.8123 180

61.8213 140

61.8304 146

61.8395 204

61.8486 133

61.8577 215

61.8668 192

61.8759 283

61.8849 172

61.8940 124

61.9031 155

61.9122 197

61.9213 212

61.9304 176

61.9395 130

61.9485 209

61.9576 223

61.9667 133

61.9758 157

61.9849 203

61.9940 71

62.0031 223

62.0121 132

62.0212 142

62.0303 151

62.0394 140

62.0485 160

62.0576 182

62.0667 205

62.0757 155

62.0848 151

62.0939 185

62.1030 173

62.1121 146

62.1212 198

62.1303 181

62.1393 223

62.1484 82

62.1575 227

62.1666 227

62.1757 191

62.1848 146

62.1939 188

62.2029 104

62.2120 183

62.2211 153

62.2302 117

62.2393 199

62.2484 137

62.2575 152

62.2665 138

62.2756 154

62.2847 217

62.2938 158

62.3029 134

62.3120 154

62.3211 87

62.3301 231

62.3392 228

62.3483 204

62.3574 82

62.3665 168

62.3756 186

62.3846 75

62.3937 170

62.4028 179

62.4119 144

62.4210 131

62.4301 114

62.4392 113

62.4482 182

62.4573 185

62.4664 128

62.4755 135

62.4846 157

62.4937 107

62.5028 144

62.5118 81

62.5209 162

62.5300 117

62.5391 126

62.5482 120

62.5573 143

62.5664 134

62.5754 167

62.5845 83

62.5936 142

62.6027 127

62.6118 164

62.6209 112

62.6300 89

62.6390 182

62.6481 150

62.6572 108

62.6663 114

62.6754 125

62.6845 89

62.6936 81

62.7026 115

62.7117 104

62.7208 80

62.7299 74

62.7390 97

62.7481 119

62.7572 102

62.7662 94

62.7753 99

62.7844 92

62.7935 149

62.8026 121

62.8117 144

62.8208 115

62.8298 123

62.8389 134

62.8480 149

62.8571 94

62.8662 114

62.8753 169

62.8844 130

62.8934 135

62.9025 73

62.9116 71

62.9207 83

62.9298 134

62.9389 80

62.9480 79

62.9570 168

62.9661 103

62.9752 43

62.9843 90

62.9934 81

63.0025 146

63.0116 166

63.0206 130

63.0297 113

63.0388 103

63.0479 76

63.0570 73

63.0661 134

63.0752 123

63.0842 92

63.0933 101

63.1024 101

63.1115 125

63.1206 103

63.1297 103

63.1388 68

63.1478 99

63.1569 133

63.1660 122

63.1751 29

63.1842 136

63.1933 127

63.2024 81

63.2114 75

63.2205 88

63.2296 141

63.2387 130

63.2478 94

63.2569 43

63.2660 121

63.2750 82

63.2841 80

63.2932 23

63.3023 64

63.3114 117

63.3205 90

63.3296 106

63.3386 142

63.3477 87

63.3568 -21

63.3659 17

63.3750 57

63.3841 81

63.3932 94

63.4022 81

63.4113 113

63.4204 82

63.4295 91

63.4386 51

63.4477 51

63.4568 32

63.4658 108

63.4749 139

63.4840 90

63.4931 150

63.5022 161

63.5113 70

63.5203 72

63.5294 105

63.5385 99

63.5476 139

63.5567 122

63.5658 115

63.5749 118

63.5839 108

63.5930 97

63.6021 124

63.6112 70

63.6203 101

63.6294 65

63.6385 25

63.6475 142

63.6566 108

63.6657 103

63.6748 101

63.6839 71

63.6930 109

63.7021 90

63.7111 120

63.7202 84

63.7293 17

63.7384 89

63.7475 91

63.7566 90

63.7657 63

63.7747 103

63.7838 101

63.7929 72

63.8020 46

63.8111 85

63.8202 103

63.8293 76

63.8383 80

63.8474 83

63.8565 115

63.8656 132

63.8747 92

63.8838 138

63.8929 152

63.9019 53

63.9110 101

63.9201 91

63.9292 43

63.9383 111

63.9474 148

63.9565 100

63.9655 152

63.9746 119

63.9837 101

63.9928 104

64.0019 76

64.0110 56

64.0201 87

64.0291 96

64.0382 59

64.0473 96

64.0564 58

64.0655 49

64.0746 114

64.0837 93

64.0927 115

64.1018 91

64.1109 148

64.1200 72

64.1291 78

64.1382 80

64.1473 71

64.1563 83

64.1654 55

64.1745 112

64.1836 52

64.1927 38

64.2018 99

64.2109 96

64.2199 45

64.2290 99

64.2381 61

64.2472 18

64.2563 77

64.2654 62

64.2745 97

64.2835 141

64.2926 86

64.3017 100

64.3108 105

64.3199 75

64.3290 83

64.3381 57

64.3471 97

64.3562 93

64.3653 62

64.3744 73

64.3835 79

64.3926 106

64.4017 71

64.4107 86

64.4198 82

64.4289 64

64.4380 48

64.4471 111

64.4562 116

64.4653 71

64.4743 40

64.4834 149

64.4925 110

64.5016 88

64.5107 46

64.5198 64

64.5289 62

64.5379 49

64.5470 32

64.5561 75

64.5652 134

64.5743 135

64.5834 49

64.5925 80

64.6015 66

64.6106 35

64.6197 116

64.6288 86

64.6379 121

64.6470 54

64.6560 120

64.6651 154

64.6742 72

64.6833 108

64.6924 121

64.7015 78

64.7106 112

64.7196 152

64.7287 120

64.7378 149

64.7469 103

64.7560 4

64.7651 98

64.7742 63

64.7832 65

64.7923 22

64.8014 82

64.8105 103

64.8196 127

64.8287 62

64.8378 98

64.8468 96

64.8559 91

64.8650 84

64.8741 80

64.8832 114

64.8923 69

64.9014 12

64.9104 61

64.9195 108

64.9286 21

64.9377 100

64.9468 86

64.9559 79

64.9650 105

64.9740 30

64.9831 103

64.9922 109

65.0013 16

65.0104 85

65.0195 50

65.0286 112

65.0376 95

65.0467 92

65.0558 107

65.0649 93

65.0740 109

65.0831 155

65.0922 86

65.1012 86

65.1103 29

65.1194 100

65.1285 91

65.1376 109

65.1467 122

65.1558 71

65.1648 152

65.1739 145

65.1830 131

65.1921 55

65.2012 116

65.2103 101

65.2194 98

65.2284 136

65.2375 138

65.2466 84

65.2557 141

65.2648 53

65.2739 85

65.2830 95

65.2920 122

65.3011 58

65.3102 64

65.3193 167

65.3284 35

65.3375 12

65.3466 97

65.3556 128

65.3647 23

65.3738 61

65.3829 80

65.3920 91

65.4011 102

65.4102 58

65.4192 77

65.4283 72

65.4374 118

65.4465 122

65.4556 68

65.4647 73

65.4738 128

65.4828 64

65.4919 79

65.5010 129

65.5101 147

65.5192 77

65.5283 100

65.5374 151

65.5464 105

65.5555 58

65.5646 89

65.5737 109

65.5828 106

65.5919 65

65.6010 62

65.6100 76

65.6191 46

65.6282 118

65.6373 97

65.6464 100

65.6555 123

65.6646 62

65.6736 82

65.6827 74

65.6918 87

65.7009 163

65.7100 95

65.7191 46

65.7282 92

65.7372 92

65.7463 81

65.7554 65

65.7645 82

65.7736 43

65.7827 66

65.7917 98

65.8008 31

65.8099 131

65.8190 140

65.8281 38

65.8372 140

65.8463 87

65.8553 73

65.8644 122

65.8735 126

65.8826 88

65.8917 102

65.9008 110

65.9099 36

65.9189 36

65.9280 80

65.9371 92

65.9462 61

65.9553 86

65.9644 115

65.9735 83

65.9825 62

65.9916 65

66.0007 108

66.0098 73

66.0189 82

66.0280 71

66.0371 80

66.0461 71

66.0552 106

66.0643 102

66.0734 97

66.0825 99

66.0916 143

66.1007 36

66.1097 139

66.1188 68

66.1279 114

66.1370 72

66.1461 89

66.1552 111

66.1643 94

66.1733 151

66.1824 95

66.1915 131

66.2006 109

66.2097 60

66.2188 66

66.2279 115

66.2369 29

66.2460 117

66.2551 21

66.2642 56

66.2733 77

66.2824 38

66.2915 63

66.3005 69

66.3096 64

66.3187 100

66.3278 115

66.3369 142

66.3460 102

66.3551 54

66.3641 67

66.3732 75

66.3823 78

66.3914 45

66.4005 26

66.4096 117

66.4187 78

66.4277 103

66.4368 137

66.4459 25

66.4550 52

66.4641 66

66.4732 120

66.4823 121

66.4913 100

66.5004 52

66.5095 68

66.5186 73

66.5277 71

66.5368 65

66.5459 68

66.5549 20

66.5640 80

66.5731 113

66.5822 49

66.5913 87

66.6004 144

66.6095 91

66.6185 103

66.6276 61

66.6367 58

66.6458 103

66.6549 130

66.6640 79

66.6731 65

66.6821 3

66.6912 73

66.7003 67

66.7094 42

66.7185 44

66.7276 149

66.7367 69

66.7457 41

66.7548 133

66.7639 89

66.7730 100

66.7821 140

66.7912 95

66.8003 60

66.8093 95

66.8184 93

66.8275 98

66.8366 118

66.8457 75

66.8548 91

66.8639 102

66.8729 129

66.8820 67

66.8911 110

66.9002 69

66.9093 57

66.9184 132

66.9274 138

66.9365 91

66.9456 120

66.9547 73

66.9638 145

66.9729 118

66.9820 104

66.9910 105

67.0001 43

67.0092 72

67.0183 57

67.0274 128

67.0365 87

67.0456 81

67.0546 156

67.0637 111

67.0728 90

67.0819 86

67.0910 109

67.1001 147

67.1092 80

67.1182 80

67.1273 112

67.1364 23

67.1455 122

67.1546 105

67.1637 109

67.1728 72

67.1818 125

67.1909 40

67.2000 84

67.2091 33

67.2182 100

67.2273 111

67.2364 75

67.2454 122

67.2545 147

67.2636 90

67.2727 67

67.2818 161

67.2909 70

67.3000 90

67.3090 85

67.3181 71

67.3272 89

67.3363 75

67.3454 52

67.3545 46

67.3636 86

67.3726 95

67.3817 143

67.3908 47

67.3999 59

67.4090 32

67.4181 57

67.4272 118

67.4362 132

67.4453 23

67.4544 80

67.4635 108

67.4726 100

67.4817 63

67.4908 18

67.4998 68

67.5089 120

67.5180 82

67.5271 92

67.5362 93

67.5453 103

67.5544 87

67.5634 119

67.5725 81

67.5816 75

67.5907 107

67.5998 56

67.6089 72

67.6180 136

67.6270 86

67.6361 75

67.6452 101

67.6543 50

67.6634 70

67.6725 124

67.6816 112

67.6906 84

67.6997 51

67.7088 128

67.7179 109

67.7270 107

67.7361 34

67.7452 62

67.7542 104

67.7633 71

67.7724 58

67.7815 53

67.7906 53

67.7997 106

67.8088 46

67.8178 67

67.8269 47

67.8360 58

67.8451 71

67.8542 127

67.8633 104

67.8724 109

67.8814 99

67.8905 95

67.8996 79

67.9087 62

67.9178 120

67.9269 93

67.9360 102

67.9450 161

67.9541 76

67.9632 61

67.9723 112

67.9814 53

67.9905 119

67.9996 64

68.0086 87

68.0177 7

68.0268 57

68.0359 66

68.0450 86

68.0541 62

68.0631 108

68.0722 81

68.0813 110

68.0904 99

68.0995 90

68.1086 46

68.1177 68

68.1267 122

68.1358 77

68.1449 65

68.1540 108

68.1631 82

68.1722 76

68.1813 38

68.1903 66

68.1994 97

68.2085 68

68.2176 74

68.2267 48

68.2358 140

68.2449 143

68.2539 74

68.2630 98

68.2721 91

68.2812 101

68.2903 54

68.2994 124

68.3085 132

68.3175 63

68.3266 81

68.3357 110

68.3448 77

68.3539 72

68.3630 79

68.3721 89

68.3811 65

68.3902 113

68.3993 55

68.4084 42

68.4175 99

68.4266 107

68.4357 68

68.4447 50

68.4538 102

68.4629 42

68.4720 31

68.4811 88

68.4902 72

68.4993 17

68.5083 115

68.5174 108

68.5265 91

68.5356 86

68.5447 92

68.5538 151

68.5629 107

68.5719 79

68.5810 117

68.5901 106

68.5992 68

68.6083 46

68.6174 99

68.6265 29

68.6355 60

68.6446 84

68.6537 132

68.6628 105

68.6719 103

68.6810 109

68.6901 65

68.6991 82

68.7082 3

68.7173 113

68.7264 117

68.7355 64

68.7446 88

68.7537 38

68.7627 61

68.7718 108

68.7809 79

68.7900 115

68.7991 68

68.8082 66

68.8173 137

68.8263 119

68.8354 37

68.8445 47

68.8536 129

68.8627 155

68.8718 101

68.8809 82

68.8899 157

68.8990 115

68.9081 73

68.9172 68

68.9263 53

68.9354 98

68.9445 87

68.9535 107

68.9626 89

68.9717 23

68.9808 42

68.9899 110

68.9990 119

69.0081 114

69.0171 108

69.0262 82

69.0353 79

69.0444 55

69.0535 125

69.0626 47

69.0717 83

69.0807 26

69.0898 74

69.0989 133

69.1080 36

69.1171 124

69.1262 137

69.1353 68

69.1443 36

69.1534 80

69.1625 32

69.1716 96

69.1807 47

69.1898 31

69.1988 64

69.2079 91

69.2170 41

69.2261 77

69.2352 64

69.2443 87

69.2534 54

69.2624 85

69.2715 30

69.2806 63

69.2897 93

69.2988 85

69.3079 59

69.3170 86

69.3260 72

69.3351 44

69.3442 80

69.3533 91

69.3624 83

69.3715 67

69.3806 49

69.3896 82

69.3987 123

69.4078 42

69.4169 101

69.4260 70

69.4351 105

69.4442 83

69.4532 52

69.4623 94

69.4714 56

69.4805 44

69.4896 154

69.4987 51

69.5078 71

69.5168 102

69.5259 54

69.5350 84

69.5441 71

69.5532 59

69.5623 87

69.5714 128

69.5804 103

69.5895 126

69.5986 106

69.6077 122

69.6168 51

69.6259 102

69.6350 96

69.6440 22

69.6531 29

69.6622 118

69.6713 110

69.6804 56

69.6895 40

69.6986 35

69.7076 93

69.7167 142

69.7258 111

69.7349 51

69.7440 52

69.7531 47

69.7622 69

69.7712 89

69.7803 32

69.7894 28

69.7985 137

69.8076 84

69.8167 143

69.8258 67

69.8348 109

69.8439 102

69.8530 30

69.8621 126

69.8712 74

69.8803 100

69.8894 99

69.8984 83

69.9075 17

69.9166 50

69.9257 110

69.9348 76

69.9439 104

69.9530 82

69.9620 84

69.9711 80

69.9802 109

69.9893 34

69.9984 22

70.0075 124

70.0166 90

70.0256 102

70.0347 67

70.0438 56

70.0529 128

70.0620 83

70.0711 69

70.0802 101

70.0892 60

70.0983 42

70.1074 119

70.1165 60

70.1256 26

70.1347 115

70.1438 66

70.1528 59

70.1619 144

70.1710 141

70.1801 127

70.1892 68

70.1983 118

70.2074 94

70.2164 53

70.2255 105

70.2346 156

70.2437 83

70.2528 110

70.2619 72

70.2710 143

70.2800 29

70.2891 63

70.2982 104

70.3073 98

70.3164 102

70.3255 47

70.3345 79

70.3436 104

70.3527 84

70.3618 89

70.3709 133

70.3800 107

70.3891 167

70.3981 83

70.4072 119

70.4163 120

70.4254 42

70.4345 30

70.4436 74

70.4527 99

70.4617 102

70.4708 98

70.4799 2

70.4890 57

70.4981 65

70.5072 80

70.5163 104

70.5253 74

70.5344 78

70.5435 96

70.5526 66

70.5617 40

70.5708 90

70.5799 59

70.5889 25

70.5980 109

70.6071 97

70.6162 54

70.6253 76

70.6344 85

70.6435 81

70.6525 109

70.6616 97

70.6707 10

70.6798 116

70.6889 51

70.6980 109

70.7071 125

70.7161 79

70.7252 159

70.7343 161

70.7434 93

70.7525 72

70.7616 79

70.7707 57

70.7797 79

70.7888 44

70.7979 52

70.8070 49

70.8161 102

70.8252 99

70.8343 35

70.8433 63

70.8524 96

70.8615 49

70.8706 95

70.8797 34

70.8888 146

70.8979 102

70.9069 107

70.9160 67

70.9251 65

70.9342 93

70.9433 105

70.9524 9

70.9615 113

70.9705 144

70.9796 113

70.9887 24

70.9978 80

71.0069 35

71.0160 9

71.0251 105

71.0341 64

71.0432 114

71.0523 117

71.0614 74

71.0705 60

71.0796 61

71.0887 137

71.0977 121

71.1068 -5

71.1159 136

71.1250 118

71.1341 97

71.1432 94

71.1523 63

71.1613 47

71.1704 92

71.1795 20

71.1886 126

71.1977 94

71.2068 94

71.2159 101

71.2249 103

71.2340 60

71.2431 93

71.2522 113

71.2613 15

71.2704 103

71.2795 112

71.2885 74

71.2976 116

71.3067 73

71.3158 73

71.3249 72

71.3340 91

71.3431 45

71.3521 91

71.3612 65

71.3703 148

71.3794 161

71.3885 85

71.3976 127

71.4067 102

71.4157 103

71.4248 96

71.4339 26

71.4430 97

71.4521 45

71.4612 65

71.4702 85

71.4793 62

71.4884 87

71.4975 46

71.5066 153

71.5157 77

71.5248 86

71.5338 64

71.5429 32

71.5520 110

71.5611 29

71.5702 115

71.5793 104

71.5884 111

71.5974 55

71.6065 49

71.6156 60

71.6247 131

71.6338 59

71.6429 168

71.6520 53

71.6610 38

71.6701 110

71.6792 50

71.6883 49

71.6974 106

71.7065 117

71.7156 129

71.7246 45

71.7337 90

71.7428 71

71.7519 52

71.7610 109

71.7701 96

71.7792 101

71.7882 151

71.7973 97

71.8064 77

71.8155 117

71.8246 79

71.8337 74

71.8428 143

71.8518 41

71.8609 92

71.8700 52

71.8791 52

71.8882 -1

71.8973 89

71.9064 94

71.9154 125

71.9245 53

71.9336 142

71.9427 48

71.9518 151

71.9609 62

71.9700 135

71.9790 89

71.9881 123

71.9972 111

72.0063 96

72.0154 97

72.0245 88

72.0336 101

72.0426 58

72.0517 103

72.0608 131

72.0699 112

72.0790 57

72.0881 56

72.0972 39

72.1062 119

72.1153 115

72.1244 46

72.1335 104

72.1426 106

72.1517 89

72.1608 89

72.1698 74

72.1789 111

72.1880 40

72.1971 58

72.2062 153

72.2153 48

72.2244 126

72.2334 57

72.2425 83

72.2516 63

72.2607 40

72.2698 136

72.2789 23

72.2880 68

72.2970 94

72.3061 47

72.3152 41

72.3243 83

72.3334 104

72.3425 73

72.3516 100

72.3606 68

72.3697 76

72.3788 8

72.3879 163

72.3970 76

72.4061 27

72.4152 78

72.4242 49

72.4333 126

72.4424 72

72.4515 107

72.4606 51

72.4697 114

72.4788 111

72.4878 96

72.4969 54

72.5060 115

72.5151 47

72.5242 44

72.5333 37

72.5424 73

72.5514 64

72.5605 113

72.5696 70

72.5787 77

72.5878 107

72.5969 89

72.6059 84

72.6150 125

72.6241 104

72.6332 75

72.6423 81

72.6514 87

72.6605 78

72.6695 130

72.6786 64

72.6877 122

72.6968 42

72.7059 49

72.7150 143

72.7241 92

72.7331 55

72.7422 103

72.7513 91

72.7604 107

72.7695 67

72.7786 68

72.7877 46

72.7967 61

72.8058 107

72.8149 68

72.8240 62

72.8331 53

72.8422 49

72.8513 83

72.8603 102

72.8694 118

72.8785 72

72.8876 35

72.8967 105

72.9058 40

72.9149 40

72.9239 75

72.9330 126

72.9421 107

72.9512 72

72.9603 89

72.9694 81

72.9785 42

72.9875 82

72.9966 122

73.0057 107

73.0148 34

73.0239 81

73.0330 50

73.0421 37

73.0511 113

73.0602 68

73.0693 160

73.0784 98

73.0875 100

73.0966 90

73.1057 82

73.1147 92

73.1238 99

73.1329 131

73.1420 53

73.1511 105

73.1602 92

73.1693 88

73.1783 75

73.1874 95

73.1965 119

73.2056 108

73.2147 100

73.2238 93

73.2329 102

73.2419 85

73.2510 119

73.2601 75

73.2692 160

73.2783 69

73.2874 151

73.2965 97

73.3055 57

73.3146 49

73.3237 60

73.3328 86

73.3419 137

73.3510 64

73.3601 119

73.3691 63

73.3782 16

73.3873 26

73.3964 74

73.4055 62

73.4146 66

73.4237 103

73.4327 121

73.4418 111

73.4509 117

73.4600 75

73.4691 120

73.4782 109

73.4873 51

73.4963 89

73.5054 163

73.5145 114

73.5236 88

73.5327 124

73.5418 86

73.5509 116

73.5599 103

73.5690 112

73.5781 66

73.5872 79

73.5963 67

73.6054 83

73.6145 71

73.6235 54

73.6326 85

73.6417 110

73.6508 98

73.6599 103

73.6690 158

73.6781 107

73.6871 86

73.6962 76

73.7053 97

73.7144 90

73.7235 95

73.7326 161

73.7416 66

73.7507 118

73.7598 103

73.7689 123

73.7780 103

73.7871 88

73.7962 142

73.8052 123

73.8143 129

73.8234 147

73.8325 53

73.8416 125

73.8507 127

73.8598 121

73.8688 117

73.8779 101

73.8870 56

73.8961 123

73.9052 80

73.9143 63

73.9234 78

73.9324 108

73.9415 147

73.9506 85

73.9597 114

73.9688 76

73.9779 101

73.9870 137

73.9960 135

74.0051 66

74.0142 73

74.0233 135

74.0324 131

74.0415 59

74.0506 119

74.0596 146

74.0687 87

74.0778 73

74.0869 73

74.0960 160

74.1051 86

74.1142 141

74.1232 115

74.1323 124

74.1414 79

74.1505 131

74.1596 122

74.1687 104

74.1778 104

74.1868 163

74.1959 164

74.2050 122

74.2141 121

74.2232 95

74.2323 98

74.2414 195

74.2504 97

74.2595 141

74.2686 176

74.2777 127

74.2868 188

74.2959 146

74.3050 169

74.3140 88

74.3231 131

74.3322 48

74.3413 109

74.3504 119

74.3595 102

74.3686 160

74.3776 117

74.3867 141

74.3958 76

74.4049 68

74.4140 106

74.4231 80

74.4322 133

74.4412 100

74.4503 177

74.4594 96

74.4685 149

74.4776 112

74.4867 163

74.4958 134

74.5048 43

74.5139 33

74.5230 114

74.5321 91

74.5412 104

74.5503 85

74.5594 166

74.5684 137

74.5775 175

74.5866 105

74.5957 140

74.6048 170

74.6139 112

74.6230 53

74.6320 123

74.6411 125

74.6502 118

74.6593 129

74.6684 69

74.6775 80

74.6866 123

74.6956 114

74.7047 122

74.7138 116

74.7229 160

74.7320 86

74.7411 120

74.7502 78

74.7592 48

74.7683 105

74.7774 115

74.7865 100

74.7956 111

74.8047 95

74.8138 156

74.8228 84

74.8319 126

74.8410 98

74.8501 114

74.8592 128

74.8683 97

74.8773 64

74.8864 94

74.8955 68

74.9046 116

74.9137 105

74.9228 99

74.9319 114

74.9409 127

74.9500 146

74.9591 88

74.9682 112

74.9773 146

74.9864 131

74.9955 81

75.0045 123

75.0136 98

75.0227 51

75.0318 100

75.0409 126

75.0500 108

75.0591 89

75.0681 105

75.0772 133

75.0863 137

75.0954 109

75.1045 104

75.1136 100

75.1227 80

75.1317 133

75.1408 74

75.1499 156

75.1590 82

75.1681 146

75.1772 142

75.1863 77

75.1953 109

75.2044 113

75.2135 92

75.2226 117

75.2317 101

75.2408 108

75.2499 96

75.2589 94

75.2680 59

75.2771 135

75.2862 156

75.2953 81

75.3044 102

75.3135 66

75.3225 97

75.3316 60

75.3407 81

75.3498 124

75.3589 34

75.3680 69

75.3771 103

75.3861 47

75.3952 101

75.4043 54

75.4134 130

75.4225 67

75.4316 113

75.4407 27

75.4497 108

75.4588 95

75.4679 74

75.4770 116

75.4861 56

75.4952 126

75.5043 13

75.5133 119

75.5224 106

75.5315 52

75.5406 102

75.5497 83

75.5588 105

75.5679 85

75.5769 70

75.5860 51

75.5951 113

75.6042 86

75.6133 85

75.6224 117

75.6315 62

75.6405 96

75.6496 81

75.6587 130

75.6678 149

75.6769 51

75.6860 107

75.6951 117

75.7041 138

75.7132 99

75.7223 140

75.7314 121

75.7405 150

75.7496 122

75.7587 97

75.7677 49

75.7768 107

75.7859 22

75.7950 18

75.8041 154

75.8132 100

75.8223 131

75.8313 34

75.8404 143

75.8495 82

75.8586 106

75.8677 104

75.8768 127

75.8859 116

75.8949 74

75.9040 21

75.9131 94

75.9222 108

75.9313 97

75.9404 71

75.9495 75

75.9585 136

75.9676 184

75.9767 144

75.9858 70

75.9949 96

76.0040 94

76.0130 130

76.0221 48

76.0312 114

76.0403 63

76.0494 73

76.0585 86

76.0676 75

76.0766 63

76.0857 134

76.0948 96

76.1039 55

76.1130 145

76.1221 91

76.1312 126

76.1402 65

76.1493 102

76.1584 83

76.1675 83

76.1766 49

76.1857 112

76.1948 52

76.2038 51

76.2129 94

76.2220 85

76.2311 72

76.2402 94

76.2493 108

76.2584 28

76.2674 98

76.2765 115

76.2856 17

76.2947 65

76.3038 147

76.3129 78

76.3220 64

76.3310 131

76.3401 100

76.3492 85

76.3583 60

76.3674 107

76.3765 76

76.3856 45

76.3946 112

76.4037 34

76.4128 117

76.4219 107

76.4310 63

76.4401 104

76.4492 92

76.4582 100

76.4673 78

76.4764 56

76.4855 62

76.4946 32

76.5037 58

76.5128 101

76.5218 83

76.5309 85

76.5400 111

76.5491 68

76.5582 57

76.5673 47

76.5764 65

76.5854 77

76.5945 90

76.6036 36

76.6127 55

76.6218 68

76.6309 97

76.6400 114

76.6490 102

76.6581 108

76.6672 139

76.6763 92

76.6854 18

76.6945 69

76.7036 56

76.7126 116

76.7217 35

76.7308 109

76.7399 71

76.7490 47

76.7581 67

76.7672 62

76.7762 47

76.7853 98

76.7944 54

76.8035 55

76.8126 76

76.8217 -11

76.8308 72

76.8398 55

76.8489 94

76.8580 119

76.8671 123

76.8762 85

76.8853 62

76.8944 106

76.9034 80

76.9125 60

76.9216 119

76.9307 67

76.9398 65

76.9489 53

76.9580 109

76.9670 66

76.9761 68

76.9852 113

76.9943 98

77.0034 73

77.0125 84

77.0216 67

77.0306 -2

77.0397 162

77.0488 84

77.0579 48

77.0670 103

77.0761 125

77.0852 106

77.0942 76

77.1033 118

77.1124 60

77.1215 119

77.1306 80

77.1397 149

77.1487 109

77.1578 83

77.1669 128

77.1760 89

77.1851 110

77.1942 124

77.2033 107

77.2123 84

77.2214 77

77.2305 72

77.2396 84

77.2487 45

77.2578 117

77.2669 111

77.2759 132

77.2850 51

77.2941 69

77.3032 104

77.3123 83

77.3214 108

77.3305 130

77.3395 82

77.3486 104

77.3577 130

77.3668 125

77.3759 82

77.3850 59

77.3941 103

77.4031 105

77.4122 37

77.4213 97

77.4304 110

77.4395 57

77.4486 23

77.4577 108

77.4667 70

77.4758 14

77.4849 79

77.4940 73

77.5031 110

77.5122 66

77.5213 73

77.5303 60

77.5394 80

77.5485 72

77.5576 87

77.5667 72

77.5758 39

77.5849 97

77.5939 98

77.6030 35

77.6121 82

77.6212 55

77.6303 62

77.6394 98

77.6485 101

77.6575 93

77.6666 50

77.6757 112

77.6848 31

77.6939 68

77.7030 96

77.7121 96

77.7211 106

77.7302 83

77.7393 131

77.7484 126

77.7575 141

77.7666 105

77.7757 70

77.7847 75

77.7938 48

77.8029 140

77.8120 126

77.8211 70

77.8302 83

77.8393 57

77.8483 51

77.8574 104

77.8665 171

77.8756 88

77.8847 103

77.8938 76

77.9029 88

77.9119 103

77.9210 69

77.9301 72

77.9392 75

77.9483 100

77.9574 95

77.9665 78

77.9755 106

77.9846 86

77.9937 134

78.0028 73

78.0119 165

78.0210 167

78.0301 157

78.0391 123

78.0482 115

78.0573 77

78.0664 56

78.0755 80

78.0846 84

78.0937 67

78.1027 96

78.1118 129

78.1209 110

78.1300 110

78.1391 88

78.1482 114

78.1573 91

78.1663 102

78.1754 37

78.1845 136

78.1936 93

78.2027 96

78.2118 32

78.2209 179

78.2299 54

78.2390 57

78.2481 45

78.2572 123

78.2663 99

78.2754 105

78.2844 56

78.2935 157

78.3026 178

78.3117 86

78.3208 124

78.3299 72

78.3390 70

78.3480 134

78.3571 40

78.3662 85

78.3753 116

78.3844 129

78.3935 81

78.4026 107

78.4116 40

78.4207 92

78.4298 99

78.4389 125

78.4480 56

78.4571 122

78.4662 106

78.4752 141

78.4843 117

78.4934 40

78.5025 140

78.5116 97

78.5207 80

78.5298 81

78.5388 135

78.5479 50

78.5570 133

78.5661 67

78.5752 104

78.5843 43

78.5934 81

78.6024 75

78.6115 116

78.6206 117

78.6297 114

78.6388 88

78.6479 28

78.6570 159

78.6660 130

78.6751 24

78.6842 166

78.6933 88

78.7024 32

78.7115 136

78.7206 19

78.7296 57

78.7387 80

78.7478 109

78.7569 72

78.7660 97

78.7751 102

78.7842 74

78.7932 84

78.8023 114

78.8114 162

78.8205 83

78.8296 84

78.8387 174

78.8478 71

78.8568 20

78.8659 40

78.8750 75

78.8841 102

78.8932 83

78.9023 123

78.9114 141

78.9204 137

78.9295 66

78.9386 105

78.9477 130

78.9568 110

78.9659 74

78.9750 74

78.9840 87

78.9931 131

79.0022 110

79.0113 61

79.0204 58

79.0295 71

79.0386 102

79.0476 46

79.0567 49

79.0658 51

79.0749 128

79.0840 124

79.0931 66

79.1022 75

79.1112 82

79.1203 88

79.1294 152

79.1385 118

79.1476 115

79.1567 148

79.1658 35

79.1748 93

79.1839 67

79.1930 72

79.2021 87

79.2112 57

79.2203 110

79.2294 72

79.2384 131

79.2475 73

79.2566 80

79.2657 50

79.2748 105

79.2839 109

79.2930 72

79.3020 58

79.3111 35

79.3202 86

79.3293 133

79.3384 88

79.3475 95

79.3566 55

79.3656 83

79.3747 81

79.3838 29

79.3929 101

79.4020 11

79.4111 3

79.4201 41

79.4292 132

79.4383 106

79.4474 64

79.4565 113

79.4656 92

79.4747 45

79.4837 104

79.4928 88

79.5019 67

79.5110 30

79.5201 67

79.5292 29

79.5383 99

79.5473 78

79.5564 33

79.5655 118

79.5746 117

79.5837 79

79.5928 89

79.6019 27

79.6109 111

79.6200 65

79.6291 106

79.6382 32

79.6473 67

79.6564 122

79.6655 110

79.6745 67

79.6836 59

79.6927 25

79.7018 76

79.7109 86

79.7200 62

79.7291 103

79.7381 35

79.7472 161

79.7563 25

79.7654 91

79.7745 105

79.7836 46

79.7927 81

79.8017 90

79.8108 91

79.8199 94

79.8290 -15

79.8381 92

79.8472 94

79.8563 137

79.8653 51

79.8744 40

79.8835 51

79.8926 117

79.9017 92

79.9108 73

79.9199 131

79.9289 80

79.9380 32

79.9471 82

79.9562 25

79.9653 89

79.9744 106

79.9835 47

79.9925 81

80.0016 21

80.0107 89

80.0198 97

80.0289 68

80.0380 97

80.0471 56

80.0561 37

80.0652 137

80.0743 74

80.0834 62

80.0925 75

80.1016 146

80.1107 88

80.1197 54

80.1288 70

80.1379 46

80.1470 127

80.1561 70

80.1652 18

80.1743 64

80.1833 22

80.1924 104

80.2015 81

80.2106 57

80.2197 80

80.2288 65

80.2379 76

80.2469 91

80.2560 118

80.2651 130

80.2742 56

80.2833 70

80.2924 132

80.3015 113

80.3105 69

80.3196 94

80.3287 54

80.3378 124

80.3469 119

80.3560 73

80.3651 85

80.3741 112

80.3832 72

80.3923 59

80.4014 77

80.4105 82

80.4196 124

80.4287 73

80.4377 47

80.4468 47

80.4559 82

80.4650 59

80.4741 91

80.4832 33

80.4923 112

80.5013 64

80.5104 84

80.5195 64

80.5286 81

80.5377 98

80.5468 67

80.5558 64

80.5649 78

80.5740 115

80.5831 77

80.5922 8

80.6013 32

80.6104 53

80.6194 23

80.6285 48

80.6376 40

80.6467 24

80.6558 126

80.6649 17

80.6740 138

80.6830 65

80.6921 84

80.7012 69

80.7103 75

80.7194 73

80.7285 69

80.7376 101

80.7466 124

80.7557 69

80.7648 68

80.7739 137

80.7830 108

80.7921 49

80.8012 83

80.8102 112

80.8193 75

80.8284 111

80.8375 100

80.8466 116

80.8557 55

80.8648 112

80.8738 44

80.8829 66

80.8920 73

80.9011 111

80.9102 50

80.9193 64

80.9284 47

80.9374 31

80.9465 81

80.9556 91

80.9647 93

80.9738 124

80.9829 98

80.9920 46

81.0010 82

81.0101 84

81.0192 105

81.0283 1

81.0374 55

81.0465 54

81.0556 123

81.0646 71

81.0737 46

81.0828 109

81.0919 35

81.1010 63

81.1101 109

81.1192 58

81.1282 91

81.1373 79

81.1464 55

81.1555 88

81.1646 96

81.1737 18

81.1828 61

81.1918 92

81.2009 144

81.2100 76

81.2191 20

81.2282 61

81.2373 88

81.2464 40

81.2554 94

81.2645 53

81.2736 49

81.2827 99

81.2918 67

81.3009 91

81.3100 77

81.3190 36

81.3281 74

81.3372 63

81.3463 69

81.3554 95

81.3645 121

81.3736 36

81.3826 96

81.3917 88

81.4008 73

81.4099 117

81.4190 43

81.4281 98

81.4372 93

81.4462 61

81.4553 86

81.4644 81

81.4735 19

81.4826 66

81.4917 111

81.5008 80

81.5098 121

81.5189 140

81.5280 143

81.5371 64

81.5462 79

81.5553 41

81.5644 51

81.5734 110

81.5825 60

81.5916 58

81.6007 135

81.6098 85

81.6189 78

81.6280 82

81.6370 91

81.6461 65

81.6552 20

81.6643 100

81.6734 66

81.6825 94

81.6915 40

81.7006 69

81.7097 52

81.7188 80

81.7279 35

81.7370 86

81.7461 47

81.7551 89

81.7642 107

81.7733 95

81.7824 68

81.7915 127

81.8006 88

81.8097 76

81.8187 86

81.8278 137

81.8369 49

81.8460 82

81.8551 134

81.8642 105

81.8733 81

81.8823 27

81.8914 40

81.9005 103

81.9096 99

81.9187 99

81.9278 122

81.9369 48

81.9459 70

81.9550 69

81.9641 97

81.9732 94

81.9823 94

81.9914 58

82.0005 -42

82.0095 50

82.0186 37

82.0277 74

82.0368 21

82.0459 46

82.0550 90

82.0641 133

82.0731 59

82.0822 57

82.0913 10

82.1004 0

82.1095 117

82.1186 94

82.1277 40

82.1367 29

82.1458 96

82.1549 42

82.1640 65

82.1731 39

82.1822 1

82.1913 85

82.2003 81

82.2094 59

82.2185 55

82.2276 138

82.2367 77

82.2458 103

82.2549 112

82.2639 95

82.2730 94

82.2821 87

82.2912 22

82.3003 89

82.3094 59

82.3185 77

82.3275 131

82.3366 63

82.3457 55

82.3548 13

82.3639 64

82.3730 51

82.3821 71

82.3911 33

82.4002 40

82.4093 100

82.4184 91

82.4275 77

82.4366 50

82.4457 62

82.4547 25

82.4638 101

82.4729 116

82.4820 59

82.4911 83

82.5002 103

82.5093 68

82.5183 74

82.5274 90

82.5365 95

82.5456 135

82.5547 45

82.5638 141

82.5729 112

82.5819 105

82.5910 102

82.6001 75

82.6092 50

82.6183 30

82.6274 56

82.6365 125

82.6455 73

82.6546 54

82.6637 117

82.6728 17

82.6819 128

82.6910 72

82.7001 46

82.7091 89

82.7182 73

82.7273 109

82.7364 64

82.7455 62

82.7546 56

82.7637 50

82.7727 68

82.7818 58

82.7909 38

82.8000 50

82.8091 69

82.8182 45

82.8272 100

82.8363 77

82.8454 52

82.8545 109

82.8636 78

82.8727 59

82.8818 137

82.8908 78

82.8999 26

82.9090 24

82.9181 79

82.9272 25

82.9363 42

82.9454 26

82.9544 38

82.9635 68

82.9726 21

82.9817 148

82.9908 84

82.9999 144

83.0090 82

83.0180 107

83.0271 49

83.0362 104

83.0453 37

83.0544 69

83.0635 96

83.0726 132

83.0816 76

83.0907 84

83.0998 63

83.1089 75

83.1180 109

83.1271 92

83.1362 92

83.1452 53

83.1543 47

83.1634 82

83.1725 53

83.1816 15

83.1907 68

83.1998 123

83.2088 89

83.2179 -25

83.2270 68

83.2361 66

83.2452 78

83.2543 108

83.2634 102

83.2724 109

83.2815 55

83.2906 35

83.2997 29

83.3088 26

83.3179 77

83.3270 93

83.3360 89

83.3451 57

83.3542 45

83.3633 33

83.3724 57

83.3815 116

83.3906 68

83.3996 53

83.4087 114

83.4178 17

83.4269 9

83.4360 67

83.4451 54

83.4542 47

83.4632 87

83.4723 51

83.4814 107

83.4905 88

83.4996 88

83.5087 148

83.5178 77

83.5268 58

83.5359 83

83.5450 76

83.5541 109

83.5632 82

83.5723 87

83.5814 40

83.5904 87

83.5995 94

83.6086 10

83.6177 135

83.6268 140

83.6359 141

83.6450 53

83.6540 31

83.6631 18

83.6722 55

83.6813 107

83.6904 96

83.6995 123

83.7086 28

83.7176 133

83.7267 53

83.7358 112

83.7449 81

83.7540 91

83.7631 102

83.7722 115

83.7812 71

83.7903 91

83.7994 36

83.8085 47

83.8176 56

83.8267 77

83.8358 47

83.8448 43

83.8539 107

83.8630 23

83.8721 54

83.8812 75

83.8903 25

83.8994 101

83.9084 22

83.9175 48

83.9266 39

83.9357 84

83.9448 45

83.9539 67

83.9629 80

83.9720 59

83.9811 37

83.9902 93

83.9993 33

84.0084 66

84.0175 61

84.0265 56

84.0356 51

84.0447 39

84.0538 63

84.0629 63

84.0720 43

84.0811 80

84.0901 142

84.0992 58

84.1083 91

84.1174 94

84.1265 67

84.1356 64

84.1447 53

84.1537 76

84.1628 59

84.1719 53

84.1810 64

84.1901 73

84.1992 102

84.2083 77

84.2173 90

84.2264 118

84.2355 81

84.2446 104

84.2537 146

84.2628 75

84.2719 59

84.2809 86

84.2900 64

84.2991 72

84.3082 100

84.3173 130

84.3264 50

84.3355 29

84.3445 114

84.3536 25

84.3627 82

84.3718 41

84.3809 104

84.3900 84

84.3991 72

84.4081 138

84.4172 65

84.4263 128

84.4354 17

84.4445 52

84.4536 62

84.4627 129

84.4717 88

84.4808 146

84.4899 63

84.4990 21

84.5081 103

84.5172 160

84.5263 99

84.5353 34

84.5444 67

84.5535 69

84.5626 33

84.5717 67

84.5808 104

84.5899 77

84.5989 12

84.6080 59

84.6171 37

84.6262 116

84.6353 51

84.6444 68

84.6535 69

84.6625 28

84.6716 66

84.6807 65

84.6898 143

84.6989 30

84.7080 93

84.7171 75

84.7261 36

84.7352 36

84.7443 32

84.7534 123

84.7625 76

84.7716 73

84.7807 62

84.7897 79

84.7988 94

84.8079 103

84.8170 53

84.8261 116

84.8352 60

84.8443 88

84.8533 72

84.8624 40

84.8715 64

84.8806 107

84.8897 69

84.8988 101

84.9079 90

84.9169 14

84.9260 84

84.9351 74

84.9442 9

84.9533 92

84.9624 46

84.9715 70

84.9805 113

84.9896 35

84.9987 76

85.0078 50

85.0169 18

85.0260 33

85.0351 57

85.0441 35

85.0532 43

85.0623 8

85.0714 119

85.0805 117

85.0896 42

85.0986 -8

85.1077 27

85.1168 61

85.1259 75

85.1350 64

85.1441 98

85.1532 135

85.1622 117

85.1713 87

85.1804 53

85.1895 80

85.1986 15

85.2077 54

85.2168 98

85.2258 77

85.2349 40

85.2440 82

85.2531 35

85.2622 47

85.2713 71

85.2804 127

85.2894 108

85.2985 30

85.3076 65

85.3167 57

85.3258 38

85.3349 36

85.3440 84

85.3530 92

85.3621 41

85.3712 110

85.3803 145

85.3894 101

85.3985 142

85.4076 64

85.4166 51

85.4257 49

85.4348 32

85.4439 37

85.4530 16

85.4621 84

85.4712 32

85.4802 68

85.4893 12

85.4984 54

85.5075 38

85.5166 109

85.5257 43

85.5348 67

85.5438 45

85.5529 79

85.5620 26

85.5711 -19

85.5802 60

85.5893 79

85.5984 105

85.6074 100

85.6165 92

85.6256 46

85.6347 -9

85.6438 42

85.6529 62

85.6620 122

85.6710 11

85.6801 101

85.6892 51

85.6983 -6

85.7074 65

85.7165 47

85.7256 77

85.7346 94

85.7437 122

85.7528 124

85.7619 44

85.7710 59

85.7801 94

85.7892 49

85.7982 93

85.8073 40

85.8164 53

85.8255 102

85.8346 97

85.8437 92

85.8528 63

85.8618 113

85.8709 90

85.8800 9

85.8891 8

85.8982 47

85.9073 109

85.9164 61

85.9254 27

85.9345 132

85.9436 95

85.9527 60

85.9618 69

85.9709 22

85.9800 96

85.9890 28

85.9981 52

86.0072 101

86.0163 63

86.0254 85

86.0345 85

86.0436 71

86.0526 111

86.0617 19

86.0708 96

86.0799 67

86.0890 104

86.0981 146

86.1072 43

86.1162 73

86.1253 6

86.1344 85

86.1435 43

86.1526 98

86.1617 78

86.1708 82

86.1798 94

86.1889 65

86.1980 65

86.2071 89

86.2162 69

86.2253 143

86.2343 80

86.2434 32

86.2525 76

86.2616 99

86.2707 88

86.2798 86

86.2889 10

86.2979 51

86.3070 93

86.3161 93

86.3252 92

86.3343 46

86.3434 65

86.3525 66

86.3615 100

86.3706 84

86.3797 76

86.3888 60

86.3979 71

86.4070 46

86.4161 51

86.4251 57

86.4342 101

86.4433 66

86.4524 24

86.4615 26

86.4706 78

86.4797 43

86.4887 87

86.4978 90

86.5069 91

86.5160 59

86.5251 68

86.5342 40

86.5433 66

86.5523 93

86.5614 85

86.5705 113

86.5796 58

86.5887 27

86.5978 37

86.6069 91

86.6159 100

86.6250 98

86.6341 91

86.6432 69

86.6523 0

86.6614 88

86.6705 -18

86.6795 40

86.6886 51

86.6977 95

86.7068 35

86.7159 82

86.7250 70

86.7341 16

86.7431 133

86.7522 32

86.7613 93

86.7704 36

86.7795 76

86.7886 38

86.7977 119

86.8067 149

86.8158 138

86.8249 87

86.8340 95

86.8431 102

86.8522 110

86.8613 85

86.8703 72

86.8794 80

86.8885 114

86.8976 55

86.9067 65

86.9158 48

86.9249 52

86.9339 36

86.9430 37

86.9521 79

86.9612 105

86.9703 123

86.9794 97

86.9885 106

86.9975 104

87.0066 86

87.0157 80

87.0248 112

87.0339 56

87.0430 34

87.0521 38

87.0611 18

87.0702 103

87.0793 65

87.0884 61

87.0975 101

87.1066 112

87.1157 98

87.1247 7

87.1338 87

87.1429 73

87.1520 34

87.1611 48

87.1702 59

87.1793 34

87.1883 109

87.1974 87

87.2065 38

87.2156 40

87.2247 63

87.2338 100

87.2429 84

87.2519 58

87.2610 126

87.2701 40

87.2792 57

87.2883 43

87.2974 93

87.3065 67

87.3155 100

87.3246 53

87.3337 88

87.3428 67

87.3519 67

87.3610 127

87.3700 61

87.3791 10

87.3882 46

87.3973 54

87.4064 67

87.4155 86

87.4246 30

87.4336 89

87.4427 50

87.4518 87

87.4609 64

87.4700 89

87.4791 98

87.4882 83

87.4972 68

87.5063 30

87.5154 55

87.5245 56

87.5336 94

87.5427 70

87.5518 82

87.5608 64

87.5699 74

87.5790 65

87.5881 81

87.5972 93

87.6063 117

87.6154 101

87.6244 38

87.6335 89

87.6426 73

87.6517 56

87.6608 49

87.6699 46

87.6790 110

87.6880 99

87.6971 87

87.7062 134

87.7153 79

87.7244 38

87.7335 77

87.7426 132

87.7516 52

87.7607 64

87.7698 41

87.7789 62

87.7880 141

87.7971 74

87.8062 61

87.8152 29

87.8243 39

87.8334 44

87.8425 66

87.8516 48

87.8607 96

87.8698 31

87.8788 85

87.8879 42

87.8970 40

87.9061 18

87.9152 88

87.9243 15

87.9334 16

87.9424 45

87.9515 19

87.9606 61

87.9697 79

87.9788 81

87.9879 78

87.9970 75

88.0060 62

88.0151 104

88.0242 61

88.0333 58

88.0424 73

88.0515 65

88.0606 28

88.0696 34

88.0787 54

88.0878 84

88.0969 101

88.1060 64

88.1151 46

88.1242 61

88.1332 60

88.1423 43

88.1514 47

88.1605 43

88.1696 37

88.1787 69

88.1878 61

88.1968 111

88.2059 55

88.2150 120

88.2241 104

88.2332 50

88.2423 91

88.2514 80

88.2604 42

88.2695 96

88.2786 74

88.2877 111

88.2968 38

88.3059 102

88.3150 84

88.3240 134

88.3331 75

88.3422 57

88.3513 76

88.3604 95

88.3695 30

88.3786 57

88.3876 77

88.3967 92

88.4058 115

88.4149 6

88.4240 70

88.4331 79

88.4422 46

88.4512 70

88.4603 53

88.4694 63

88.4785 89

88.4876 28

88.4967 107

88.5057 50

88.5148 15

88.5239 32

88.5330 54

88.5421 80

88.5512 118

88.5603 20

88.5693 46

88.5784 25

88.5875 112

88.5966 11

88.6057 54

88.6148 87

88.6239 55

88.6329 79

88.6420 111

88.6511 93

88.6602 12

88.6693 74

88.6784 115

88.6875 28

88.6965 76

88.7056 77

88.7147 101

88.7238 5

88.7329 45

88.7420 64

88.7511 98

88.7601 80

88.7692 96

88.7783 62

88.7874 65

88.7965 10

88.8056 93

88.8147 11

88.8237 60

88.8328 37

88.8419 95

88.8510 87

88.8601 60

88.8692 75

88.8783 93

88.8873 41

88.8964 38

88.9055 91

88.9146 58

88.9237 14

88.9328 106

88.9419 20

88.9509 81

88.9600 -10

88.9691 42

88.9782 22

88.9873 86

88.9964 110

89.0055 73

89.0145 63

89.0236 94

89.0327 84

89.0418 98

89.0509 48

89.0600 110

89.0691 80

89.0781 52

89.0872 69

89.0963 105

89.1054 79

89.1145 5

89.1236 50

89.1327 55

89.1417 52

89.1508 68

89.1599 104

89.1690 118

89.1781 110

89.1872 83

89.1963 102

89.2053 66

89.2144 139

89.2235 38

89.2326 71

89.2417 47

89.2508 40

89.2599 98

89.2689 58

89.2780 67

89.2871 92

89.2962 68

89.3053 138

89.3144 52

89.3235 32

89.3325 66

89.3416 76

89.3507 43

89.3598 79

89.3689 87

89.3780 62

89.3871 88

89.3961 48

89.4052 27

89.4143 76

89.4234 51

89.4325 72

89.4416 78

89.4507 58

89.4597 46

89.4688 77

89.4779 49

89.4870 61

89.4961 87

89.5052 100

89.5143 32

89.5233 50

89.5324 16

89.5415 13

89.5506 88

89.5597 4

89.5688 84

89.5779 97

89.5869 95

89.5960 52

89.6051 79

89.6142 44

89.6233 36

89.6324 98

89.6414 54

89.6505 28

89.6596 127

89.6687 72

89.6778 108

89.6869 48

89.6960 24

89.7050 44

89.7141 62

89.7232 137

89.7323 118

89.7414 14

89.7505 45

89.7596 43

89.7686 31

89.7777 125

89.7868 68

89.7959 68

89.8050 34

89.8141 110

89.8232 104

89.8322 12

89.8413 97

89.8504 105

89.8595 62

89.8686 63

89.8777 35

89.8868 94

89.8958 123

89.9049 57

89.9140 40

89.9231 66

89.9322 33

89.9413 37

89.9504 53

89.9594 7

89.9685 0

89.9776 16

89.9867 55

89.9958 35

90.0049 50

90.0140 72

90.0230 44

90.0321 76

90.0412 56

90.0503 0

**Raw data 4**. XRD raw data of microsphere obtained spray pyrolysis at 850 ℃.

; (content of file C:\DATA\CBNU_IN\ENG-Chemical\JoJungSang\Oh Sehwan\190104\Ni-Co_850C.raw)

_FILEVERSION = 2

_SAMPLE =

_+SAMPLE =

_SITE = Korea

_USER = "Chungbuk UNI."

_GONIOMETER_CODE = 21

; Goniometer : D8 theta/theta, stage : Unknown

_SAMPLE_CHANGER_CODE = 0

_ATTACHMENTS_CODE = 0

_GONIOMETER_RADIUS = 250

_FIXED_DIVSLIT = 0.6

_FIXED_SAMPLESLIT = 0

_FIXED_DETSLIT = 12.21

_MONOCHROMATOR = 0

; Incident beam monochromator : None

_THIN_FILM = N

_BETA_FILTER = N

_FIXED_ANTISLIT = 8.46

_ANALYZER_CODE = 4

; Received beam analyzer : Gobel mirror

_DATEMEASURED = "04-Jan-2019 15:37:33"

_WL_UNIT = A

_WL1 = 1.5406

_WL2 = 1.54439

_WL3 = 1.39222

_WLRATIO = 0.5

_ANODE = Cu

; Data for range 1

_DRIVE = COUPLED

_STEPTIME = 35.8

_STEPSIZE = 0.00908485

_STEPMODE = C

_START = 10

_THETA = 5

_2THETA = 10

_KHI = 0

_PHI = 0

_X = 0

_Y = 0

_Z = 0

_DETECTOR = 5

; Detector type : Unknown

_DETECTORSLIT = out

_AUX1 = 0

_AUX2 = 0

_AUX3 = 0

_TIMESTARTED = 12

_TEMP_RATE = -1

_TEMP_DELAY = -1

_KV = 40

_MA = 40

_RANGE_WL = 1.5406

_3DPLANE = 0

_V4_COUNTERS_MASK = 4096

_V4_DRIVES_MASK = 0

_V4_ENCODERS_MASK = 0

_2THETACOUNTS = 1

; 2THETA PSD

10.0000 871

10.0091 899

10.0182 855

10.0273 839

10.0363 841

10.0454 833

10.0545 804

10.0636 820

10.0727 801

10.0818 830

10.0908 836

10.0999 877

10.1090 825

10.1181 836

10.1272 834

10.1363 795

10.1454 838

10.1544 864

10.1635 843

10.1726 897

10.1817 890

10.1908 845

10.1999 805

10.2090 822

10.2180 794

10.2271 808

10.2362 884

10.2453 802

10.2544 850

10.2635 830

10.2725 796

10.2816 846

10.2907 811

10.2998 860

10.3089 824

10.3180 826

10.3271 782

10.3361 851

10.3452 859

10.3543 759

10.3634 859

10.3725 805

10.3816 764

10.3906 822

10.3997 880

10.4088 832

10.4179 827

10.4270 777

10.4361 806

10.4452 768

10.4542 859

10.4633 791

10.4724 838

10.4815 801

10.4906 842

10.4997 869

10.5088 833

10.5178 798

10.5269 827

10.5360 811

10.5451 798

10.5542 812

10.5633 819

10.5723 918

10.5814 840

10.5905 844

10.5996 818

10.6087 808

10.6178 839

10.6269 859

10.6359 836

10.6450 759

10.6541 809

10.6632 817

10.6723 795

10.6814 809

10.6904 804

10.6995 793

10.7086 854

10.7177 811

10.7268 836

10.7359 786

10.7450 805

10.7540 830

10.7631 797

10.7722 808

10.7813 781

10.7904 776

10.7995 846

10.8086 781

10.8176 795

10.8267 836

10.8358 866

10.8449 830

10.8540 816

10.8631 793

10.8721 796

10.8812 803

10.8903 784

10.8994 771

10.9085 788

10.9176 792

10.9267 811

10.9357 784

10.9448 826

10.9539 821

10.9630 801

10.9721 796

10.9812 802

10.9902 771

10.9993 807

11.0084 757

11.0175 773

11.0266 806

11.0357 765

11.0448 791

11.0538 765

11.0629 772

11.0720 817

11.0811 814

11.0902 746

11.0993 729

11.1084 763

11.1174 743

11.1265 776

11.1356 807

11.1447 803

11.1538 753

11.1629 776

11.1719 753

11.1810 823

11.1901 790

11.1992 771

11.2083 736

11.2174 771

11.2265 813

11.2355 829

11.2446 709

11.2537 759

11.2628 805

11.2719 786

11.2810 799

11.2900 771

11.2991 757

11.3082 813

11.3173 753

11.3264 724

11.3355 766

11.3446 788

11.3536 805

11.3627 745

11.3718 816

11.3809 817

11.3900 721

11.3991 793

11.4082 769

11.4172 772

11.4263 785

11.4354 796

11.4445 804

11.4536 793

11.4627 789

11.4717 786

11.4808 783

11.4899 770

11.4990 754

11.5081 782

11.5172 768

11.5263 824

11.5353 766

11.5444 762

11.5535 763

11.5626 833

11.5717 776

11.5808 760

11.5898 782

11.5989 768

11.6080 765

11.6171 781

11.6262 742

11.6353 777

11.6444 788

11.6534 764

11.6625 758

11.6716 780

11.6807 794

11.6898 781

11.6989 766

11.7080 710

11.7170 749

11.7261 744

11.7352 779

11.7443 773

11.7534 748

11.7625 737

11.7715 763

11.7806 785

11.7897 766

11.7988 755

11.8079 722

11.8170 758

11.8261 790

11.8351 781

11.8442 761

11.8533 727

11.8624 727

11.8715 742

11.8806 785

11.8896 764

11.8987 745

11.9078 800

11.9169 752

11.9260 768

11.9351 783

11.9442 746

11.9532 751

11.9623 754

11.9714 785

11.9805 709

11.9896 709

11.9987 749

12.0078 761

12.0168 787

12.0259 759

12.0350 758

12.0441 748

12.0532 787

12.0623 721

12.0713 744

12.0804 771

12.0895 726

12.0986 782

12.1077 774

12.1168 759

12.1259 725

12.1349 785

12.1440 781

12.1531 772

12.1622 772

12.1713 726

12.1804 715

12.1894 744

12.1985 772

12.2076 736

12.2167 690

12.2258 737

12.2349 704

12.2440 754

12.2530 723

12.2621 735

12.2712 660

12.2803 736

12.2894 713

12.2985 793

12.3076 745

12.3166 710

12.3257 784

12.3348 694

12.3439 738

12.3530 768

12.3621 733

12.3711 741

12.3802 719

12.3893 722

12.3984 744

12.4075 689

12.4166 711

12.4257 721

12.4347 781

12.4438 761

12.4529 754

12.4620 754

12.4711 752

12.4802 742

12.4892 764

12.4983 700

12.5074 711

12.5165 711

12.5256 721

12.5347 708

12.5438 688

12.5528 723

12.5619 714

12.5710 763

12.5801 745

12.5892 776

12.5983 721

12.6074 722

12.6164 709

12.6255 688

12.6346 700

12.6437 739

12.6528 715

12.6619 779

12.6709 734

12.6800 666

12.6891 721

12.6982 714

12.7073 755

12.7164 743

12.7255 722

12.7345 694

12.7436 707

12.7527 730

12.7618 746

12.7709 731

12.7800 722

12.7890 723

12.7981 694

12.8072 657

12.8163 711

12.8254 737

12.8345 738

12.8436 726

12.8526 757

12.8617 717

12.8708 731

12.8799 710

12.8890 690

12.8981 714

12.9072 743

12.9162 694

12.9253 730

12.9344 681

12.9435 709

12.9526 726

12.9617 679

12.9707 722

12.9798 724

12.9889 761

12.9980 688

13.0071 744

13.0162 711

13.0253 724

13.0343 640

13.0434 762

13.0525 697

13.0616 720

13.0707 681

13.0798 702

13.0888 706

13.0979 657

13.1070 680

13.1161 718

13.1252 711

13.1343 623

13.1434 737

13.1524 686

13.1615 691

13.1706 690

13.1797 704

13.1888 721

13.1979 699

13.2070 692

13.2160 658

13.2251 684

13.2342 688

13.2433 697

13.2524 744

13.2615 717

13.2705 759

13.2796 746

13.2887 699

13.2978 722

13.3069 725

13.3160 703

13.3251 689

13.3341 702

13.3432 653

13.3523 698

13.3614 723

13.3705 669

13.3796 691

13.3886 706

13.3977 693

13.4068 738

13.4159 729

13.4250 714

13.4341 707

13.4432 726

13.4522 672

13.4613 734

13.4704 700

13.4795 663

13.4886 715

13.4977 691

13.5068 720

13.5158 689

13.5249 676

13.5340 671

13.5431 665

13.5522 713

13.5613 710

13.5703 725

13.5794 721

13.5885 690

13.5976 710

13.6067 738

13.6158 751

13.6249 750

13.6339 652

13.6430 700

13.6521 697

13.6612 724

13.6703 663

13.6794 701

13.6884 749

13.6975 690

13.7066 696

13.7157 699

13.7248 719

13.7339 707

13.7430 719

13.7520 684

13.7611 689

13.7702 714

13.7793 730

13.7884 698

13.7975 702

13.8066 710

13.8156 714

13.8247 699

13.8338 687

13.8429 697

13.8520 741

13.8611 672

13.8701 740

13.8792 691

13.8883 704

13.8974 676

13.9065 731

13.9156 706

13.9247 741

13.9337 743

13.9428 745

13.9519 698

13.9610 712

13.9701 734

13.9792 749

13.9882 708

13.9973 743

14.0064 709

14.0155 701

14.0246 693

14.0337 695

14.0428 702

14.0518 731

14.0609 740

14.0700 702

14.0791 699

14.0882 711

14.0973 692

14.1064 733

14.1154 680

14.1245 693

14.1336 680

14.1427 697

14.1518 684

14.1609 697

14.1699 660

14.1790 679

14.1881 741

14.1972 691

14.2063 661

14.2154 679

14.2245 673

14.2335 673

14.2426 686

14.2517 707

14.2608 657

14.2699 708

14.2790 738

14.2880 656

14.2971 662

14.3062 707

14.3153 646

14.3244 718

14.3335 709

14.3426 701

14.3516 756

14.3607 743

14.3698 688

14.3789 704

14.3880 693

14.3971 621

14.4062 674

14.4152 700

14.4243 700

14.4334 661

14.4425 714

14.4516 682

14.4607 694

14.4697 722

14.4788 692

14.4879 686

14.4970 710

14.5061 722

14.5152 738

14.5243 694

14.5333 709

14.5424 720

14.5515 679

14.5606 731

14.5697 706

14.5788 755

14.5878 725

14.5969 685

14.6060 692

14.6151 706

14.6242 697

14.6333 692

14.6424 712

14.6514 683

14.6605 688

14.6696 670

14.6787 721

14.6878 750

14.6969 667

14.7060 716

14.7150 671

14.7241 682

14.7332 689

14.7423 714

14.7514 694

14.7605 700

14.7695 691

14.7786 690

14.7877 695

14.7968 689

14.8059 693

14.8150 698

14.8241 687

14.8331 690

14.8422 691

14.8513 791

14.8604 691

14.8695 686

14.8786 738

14.8876 672

14.8967 698

14.9058 658

14.9149 695

14.9240 658

14.9331 709

14.9422 675

14.9512 630

14.9603 700

14.9694 661

14.9785 720

14.9876 640

14.9967 706

15.0058 697

15.0148 632

15.0239 733

15.0330 674

15.0421 732

15.0512 678

15.0603 751

15.0693 724

15.0784 669

15.0875 692

15.0966 720

15.1057 708

15.1148 704

15.1239 694

15.1329 665

15.1420 661

15.1511 730

15.1602 744

15.1693 730

15.1784 689

15.1874 657

15.1965 678

15.2056 712

15.2147 687

15.2238 687

15.2329 687

15.2420 707

15.2510 704

15.2601 693

15.2692 664

15.2783 702

15.2874 687

15.2965 741

15.3056 675

15.3146 687

15.3237 700

15.3328 666

15.3419 699

15.3510 685

15.3601 691

15.3691 689

15.3782 730

15.3873 698

15.3964 728

15.4055 685

15.4146 664

15.4237 732

15.4327 704

15.4418 675

15.4509 638

15.4600 721

15.4691 698

15.4782 681

15.4872 664

15.4963 700

15.5054 689

15.5145 676

15.5236 703

15.5327 697

15.5418 702

15.5508 717

15.5599 733

15.5690 697

15.5781 733

15.5872 700

15.5963 727

15.6054 637

15.6144 691

15.6235 738

15.6326 685

15.6417 689

15.6508 657

15.6599 671

15.6689 728

15.6780 645

15.6871 701

15.6962 694

15.7053 684

15.7144 707

15.7235 717

15.7325 667

15.7416 657

15.7507 650

15.7598 678

15.7689 680

15.7780 676

15.7870 702

15.7961 684

15.8052 671

15.8143 679

15.8234 688

15.8325 663

15.8416 757

15.8506 693

15.8597 723

15.8688 688

15.8779 686

15.8870 750

15.8961 692

15.9052 731

15.9142 706

15.9233 723

15.9324 688

15.9415 665

15.9506 686

15.9597 689

15.9687 737

15.9778 699

15.9869 738

15.9960 669

16.0051 707

16.0142 688

16.0233 707

16.0323 703

16.0414 658

16.0505 741

16.0596 699

16.0687 698

16.0778 681

16.0868 718

16.0959 642

16.1050 729

16.1141 692

16.1232 682

16.1323 705

16.1414 695

16.1504 659

16.1595 704

16.1686 702

16.1777 689

16.1868 712

16.1959 675

16.2050 714

16.2140 690

16.2231 666

16.2322 666

16.2413 682

16.2504 760

16.2595 728

16.2685 673

16.2776 629

16.2867 696

16.2958 688

16.3049 641

16.3140 654

16.3231 716

16.3321 639

16.3412 676

16.3503 661

16.3594 671

16.3685 687

16.3776 702

16.3866 655

16.3957 692

16.4048 668

16.4139 657

16.4230 698

16.4321 690

16.4412 689

16.4502 666

16.4593 677

16.4684 663

16.4775 649

16.4866 673

16.4957 719

16.5048 660

16.5138 672

16.5229 679

16.5320 667

16.5411 710

16.5502 693

16.5593 674

16.5683 652

16.5774 687

16.5865 690

16.5956 709

16.6047 693

16.6138 691

16.6229 655

16.6319 656

16.6410 671

16.6501 689

16.6592 714

16.6683 731

16.6774 673

16.6864 704

16.6955 689

16.7046 672

16.7137 685

16.7228 673

16.7319 683

16.7410 683

16.7500 723

16.7591 686

16.7682 720

16.7773 661

16.7864 674

16.7955 679

16.8046 713

16.8136 756

16.8227 666

16.8318 699

16.8409 717

16.8500 696

16.8591 690

16.8681 687

16.8772 655

16.8863 636

16.8954 708

16.9045 698

16.9136 664

16.9227 688

16.9317 663

16.9408 645

16.9499 664

16.9590 697

16.9681 689

16.9772 680

16.9862 649

16.9953 692

17.0044 685

17.0135 694

17.0226 696

17.0317 681

17.0408 722

17.0498 692

17.0589 680

17.0680 697

17.0771 721

17.0862 693

17.0953 701

17.1044 674

17.1134 707

17.1225 713

17.1316 696

17.1407 715

17.1498 681

17.1589 653

17.1679 661

17.1770 664

17.1861 706

17.1952 718

17.2043 657

17.2134 662

17.2225 713

17.2315 653

17.2406 656

17.2497 651

17.2588 692

17.2679 634

17.2770 716

17.2860 682

17.2951 716

17.3042 657

17.3133 694

17.3224 656

17.3315 652

17.3406 656

17.3496 681

17.3587 635

17.3678 655

17.3769 711

17.3860 703

17.3951 696

17.4042 674

17.4132 734

17.4223 670

17.4314 684

17.4405 693

17.4496 670

17.4587 690

17.4677 707

17.4768 649

17.4859 641

17.4950 636

17.5041 685

17.5132 701

17.5223 747

17.5313 651

17.5404 663

17.5495 720

17.5586 648

17.5677 650

17.5768 728

17.5858 657

17.5949 673

17.6040 688

17.6131 689

17.6222 711

17.6313 702

17.6404 710

17.6494 705

17.6585 727

17.6676 681

17.6767 736

17.6858 658

17.6949 660

17.7040 731

17.7130 717

17.7221 690

17.7312 720

17.7403 675

17.7494 714

17.7585 698

17.7675 695

17.7766 700

17.7857 662

17.7948 691

17.8039 670

17.8130 660

17.8221 744

17.8311 704

17.8402 712

17.8493 649

17.8584 651

17.8675 673

17.8766 676

17.8856 656

17.8947 687

17.9038 654

17.9129 679

17.9220 741

17.9311 712

17.9402 654

17.9492 691

17.9583 680

17.9674 701

17.9765 675

17.9856 694

17.9947 694

18.0038 684

18.0128 740

18.0219 703

18.0310 712

18.0401 706

18.0492 684

18.0583 737

18.0673 740

18.0764 714

18.0855 665

18.0946 674

18.1037 686

18.1128 715

18.1219 668

18.1309 714

18.1400 710

18.1491 713

18.1582 639

18.1673 684

18.1764 699

18.1854 665

18.1945 696

18.2036 677

18.2127 716

18.2218 702

18.2309 697

18.2400 717

18.2490 683

18.2581 728

18.2672 703

18.2763 650

18.2854 682

18.2945 693

18.3036 701

18.3126 701

18.3217 674

18.3308 699

18.3399 651

18.3490 705

18.3581 709

18.3671 693

18.3762 664

18.3853 672

18.3944 692

18.4035 699

18.4126 666

18.4217 643

18.4307 689

18.4398 703

18.4489 661

18.4580 721

18.4671 685

18.4762 717

18.4852 701

18.4943 740

18.5034 648

18.5125 652

18.5216 665

18.5307 635

18.5398 741

18.5488 691

18.5579 719

18.5670 712

18.5761 718

18.5852 670

18.5943 722

18.6034 691

18.6124 708

18.6215 691

18.6306 659

18.6397 705

18.6488 698

18.6579 674

18.6669 653

18.6760 719

18.6851 672

18.6942 747

18.7033 657

18.7124 682

18.7215 699

18.7305 721

18.7396 714

18.7487 669

18.7578 696

18.7669 687

18.7760 665

18.7850 663

18.7941 690

18.8032 713

18.8123 701

18.8214 738

18.8305 661

18.8396 685

18.8486 670

18.8577 686

18.8668 678

18.8759 660

18.8850 707

18.8941 712

18.9032 659

18.9122 663

18.9213 696

18.9304 759

18.9395 716

18.9486 667

18.9577 696

18.9667 690

18.9758 723

18.9849 694

18.9940 703

19.0031 656

19.0122 705

19.0213 716

19.0303 677

19.0394 689

19.0485 627

19.0576 674

19.0667 720

19.0758 696

19.0848 718

19.0939 721

19.1030 707

19.1121 686

19.1212 682

19.1303 641

19.1394 692

19.1484 661

19.1575 701

19.1666 747

19.1757 730

19.1848 692

19.1939 705

19.2030 687

19.2120 658

19.2211 745

19.2302 719

19.2393 747

19.2484 691

19.2575 727

19.2665 702

19.2756 675

19.2847 708

19.2938 749

19.3029 690

19.3120 693

19.3211 653

19.3301 676

19.3392 705

19.3483 684

19.3574 670

19.3665 685

19.3756 687

19.3847 732

19.3937 715

19.4028 675

19.4119 666

19.4210 681

19.4301 688

19.4392 711

19.4482 679

19.4573 690

19.4664 687

19.4755 644

19.4846 661

19.4937 699

19.5028 709

19.5118 662

19.5209 645

19.5300 690

19.5391 689

19.5482 722

19.5573 675

19.5663 682

19.5754 691

19.5845 738

19.5936 665

19.6027 693

19.6118 712

19.6209 660

19.6299 705

19.6390 737

19.6481 678

19.6572 671

19.6663 625

19.6754 730

19.6845 729

19.6935 720

19.7026 739

19.7117 704

19.7208 671

19.7299 678

19.7390 670

19.7480 660

19.7571 717

19.7662 766

19.7753 683

19.7844 723

19.7935 725

19.8026 664

19.8116 647

19.8207 703

19.8298 701

19.8389 682

19.8480 661

19.8571 699

19.8661 699

19.8752 685

19.8843 704

19.8934 715

19.9025 702

19.9116 654

19.9207 655

19.9297 657

19.9388 678

19.9479 670

19.9570 732

19.9661 711

19.9752 699

19.9843 741

19.9933 715

20.0024 677

20.0115 653

20.0206 656

20.0297 685

20.0388 675

20.0478 681

20.0569 719

20.0660 672

20.0751 668

20.0842 706

20.0933 669

20.1024 653

20.1114 721

20.1205 677

20.1296 719

20.1387 652

20.1478 677

20.1569 694

20.1659 720

20.1750 715

20.1841 701

20.1932 686

20.2023 684

20.2114 681

20.2205 677

20.2295 652

20.2386 762

20.2477 688

20.2568 745

20.2659 672

20.2750 676

20.2841 646

20.2931 664

20.3022 669

20.3113 730

20.3204 696

20.3295 736

20.3386 682

20.3476 628

20.3567 675

20.3658 670

20.3749 679

20.3840 684

20.3931 667

20.4022 660

20.4112 671

20.4203 700

20.4294 670

20.4385 652

20.4476 674

20.4567 711

20.4657 682

20.4748 685

20.4839 662

20.4930 733

20.5021 687

20.5112 640

20.5203 669

20.5293 741

20.5384 761

20.5475 711

20.5566 691

20.5657 711

20.5748 680

20.5839 674

20.5929 693

20.6020 688

20.6111 707

20.6202 733

20.6293 643

20.6384 691

20.6474 704

20.6565 697

20.6656 672

20.6747 696

20.6838 698

20.6929 711

20.7020 663

20.7110 654

20.7201 682

20.7292 698

20.7383 713

20.7474 690

20.7565 678

20.7655 673

20.7746 686

20.7837 596

20.7928 699

20.8019 682

20.8110 714

20.8201 696

20.8291 676

20.8382 667

20.8473 697

20.8564 690

20.8655 684

20.8746 686

20.8837 673

20.8927 720

20.9018 685

20.9109 743

20.9200 626

20.9291 672

20.9382 726

20.9472 681

20.9563 626

20.9654 670

20.9745 695

20.9836 701

20.9927 678

21.0018 690

21.0108 702

21.0199 701

21.0290 674

21.0381 699

21.0472 688

21.0563 665

21.0653 649

21.0744 655

21.0835 674

21.0926 708

21.1017 666

21.1108 679

21.1199 708

21.1289 608

21.1380 681

21.1471 665

21.1562 681

21.1653 727

21.1744 680

21.1835 712

21.1925 681

21.2016 674

21.2107 695

21.2198 697

21.2289 639

21.2380 722

21.2470 684

21.2561 678

21.2652 704

21.2743 742

21.2834 643

21.2925 698

21.3016 699

21.3106 673

21.3197 697

21.3288 688

21.3379 677

21.3470 647

21.3561 612

21.3651 697

21.3742 736

21.3833 683

21.3924 668

21.4015 704

21.4106 691

21.4197 690

21.4287 664

21.4378 684

21.4469 715

21.4560 692

21.4651 674

21.4742 671

21.4833 699

21.4923 686

21.5014 718

21.5105 694

21.5196 671

21.5287 686

21.5378 666

21.5468 643

21.5559 681

21.5650 686

21.5741 666

21.5832 674

21.5923 646

21.6014 687

21.6104 670

21.6195 692

21.6286 672

21.6377 695

21.6468 678

21.6559 671

21.6649 657

21.6740 680

21.6831 640

21.6922 713

21.7013 731

21.7104 676

21.7195 691

21.7285 705

21.7376 687

21.7467 684

21.7558 670

21.7649 726

21.7740 676

21.7831 684

21.7921 649

21.8012 687

21.8103 670

21.8194 655

21.8285 699

21.8376 672

21.8466 677

21.8557 695

21.8648 674

21.8739 678

21.8830 674

21.8921 653

21.9012 632

21.9102 641

21.9193 723

21.9284 641

21.9375 715

21.9466 653

21.9557 675

21.9647 724

21.9738 668

21.9829 663

21.9920 745

22.0011 658

22.0102 669

22.0193 708

22.0283 678

22.0374 649

22.0465 676

22.0556 625

22.0647 689

22.0738 646

22.0829 693

22.0919 655

22.1010 626

22.1101 657

22.1192 689

22.1283 665

22.1374 664

22.1464 697

22.1555 681

22.1646 657

22.1737 685

22.1828 732

22.1919 703

22.2010 713

22.2100 654

22.2191 663

22.2282 692

22.2373 719

22.2464 672

22.2555 667

22.2645 663

22.2736 645

22.2827 673

22.2918 657

22.3009 685

22.3100 640

22.3191 702

22.3281 692

22.3372 716

22.3463 686

22.3554 687

22.3645 676

22.3736 631

22.3827 663

22.3917 644

22.4008 690

22.4099 694

22.4190 684

22.4281 723

22.4372 660

22.4462 706

22.4553 710

22.4644 649

22.4735 640

22.4826 699

22.4917 712

22.5008 670

22.5098 703

22.5189 721

22.5280 674

22.5371 680

22.5462 623

22.5553 653

22.5643 647

22.5734 672

22.5825 687

22.5916 675

22.6007 668

22.6098 656

22.6189 683

22.6279 680

22.6370 699

22.6461 689

22.6552 686

22.6643 686

22.6734 619

22.6825 686

22.6915 677

22.7006 640

22.7097 730

22.7188 661

22.7279 692

22.7370 684

22.7460 683

22.7551 659

22.7642 681

22.7733 687

22.7824 685

22.7915 702

22.8006 671

22.8096 695

22.8187 690

22.8278 677

22.8369 641

22.8460 708

22.8551 683

22.8641 668

22.8732 715

22.8823 667

22.8914 682

22.9005 672

22.9096 655

22.9187 665

22.9277 702

22.9368 703

22.9459 638

22.9550 674

22.9641 700

22.9732 662

22.9823 683

22.9913 617

23.0004 650

23.0095 663

23.0186 658

23.0277 678

23.0368 639

23.0458 688

23.0549 682

23.0640 696

23.0731 666

23.0822 650

23.0913 637

23.1004 658

23.1094 704

23.1185 702

23.1276 641

23.1367 656

23.1458 707

23.1549 716

23.1639 678

23.1730 675

23.1821 670

23.1912 669

23.2003 692

23.2094 723

23.2185 672

23.2275 696

23.2366 695

23.2457 635

23.2548 672

23.2639 612

23.2730 627

23.2821 682

23.2911 671

23.3002 683

23.3093 652

23.3184 657

23.3275 708

23.3366 681

23.3456 657

23.3547 639

23.3638 658

23.3729 677

23.3820 690

23.3911 627

23.4002 706

23.4092 645

23.4183 675

23.4274 701

23.4365 709

23.4456 692

23.4547 682

23.4637 646

23.4728 659

23.4819 685

23.4910 621

23.5001 683

23.5092 697

23.5183 687

23.5273 678

23.5364 674

23.5455 676

23.5546 596

23.5637 642

23.5728 699

23.5819 709

23.5909 674

23.6000 622

23.6091 689

23.6182 623

23.6273 713

23.6364 643

23.6454 659

23.6545 672

23.6636 677

23.6727 663

23.6818 708

23.6909 679

23.7000 721

23.7090 674

23.7181 674

23.7272 632

23.7363 683

23.7454 654

23.7545 732

23.7635 646

23.7726 713

23.7817 698

23.7908 680

23.7999 682

23.8090 696

23.8181 699

23.8271 712

23.8362 665

23.8453 704

23.8544 674

23.8635 675

23.8726 650

23.8817 672

23.8907 650

23.8998 714

23.9089 682

23.9180 729

23.9271 661

23.9362 686

23.9452 663

23.9543 672

23.9634 706

23.9725 717

23.9816 681

23.9907 637

23.9998 696

24.0088 684

24.0179 697

24.0270 662

24.0361 698

24.0452 689

24.0543 705

24.0633 721

24.0724 681

24.0815 688

24.0906 658

24.0997 719

24.1088 697

24.1179 658

24.1269 656

24.1360 690

24.1451 650

24.1542 713

24.1633 663

24.1724 708

24.1815 669

24.1905 678

24.1996 699

24.2087 684

24.2178 658

24.2269 675

24.2360 686

24.2450 710

24.2541 632

24.2632 686

24.2723 706

24.2814 644

24.2905 644

24.2996 677

24.3086 656

24.3177 631

24.3268 708

24.3359 692

24.3450 673

24.3541 629

24.3631 661

24.3722 680

24.3813 638

24.3904 665

24.3995 673

24.4086 681

24.4177 695

24.4267 732

24.4358 654

24.4449 644

24.4540 700

24.4631 669

24.4722 688

24.4813 690

24.4903 683

24.4994 686

24.5085 706

24.5176 709

24.5267 643

24.5358 682

24.5448 643

24.5539 644

24.5630 691

24.5721 664

24.5812 690

24.5903 655

24.5994 664

24.6084 697

24.6175 654

24.6266 653

24.6357 699

24.6448 695

24.6539 674

24.6629 717

24.6720 658

24.6811 677

24.6902 699

24.6993 692

24.7084 672

24.7175 679

24.7265 647

24.7356 665

24.7447 671

24.7538 697

24.7629 714

24.7720 702

24.7811 674

24.7901 651

24.7992 731

24.8083 646

24.8174 719

24.8265 655

24.8356 665

24.8446 683

24.8537 637

24.8628 720

24.8719 684

24.8810 677

24.8901 663

24.8992 654

24.9082 671

24.9173 675

24.9264 657

24.9355 671

24.9446 630

24.9537 698

24.9627 653

24.9718 690

24.9809 696

24.9900 667

24.9991 679

25.0082 680

25.0173 676

25.0263 668

25.0354 668

25.0445 654

25.0536 672

25.0627 660

25.0718 673

25.0809 702

25.0899 685

25.0990 742

25.1081 683

25.1172 641

25.1263 715

25.1354 662

25.1444 730

25.1535 678

25.1626 685

25.1717 712

25.1808 705

25.1899 664

25.1990 700

25.2080 653

25.2171 656

25.2262 676

25.2353 684

25.2444 702

25.2535 742

25.2625 691

25.2716 670

25.2807 658

25.2898 714

25.2989 665

25.3080 679

25.3171 691

25.3261 697

25.3352 677

25.3443 657

25.3534 705

25.3625 653

25.3716 763

25.3807 658

25.3897 744

25.3988 743

25.4079 753

25.4170 667

25.4261 663

25.4352 716

25.4442 639

25.4533 638

25.4624 674

25.4715 700

25.4806 676

25.4897 658

25.4988 661

25.5078 705

25.5169 683

25.5260 677

25.5351 718

25.5442 711

25.5533 671

25.5623 679

25.5714 673

25.5805 617

25.5896 676

25.5987 669

25.6078 654

25.6169 744

25.6259 686

25.6350 672

25.6441 679

25.6532 653

25.6623 655

25.6714 653

25.6805 648

25.6895 617

25.6986 657

25.7077 684

25.7168 697

25.7259 654

25.7350 671

25.7440 683

25.7531 647

25.7622 709

25.7713 684

25.7804 705

25.7895 650

25.7986 663

25.8076 704

25.8167 722

25.8258 678

25.8349 719

25.8440 656

25.8531 711

25.8621 697

25.8712 705

25.8803 672

25.8894 683

25.8985 647

25.9076 692

25.9167 609

25.9257 732

25.9348 685

25.9439 718

25.9530 663

25.9621 660

25.9712 709

25.9803 691

25.9893 665

25.9984 688

26.0075 721

26.0166 637

26.0257 693

26.0348 699

26.0438 667

26.0529 655

26.0620 677

26.0711 664

26.0802 666

26.0893 683

26.0984 715

26.1074 688

26.1165 694

26.1256 650

26.1347 691

26.1438 644

26.1529 702

26.1619 680

26.1710 708

26.1801 684

26.1892 697

26.1983 646

26.2074 730

26.2165 688

26.2255 639

26.2346 704

26.2437 683

26.2528 673

26.2619 705

26.2710 684

26.2801 682

26.2891 698

26.2982 706

26.3073 656

26.3164 675

26.3255 706

26.3346 686

26.3436 717

26.3527 637

26.3618 672

26.3709 663

26.3800 685

26.3891 687

26.3982 696

26.4072 669

26.4163 704

26.4254 697

26.4345 695

26.4436 680

26.4527 676

26.4617 677

26.4708 637

26.4799 686

26.4890 684

26.4981 696

26.5072 682

26.5163 661

26.5253 709

26.5344 670

26.5435 656

26.5526 665

26.5617 682

26.5708 693

26.5799 679

26.5889 647

26.5980 722

26.6071 675

26.6162 664

26.6253 700

26.6344 666

26.6434 682

26.6525 656

26.6616 681

26.6707 683

26.6798 702

26.6889 672

26.6980 669

26.7070 700

26.7161 655

26.7252 656

26.7343 659

26.7434 662

26.7525 649

26.7615 708

26.7706 678

26.7797 636

26.7888 676

26.7979 696

26.8070 684

26.8161 703

26.8251 668

26.8342 719

26.8433 697

26.8524 662

26.8615 709

26.8706 688

26.8797 689

26.8887 684

26.8978 668

26.9069 694

26.9160 706

26.9251 739

26.9342 671

26.9432 691

26.9523 661

26.9614 686

26.9705 687

26.9796 676

26.9887 739

26.9978 697

27.0068 712

27.0159 727

27.0250 671

27.0341 712

27.0432 690

27.0523 689

27.0613 684

27.0704 647

27.0795 666

27.0886 714

27.0977 660

27.1068 675

27.1159 653

27.1249 728

27.1340 660

27.1431 710

27.1522 722

27.1613 661

27.1704 689

27.1795 696

27.1885 670

27.1976 681

27.2067 665

27.2158 680

27.2249 693

27.2340 654

27.2430 726

27.2521 656

27.2612 714

27.2703 676

27.2794 676

27.2885 693

27.2976 658

27.3066 680

27.3157 653

27.3248 749

27.3339 705

27.3430 700

27.3521 694

27.3611 704

27.3702 706

27.3793 682

27.3884 712

27.3975 668

27.4066 667

27.4157 664

27.4247 685

27.4338 709

27.4429 746

27.4520 671

27.4611 713

27.4702 690

27.4793 672

27.4883 672

27.4974 647

27.5065 749

27.5156 703

27.5247 703

27.5338 674

27.5428 701

27.5519 656

27.5610 714

27.5701 743

27.5792 695

27.5883 669

27.5974 697

27.6064 678

27.6155 692

27.6246 669

27.6337 691

27.6428 681

27.6519 722

27.6609 712

27.6700 672

27.6791 704

27.6882 720

27.6973 662

27.7064 727

27.7155 664

27.7245 686

27.7336 658

27.7427 717

27.7518 725

27.7609 677

27.7700 713

27.7791 704

27.7881 708

27.7972 687

27.8063 694

27.8154 704

27.8245 710

27.8336 731

27.8426 729

27.8517 707

27.8608 745

27.8699 666

27.8790 753

27.8881 688

27.8972 681

27.9062 709

27.9153 696

27.9244 744

27.9335 713

27.9426 745

27.9517 718

27.9607 737

27.9698 687

27.9789 745

27.9880 688

27.9971 731

28.0062 705

28.0153 735

28.0243 734

28.0334 657

28.0425 674

28.0516 686

28.0607 705

28.0698 719

28.0789 738

28.0879 675

28.0970 671

28.1061 729

28.1152 742

28.1243 676

28.1334 691

28.1424 688

28.1515 710

28.1606 721

28.1697 698

28.1788 694

28.1879 691

28.1970 710

28.2060 654

28.2151 664

28.2242 695

28.2333 690

28.2424 728

28.2515 696

28.2605 705

28.2696 676

28.2787 661

28.2878 681

28.2969 717

28.3060 681

28.3151 762

28.3241 651

28.3332 691

28.3423 685

28.3514 719

28.3605 708

28.3696 721

28.3787 707

28.3877 724

28.3968 717

28.4059 682

28.4150 698

28.4241 703

28.4332 689

28.4422 704

28.4513 704

28.4604 718

28.4695 665

28.4786 740

28.4877 717

28.4968 687

28.5058 714

28.5149 651

28.5240 693

28.5331 718

28.5422 707

28.5513 731

28.5603 694

28.5694 696

28.5785 648

28.5876 701

28.5967 723

28.6058 688

28.6149 679

28.6239 716

28.6330 656

28.6421 658

28.6512 720

28.6603 675

28.6694 703

28.6785 691

28.6875 756

28.6966 672

28.7057 689

28.7148 707

28.7239 724

28.7330 682

28.7420 679

28.7511 714

28.7602 651

28.7693 674

28.7784 750

28.7875 734

28.7966 733

28.8056 683

28.8147 656

28.8238 669

28.8329 695

28.8420 706

28.8511 671

28.8601 718

28.8692 769

28.8783 687

28.8874 732

28.8965 735

28.9056 684

28.9147 709

28.9237 699

28.9328 684

28.9419 697

28.9510 694

28.9601 693

28.9692 687

28.9783 672

28.9873 695

28.9964 669

29.0055 692

29.0146 686

29.0237 684

29.0328 683

29.0418 724

29.0509 711

29.0600 644

29.0691 692

29.0782 710

29.0873 688

29.0964 744

29.1054 710

29.1145 718

29.1236 697

29.1327 626

29.1418 648

29.1509 683

29.1599 705

29.1690 676

29.1781 676

29.1872 699

29.1963 730

29.2054 670

29.2145 725

29.2235 682

29.2326 655

29.2417 679

29.2508 668

29.2599 710

29.2690 721

29.2781 690

29.2871 713

29.2962 714

29.3053 686

29.3144 657

29.3235 735

29.3326 670

29.3416 636

29.3507 706

29.3598 714

29.3689 690

29.3780 690

29.3871 681

29.3962 656

29.4052 667

29.4143 691

29.4234 702

29.4325 682

29.4416 691

29.4507 683

29.4597 595

29.4688 637

29.4779 698

29.4870 691

29.4961 691

29.5052 679

29.5143 674

29.5233 657

29.5324 655

29.5415 701

29.5506 659

29.5597 676

29.5688 669

29.5779 682

29.5869 675

29.5960 687

29.6051 704

29.6142 690

29.6233 675

29.6324 682

29.6414 698

29.6505 709

29.6596 689

29.6687 658

29.6778 646

29.6869 687

29.6960 732

29.7050 672

29.7141 704

29.7232 670

29.7323 673

29.7414 629

29.7505 662

29.7595 699

29.7686 655

29.7777 673

29.7868 647

29.7959 688

29.8050 657

29.8141 726

29.8231 675

29.8322 708

29.8413 697

29.8504 668

29.8595 645

29.8686 717

29.8777 664

29.8867 638

29.8958 657

29.9049 665

29.9140 653

29.9231 645

29.9322 658

29.9412 668

29.9503 676

29.9594 682

29.9685 716

29.9776 699

29.9867 695

29.9958 632

30.0048 631

30.0139 674

30.0230 646

30.0321 674

30.0412 643

30.0503 661

30.0593 684

30.0684 672

30.0775 692

30.0866 695

30.0957 686

30.1048 655

30.1139 632

30.1229 674

30.1320 646

30.1411 627

30.1502 668

30.1593 684

30.1684 649

30.1775 625

30.1865 692

30.1956 654

30.2047 712

30.2138 675

30.2229 654

30.2320 667

30.2410 677

30.2501 690

30.2592 727

30.2683 669

30.2774 666

30.2865 675

30.2956 620

30.3046 712

30.3137 709

30.3228 679

30.3319 666

30.3410 705

30.3501 694

30.3591 674

30.3682 648

30.3773 685

30.3864 684

30.3955 605

30.4046 650

30.4137 673

30.4227 629

30.4318 661

30.4409 683

30.4500 700

30.4591 650

30.4682 676

30.4773 690

30.4863 661

30.4954 663

30.5045 717

30.5136 701

30.5227 715

30.5318 666

30.5408 677

30.5499 652

30.5590 692

30.5681 667

30.5772 682

30.5863 625

30.5954 675

30.6044 685

30.6135 657

30.6226 671

30.6317 651

30.6408 668

30.6499 662

30.6589 670

30.6680 637

30.6771 661

30.6862 679

30.6953 656

30.7044 658

30.7135 652

30.7225 663

30.7316 657

30.7407 676

30.7498 617

30.7589 638

30.7680 654

30.7771 719

30.7861 663

30.7952 682

30.8043 657

30.8134 737

30.8225 635

30.8316 687

30.8406 654

30.8497 659

30.8588 669

30.8679 680

30.8770 666

30.8861 723

30.8952 679

30.9042 701

30.9133 681

30.9224 677

30.9315 705

30.9406 666

30.9497 648

30.9587 701

30.9678 664

30.9769 656

30.9860 722

30.9951 687

31.0042 654

31.0133 650

31.0223 715

31.0314 666

31.0405 686

31.0496 669

31.0587 718

31.0678 673

31.0769 674

31.0859 637

31.0950 655

31.1041 673

31.1132 696

31.1223 693

31.1314 699

31.1404 671

31.1495 675

31.1586 673

31.1677 700

31.1768 702

31.1859 648

31.1950 697

31.2040 689

31.2131 686

31.2222 720

31.2313 669

31.2404 621

31.2495 685

31.2585 679

31.2676 675

31.2767 684

31.2858 702

31.2949 672

31.3040 711

31.3131 680

31.3221 655

31.3312 665

31.3403 668

31.3494 707

31.3585 683

31.3676 768

31.3767 708

31.3857 694

31.3948 714

31.4039 671

31.4130 697

31.4221 699

31.4312 708

31.4402 702

31.4493 727

31.4584 717

31.4675 674

31.4766 717

31.4857 719

31.4948 734

31.5038 724

31.5129 711

31.5220 699

31.5311 717

31.5402 713

31.5493 719

31.5583 765

31.5674 735

31.5765 706

31.5856 732

31.5947 713

31.6038 734

31.6129 685

31.6219 771

31.6310 680

31.6401 742

31.6492 695

31.6583 714

31.6674 758

31.6765 746

31.6855 703

31.6946 758

31.7037 742

31.7128 716

31.7219 727

31.7310 727

31.7400 771

31.7491 716

31.7582 763

31.7673 717

31.7764 815

31.7855 763

31.7946 742

31.8036 713

31.8127 766

31.8218 748

31.8309 715

31.8400 769

31.8491 732

31.8581 720

31.8672 771

31.8763 750

31.8854 794

31.8945 735

31.9036 711

31.9127 795

31.9217 759

31.9308 746

31.9399 756

31.9490 753

31.9581 788

31.9672 768

31.9763 763

31.9853 703

31.9944 746

32.0035 711

32.0126 726

32.0217 736

32.0308 702

32.0398 748

32.0489 710

32.0580 777

32.0671 761

32.0762 734

32.0853 762

32.0944 742

32.1034 719

32.1125 730

32.1216 689

32.1307 711

32.1398 733

32.1489 677

32.1579 736

32.1670 750

32.1761 673

32.1852 728

32.1943 730

32.2034 744

32.2125 745

32.2215 740

32.2306 738

32.2397 713

32.2488 668

32.2579 697

32.2670 724

32.2761 714

32.2851 712

32.2942 721

32.3033 691

32.3124 689

32.3215 685

32.3306 685

32.3396 667

32.3487 699

32.3578 729

32.3669 718

32.3760 727

32.3851 706

32.3942 675

32.4032 734

32.4123 696

32.4214 650

32.4305 671

32.4396 694

32.4487 735

32.4577 690

32.4668 704

32.4759 672

32.4850 718

32.4941 704

32.5032 663

32.5123 645

32.5213 664

32.5304 664

32.5395 676

32.5486 682

32.5577 689

32.5668 710

32.5759 652

32.5849 690

32.5940 679

32.6031 667

32.6122 724

32.6213 636

32.6304 691

32.6394 634

32.6485 677

32.6576 671

32.6667 670

32.6758 710

32.6849 660

32.6940 673

32.7030 674

32.7121 660

32.7212 687

32.7303 654

32.7394 689

32.7485 654

32.7575 648

32.7666 645

32.7757 662

32.7848 645

32.7939 637

32.8030 643

32.8121 726

32.8211 717

32.8302 687

32.8393 688

32.8484 695

32.8575 650

32.8666 648

32.8757 701

32.8847 657

32.8938 739

32.9029 666

32.9120 665

32.9211 700

32.9302 703

32.9392 658

32.9483 676

32.9574 645

32.9665 697

32.9756 659

32.9847 685

32.9938 683

33.0028 633

33.0119 731

33.0210 709

33.0301 737

33.0392 688

33.0483 713

33.0573 674

33.0664 660

33.0755 713

33.0846 668

33.0937 675

33.1028 680

33.1119 688

33.1209 642

33.1300 719

33.1391 652

33.1482 640

33.1573 664

33.1664 683

33.1755 666

33.1845 664

33.1936 640

33.2027 672

33.2118 708

33.2209 625

33.2300 659

33.2390 657

33.2481 673

33.2572 684

33.2663 721

33.2754 668

33.2845 677

33.2936 703

33.3026 675

33.3117 718

33.3208 666

33.3299 713

33.3390 676

33.3481 657

33.3571 680

33.3662 711

33.3753 650

33.3844 673

33.3935 665

33.4026 671

33.4117 709

33.4207 665

33.4298 668

33.4389 676

33.4480 678

33.4571 703

33.4662 693

33.4753 641

33.4843 718

33.4934 698

33.5025 626

33.5116 679

33.5207 671

33.5298 657

33.5388 666

33.5479 700

33.5570 698

33.5661 666

33.5752 640

33.5843 648

33.5934 721

33.6024 691

33.6115 678

33.6206 702

33.6297 650

33.6388 687

33.6479 668

33.6569 700

33.6660 734

33.6751 685

33.6842 689

33.6933 667

33.7024 670

33.7115 723

33.7205 701

33.7296 696

33.7387 711

33.7478 693

33.7569 680

33.7660 669

33.7751 724

33.7841 718

33.7932 671

33.8023 725

33.8114 690

33.8205 729

33.8296 669

33.8386 657

33.8477 706

33.8568 669

33.8659 679

33.8750 713

33.8841 749

33.8932 660

33.9022 640

33.9113 678

33.9204 662

33.9295 664

33.9386 654

33.9477 748

33.9567 728

33.9658 672

33.9749 700

33.9840 696

33.9931 692

34.0022 684

34.0113 687

34.0203 714

34.0294 681

34.0385 728

34.0476 717

34.0567 672

34.0658 677

34.0749 703

34.0839 660

34.0930 669

34.1021 664

34.1112 666

34.1203 746

34.1294 678

34.1384 714

34.1475 701

34.1566 738

34.1657 669

34.1748 730

34.1839 668

34.1930 698

34.2020 686

34.2111 691

34.2202 704

34.2293 700

34.2384 742

34.2475 720

34.2565 739

34.2656 711

34.2747 739

34.2838 709

34.2929 694

34.3020 762

34.3111 688

34.3201 758

34.3292 712

34.3383 727

34.3474 741

34.3565 676

34.3656 771

34.3747 738

34.3837 713

34.3928 717

34.4019 709

34.4110 759

34.4201 729

34.4292 737

34.4382 740

34.4473 733

34.4564 710

34.4655 754

34.4746 733

34.4837 741

34.4928 701

34.5018 782

34.5109 737

34.5200 729

34.5291 683

34.5382 721

34.5473 739

34.5563 713

34.5654 720

34.5745 773

34.5836 731

34.5927 694

34.6018 755

34.6109 759

34.6199 704

34.6290 702

34.6381 745

34.6472 711

34.6563 679

34.6654 727

34.6745 685

34.6835 724

34.6926 676

34.7017 722

34.7108 705

34.7199 723

34.7290 712

34.7380 726

34.7471 734

34.7562 717

34.7653 683

34.7744 699

34.7835 702

34.7926 677

34.8016 745

34.8107 694

34.8198 691

34.8289 710

34.8380 714

34.8471 725

34.8561 713

34.8652 671

34.8743 702

34.8834 725

34.8925 680

34.9016 657

34.9107 696

34.9197 690

34.9288 662

34.9379 661

34.9470 660

34.9561 689

34.9652 686

34.9743 707

34.9833 693

34.9924 671

35.0015 700

35.0106 723

35.0197 662

35.0288 684

35.0378 713

35.0469 742

35.0560 717

35.0651 680

35.0742 662

35.0833 669

35.0924 725

35.1014 696

35.1105 713

35.1196 670

35.1287 697

35.1378 773

35.1469 716

35.1559 706

35.1650 663

35.1741 686

35.1832 697

35.1923 691

35.2014 681

35.2105 702

35.2195 686

35.2286 738

35.2377 688

35.2468 676

35.2559 710

35.2650 736

35.2741 730

35.2831 672

35.2922 704

35.3013 665

35.3104 730

35.3195 663

35.3286 643

35.3376 679

35.3467 695

35.3558 678

35.3649 675

35.3740 688

35.3831 632

35.3922 665

35.4012 737

35.4103 673

35.4194 674

35.4285 701

35.4376 662

35.4467 704

35.4557 717

35.4648 697

35.4739 677

35.4830 660

35.4921 689

35.5012 715

35.5103 725

35.5193 653

35.5284 727

35.5375 676

35.5466 721

35.5557 680

35.5648 669

35.5739 698

35.5829 670

35.5920 695

35.6011 734

35.6102 704

35.6193 690

35.6284 695

35.6374 685

35.6465 720

35.6556 691

35.6647 718

35.6738 672

35.6829 653

35.6920 697

35.7010 716

35.7101 707

35.7192 712

35.7283 746

35.7374 774

35.7465 690

35.7555 688

35.7646 722

35.7737 731

35.7828 715

35.7919 692

35.8010 662

35.8101 760

35.8191 702

35.8282 727

35.8373 753

35.8464 758

35.8555 723

35.8646 787

35.8737 713

35.8827 720

35.8918 755

35.9009 708

35.9100 767

35.9191 693

35.9282 726

35.9372 760

35.9463 725

35.9554 798

35.9645 716

35.9736 726

35.9827 745

35.9918 738

36.0008 725

36.0099 728

36.0190 746

36.0281 713

36.0372 774

36.0463 711

36.0553 768

36.0644 740

36.0735 741

36.0826 783

36.0917 763

36.1008 743

36.1099 757

36.1189 769

36.1280 781

36.1371 767

36.1462 746

36.1553 800

36.1644 792

36.1735 785

36.1825 776

36.1916 784

36.2007 772

36.2098 819

36.2189 811

36.2280 754

36.2370 740

36.2461 743

36.2552 852

36.2643 808

36.2734 814

36.2825 810

36.2916 805

36.3006 795

36.3097 782

36.3188 822

36.3279 830

36.3370 793

36.3461 846

36.3551 799

36.3642 780

36.3733 876

36.3824 800

36.3915 826

36.4006 820

36.4097 757

36.4187 775

36.4278 847

36.4369 822

36.4460 812

36.4551 781

36.4642 767

36.4733 765

36.4823 794

36.4914 789

36.5005 773

36.5096 764

36.5187 785

36.5278 742

36.5368 759

36.5459 757

36.5550 798

36.5641 785

36.5732 773

36.5823 781

36.5914 749

36.6004 808

36.6095 763

36.6186 720

36.6277 802

36.6368 747

36.6459 727

36.6549 767

36.6640 793

36.6731 747

36.6822 778

36.6913 737

36.7004 737

36.7095 741

36.7185 823

36.7276 741

36.7367 715

36.7458 793

36.7549 744

36.7640 696

36.7731 727

36.7821 740

36.7912 705

36.8003 696

36.8094 723

36.8185 748

36.8276 742

36.8366 742

36.8457 684

36.8548 714

36.8639 738

36.8730 742

36.8821 687

36.8912 710

36.9002 716

36.9093 724

36.9184 730

36.9275 704

36.9366 663

36.9457 733

36.9547 724

36.9638 712

36.9729 767

36.9820 740

36.9911 645

37.0002 702

37.0093 720

37.0183 653

37.0274 689

37.0365 683

37.0456 624

37.0547 712

37.0638 686

37.0729 714

37.0819 716

37.0910 681

37.1001 714

37.1092 700

37.1183 718

37.1274 662

37.1364 659

37.1455 647

37.1546 663

37.1637 652

37.1728 653

37.1819 708

37.1910 697

37.2000 668

37.2091 639

37.2182 672

37.2273 707

37.2364 651

37.2455 653

37.2546 655

37.2636 692

37.2727 619

37.2818 687

37.2909 619

37.3000 705

37.3091 693

37.3181 647

37.3272 670

37.3363 673

37.3454 694

37.3545 697

37.3636 670

37.3727 667

37.3817 652

37.3908 663

37.3999 693

37.4090 619

37.4181 700

37.4272 664

37.4362 659

37.4453 653

37.4544 658

37.4635 677

37.4726 642

37.4817 638

37.4908 642

37.4998 656

37.5089 681

37.5180 673

37.5271 651

37.5362 735

37.5453 659

37.5544 688

37.5634 659

37.5725 741

37.5816 639

37.5907 654

37.5998 694

37.6089 669

37.6179 687

37.6270 656

37.6361 676

37.6452 681

37.6543 697

37.6634 701

37.6725 627

37.6815 652

37.6906 640

37.6997 651

37.7088 652

37.7179 643

37.7270 640

37.7360 700

37.7451 653

37.7542 656

37.7633 702

37.7724 640

37.7815 685

37.7906 669

37.7996 635

37.8087 649

37.8178 579

37.8269 639

37.8360 671

37.8451 681

37.8542 633

37.8632 630

37.8723 665

37.8814 669

37.8905 647

37.8996 644

37.9087 661

37.9177 633

37.9268 674

37.9359 644

37.9450 615

37.9541 661

37.9632 657

37.9723 736

37.9813 651

37.9904 649

37.9995 672

38.0086 614

38.0177 692

38.0268 601

38.0358 641

38.0449 671

38.0540 649

38.0631 645

38.0722 632

38.0813 654

38.0904 621

38.0994 638

38.1085 643

38.1176 692

38.1267 623

38.1358 622

38.1449 675

38.1540 673

38.1630 654

38.1721 633

38.1812 669

38.1903 660

38.1994 627

38.2085 635

38.2175 628

38.2266 669

38.2357 631

38.2448 624

38.2539 679

38.2630 665

38.2721 667

38.2811 683

38.2902 639

38.2993 681

38.3084 686

38.3175 674

38.3266 677

38.3356 610

38.3447 655

38.3538 654

38.3629 659

38.3720 680

38.3811 660

38.3902 660

38.3992 615

38.4083 643

38.4174 685

38.4265 630

38.4356 698

38.4447 617

38.4538 672

38.4628 639

38.4719 665

38.4810 645

38.4901 700

38.4992 656

38.5083 696

38.5173 701

38.5264 617

38.5355 654

38.5446 651

38.5537 660

38.5628 628

38.5719 658

38.5809 710

38.5900 675

38.5991 651

38.6082 634

38.6173 680

38.6264 622

38.6354 629

38.6445 673

38.6536 651

38.6627 609

38.6718 624

38.6809 647

38.6900 624

38.6990 659

38.7081 630

38.7172 652

38.7263 619

38.7354 637

38.7445 662

38.7536 679

38.7626 702

38.7717 646

38.7808 671

38.7899 636

38.7990 630

38.8081 640

38.8171 646

38.8262 613

38.8353 646

38.8444 666

38.8535 674

38.8626 634

38.8717 675

38.8807 663

38.8898 672

38.8989 613

38.9080 660

38.9171 651

38.9262 624

38.9352 641

38.9443 618

38.9534 652

38.9625 684

38.9716 638

38.9807 646

38.9898 625

38.9988 650

39.0079 687

39.0170 640

39.0261 644

39.0352 677

39.0443 658

39.0534 635

39.0624 637

39.0715 600

39.0806 689

39.0897 673

39.0988 662

39.1079 611

39.1169 638

39.1260 727

39.1351 705

39.1442 666

39.1533 642

39.1624 638

39.1715 683

39.1805 638

39.1896 656

39.1987 643

39.2078 684

39.2169 674

39.2260 671

39.2350 666

39.2441 621

39.2532 634

39.2623 669

39.2714 643

39.2805 630

39.2896 654

39.2986 637

39.3077 629

39.3168 642

39.3259 658

39.3350 647

39.3441 655

39.3532 651

39.3622 620

39.3713 664

39.3804 641

39.3895 640

39.3986 672

39.4077 691

39.4167 632

39.4258 666

39.4349 668

39.4440 706

39.4531 613

39.4622 667

39.4713 660

39.4803 646

39.4894 637

39.4985 616

39.5076 606

39.5167 607

39.5258 624

39.5348 628

39.5439 635

39.5530 652

39.5621 653

39.5712 680

39.5803 642

39.5894 611

39.5984 646

39.6075 692

39.6166 695

39.6257 599

39.6348 683

39.6439 698

39.6530 596

39.6620 633

39.6711 643

39.6802 649

39.6893 651

39.6984 710

39.7075 661

39.7165 729

39.7256 600

39.7347 668

39.7438 635

39.7529 688

39.7620 647

39.7711 702

39.7801 691

39.7892 679

39.7983 589

39.8074 685

39.8165 649

39.8256 617

39.8346 609

39.8437 634

39.8528 640

39.8619 687

39.8710 649

39.8801 686

39.8892 644

39.8982 651

39.9073 646

39.9164 647

39.9255 593

39.9346 627

39.9437 681

39.9528 639

39.9618 648

39.9709 615

39.9800 642

39.9891 667

39.9982 646

40.0073 640

40.0163 621

40.0254 648

40.0345 612

40.0436 662

40.0527 621

40.0618 661

40.0709 638

40.0799 683

40.0890 633

40.0981 620

40.1072 657

40.1163 696

40.1254 654

40.1344 668

40.1435 626

40.1526 629

40.1617 634

40.1708 649

40.1799 625

40.1890 620

40.1980 669

40.2071 671

40.2162 664

40.2253 686

40.2344 609

40.2435 644

40.2526 640

40.2616 655

40.2707 627

40.2798 594

40.2889 624

40.2980 612

40.3071 657

40.3161 609

40.3252 660

40.3343 661

40.3434 642

40.3525 630

40.3616 608

40.3707 645

40.3797 668

40.3888 653

40.3979 644

40.4070 655

40.4161 655

40.4252 624

40.4342 617

40.4433 640

40.4524 681

40.4615 639

40.4706 649

40.4797 600

40.4888 682

40.4978 667

40.5069 586

40.5160 662

40.5251 596

40.5342 632

40.5433 640

40.5524 672

40.5614 681

40.5705 647

40.5796 646

40.5887 644

40.5978 613

40.6069 636

40.6159 617

40.6250 621

40.6341 655

40.6432 639

40.6523 653

40.6614 615

40.6705 597

40.6795 639

40.6886 643

40.6977 648

40.7068 619

40.7159 637

40.7250 629

40.7340 719

40.7431 695

40.7522 626

40.7613 617

40.7704 656

40.7795 624

40.7886 648

40.7976 681

40.8067 607

40.8158 661

40.8249 594

40.8340 617

40.8431 638

40.8522 626

40.8612 655

40.8703 653

40.8794 663

40.8885 658

40.8976 620

40.9067 627

40.9157 647

40.9248 648

40.9339 665

40.9430 655

40.9521 692

40.9612 651

40.9703 647

40.9793 656

40.9884 592

40.9975 666

41.0066 632

41.0157 653

41.0248 653

41.0338 626

41.0429 637

41.0520 642

41.0611 664

41.0702 648

41.0793 626

41.0884 654

41.0974 631

41.1065 656

41.1156 680

41.1247 657

41.1338 609

41.1429 671

41.1520 635

41.1610 670

41.1701 625

41.1792 680

41.1883 623

41.1974 651

41.2065 641

41.2155 686

41.2246 688

41.2337 614

41.2428 622

41.2519 689

41.2610 651

41.2701 577

41.2791 677

41.2882 618

41.2973 665

41.3064 682

41.3155 693

41.3246 673

41.3336 621

41.3427 599

41.3518 669

41.3609 624

41.3700 636

41.3791 699

41.3882 604

41.3972 646

41.4063 633

41.4154 634

41.4245 667

41.4336 662

41.4427 673

41.4518 594

41.4608 665

41.4699 623

41.4790 649

41.4881 648

41.4972 619

41.5063 615

41.5153 638

41.5244 624

41.5335 664

41.5426 686

41.5517 606

41.5608 683

41.5699 638

41.5789 654

41.5880 658

41.5971 600

41.6062 606

41.6153 611

41.6244 591

41.6334 679

41.6425 661

41.6516 672

41.6607 615

41.6698 637

41.6789 600

41.6880 651

41.6970 613

41.7061 628

41.7152 684

41.7243 661

41.7334 608

41.7425 646

41.7516 612

41.7606 640

41.7697 622

41.7788 706

41.7879 700

41.7970 708

41.8061 640

41.8151 668

41.8242 676

41.8333 632

41.8424 650

41.8515 657

41.8606 639

41.8697 669

41.8787 680

41.8878 694

41.8969 610

41.9060 637

41.9151 621

41.9242 654

41.9332 635

41.9423 585

41.9514 672

41.9605 640

41.9696 674

41.9787 646

41.9878 648

41.9968 679

42.0059 624

42.0150 635

42.0241 657

42.0332 641

42.0423 642

42.0514 665

42.0604 675

42.0695 663

42.0786 667

42.0877 654

42.0968 636

42.1059 624

42.1149 640

42.1240 656

42.1331 630

42.1422 659

42.1513 641

42.1604 669

42.1695 643

42.1785 612

42.1876 644

42.1967 670

42.2058 697

42.2149 682

42.2240 651

42.2330 647

42.2421 606

42.2512 618

42.2603 634

42.2694 628

42.2785 616

42.2876 600

42.2966 663

42.3057 633

42.3148 666

42.3239 673

42.3330 683

42.3421 663

42.3512 668

42.3602 676

42.3693 629

42.3784 685

42.3875 672

42.3966 616

42.4057 663

42.4147 610

42.4238 623

42.4329 668

42.4420 696

42.4511 638

42.4602 651

42.4693 678

42.4783 645

42.4874 621

42.4965 678

42.5056 704

42.5147 661

42.5238 670

42.5328 677

42.5419 636

42.5510 682

42.5601 643

42.5692 672

42.5783 648

42.5874 697

42.5964 689

42.6055 662

42.6146 623

42.6237 638

42.6328 649

42.6419 661

42.6510 629

42.6600 722

42.6691 646

42.6782 657

42.6873 640

42.6964 637

42.7055 645

42.7145 678

42.7236 632

42.7327 651

42.7418 634

42.7509 674

42.7600 668

42.7691 664

42.7781 656

42.7872 679

42.7963 624

42.8054 671

42.8145 606

42.8236 634

42.8326 678

42.8417 635

42.8508 617

42.8599 663

42.8690 678

42.8781 675

42.8872 692

42.8962 684

42.9053 644

42.9144 630

42.9235 633

42.9326 614

42.9417 651

42.9508 646

42.9598 695

42.9689 650

42.9780 633

42.9871 668

42.9962 661

43.0053 636

43.0143 626

43.0234 714

43.0325 718

43.0416 657

43.0507 628

43.0598 644

43.0689 617

43.0779 660

43.0870 675

43.0961 685

43.1052 683

43.1143 594

43.1234 603

43.1324 654

43.1415 706

43.1506 676

43.1597 616

43.1688 664

43.1779 651

43.1870 660

43.1960 673

43.2051 676

43.2142 650

43.2233 632

43.2324 630

43.2415 672

43.2506 619

43.2596 642

43.2687 692

43.2778 630

43.2869 658

43.2960 665

43.3051 658
[truncated: 67,611 more chars]
